# Beyond comparisons of means: understanding changes in gene expression at the single-cell level

**DOI:** 10.1186/s13059-016-0930-3

**Published:** 2016-04-15

**Authors:** Catalina A. Vallejos, Sylvia Richardson, John C. Marioni

**Affiliations:** MRC Biostatistics Unit, Cambridge Institute of Public Health, Cambridge, UK; EMBL European Bioinformatics Institute, Wellcome Trust Genome Campus, Cambridge, UK; Cancer Research UK Cambridge Institute, University of Cambridge, Li Ka Shing Centre, Cambridge, UK

**Keywords:** Single-cell RNA-seq, Differential expression, Cellular heterogeneity

## Abstract

Traditional differential expression tools are limited to detecting changes in overall expression, and fail to uncover the rich information provided by single-cell level data sets. We present a Bayesian hierarchical model that builds upon BASiCS to study changes that lie beyond comparisons of means, incorporating built-in normalization and quantifying technical artifacts by borrowing information from spike-in genes. Using a probabilistic approach, we highlight genes undergoing changes in cell-to-cell heterogeneity but whose overall expression remains unchanged. Control experiments validate our method’s performance and a case study suggests that novel biological insights can be revealed. Our method is implemented in R and available at https://github.com/catavallejos/BASiCS.

**Electronic supplementary material:**

The online version of this article (doi:10.1186/s13059-016-0930-3) contains supplementary material, which is available to authorized users.

## Background

The transcriptomics revolution – moving from bulk samples to single-cell (SC) resolution – provides novel insights into a tissue’s function and regulation. In particular, single-cell RNA sequencing (scRNA-seq) has led to the identification of novel sub-populations of cells in multiple contexts [1–3]. However, compared to bulk RNA-seq, a critical aspect of scRNA-seq data sets is an increased cell-to-cell variability among the expression counts. Part of this variance inflation is related to biological differences in the expression profiles of the cells (e.g., changes in mRNA content and the existence of cell sub-populations or transient states), which disappears when measuring bulk gene expression as an average across thousands of cells. Nonetheless, this increase in variability is also due in part to *technical noise* arising from the manipulation of small amounts of starting material, which is reflected in weak correlations between technical replicates [4]. Such technical artifacts are confounded with genuine transcriptional heterogeneity and can mask the biological signal.

Among others, one objective of RNA-seq experiments is to characterize transcriptional differences between pre-specified populations of cells (given by experimental conditions or cell types). This is a key step for understanding a cell’s fate and functionality. In the context of bulk RNA-seq, two popular methods for this purpose are edgeR [5] and DESeq2 [6]. However, these are not designed to capture features that are specific to scRNA-seq data sets. In contrast, SCDE [7] has been specifically developed to deal with scRNA-seq data sets. All of these methods target the detection of *differentially expressed genes* based on log-fold changes (LFCs) of overall expression between the populations. However, restricting the analysis to changes in overall expression does not take full advantage of the rich information provided by scRNA-seq. In particular – and unlike bulk RNA-seq – scRNA-seq can also reveal information about cell-to-cell expression heterogeneity. Critically, traditional approaches will fail to highlight genes whose expression is less stable in any given population but whose overall expression remains unchanged between populations.

More flexible approaches, capable of studying changes that lie beyond comparisons of means, are required to characterize differences between distinct populations of cells better. In this article, we develop a quantitative method to fill this gap, allowing the identification of genes whose cell-to-cell heterogeneity pattern changes between pre-specified populations of cells. In particular, genes with less variation in expression levels within a specific population of cells might be under more stringent regulatory control. Additionally, genes having increased biological variability in a given population of cells could suggest the existence of additional sub-groups within the analyzed populations. To the best of our knowledge, this is the first probabilistic tool developed for this purpose in the context of scRNA-seq analyses. We demonstrate the performance of our method using control experiments and by comparing expression patterns of mouse embryonic stem cells (mESCs) between different stages of the cell cycle.

## Results and discussion

### A statistical model to detect changes in expression patterns for scRNA-seq data sets

We propose a statistical approach to compare expression patterns between *P* pre-specified populations of cells. It builds upon BASiCS [8], a Bayesian model for the analysis of scRNA-seq data. As in traditional differential expression analyses, for any given gene *i*, changes in overall expression are identified by comparing population-specific expression rates $\mu ^{(p)}_{i}$ (*p*=1,…,*P*), defined as the relative abundance of gene *i* within the cells in population *p*. However, the main focus of our approach is to assess differences in biological cell-to-cell heterogeneity between the populations. These are quantified through changes in population- and gene-specific biological *over-dispersion* parameters $\delta ^{(p)}_{i}$ (*p*=1,…,*P*), designed to capture residual variance inflation (after normalization and technical noise removal) while attenuating the well-known confounding relationship between mean and variance in count-based data sets [9] (a similar concept was defined in the context of bulk RNA-seq by [10], using the term *biological coefficient of variation*). Importantly, such changes cannot be uncovered by standard differential expression methods, which are restricted to changes in overall expression. Hence, our approach provides novel biological insights by highlighting genes that undergo changes in cell-to-cell heterogeneity between the populations despite the overall expression level being preserved.

To disentangle technical from biological effects, we exploit *spike-in* genes that are added to the lysis buffer and thence theoretically present at the same amount in every cell (e.g., the 92 ERCC molecules developed by the External RNA Control Consortium [11]). These provide an internal control or gold standard to estimate the strength of technical variability and to aid normalization. In particular, these control genes allow inference on cell-to-cell differences in mRNA content, providing additional information about the analyzed populations of cells [12]. These are quantified through changes between cell-specific normalizing constants $\phi ^{(p)}_{j}$ (for the *j*th cell within the *p*th population). Critically, as described in Additional file 1: Note S1 and Fig. S1, global shifts in mRNA content between populations do not induce spurious differences when comparing gene-specific parameters (provided the offset correction described in ‘Methods’ is applied).

**Fig. 1:**
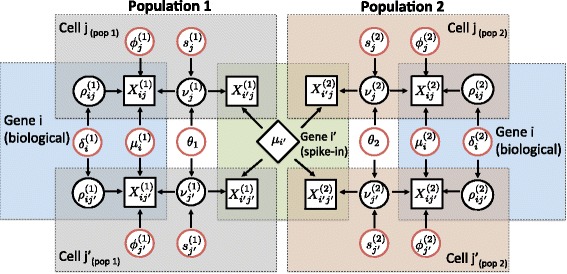
Graphical representation of our model for detecting changes in expression patterns (mean and over-dispersion) based on comparing two predefined population of cells. The diagram considers expression counts of two genes (*i* is biological and *i* ^′^ is technical) and two cells (*j*_*p*_ and $j^{\prime }_{p}$) from each population *p*=1,2. Observed expression counts are represented by *square nodes*. The central *rhomboid node* denotes the known input number of mRNA molecules for a technical gene *i* ^′^, which is assumed to be constant across all cells. The remaining *circular nodes* represent unknown elements, using *black* to denote random effects and *red* to denote model parameters (fixed effects) that lie on the top of the model’s hierarchy. Here, $\phi ^{(p)}_{j}$’s and $s^{(p)}_{j}$’s act as normalizing constants that are cell-specific and *θ*_*p*_’s are global over-dispersion parameters capturing technical variability, which affect the expression counts of all genes and cells within each population. In this diagram, $\nu ^{(p)}_{j}$’s and $\rho ^{(p)}_{ij}$’s represent random effects related to technical and biological variability components, whose variability is controlled by *θ*_*p*_’s and $\delta ^{(p)}_{i}$’s, respectively (see Additional file 1: Note 6.1). Finally, $\mu ^{(p)}_{i}$’s and $\delta ^{(p)}_{i}$’s, respectively, measure the overall expression of a gene *i* and its residual biological cell-to-cell over-dispersion (after normalization, technical noise removal and adjustment for overall expression) within each population. Colored areas highlight elements that are shared within a gene and/or cell. The latter emphasizes how our model borrows information across all cells to estimate parameters that are gene-specific and all genes to estimate parameters that are cell-specific. More details regarding the model setup can be found in the ‘Methods’ section of this article

A graphical representation of our model is displayed in Fig. 1 (based on a two-group comparison). It illustrates how our method borrows information across all cells and genes (biological transcripts and spike-in genes) to perform inference. Posterior inference is implemented via a Markov chain Monte Carlo (MCMC) algorithm, generating draws from the posterior distribution of all model parameters. Post-processing of these draws allows quantification of supporting evidence regarding changes in expression patterns (mean and over-dispersion). These are measured using a probabilistic approach based on tail posterior probabilities associated with decision rules, where a probability cut-off is calibrated through the expected false discovery rate (EFDR) [13].

Our strategy is flexible and can be combined with a variety of decision rules, which can be altered to reflect the biological question of interest. For example, if the aim is to detect genes whose overall expression changes between populations *p* and *p*^′^, a natural decision rule is $|\log (\mu ^{(p)}_{i}/\mu ^{(p')}_{i})| > \tau _{0}$, where *τ*_0_≥0 is an a priori chosen biologically significant threshold for LFCs in overall expression, to avoid highlighting genes with small changes in expression that are likely to be less biologically relevant [6, 14]. Alternatively, changes in biological cell-to-cell heterogeneity can be assessed using $|\log (\delta ^{(p)}_{i}/\delta ^{(p')}_{i})| > \omega _{0}$, for a given minimum tolerance threshold *ω*_0_≥0. This is the main focus of this article. As a default option, we suggest setting *τ*_0_=*ω*_0_=0.4, which roughly coincides with a 50 *%* increase in overall expression or over-dispersion in whichever group of cells has the largest value (this choice is also supported by the control experiments shown in this article). To improve the interpretation of the genes highlighted by our method, these decision rules can also be complemented by, e.g., requiring a minimum number of cells where the expression of a gene is detected.

More details regarding the model setup and the implementation of posterior inference can be found in ‘Methods’.

### Alternative approaches for identifying changes in mean expression

To date, most differential expression analyses of scRNA-seq data sets have borrowed methodology from bulk RNA-seq literature (e.g., DESeq2 [6] and edgeR [5]). However, such methods are not designed to capture features that are specific to SC-level experiments (e.g., the increased levels of technical noise). Instead, BASiCS, SCDE [7] and MAST [15] have been specifically developed with scRNA-seq data sets in mind. SCDE is designed to detect changes in mean expression while accounting for *dropout* events, where the expression of a gene is undetected in some cells due to biological variability or technical artifacts. For this purpose, SCDE employs a two-component mixture model where negative binomial and low-magnitude Poisson components model amplified genes and the background signal related to *dropout* events, respectively. MAST is designed to capture more complex changes in expression, using a hurdle model to study both changes in the proportion of cells where a gene is expressed above background and in the *positive expression mean*, defined as a conditional value – given than the gene is expressed above background levels. Additionally, MAST uses the fraction of genes that are detectably expressed in each cell (the cellular detection rate or CDR) as a proxy to quantify technical and biological artifacts (e.g., cell volume). SCDE and MAST rely on pre-normalized expression counts. Moreover, unlike BASiCS, SCDE and MAST use a definition of changes in expression mean that is conceptually different to what would be obtained based on a bulk population (which would consider all cells within a group, regardless of whether a gene is expressed above background or not).

The performance of these methods is compared in Additional file 1: Note S2 using real and simulated data sets. While control of the false discovery rate (FDR) is not well calibrated for BASiCS when setting *τ*_0_=0, this control is substantially improved when increasing the LFC threshold to *τ*_0_=0.4 – which is the default option we recommend (Additional file 1: Table S1). Not surprisingly, the higher FDR rates of BASiCS lead to higher sensitivity. In fact, our simulations suggest that BASiCS can correctly identify more genes that are differentially expressed than other methods. While this conclusion is based on synthetic data, it is also supported by the analysis of the cell-cycle data set described in [16] (see Additional file 1: Fig. S2), where we observe that SCDE and MAST fail to highlight a large number of genes for which a visual inspection suggests clear changes in overall expression (Additional file 1: Figs. S3 and S4). We hypothesize that this is partly due to conceptual differences in the definition of overall expression and, for MAST, the use of CDR as a covariate.

### Alternative approaches for identifying changes in heterogeneity of expression

To the best of our knowledge, BASiCS is the first probabilistic tool to quantify gene-specific changes in the variability of expression between populations of cells. Instead, previous literature has focused on comparisons based on the coefficient of variation (CV), calculated from pre-normalized expression counts (e.g., [17]), for which no quantitative measure of differential variability has been obtained. More recently, [9] proposed a mean-corrected measure of variability to avoid the confounding effect between mean expression and CV. Nonetheless, the latter was designed to compare expression patterns for sets of genes, rather than for individual genes.

Not surprisingly, our analysis suggests that a quantification of technical variability is critical when comparing variability estimates between cell populations (Additional file 1: Note S3 and Fig. S5). In particular, comparisons based on CV estimates can mask the biological signal if the strength of technical variability varies between populations.

### A control experiment: comparing single cells vs pool-and-split samples

To demonstrate the efficacy of our method, we use the control experiment described in [17], where single mESCs are compared against *pool-and-split* (P&S) samples, consisting of pooled RNA from thousands of mESCs split into SC equivalent volumes. Such a controlled setting provides a situation where substantial changes in overall expression are not expected as, on average, the overall expression of SCs should match the levels measured in P&S samples. Additionally, the design of P&S samples should remove biological variation, leading to a homogeneous set of samples. Hence, P&S samples are expected to show a genuine reduction in biological cell-to-cell heterogeneity compared to SCs.

Here, we display the analysis of samples cultured in a 2i media. Hyper-parameter values for $\mu _{i}^{(p)}$’s and $\delta _{i}^{(p)}$’s were set to $a^{2}_{\mu } = a^{2}_{\delta } = 0.5$, so that extreme LFC estimates are shrunk towards (−3,3) (see ‘Methods’). However, varying $a^{2}_{\mu }$ and $a^{2}_{\delta }$ leads to almost identical results (not shown), suggesting that posterior inference is in fact dominated by the data. In these data, expression counts correspond to the number of molecules mapping to each gene within each cell. This is achieved by using unique molecular identifiers (UMIs), which remove amplification biases and reduce sources of technical variation [18]. Our analysis includes 74 SCs and 76 P&S samples (same inclusion criteria as in [17]) and expression counts for 9378 genes (9343 biological and 35 ERCC spikes) defined as those with at least 50 detected molecules in total across all cells. The R code used to perform this analysis is provided in Additional file 2.

To account for potential batch effects, we allowed different levels of technical variability to be estimated in each batch (see Additional file 1: Note S4 and Fig. S6). Moreover, we also performed an independent analysis of each batch of cells. As seen in Additional file 1: Fig. S7, the results based on the full data are roughly replicated in each batch, suggesting that our strategy is able to remove potential artifacts related to this batch effect.

**Fig. 2:**
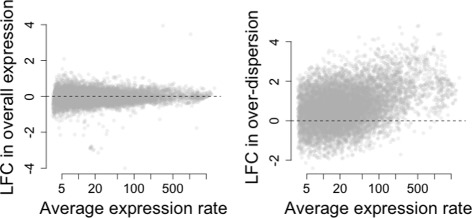
Estimated LFCs in expression (mean and over-dispersion) when comparing SCs vs P&S samples (2i serum culture). Posterior medians of LFC in (**a**) overall expression log(*μ* *i*(SC)/*μ* *i*(P&S)) and (**b**) biological over-dispersion log(*δ* *i*(SC)/*δ* *i*(P&S)) against the average between estimates of overall expression rates for SCs and P&S samples. Average values are defined as a weighted average between groups, with weights given by the number of samples within each group of cells. As expected, our analysis does not reveal major changes in expression levels between SC and P&S samples. In fact, the distribution of estimated LFCs in overall expression is roughly symmetric with respect to the origin. In contrast, we infer a substantial decrease in biological over-dispersion in the P&S samples. This is reflected by a skewed distribution of estimated LFCs in biological over-dispersion towards positive values. *LFC* log-fold change, *P&S* pool-and-split, *SC* single cell

**Fig. 3:**
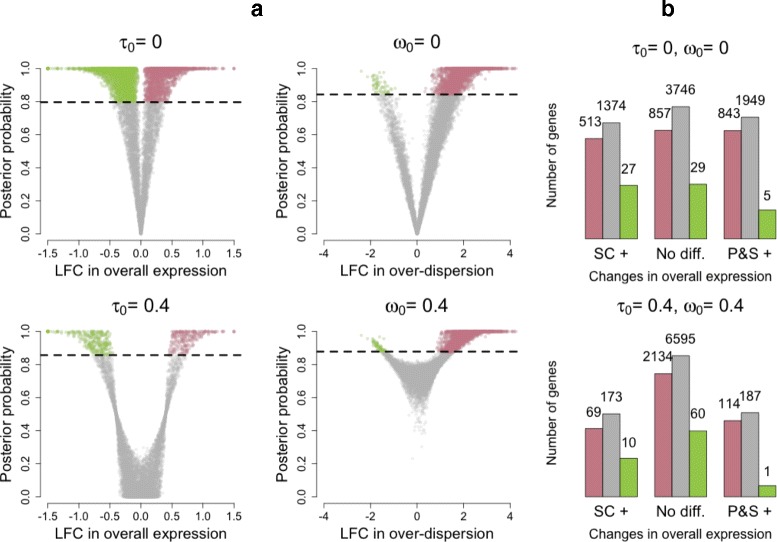
Summary of changes in expression patterns (mean and over-dispersion) for SCs vs P&S samples (EFDR = 5 %). **a** Volcano plots showing posterior medians of LFCs against estimated tail posterior probabilities. *Left panels* relate to the test where we assess if the absolute LFC in overall expression between SCs and P&S samples exceeds a minimum threshold *τ*_0_. Estimates for LFCs in overall expression are truncated to the range (−1.5,1.5). *Pink* and *green dots* represent genes highlighted to have higher overall expression in the SC and P&S samples, respectively. *Right panels* relate to the test where we assess if the absolute LFC in biological over-dispersion between SC and P&S samples exceeds a minimum threshold *ω*_0_. In all cases, *horizontal dashed lines* are located at probability cut-offs defined by EFDR = 5 %. *Pink* and *green dots* represent genes highlighted to have higher biological over-dispersion in the SC and P&S samples, respectively. **b** Bins in the horizontal axis summarize changes in overall expression between the groups. We use SC+ and P&S+ to denote that higher overall expression was detected in SC and P&S samples, respectively [the central group of *bars* (No diff.) corresponds to those genes where no significant differences were found]. *Colored bars* within each group summarize changes in biological over-dispersion between the groups. We use *pink* and *green bars* to denote higher biological over-dispersion in SC and P&S+ samples, respectively (and *gray* to denote no significant differences were found). The numbers of genes are displayed in log-scale. *LFC* log-fold change, *P&S* pool-and-split, *SC* single cell

As expected, our method does not reveal major changes in overall expression between SCs and P&S samples as the distribution of LFC estimates is roughly symmetric with respect to the origin (see Fig. 2a) and the majority of genes are not classified as differentially expressed at 5 % EFDR (see Fig. 3b). However, this analysis suggests that setting the minimum LFC tolerance threshold *τ*_0_ equal to 0 is too liberal as small LFCs are associated with high posterior probabilities of changes in expression (see Fig. 3a) and the number of differentially expressed genes is inflated (see Fig. 3b). In fact, counter-intuitively, 4710 genes (≈50 *%* of all analyzed genes) are highlighted to have a change in overall expression when using *τ*_0_=0. This is partially explained by the high nominal FDR rates displayed in Additional file 1: Note S2.1 where, for *τ*_0_=0, FDR is poorly calibrated when simulating under the null model. In addition, we hypothesize this heavy inflation is also due to small but statistically significant differences in expression that are not biologically meaningful. In fact, the number of genes whose overall expression changes is reduced to 559 (≈6 *%* of all analyzed genes) when setting *τ*_0_=0.4. As discussed earlier, this minimum threshold roughly coincides with a 50 % increase in overall expression and with the 90th percentile of empirical LFC estimates when simulating under the null model (no changes in expression). Posterior inference regarding biological over-dispersion is consistent with the experimental design, where the P&S samples are expected to have more homogeneous expression patterns. In fact, as shown in Fig. 2b, the distribution of estimated LFCs in biological over-dispersion is skewed towards positive values (higher biological over-dispersion in SCs). This is also supported by the results shown in Fig. 3b, where slightly more than 2000 genes exhibit increased biological over-dispersion in SCs and almost no genes (≈60 genes) are highlighted to have higher biological over-dispersion in the P&S samples (EFDR = 5 %). In this case, the choice of *ω*_0_ is less critical (within the range explored here). This is illustrated by the left panels in Fig. 3a, where tail posterior probabilities exceeding the cut-off defined by EFDR = 5 % correspond to similar ranges of LFC estimates.

### mESCs across different cell-cycle stages

Our second example shows the analysis of the mESC data set presented in [16], which contains cells where the cell-cycle phase is known (G1, S and G2M). After applying the same quality control criteria as in [16], our analysis considers 182 cells (59, 58 and 65 cells in stages G1, S and G2M, respectively). To remove genes with consistently low expression across all cells, we excluded those genes with less than 20 reads per million (RPM), on average, across all cells. After this filter, 5,687 genes remain (including 5,634 intrinsic transcripts, and 53 ERCC spike-in genes). The R code used to perform this analysis is provided in Additional file 3.

**Fig. 4:**
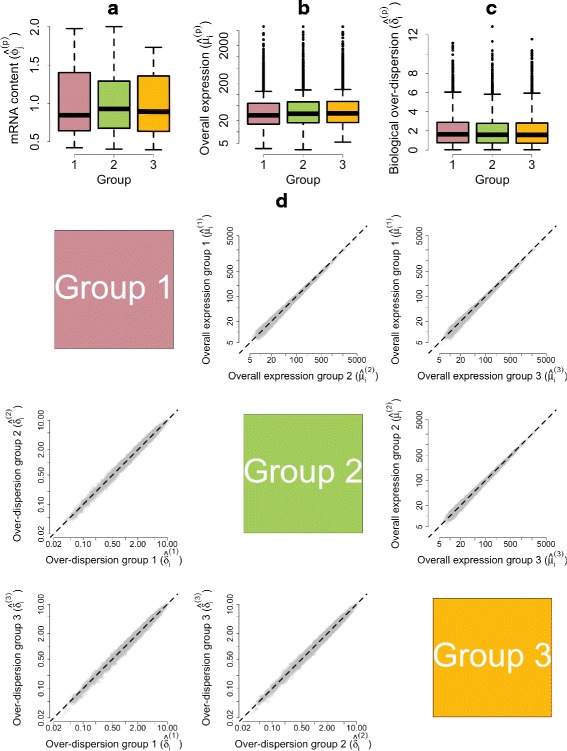
Posterior estimates of model parameters based on random permutations of the mESC cell-cycle data set. For a single permuted data set: **a** Empirical distribution of posterior medians for mRNA content normalizing constants $\phi _{j_{p}}$ across all cells. **b** Empirical distribution of posterior medians for gene-specific expression rates *μ*_*ip*_ across all genes. **c** Empirical distribution of posterior medians for gene-specific biological over-dispersion parameters *δ*_*ip*_ across all genes. **d** As an average across ten random permutations. *Upper diagonal panels* compare estimates for gene-specific expression rates *μ*_*ip*_ between groups of cells. *Lower diagonal panels* compare gene-specific biological over-dispersion parameters *δ*_*ip*_ between groups of cells

As a proof of concept, to demonstrate the efficacy of our approach under a negative control, we performed permutation experiments, where cell labels were randomly permuted into three groups (containing 60, 60 and 62 samples, respectively). In this case, our method correctly infers that mRNA content as well as gene expression profiles do not vary across groups of randomly permuted cells (Fig. 4).

**Fig. 5:**
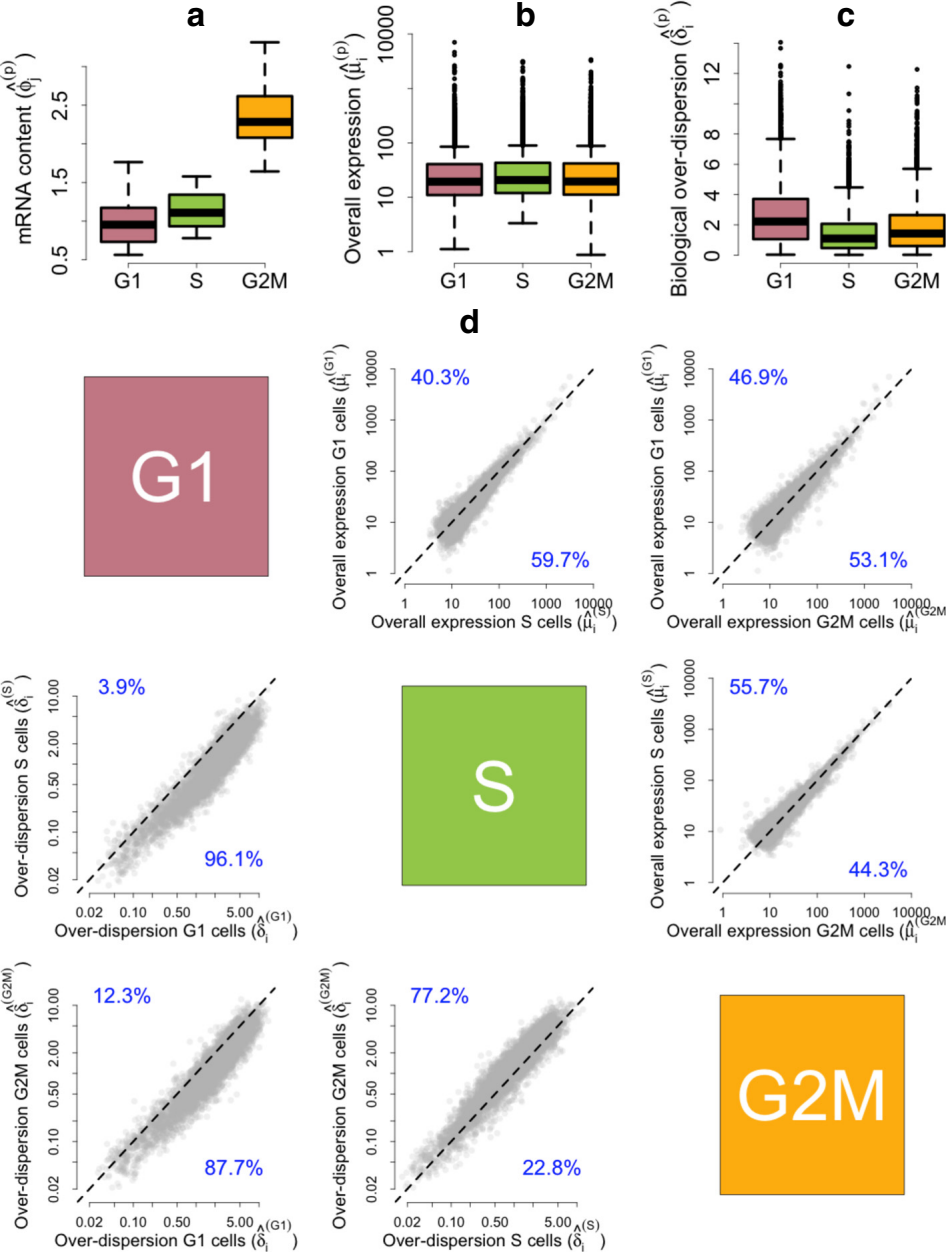
Posterior estimates of model parameters for mESCs across different cell-cycle phases. **a** Empirical distribution of posterior medians for mRNA content normalizing constants $\phi ^{(p)}_{j}$ across all cells. **b** Empirical distribution of posterior medians for gene-specific expression rates $\mu ^{(p)}_{i}$ across all genes. **c** Empirical distribution of posterior medians for gene-specific biological over-dispersion parameters $\delta ^{(p)}_{i}$ across all genes. **d** *Upper diagonal panels* compare estimates for gene-specific expression rates $\mu ^{(p)}_{i}$ between groups of cells. *Lower diagonal panels* compare gene-specific biological over-dispersion parameters $\delta ^{(p)}_{i}$ between groups of cells. While our results suggest there are no major shifts in mean expression between cell-cycle stages, our results suggest a substantial decrease in biological over-dispersion when cells move from G1 to the S phase, followed by a slight increase after the transition from S to the G2M phase (to give a rough quantification of this statement, panel (**d**) includes the percentage of point estimates that lie on each side of the *diagonal line*)

**Fig. 6:**
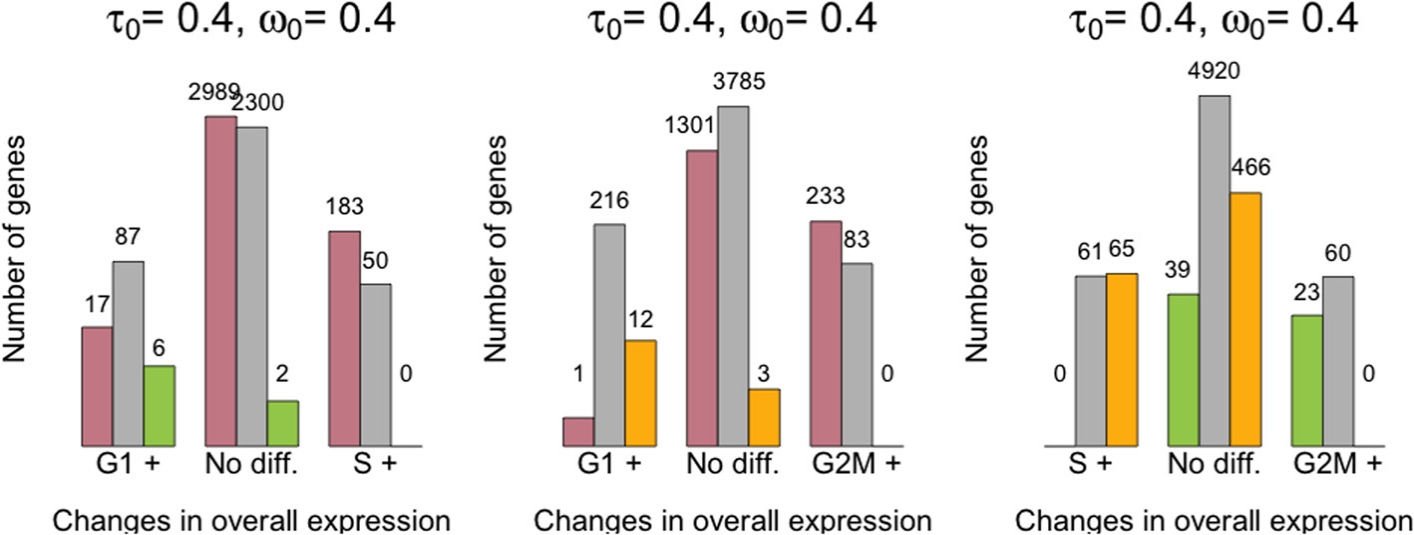
Summary of changes in expression patterns (mean and over-dispersion) for the mESC cell-cycle data set (EFDR = 5 %). Bins in the horizontal axis summarize changes in overall expression between each pair of groups. We use G1+, S+ and G2M+ to denote that higher overall expression was detected in cell-cycle phase G1, S and G2M, respectively [the central group of *bars* (No diff.) corresponds to those genes where no significant differences were found]. *Colored bars* within each group summarize changes in biological over-dispersion between the groups. We use *pink*, *green* and *yellow bars* to denote higher biological over-dispersion in cell-cycle phases G1, S and G2M, respectively (and *gray* to denote no significant differences were found). The numbers of genes are displayed in log-scale

As cells progress through the cell cycle, cellular mRNA content increases. In particular, our model infers that mRNA content is roughly doubled when comparing cells in G1 vs G2M, which is consistent with the duplication of genetic material prior to cell division (Fig. 5a). Our analysis suggests there are no major shifts in expression levels between cell-cycle stages (Fig. 5b and upper triangular panels in Fig. 5d). Nonetheless, a small number of genes are identified as displaying changes in overall expression between cell-cycle phases at 5 % EFDR for *τ*_0_=0.4 (Fig. 6). To validate our results, we performed gene ontology (GO) enrichment analysis within those genes classified as differentially expressed between cell-cycle phases (see Additional file 3). Not surprisingly, we found an enrichment of mitotic genes among the 545 genes classified as differentially expressed between G1 and G2M cells. In addition, the 209 differentially expressed genes between S and G2M are enriched for regulators of cytokinesis, which is the final stage of the cell cycle where a progenitor cell divides into two daughter cells [19].

Our method suggests a substantial decrease in biological over-dispersion when cells move from G1 to the S phase, followed by a slight increase after the transition from S to the G2M phase (see Fig. 5c and the lower triangular panels in Fig. 5d). This is consistent with the findings in [19], where the increased gene expression variability observed in G2M cells is attributed to an unequal distribution of genetic material during cytokinesis and the S phase is shown to have the most stable expression patterns within the cell cycle. Here, we discuss GO enrichment of those genes whose overall expression rate remains constant (EFDR = 5 %, *τ*_0_=0.4) but that exhibit changes in biological over-dispersion between cell-cycle stages (EFDR = 5 %, *ω*_0_=0.4). Critically, these genes will not be highlighted by traditional differential expression tools, which are restricted to differences in overall expression rates. For example, among the genes with higher biological over-dispersion in G1 with respect to the S phase, we found an enrichment of genes related to protein dephosphorylation. These are known regulators of the cell cycle [20]. Moreover, we found that genes with lower biological over-dispersion in G2M cells are enriched for genes related to DNA replication checkpoint regulation (which delays entry into mitosis until DNA synthesis is completed [21]) relative to G1 cells and mitotic cytokinesis when comparing to S cells. Both of these processes are likely to be more tightly regulated in the G2M phase. A full table with GO enrichment analysis of the results described here is provided in Additional file 3.

## Conclusions

Our method provides a quantitative tool to study changes in gene expression patterns between pre-specified populations of cells. Unlike traditional differential expression analyses, our model is able to identify changes in expression that are not necessarily reflected by shifts in the mean. This allows a better understanding of the differences between distinct populations of cells. In particular, we focus on the detection of genes whose residual biological heterogeneity (after normalization and technical noise removal) varies between the populations. This is quantified through biological over-dispersion parameters, which capture variance inflation with respect to the level that would be expected in a homogeneous population of cells while attenuating the well-known confounding relationship between mean and variance in count-based data sets. Despite this, several case studies (including the ones displayed in the manuscript and other examples analyzed throughout model development) suggest that – for a homogeneous population of cells – there is a strong relationship between posterior estimates of overall expression parameters $\mu ^{(p)}_{i}$ and over-dispersion parameters $\delta ^{(p)}_{i}$ (this is broken when analyzing heterogeneous populations, see Section S8 in [8]). This is illustrated in Additional file 1: Note S5 using the cell-cycle data set analyzed here (Additional file 1: Figs. S8 and S9). Due to this interplay between overall expression and over-dispersion, the interpretation of over-dispersion parameters $\delta ^{(p)}_{i}$ requires careful consideration. In particular, it is not trivial to interpret differences between $\delta ^{(p)}_{i}$’s when the $\mu ^{(p)}_{i}$’s also change. As a consequence, our analysis focuses on genes undergoing changes in over-dispersion but whose overall expression remains unchanged. This set of genes can provide novel biological insights that would not be uncovered by traditional differential expression analysis tools.

A decision rule to determine changes in expression patterns is defined through a probabilistic approach based on tail posterior probabilities and calibrated using the EFDR. The performance of our method was demonstrated using a controlled experiment where we recovered the expected behavior of gene expression patterns.

One caveat of our approach is the limited interpretation of the over-dispersion parameter when a gene is not expressed in a given population of cells or when the expression of a gene is only detected in a small proportion of cells (e.g., high expression in a handful of cells but no expression in the remaining cells). These situations will be reflected in low and high estimates of $\delta _{i}^{(p)}$, respectively. However, the biological relevance of these estimates is not clear. Hence, to improve the interpretation of the genes highlighted by our method, we suggest complementing the decision rules presented here by conditioning the results of the test on a minimum number of cells where the expression of a gene is detected.

Currently, our approach requires predefined populations of cells (e.g., defined by cell types or experimental conditions). However, a large number of scRNA-seq experiments involve a mixed population of cells, where cell types are not known a priori (e.g., [1–3]). In such cases, expression profiles can be used to *cluster* cells into distinct groups and to characterize markers for such sub-populations. Nonetheless, unknown group structures introduce additional challenges for normalization and quantification of technical variability since, e.g., noise levels can vary substantially between different cell populations. A future extension of our work is to combine the estimation procedure within our model with a clustering step, propagating the uncertainty associated with each of these steps into downstream analysis. In the meantime, if the analyzed population of cells contains a sub-population structure, we advise the user to cluster cells first (e.g., using a rank-based correlation, which is more robust to normalization), thus defining groups of cells that can be used as an input for BASiCS. This step will also aid the interpretation of model parameters that are gene-specific.

Until recently, most scRNA-seq data sets consisted of hundreds (and sometimes thousands) of cells. However, droplet-based approaches [22, 23] have recently allowed parallel sequencing of substantially larger numbers of cells in an effective manner. This brings additional challenges to the statistical analysis of scRNA-seq data sets (e.g., due to the existence of unknown sub-populations, requiring unsupervised approaches). In particular, current protocols do not allow the addition of technical spike-in genes. As a result, the deconvolution of biological and technical artifacts has become less straightforward. Moreover, the increased sample sizes emphasize the need for more computationally efficient approaches that are still able to capture the complex structure embedded within scRNA-seq data sets. To this end, we foresee the use of parallel programming as a tool for reducing computing times. Additionally, we are also exploring approximated posterior inference based, for example, on an integrated nested Laplace approximation [24].

Finally, our approach lies within a generalized linear mixed model framework. Hence, it can be easily extended to include additional information such as covariates (e.g., cell-cycle stage, gene length and GC content) and experimental design (e.g., batch effects) using fixed and/or random effects.

## Methods

### A statistical model to detect changes in expression patterns for scRNA-seq data sets

In this article, we introduce a statistical model for identifying genes whose expression patterns change between predefined populations of cells (given by experimental conditions or cell types). Such changes can be reflected via the overall expression level of each gene as well as through changes in cell-to-cell biological heterogeneity. Our method is motivated by features that are specific to scRNA-seq data sets. In this context, it is essential to normalize and remove technical artifacts appropriately from the data before extracting the biological signal. This is particularly critical when there are substantial differences in cellular mRNA content, amplification biases and other sources of technical variation. For this purpose, we exploit technical spike-in genes, which are added at the (theoretically) same quantity to each cell’s lysate. A typical example is the set of 92 ERCC molecules developed by the External RNA Control Consortium [11]. Our method builds upon BASiCS [8] and can perform comparisons between multiple populations of cells using a single model. Importantly, our strategy avoids stepwise procedures where data sets are normalized prior to any downstream analysis. This is an advantage over methods using pre-normalized counts, as the normalization step can be distorted by technical artifacts.

We assume that there are *P* groups of cells to be compared, each containing *n*_*p*_ cells (*p*=1,…,*P*). Let $X^{(p)}_{ij}$ be a random variable representing the expression count of a gene *i* (*i*=1,…,*q*) in the *j*th cell from group *p*. Without loss of generality, we assume the first *q*_0_ genes are biological and the remaining *q*−*q*_0_ are technical spikes. Extending the formulation in BASiCS, we assume that

(1) $$  \text{E}\left(X^{(p)}_{ij}\right) = \left\{ \begin{array}{ll} \phi^{(p)}_{j} s^{(p)}_{j} \mu^{(p)}_{i}, & i = 1, \ldots, q_{0}; \\ s^{(p)}_{j} \mu^{(p)}_{i}, & i = q_{0}+1, \ldots, q. \end{array} \right. \text{and}  $$

(2) $$ {\begin{aligned} \text{CV}^{2}\left(X^{(p)}_{ij}\right) = \left\{ \begin{array}{ll} (\phi^{(p)}_{j} s^{(p)}_{j} \mu^{(p)}_{i})^{-1} + \theta_{p} + \delta^{(p)}_{i} (\theta_{p} + 1), & i = 1, \ldots, q_{0}; \\ (s^{(p)}_{j} \mu^{(p)}_{i})^{-1} + \theta_{p}, & i = q_{0}+1, \ldots, q, \end{array} \right. \end{aligned}}  $$

with $\mu ^{(p)}_{i} \equiv \mu _{i}$ for *i*=*q*_0_+1,…,*q* and where CV stands for *coefficient of variation* (i.e., the ratio between standard deviation and mean). These expressions are the result of a Poisson hierarchical structure (see Additional file 1: Note S6.1). Here, $\phi ^{(p)}_{j}$’s act as cell-specific normalizing constants (fixed effects), capturing differences in input mRNA content across cells (reflected by the expression counts of intrinsic transcripts only). A second set of normalizing constants, $s^{(p)}_{j}$’s, capture cell-specific scale differences affecting the expression counts of all genes (intrinsic and technical). Among others, these differences can relate to sequencing depth, capture efficiency and amplification biases. However, a precise interpretation of the $s^{(p)}_{j}$’s varies across experimental protocols, e.g., amplification biases are removed when using UMIs [18]. In addition, *θ*_*p*_’s are global technical noise parameters controlling the over-dispersion (with respect to Poisson sampling) of all genes within group *p*. The overall expression rate of a gene *i* in group *p* is denoted by $\mu ^{(p)}_{i}$. These are used to quantify changes in the overall expression of a gene across groups. Similarly, the $\delta ^{(p)}_{i}$’s capture residual over-dispersion (beyond what is due to technical artifacts) of every gene within each group. These so-called biological over-dispersion parameters relate to heterogeneous expression of a gene across cells. For each group, stable *housekeeping-like* genes lead to $\delta ^{(p)}_{i} \approx 0$ (low residual variance in expression across cells) and highly variable genes are linked to large values of $\delta ^{(p)}_{i}$. A novelty of our approach is the use of $\delta ^{(p)}_{i}$ to quantify changes in biological over-dispersion. Importantly, this attenuates confounding effects due to changes in overall expression between the groups.

A graphical representation of this model is displayed in Fig. 1. To ensure identifiability of all model parameters, we assume that $\mu ^{(p)}_{i}$’s are known for the spike-in genes (and given by the number of spike-in molecules that are added to each well). Additionally, we impose the identifiability restriction

(3) $$  \frac{1}{n_{p}}\sum\limits_{j=1}^{n_{p}} \phi^{(p)}_{j} = 1, \text{for}~ p = 1,\ldots, P.  $$

Here, we discuss the priors assigned to parameters that are gene- and group-specific (see Additional file 1: Note S6.2 for the remaining elements of the prior). These are given by

(4) $$ \begin{aligned} \mu^{(p)}_{i} \stackrel{\text{iid}}{\sim} \log\text{N}\left(0, a^{2}_{\mu}\right)~ \text{and}&~ \delta^{(p)}_{i} \stackrel{\text{iid}}{\sim} {\log\text{N}}\left(0, a^{2}_{\delta}\right)~\\&\text{for}~ i = 1, \ldots, q_{0}. \end{aligned}  $$

Hereafter, without loss of generality, we simplify our notation to focus on two-group comparisons. This is equivalent to assigning Gaussian prior distributions for LFCs in overall expression (*τ*_*i*_) or biological over-dispersion (*ω*_*i*_). In such a case, it follows that

(5) $$ \begin{aligned} \tau_{i} &\equiv \log \left(\mu^{(1)}_{i} \big/ \mu^{(2)}_{i}\right) \sim~\text{N}\left(0, 2 a^{2}_{\mu}\right)~\text{and}~ \\&\!\!\!\!\!\omega_{i} \equiv \log\left(\delta^{(1)}_{i} \big/ \delta^{(2)}_{i}\right) \sim~\text{N}\left(0, 2 a^{2}_{\delta}\right). \end{aligned}  $$

Hence, our prior is *symmetric*, meaning that we do not a priori expect changes in expression to be skewed towards either group of cells. Values for $a^{2}_{\mu }$ and $a^{2}_{\delta }$ can be elicited using an *expected* range of values for LFC in expression and biological over-dispersion, respectively. The latter is particularly useful in situations where a gene is not expressed (or very lowly expressed) in one of the groups, where, e.g., LFCs in overall expression are undefined (the maximum likelihood estimate of *τ*_*i*_ would be ±*∞*, the sign depending on which group expresses gene *i*). A popular solution to this issue is the addition of *pseudo-counts*, where an arbitrary number is added to all expression counts (in all genes and cells). This strategy is also adopted in models that are based on log-transformed expression counts (e.g., [15]). While the latter guarantees that *τ*_*i*_ is well defined, it leads to artificial estimates for *τ*_*i*_ (see Table 1). Instead, our approach exploits an informative prior (indexed by $a^{2}_{\mu }$) to *shrink* extreme estimates of *τ*_*i*_ towards an expected range. This strategy leads to a meaningful shrinkage strength, which is based on prior knowledge. Importantly – and unlike the addition of pseudo-counts – our approach is also helpful when comparing biological over-dispersion between the groups. In fact, if a gene *i* is not expressed in one of the groups, this will lead to a non-finite estimate of *ω*_*i*_ (if all expression counts in a group are equal to zero, the corresponding estimate of the biological over-dispersion parameters would be equal to zero). Adding pseudo-counts cannot resolve this issue, but imposing an informative prior for *ω*_*i*_ (indexed by $a^{2}_{\omega }$) will shrink estimates towards the appropriate range.

**Table 1:** Synthetic example to illustrate the effect of addition of pseudo-counts over the estimation of LFCs in overall expression

	Empirical	Adding 0.5	Adding 1
	estimate	pseudo-counts	pseudo-count
Overall expression rate in population 1 ($\mu ^{(1)}_{i}$)	10	10.5	11
Overall expression rate in population 2 ($\mu ^{(2)}_{i}$)	0	0.5	1
LFC in overall expression 1 vs 2	+*∞*	3.04	2.40

For simplicity, we assume that normalization is not required so that pseudo-counts are linearly reflected in the overall expression rates. While pseudo-counts introduce an additive effect, LFC estimates measure changes on a multiplicative scale. Hence, addition of pseudo-counts leads to an artificial deflation of LFC estimates. As a consequence, such estimates cannot be meaningfully interpreted

Generally, posterior estimates of *τ*_*i*_ and *ω*_*i*_ are robust to the choice of $a^{2}_{\mu }$ and $a^{2}_{\delta }$, as the data is informative and dominates posterior inference. In fact, these values are only influential when shrinkage is needed, e.g., when there are zero total counts in one of the groups. In such cases, posterior estimates of *τ*_*i*_ and *ω*_*i*_ are dominated by the prior, yet the method described below still provides a tool to quantify evidence of changes in expression. As a default option, we use $a^{2}_{\mu } = a^{2}_{\delta } = 0.5$ leading to *τ*_*i*_,*ω*_*i*_∼ N(0,1). These default values imply that approximately 99 % of the LFCs in overall expression and over-dispersion are expected a priori to lie in the interval (−3,3). This range seems reasonable in light of the case studies we have explored. If a different range is expected, this can be easily modified by the user by setting different values for $a^{2}_{\mu }$ and $a^{2}_{\delta }$.

Posterior samples for all model parameters are generated via an adaptive Metropolis within a Gibbs sampling algorithm [25]. A detailed description of our implementation can be found in Additional file 1: Note S6.3.

### Post hoc correction of global shifts in input mRNA content between the groups

The identifiability restriction in Eq. 3 applies only to cells within each group. As a consequence, if they exist, global shifts in cellular mRNA content between groups (e.g., if all mRNAs were present at twice the level in one population related to another) are absorbed by the $\mu ^{(p)}_{i}$’s. To assess changes in the relative abundance of a gene, we adopt a two-step strategy where: (1) model parameters are estimated using the identifiability restriction in Eq. 3 and (2) global shifts in endogenous mRNA content are treated as a fixed *offset* and corrected post hoc. For this purpose, we use the sum of overall expression rates (intrinsic genes only) as a proxy for the total mRNA content within each group. Without loss of generality, we use the first group of cells as a reference population. For each population *p* (*p*=1,…,*P*), we define a population-specific offset effect:

(6) $$ \Lambda_{p} = \left(\sum\limits_{i=1}^{q_{0}} \mu^{(p)}_{i} \right) \bigg/ \left(\sum\limits_{i=1}^{q_{0}} \mu^{(1)}_{i} \right)  $$

and perform the following offset correction:

(7) $$ \begin{aligned} \tilde{\mu}^{(p)}_{i} &= \mu^{(p)}_{i} \big/ \Lambda_{p}, \quad\tilde{\phi}^{(p)}_{j} = \phi^{(p)}_{j} \times \Lambda_{p},\\& \!\! i = 1,\ldots,q_{0}; \quad\quad j_{p} = 1, \ldots, n_{p}. \end{aligned}  $$

This is equivalent to replacing the identifiability restriction in Eq. 3 by

(8) $$ \frac{1}{n_{p}}\sum\limits_{j=1}^{n_{p}} \phi^{(p)}_{j} = \Lambda_{p}, \quad \text{for}~ p = 1,\ldots, P.  $$

**Fig. 7:**
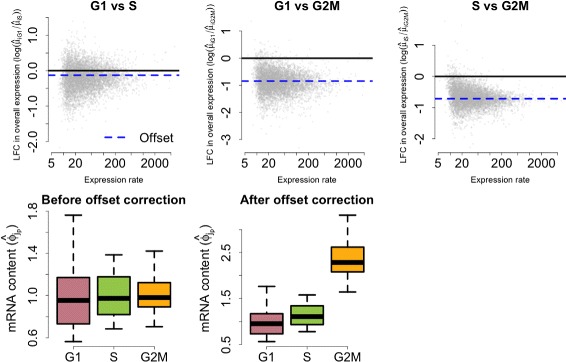
Post hoc offset correction for cell-cycle data set. *Upper panels* display posterior medians for LFC in overall expression against the weighted average between estimates of overall expression rates for G1, S and G2M cells (weights defined by the number of cells in each group). *Lower panels* illustrate the effect of the offset correction upon the empirical distribution of posterior estimates for mRNA content normalizing constants $\phi ^{(p)}_{j}$. These figures illustrate a shift in mRNA content throughout cell-cycle phases. In particular, our model infers that cellular mRNA is roughly duplicated when comparing G1 to G2M cells. *LFC* log-fold change

Technical details regarding the implementation of this post hoc offset correction are explained in Additional file 1: Note S6.4. The effect of this correction is illustrated in Fig. 7 using the cell-cycle data set described in the main text. As an alternative, we also explored the use of the ratio between the total intrinsic counts over total spike-in counts to define a similar offset correction based on

(9) $$ {\begin{aligned} \Lambda'_{p} = \left(\underset{j = 1, \ldots, n_{p}}{\text{median}} \left\{ \frac{\sum_{i=1}^{q_{0}} X^{(p)}_{ij}}{\sum_{i=q_{0} + 1}^{q} X^{(p)}_{ij}} \right\} \right) \bigg/ \left(\underset{j = 1, \ldots, n_{1}}{\text{median}} \left\{ \frac{\sum_{i=1}^{q_{0}} X^{(1)}_{ij}}{\sum_{i=q_{0} + 1}^{q} X^{(1)}_{ij}} \right\} \right). \end{aligned}}  $$

For the cell-cycle data set, both alternatives are equivalent. Nonetheless, the first option is more robust in cases where a large number of differentially expressed genes are present. Hereafter, we use $\mu ^{(p)}_{i}$ and $\phi ^{(p)}_{j}$ to denote $\tilde {\mu }^{(p)}_{i}$ and $\tilde {\phi }^{(p)}_{j}$, respectively.

### A probabilistic approach to quantify evidence of changes in expression patterns

A probabilistic approach is adopted, assessing changes in expression patterns (mean and over-dispersion) through a simple and intuitive scale of evidence. Our strategy is flexible and can be combined with a variety of decision rules. In particular, here we focus on highlighting genes whose absolute LFC in overall expression and biological over-dispersion between the populations exceeds minimum tolerance thresholds *τ*_0_ and *ω*_0_, respectively (*τ*_0_,*ω*_0_≥0), set a priori. The usage of such minimum tolerance levels for LFCs in expression has also been discussed in [14] and [6] as a tool to improve the biological significance of detected changes in expression and to improve upon FDRs.

For a given probability threshold $\alpha _{_{M}}$ ($0.5 < \alpha _{_{M}} < 1$), a gene *i* is identified as exhibiting a change in overall expression between populations *p* and *p*^′^ if

(10) $$ \begin{aligned} \pi^{M}_{i p p'} (\tau_{0}) &\equiv \text{P}(|\log(\mu^{(p)}_{i}/\mu^{(p')}_{i})| > \tau_{0} | \{\text{data}\}) > \alpha_{_{M}},\\& \quad i = 1,\ldots, q_{0}. \end{aligned}  $$

If *τ*_0_→0, ${\pi ^{M}_{i}}(\tau _{0}) \rightarrow 1$ becoming uninformative to detect changes in expression. As in [26], in the limiting case where *τ*_0_=0, we define

(11) $$ \pi^{M}_{i p p'} (0) = 2 \max\left\{\tilde{\pi}^{M}_{i p p'}, 1- \tilde{\pi}^{M}_{i p p'}\right\} - 1  $$

with

(12) $$ \tilde{\pi}^{M}_{i p p'} = \mathrm{P}\left(\log\left(\mu^{(p)}_{i}/\mu^{(p')}_{i}\right) > 0 \mid \{\text{data}\}\right).  $$

A similar approach is adopted to study changes in biological over-dispersion between populations *p* and *p*^′^, using

(13) $$ \pi^{D}_{i p p'} (\omega_{0}) \equiv \text{P}\left(|\log\left(\delta^{(p)}_{i}/\delta^{(p')}_{i}\right)| > \omega_{0} | \{\text{data}\}\right) > \alpha_{_{D}},  $$

for a fixed probability threshold $\alpha _{_{D}}$ ($0.5 < \alpha _{_{D}} < 1$). In line with Eqs. 11 and 12, we also define

(14) $$ \pi^{D}_{i p p'} (0) = 2 \max\left\{\tilde{\pi}^{D}_{i p p'}, 1-\tilde{\pi}^{D}_{i p p'}\right\} - 1  $$

with

(15) $$ \tilde{\pi}^{D}_{i p p'} = \text{P}\left(\log\left(\delta^{(p)}_{i}/\delta^{(p')}_{i} \right) > 0 \mid \{\text{data}\}\right).  $$

Evidence thresholds $\alpha _{_{M}}$ and $\alpha _{_{D}}$ can be fixed a priori. Otherwise, these can be defined by controlling the EFDR [13]. In our context, these are given by

(16) $$ \text{EFDR}_{\alpha_{_{M}}}(\tau_{0})= \frac{\sum_{i=1}^{q_{0}} \left(1-\pi^{M}_{i} (\tau_{0})\right) \text{I}\left(\pi^{M}_{i} (\tau_{0}) > \alpha_{_{M}}\right)}{\sum_{i=1}^{q_{0}} I\left(\pi^{M}_{i} (\tau_{0}) > \alpha_{_{M}}\right)}  $$

and

(17) $$ \text{EFDR}_{\alpha_{_{D}}}(\omega_{0})= \frac{\sum_{i=1}^{q_{0}} \left(1-\pi^{D}_{i} (\omega_{0})\right) \text{I}\left(\pi^{D}_{i} (\omega_{0}) > \alpha_{_{D}}\right)}{\sum_{i=1}^{q_{0}} I\left(\pi^{D}_{i} (\omega_{0}) > \alpha_{_{D}}\right)},  $$

where I(*A*)=1 if event *A* is true, 0 otherwise. Critically, the usability of this calibration rule relies on the existence of genes under both the null and the alternative hypothesis (i.e., with and without changes in expression). While this is not a practical limitation in real case studies, this calibration might fail to return a value in benchmark data sets (e.g., simulation studies), where there are no changes in expression. As a default, if EFDR calibration is not possible, we set $\alpha _{_{M}} = \alpha _{_{D}} = 0.90$.

The posterior probabilities in Eqs. 10, 11, 13 and 14 can be easily estimated – as a post-processing step – once the model has been fitted (see Additional file 1: Note S6.5). In addition, our strategy is flexible and can be easily extended to investigate more complex hypotheses, which can be defined post hoc, e.g., to identify those genes that show significant changes in cell-to-cell biological over-dispersion but that maintain a constant level of overall expression between the groups, or conditional decision rules where we require a minimum number of cells where the expression of a gene is detected.

### Software

Our implementation is freely available as an R package [27], using a combination of R and C++ functions through the Rcpp library [28]. This can be found in https://github.com/catavallejos/BASiCS, released under the GPL license.

## Availability of supporting data

All data sets analyzed in this article are publicly available in the cited references.

## Ethics

Not applicable.

## Additional files

Additional file 1 Supplementary material. Section S1 illustrates the interaction between cell- and gene-specific model parameters. Section S2 provides a comparative analysis of BASiCS and alternative methods regarding the detection of differentially expressed genes (changes in mean). Section S3 illustrates the usage of the coefficient of variation as a measure of cellular heterogeneity. Section S4 describes the treatment of potential batch effects used for the analysis of the data set provided by [17]. Section S5 illustrates the interplay between mean and over-dispersion parameters that is typically observed in homogeneous populations of cells. Section S6 contains additional details regarding the statistical model presented in this article and the implementation of Bayesian inference. (PDF 1443 kb)

Additional file 2 Data analysis (part 1). R code used to analyze the single cells vs pool-and-split samples data set. (PDF 3952 kb)

Additional file 3 Data analysis (part 2). R code used to analyze the cell-cycle data set. (PDF 3051 kb)

## Abbreviations

BASiCS: Bayesian analysis of single-cell sequencing data
bulk RNA-seq: bulk RNA sequencing
CDR: cellular detection rate
CV: coefficient of variation
EFDR: expected false discovery rate
ERCC: External RNA Control Consortium
FDR: false discovery rate
GO: gene ontology
LFC: log-fold change
MCMC: Markov chain Monte Carlo
mESC: mouse embryonic stem cell
P&S: pool-and-split
SC: single cell
scRNA-seq: single-cell RNA sequencing
UMI: unique molecular identifier
