# Supplementary material for: Beyond comparisons of means: understanding changes in gene expression at the single-cell level
Source: Genome Biol. 2016 Apr 15;17:70. doi: 10.1186/s13059-016-0930-3 (PMC4832562; doi:10.1186/s13059-016-0930-3)
Supplement: Supplementary file 1 — Supplementary material. Section S1 illustrates the interaction between cell- and gene-specific model parameters. Section S2 provides a comparative analysis of BASiCS and alternative methods regarding the detection of differentially expressed genes (changes in mean). Section S3 illustrates the usage of the coefficient of variation as a measure of cellular heterogeneity. Section S4 describes the treatment of potential batch effects used for the analysis of the data set provided by [17]. Section S5 illustrates the interplay between mean and over-dispersion parameters that is typically observed in homogeneous populations of cells. Section S6 contains additional details regarding the statistical model presented in this article and the implementation of Bayesian inference. (PDF 1443 kb) [file 13059_2016_930_MOESM1_ESM.pdf]

Supplementary material
Beyond comparisons of means: understanding changes in gene expression at the single-cell level

Catalina A. Vallejos[1 2], Sylvia Richardson[1] and John C. Marioni [2 3]

Contents

S1 Interaction between cell- and gene-specific model parameters **2**

S2 Comparison against alternative methods (changes in mean) **4**

S2.1 Simulation study to assess error rates 5

S2.2 Comparison based on Buettner et al. (2015) dataset 6

S3 CV as a measure of cellular heterogeneity **10**

S4 Batch effect in Grün et al. (2014) dataset **12**

S5 Interplay between mean and over-dispersion **14**

S6 Methodology **16**

S6.1 The model 16

S6.2 Prior specification 17

S6.3 Markov Chain Monte Carlo implementation 18

S6.4 Post-hoc offset correction of global shifts in mRNA content between groups . . . 19

S6.5 A probabilistic approach to quantify evidence of changes in expression patterns . 20

[1]MRC Biostatistics Unit
[2]EMBL-European Bioinformatics Institute
[3]Cancer Research UK Cambridge Institute

S1 Interaction between cell- and gene-specific model parameters

To shed light upon the interplay between cell- and gene-specific model parameters, we simulated data from the model implemented in BASiCS, to assess whether changes in cell-specific parameters can introduce spurious changes in parameters that are gene-specific. To provide a realistic simulation setting, we define the simulation parameters as the parameter estimates obtained for the Grün et al. (2014) dataset (single-cells group). In all simulated datasets, we kept the same values for parameters that are gene-specific (i.e. no changes in expression) but varied parameters that are cell-specific by introducing a fixed group-specific offset i.e. $\phi_j^{(p')} = c\phi_j^{(1)}$ and $s_j^{(p')} = cs_j^{(1)}$, where the first group of cells corresponds to the same parameter estimates as in the Grün et al. (2014) dataset (single-cells group). These results are summarized in the top 2 rows of Figure S1, which displays results based on a single simulated dataset for each offset value (these are representative of results obtained in additional simulations). Importantly, all simulations suggest that global changes in the scale of cell-specific normalising constants are captured by our estimates (e.g. first panel in top row of Figure S1) and they do not alter posterior inference about gene-specific parameters (after applying the offset effect correction described in Subsection S6.4). An additional set of simulations was created by varying the value of the global noise parameter θ_p, keeping all other parameters constant (bottom panels in Figure S1). These show that increasing the value of θ_p introduces noise in the estimation of the normalising constants $\phi_j^{(p)}$ (also true for $s_j^{(p)}$, not shown). However, these increased levels of noise do not seem to affect posterior inference for gene-specific parameters.

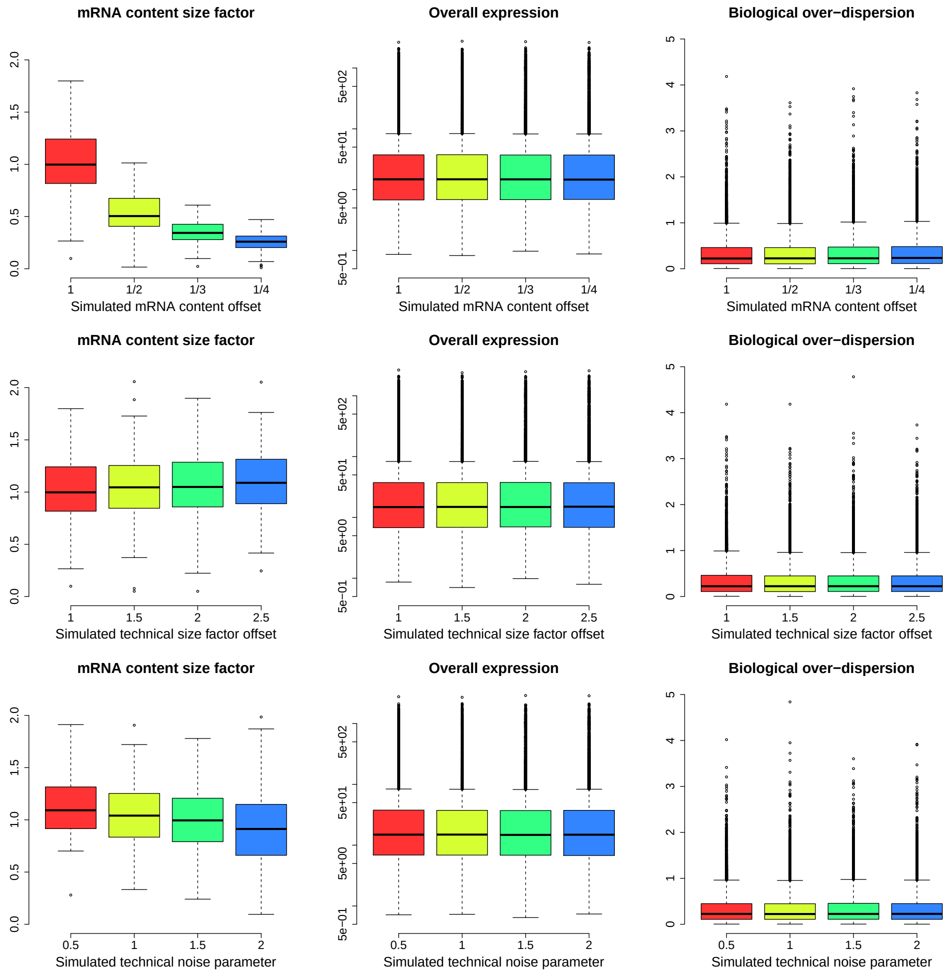

Figure S1: Distribution of posterior medians of model parameters for simulated datasets based on the parameter estimates obtained for the Grün et al. (2014) dataset, after introducing artificial global offset effects on mRNA content size factors $\phi_j^{(p)}$ (upper panels), technical size factors $s_j^{(p)}$ (middle panels) and group-specific noise parameters θ_p (bottom panels). In all cases, gene-specific expression rates and over-dispersion parameters are kept constant across different simulated datasets. These results suggest that global changes in size factors do not induce spurious differences between gene-specific model parameters. While this figure contains results based on a single simulated dataset for each setting, the conclusion is reproduced when simulating other datasets (not shown). Finally, the bottom row suggests that while increasing the value of θ_p introduces noise in the estimation of the normalising constants $\phi_j^{(p)}$ (also true for $s_j^{(p)}$, not shown), these increased levels of noise do not seem to affect posterior inference for gene-specific parameters that are of primary interest.

S2 Comparison against alternative methods (changes in mean)

Here we use synthetic data (see Section S2.1) and real data (see Section S2.2) to compare the performance of BASiCS against alternative methods that are designed to detect differentially expressed (DE) genes, i.e. changes in mean, based on RNA-seq data. The methods considered for this comparison are DESeq2 (Love et al., 2014), edgeR (Robinson et al., 2010), SCDE (Kharchenko et al., 2014) and MAST (Finak et al., 2015).

For BASiCS, we defined posterior probability thresholds by setting EFDR equal to 5%. For DESeq2, edgeR, SCDE and MAST, results were calibrated to control type I error at 5%.

For MAST, we fitted the model twice. Firstly, as recommended in Finak et al. (2015), including the cellular detection rate (CDR, i.e. the fraction of genes that are detectably expressed in a cell) as a covariate and a second fit excluding this information.

For BASiCS, DESeq2 and edgeR, we set two different minimum log-fold change threshold values τ_0 (this is not possible for SCDE and MAST).

S2.1 Simulation study to assess error rates

To assess the control of error rates of BASiCS, DESeq2, edgeR, SCDE and MAST, we simulated datasets based on the structure of the Grün et al. (2014) dataset and the model described in Section S6.1, with the values of all model parameters equal to the estimates obtained for the single-cells group. To introduce changes in mean expression between the simulated groups of cells, we randomly selected 500 genes and sampled log-fold change values from a $\mathrm{N}(0, 2)$ distribution. The results of this simulations are summarised in Table S1. These results illustrate that the false discovery rate (FDR) is poorly calibrated in BASiCS when setting $\tau_0 = 0$, but that a substantial improvement is obtained when increasing this value to $\tau_0 = 0.4$. Not surprisingly, this results in increased sensitivity (true positive rate: TPR) as our method is able to correctly identify more differentially expressed genes than other methods. All methods lead similar specificity (true negative rate: TNR) levels, except when we set $\tau_0 = 0$ for BASiCS. Furthermore, altering the BASiCS decision rule can facilitate more conservative control of the False Discovery Rate, albeit this comes at the expense of decreased statistical power.

Method	Sensitivity (TPR)	Specificity (TNR)	FDR
BASiCS (changes in mean, $\tau_0 = 0$)	89.98%	73.95%	83.30%
BASiCS (changes in mean, $\tau_0 = 0.4$)	75.34%	99.27%	14.50%
DESeq2 ($\tau_0 = 0$)	79.02%	99.67%	6.61%
DESeq2 ($\tau_0 = 0.4$)	54.26%	100%	0.00%
edgeR ($\tau_0 = 0$)	79.54%	99.71%	6.12%
edgeR ($\tau_0 = 0.4$)	63.42%	100%	0.00%
SCDE	65.90%	100%	0.03%
MAST (hurdle component, CDR as covariate)	27.10%	99.99%	0.54%
MAST (hurdle component, without CDR)	34.86%	99.99%	0.37%

Table S1: Summary of simulation results as average values across 10 repetitions. All model parameters are set equal to the estimated obtained for the Grün et al. (2014) dataset, introducing artificial log-fold changes for 500 randomly selected genes (drawn from a $\mathrm{N}(0, 2)$ distribution) .

S2.2 Comparison based on Buettner et al. (2015) dataset

The number of DE genes detected by each method is summarised in Figure S2. Since only BASiCS, SCDE and MAST have been developed for the analysis of scRNA-seq datasets, we focus on the comparison between these methods. In line to what was observed for the simulated datasets, BASiCS tends to detect the highest number of DE genes, especially when setting $\tau_0 = 0$. In contrast, SCDE and MAST (with CDR as a covariate) appear to be the most conservative methods. To visualise the differences between these methods, Figures S3 and S4 display the non-overlapping sets of genes between the DE lists provided by BASiCS, SCDE and MAST (with CDR as covariate), focusing on G1 and S cells (figures related to other pairwise comparisons included in Additional File 3). These figures display the heatmap of *denoised counts* defined as

$$\tilde{X}_{ij} = \frac{X_{ij}}{(\hat{\phi}_j \hat{\nu}_j)}, \tag{S1}$$

where $\hat{\phi}_j$ and $\hat{\nu}_j$ denote posterior medians for ϕ_j and ν_j, respectively. These heatmaps suggest that SCDE and MAST fail to highlight a large amount of genes for which a visual inspection suggests clear changes in overall expression. We hypothesise that this is partly due to conceptual differences in the definition of overall expression and, in the case of MAST, the usage of CDR as a covariate.

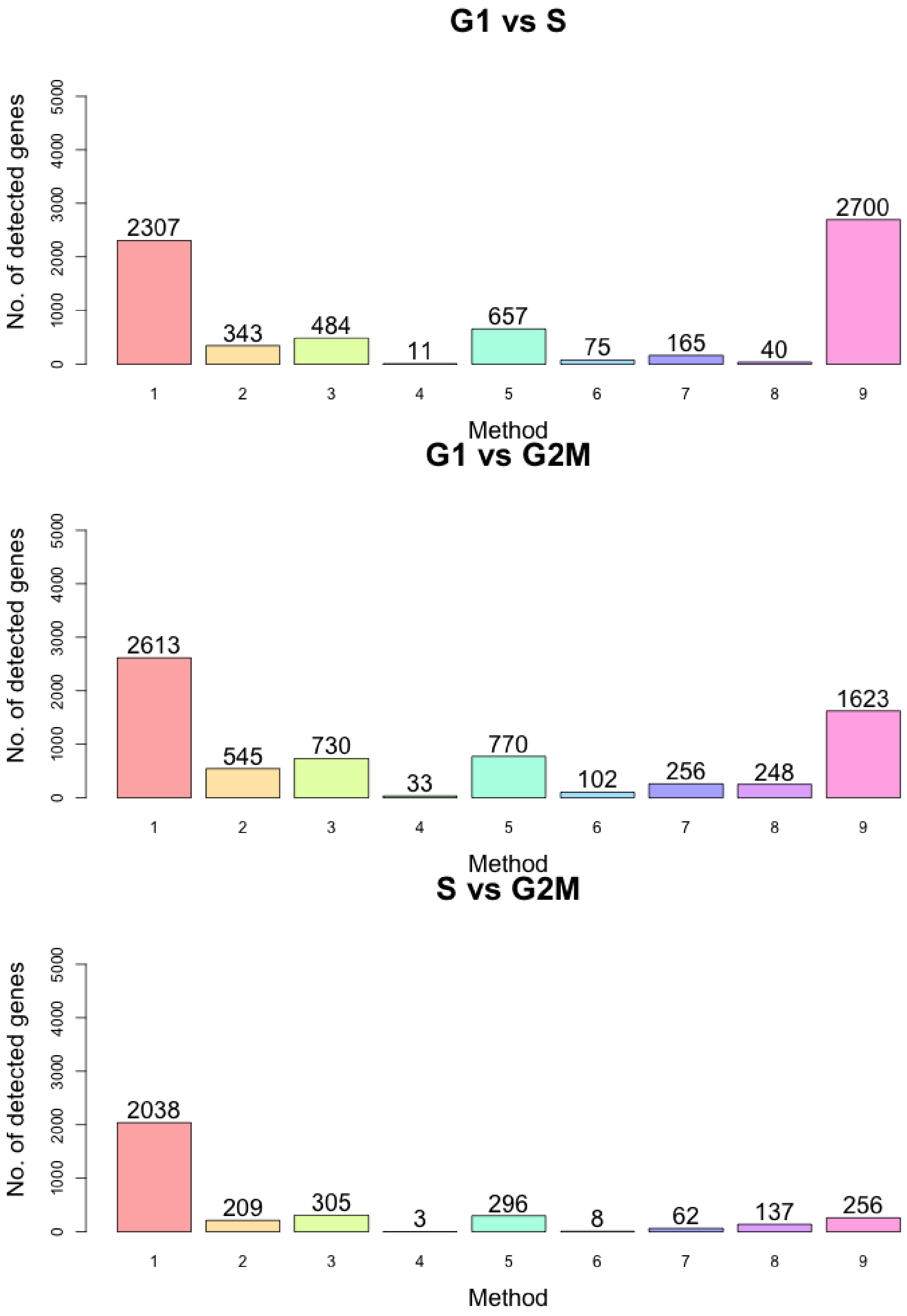

Figure S2: Changes in expression detected by different methods. 1: BASiCS change in mean ($\tau_0 = 0$); 2: BASiCS change in mean ($\tau_0 = 0.4$); 3: DESeq2 ($\tau_0 = 0$); 4: DESeq2 ($\tau_0 = 0.4$); 5: edgeR ($\tau_0 = 0$); 6: edgeR ($\tau_0 = 0.4$); 7: SCDE; 8: MAST hurdle model, CDR as covariate; 9: MAST hurdle model, without CDR as covariate.

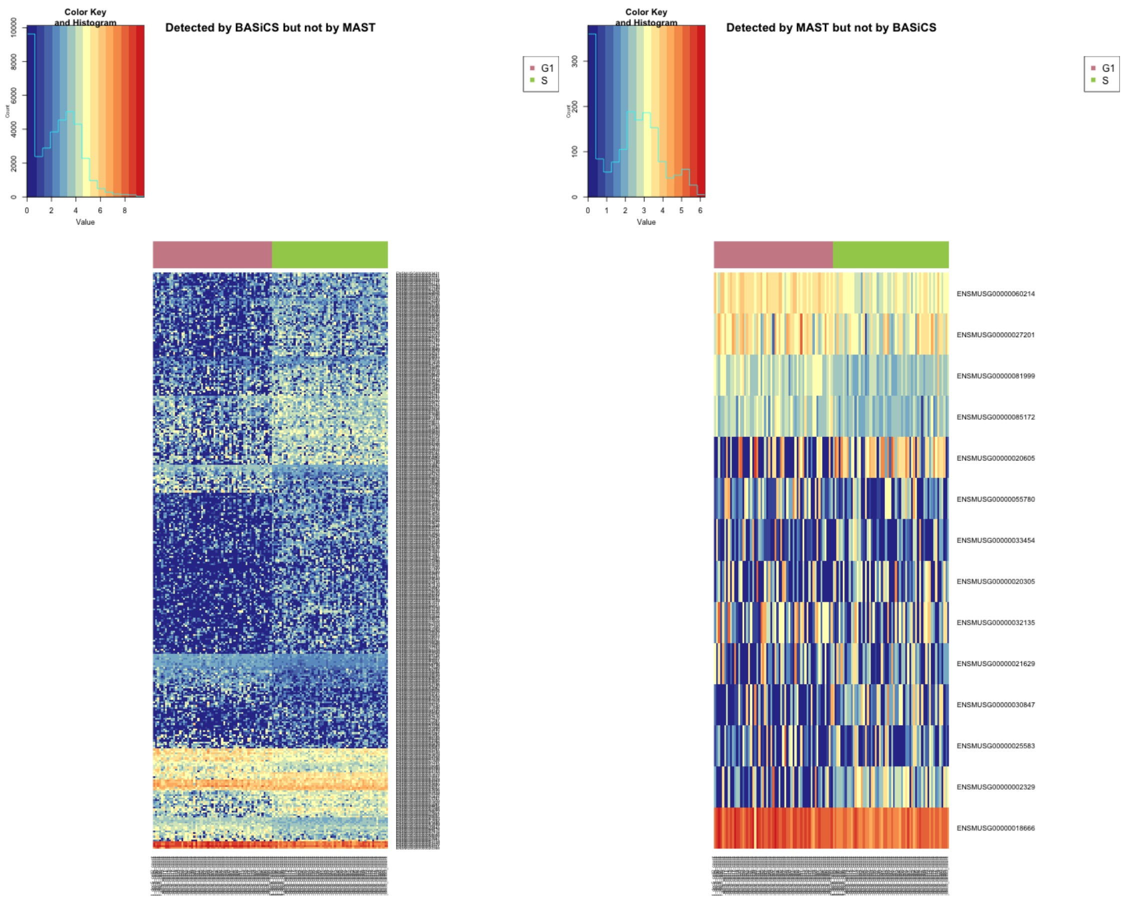

Figure S3: Heatmap of denoised counts of cells in G1 and S phase. Left: genes detected as differentially expressed by BASiCS (changes in mean) but not by MAST (hurdle model, CDR as covariate). These genes are enriched for platelet aggregation (p-value: 3.9×10^{-5}). Right: genes detected as differentially expressed by MAST (Hurdle model, CDR as covariate) but not by BASiCS (changes in mean).

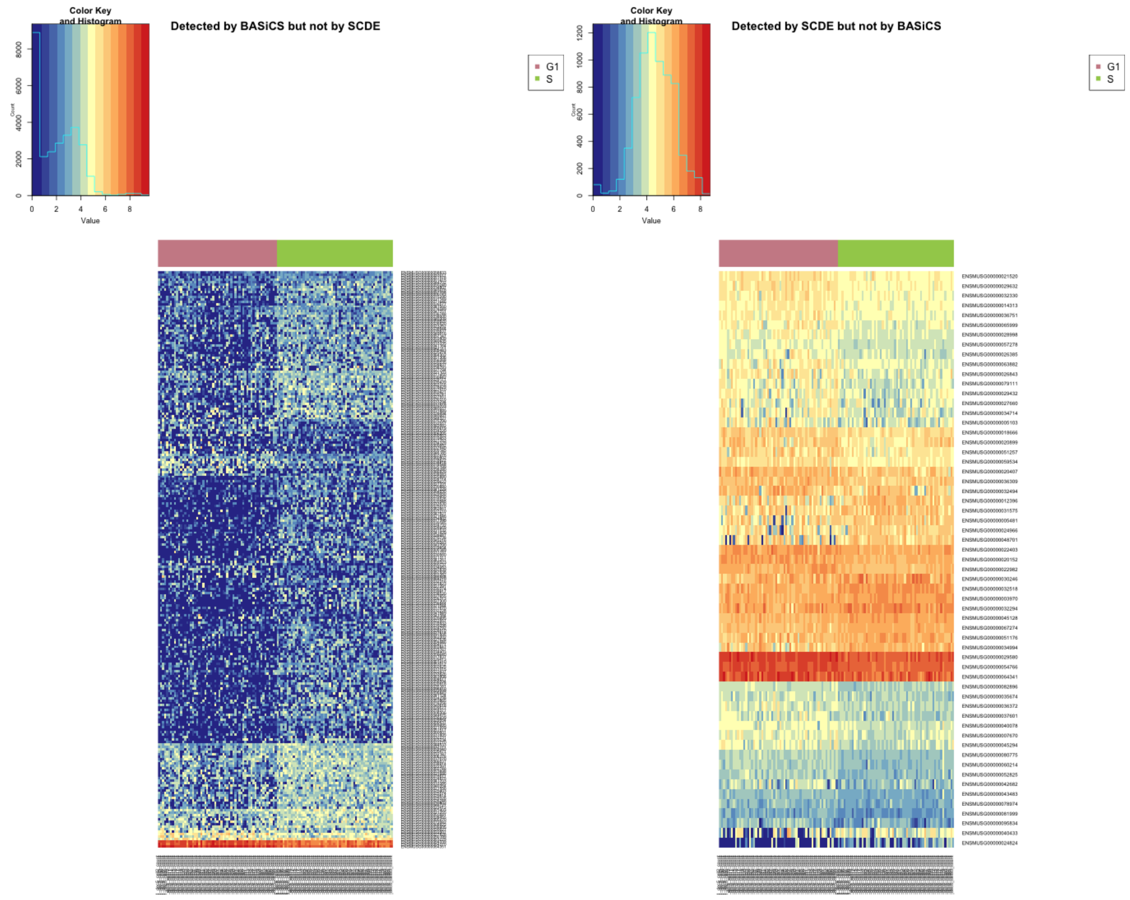

Figure S4: Heatmap of denoised counts of cells in G1 and S phase. Left: genes detected as differentially expressed by BASiCS (changes in mean) but not by SCDE. These genes are enriched for platelet aggregation (p-value: 8.2×10^{-4}). Right: genes detected as differentially expressed by SCDE but not by BASiCS (changes in mean).

S3 CV as a measure of cellular heterogeneity

To the best of our knowledge, BASiCS is the first probabilistic tool to quantify gene-specific changes in the variability of expression between populations of cells. Instead, previous literature has focused on comparisons based on the coefficient of variation (CV), calculated from pre-normalised expression counts (e.g. Grün et al., 2014). Here we illustrate what would be obtained when using this strategy for the analysis of the dataset presented in Buettner et al. (2015). Using these data, Figure S5 shows CV estimates that are based on reads per million (RPM) normalised counts. Against the evidence described in Darzynkiewicz et al. (1982), CV estimates suggest that cellular heterogeneity remains roughly constant throughout cell cycle. Our analysis suggests this is mainly due to the stronger levels of technical variability that are inferred for cells in S phase (not shown). These results highlight that, to compare variability in gene expression between populations of cells, it is critical to quantify the strength of technical variability.

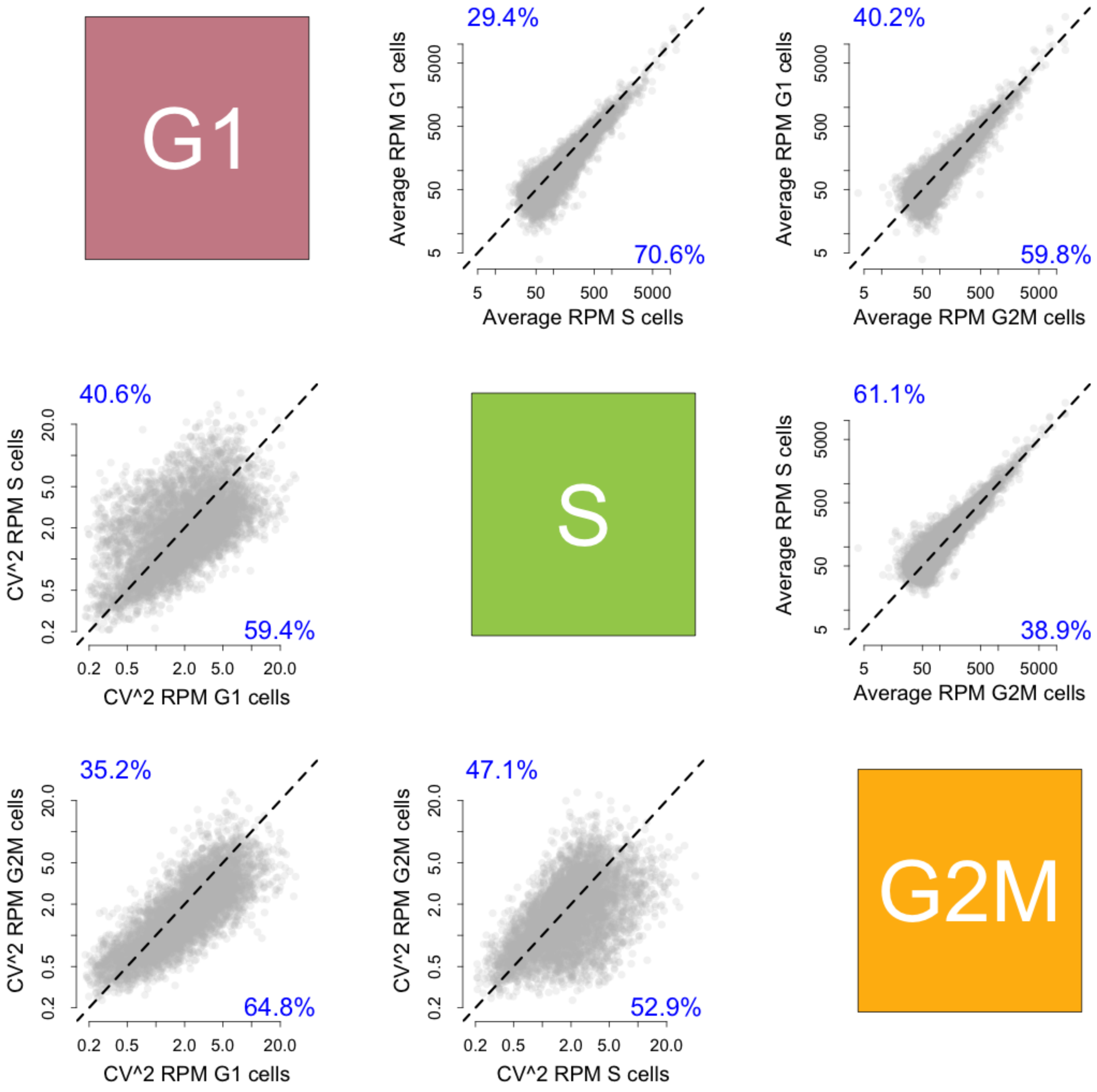

Figure S5: Cell cycle dataset. Upper diagonal panels compare estimates for gene-specific averages of (reads per million: RPM) normalised counts between groups of cells. Lower diagonal panels compare gene-specific CV^2 estimates based on normalised counts. Blue numbers indicate the proportion of gene-specific point estimates that are located above and below the diagonal line.

S4 Batch effect in Grün et al. (2014) dataset

As shown in Table S2, the samples contained in the Grün et al. (2014) datasets have been sequenced in two separate batches. Since the spike-in genes have been added at the same concentration in both batches they provide a control to remove technical artefacts between sequencing batches. Cell-specific size factors $s_j^{(p)}$ can capture changes affecting the mean of the expression counts. To allow distinct levels of noise between sequencing batches, we also allow group-specific global noise parameters θ_p to be batch-specific. As shown in Figure S6, posterior inference supports this choice since we observe elevated levels of noise in the first batch of cells in both groups of cells, which (if not accounted for) could affect posterior estimates of gene-specific parameters. To ensure that the shift in over-dispersion displayed in Figure 2 of the manuscript is not due to this batch effect, we also performed independent analysis of each batch of cells. As seen in Figure S7, results are roughly replicated in each batch, suggesting that our strategy is able to remove potential artefacts related to this batch effect.

	Single-cells	Pool-and-split
Batch 1	38	37
Batch 2	38	37

Table S2: Number of samples by group and sequencing batch (after quality control).

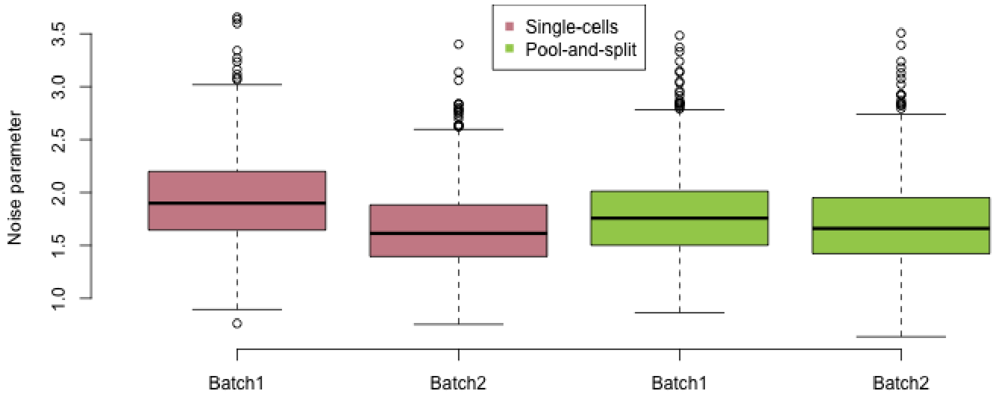

Figure S6: Boxplot of MCMC samples for technical noise parameters θ_p by group and batch.

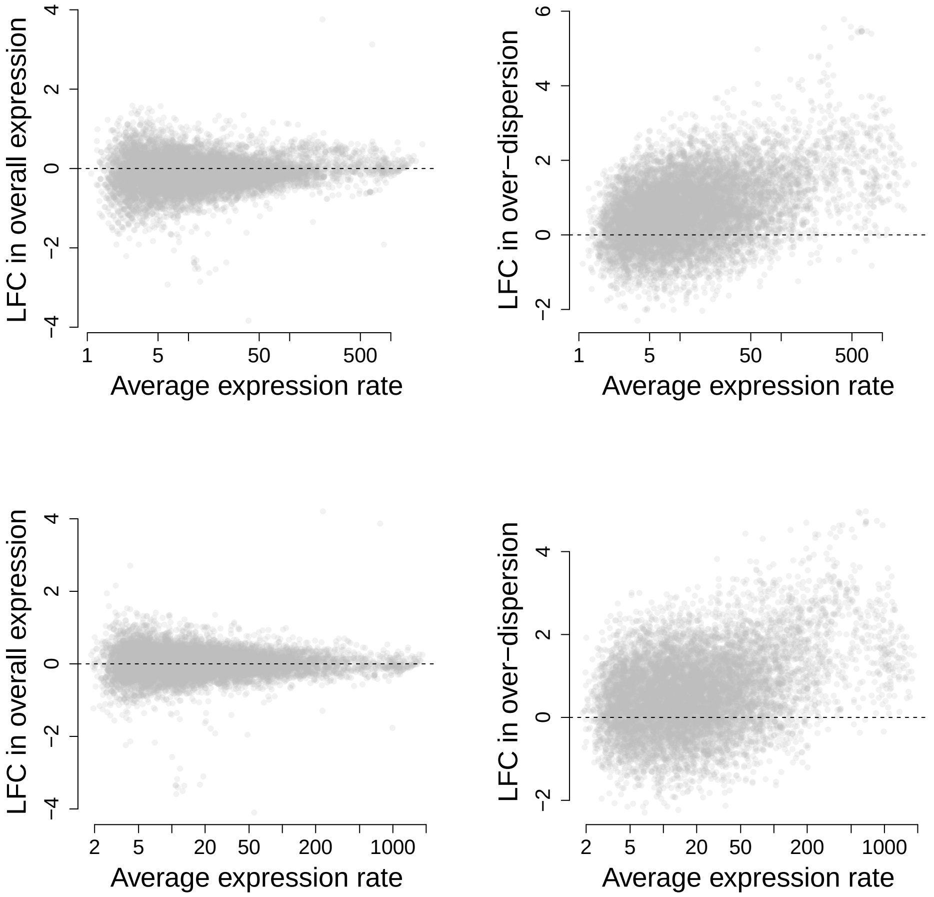

Figure S7: Estimated LFCs in expression (mean and over-dispersion) when comparing single-cells versus pool-and-split samples (*2i* serum culture), separated by sequencing batch. Upper panels: batch 1. Lower panels: batch 2.

S5 Interplay between mean and over-dispersion

In count-based datasets, there is a strong relationship between mean and variability measures. This is reflected in equation (2) of the manuscript, where the coefficient of variation of the expression counts depends upon the expected count value. This equation also shows that the over-dispersion parameters $\delta_i^{(p)}$ can be interpreted in terms of a residual coefficient of variation, after removing the value that would be expected for a fully homogeneous population of cells, where the observed variability would be explained by technical noise (Poisson sampling and residual technical over-dispersion). A similar concept was defined in the context of bulk RNA-seq by McCarthy et al. (2012), using the term *Biological Coefficient of Variation* (BCV). Despite this, several case studies (including the ones displayed in the manuscript and other examples analysed throughout model development) suggest that — for a homogeneous population of cells — there is a strong relationship between posterior estimates of overall expression parameters $\mu_i^{(p)}$ and over-dispersion parameters $\delta_i^{(p)}$ (this is broken when analysing heterogeneous populations, see S8 Text in Vallejos et al. (2015)). This is illustrated in Figure S8, where a negative trend between the posterior medians of $\mu_i^{(p)}$ and $\delta_i^{(p)}$ is observed for all three groups of cells, suggesting that — in a homogeneous population of cells — highly expressed genes tend to have tighter regulation. Note that while this trend is followed by the majority of genes, a subset of genes exhibits a different behaviour suggesting that this trend is not deterministic.

Due to this interplay between overall expression and over-dispersion, the interpretation of over-dispersion parameters $\delta_i^{(p)}$ requires careful consideration. In particular, it is not trivial to interpret differences between $\delta_i^{(p)}$'s when the $\mu_i^{(p)}$'s also change. In fact, as illustrated in Figure S9, there is a negative trend between LFCs in expression and LFCs in over-dispersion (i.e. if overall expression is higher in a given group of cells, this is typically accompanied by a decrease in over-dispersion). As a consequence, our analysis focuses on genes undergoing changes in over-dispersion but whose overall expression remains unchanged. This set of genes can provide novel biological insights that would not be uncovered by traditional differential expression analyses tools.

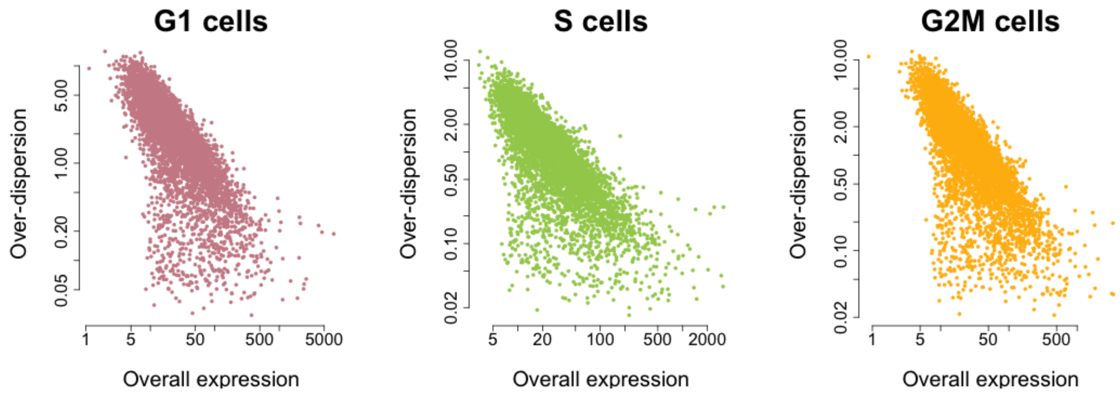

Figure S8: Posterior medians of overall expression parameters $\mu_i^{(p)}$ and over-dispersion parameters $\delta_i^{(p)}$ for cells in G1, S and G2M cell cycle phases.

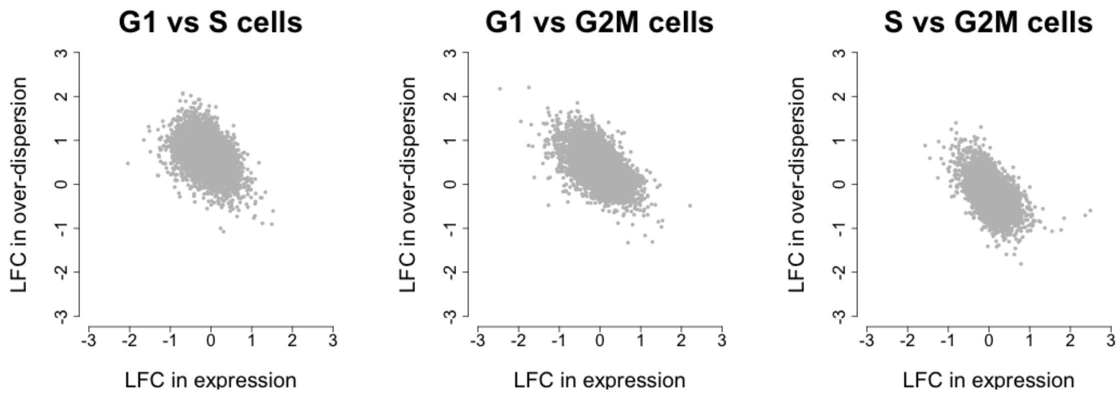

Figure S9: Posterior medians of log-fold changes in expression and over-dispersion for three pairwise comparisons between cell cycle stages.

S6 Methodology

S6.1 The model

We assume there are P groups of cells to be compared, each containing n_p cells ($p = 1, \ldots, P$). Let $X_{ij}^{(p)}$ be a random variable representing the expression count of a gene i ($i = 1, \ldots, q$) in the j-th cell from group p. To disentangle technical from biological effects, we exploit *spike-in* genes that are added to the lysis buffer and thence theoretically present at the same amount in every cell (e.g. the 92 ERCC molecules developed by the External RNA Control Consortium, Jiang et al., 2011). These provide an internal control or "gold standard", to estimate the strength of technical variability and to aid normalisation. Without loss of generality, we assume the first q_0 genes are biological and the remaining $q - q_0$ are technical spikes. Our model builds upon BASiCS (Vallejos et al., 2015), a Bayesian model for the analysis of single-cell RNA-seq (scRNA-seq) data. For each population of cells p ($p = 1, \ldots, P$), our extended model is given by

$$X_{ij}^{(p)} | \mu_i^{(p)}, \phi_j^{(p)}, \nu_j^{(p)}, \rho_{ij}^{(p)} \overset{ind}{\sim} \begin{cases} \text{Poisson}(\phi_j^{(p)} \nu_j^{(p)} \mu_i^{(p)} \rho_{ij}^{(p)}), & i = 1, \ldots, q_0, j = 1, \ldots, n_p; \\ \text{Poisson}(\nu_j^{(p)} \mu_i^{(p)}), & i = q_0 + 1, \ldots, q, j = 1, \ldots, n_p, \end{cases} \tag{S2}$$

$$\text{with} \quad \nu_j^{(p)} | s_j^{(p)}, \theta_p \overset{ind}{\sim} \text{Gamma}(1/\theta_p, 1/(s_j^{(p)} \theta_p)), \quad \rho_{ij}^{(p)} | \delta_i^{(p)} \overset{ind}{\sim} \text{Gamma}(1/\delta_i^{(p)}, 1/\delta_i^{(p)}) \tag{S3}$$

and $\mu_i^{(p)} \equiv \mu_i$ for $i = q_0 + 1, \ldots, q$. Here, $\phi_j^{(p)}$'s act as cell-specific normalising constants (fixed effects), to capture differences in input mRNA content between cells (reflected by the expression counts of intrinsic transcripts only). A second set of normalising constants, $s_j^{(p)}$'s, capture cell-specific scale differences affecting the expression counts of all genes (intrinsic and technical). Among others, these relate to sequencing depth, capture efficiency and amplification biases. However, a precise interpretation of the $s_{j_p}^{(p)}$'s varies across experimental protocols, e.g. amplification biases are removed when using unique molecular identifiers (Islam et al., 2014). The random effects $\nu_j^{(p)}$ (with $\text{E}(\nu_j^{(p)} | s_j^{(p)}, \theta_p) = s_j^{(p)}$ and $\text{Var}(\nu_j^{(p)} | s_j^{(p)}, \theta_p) = \left(s_j^{(p)}\right)^2 \theta_p$) capture unexplained technical noise, which leads to a variance inflation (with respect to Poisson sampling) of all expression counts within each group of cells. For each population, the strength of this technical component of variability is quantified through a single hyper-parameter θ_p, borrowing information across all genes and cells. A second set of random effects $\rho_{ij}^{(p)}$ (with $\text{E}(\rho_{ij}^{(p)} | \delta_i^{(p)}) = 1$ and $\text{Var}(\rho_{ij}^{(p)} | \delta_{ip}^{(p)}) = \delta_i^{(p)}$), capture heterogeneous expression of a gene across cells. This is quantified through the $\delta_i^{(p)}$'s, capturing residual over-dispersion (beyond what is due to technical artefacts) of every gene within each group. For each group, stable "*housekeeping-like*" genes lead to $\delta_i^{(p)} \approx 0$ (low residual variance in expression across cells) and highly variable genes are linked to large values of $\delta_i^{(p)}$. A novelty of our approach is the use of $\delta_i^{(p)}$'s to quantify changes

in biological over-dispersion. Importantly — and unlike the commonly used coefficient of variation — this avoids confounding effects due to changes in overall expression between the groups. Finally, the overall expression rate of a gene i in group p is denoted by $\mu_i^{(p)}$. These are used to quantify changes in the overall expression of a gene between groups of cells.

S6.2 Prior specification

We assume prior independence between all model parameters. In Vallejos et al. (2015), an improper *non-informative* prior was assigned to the overall expression rates $\mu_i^{(p)}$'s. However, it does not lead to a proper posterior distribution when all the expression counts of a gene are equal to zero at all cells within a population. The latter often occurs when comparing distinct populations of cells, where population-specific markers are likely to lie within this category. As an alternative, we assign a proper prior distribution to the overall expression rates $\mu_i^{(p)}$'s. This is given by

$$\mu_i^{(p)}, \overset{\text{iid}}{\sim} \text{log-N}(0, a_\mu^2) \quad \text{for } i = 1, \ldots, q_0, \tag{S4}$$

A similar prior is assigned to the biological over-dispersion parameters $\delta_i^{(p)}$'s using

$$\delta_i^{(p)}, \overset{\text{iid}}{\sim} \text{log-N}(0, a_\delta^2) \quad \text{for } i = 1, \ldots, q_0, \tag{S5}$$

As discussed in the manuscript, the latter is equivalent to assigning Gaussian prior distributions for log-fold changes (LFC) in overall expression or biological over-dispersion:

$$\tau_i \equiv \log(\mu_i^{(1)}/\mu_i^{(2)}) \sim \text{N}(0, 2a_\mu^2) \quad \text{and} \quad \omega_i \equiv \log(\delta_i^{(1)}/\delta_i^{(2)}) \sim \text{N}(0, 2a_\delta^2). \tag{S6}$$

Critically, these priors are symmetric with respect to the origin, meaning that we do not *a priori* expect changes in expression to be skewed towards either group of cells. Moreover, this prior specification is helpful in situations where a gene is not expressed (or very lowly expressed) in one of the groups, where the values of a_μ^2 and a_δ^2 allow shrinkage of LFC estimates towards an appropriate range (e.g. to avoid "infinite" LCF estimates when a gene has zero total counts within one population of cells). Importantly, genes for which expression was detected in all populations are not affected by the choice of these hyper-parameter values. As a default option we set $a_\mu^2 = a_\delta^2 = 1/2$, implying that approximately 99% of the log-fold changes in overall expression and over-dispersion are contained in the interval $(-3, 3)$. This range seems reasonable in light of the case studies we have explored. If a different range is expected, this can be easily modified by the user by setting different values for a_μ^2 and a_δ^2. For example, in the analysis of the single-cells versus pool-and split samples dataset (Grün et al., 2014), it would be reasonable to assign a more informative prior where LFC estimates are shrunk towards a smaller range,

e.g. $(-1,1)$ leading to $a_\mu^2 = a_\delta^2 = 1/6$. However, using this choice instead of the default prior led to almost identical results.

We also assign proper prior distributions to the remaining model parameters, using

$$
\begin{aligned}
s_j^{(p)} &\sim \text{Gamma}(a_s, b_s), \quad j = 1,\dots,n_p; p = 1,\dots,P && \text{(S7)}\\
\theta_p &\sim \text{Gamma}(a_\theta, b_\theta), \quad p = 1,\dots,P && \text{(S8)}\\
\Phi_p &\sim n_p\text{Dirichlet}(a_{\Phi_p}), \quad \Phi_p = (\phi_1^{(p)},\dots,\phi_{n_p}^{(p)})'; p = 1,\dots,P && \text{(S9)}
\end{aligned}
$$

By default, we set $a_s = b_s = a_\theta = b_\theta = 1$ and $a_{\Phi_p} = \mathbf{1}_{n_p}$, where $\mathbf{1}_{n_p}$ denotes an n_p-dimensional vector of ones.

S6.3 Markov Chain Monte Carlo implementation

Posterior inference is implemented via a Markov Chain Monte Carlo (MCMC) algorithm, generating draws from the posterior distribution of all model parameters. In particular, we use an Adaptive Metropolis within Gibbs Sampling algorithm (Roberts and Rosenthal, 2009), where the variance of the proposal distributions are internally tuned to achieve an optimal acceptance rate. However, sampling the random effects $\rho_{ij}^{(p)}$'s throughout the algorithm results in a slow convergence (despite allowing conjugate updates of other model parameters). This is particularly critical when the sample size increases. To overcome this problem, we implemented Bayesian inference based on the marginal model obtained after integrating out the $\rho_{ij}^{(p)}$'s, i.e. for each group of cells p and $j = 1,\dots,n_p$

$$
X_{ij}^{(p)} | \mu_i^{(p)}, \delta_i^{(p)}, \phi_j^{(p)}, \nu_j^{(p)}, \theta \sim \begin{cases} \text{Neg-Binomial}\left(\frac{1}{\delta_i^{(p)}}, \frac{\phi_j^{(p)}\nu_j^{(p)}\mu_i^{(p)}}{\phi_j^{(p)}\nu_j^{(p)}\mu_i^{(p)}+1/\delta_i^{(p)}}\right), & i = 1,\dots,q_0; \\ \text{Poisson}(\nu_j^{(p)}\mu_i^{(p)}), & i = q_0+1,\dots,q \end{cases} \tag{S10}
$$

for which the associated likelihood function is given by

$$
\left[\prod_{i=1}^{q_0}\prod_{p=1}^{P}\prod_{j=1}^{n_p} \frac{\Gamma(x_{ij}^{(p)} + 1/\delta_i^{(p)})}{\Gamma(1/\delta_i^{(p)}) x_{ij}^{(p)}!} \left(\frac{1/\delta_i^{(p)}}{\phi_j^{(p)}\nu_j^{(p)}\mu_i^{(p)} + 1/\delta_i^{(p)}}\right)^{1/\delta_i^{(p)}} \left(\frac{\phi_j^{(p)}\nu_j^{(p)}\mu_i^{(p)}}{\phi_j^{(p)}\nu_j^{(p)}\mu_i^{(p)} + 1/\delta_i^{(p)}}\right)^{x_{ij}^{(p)}}\right] \times \left[\prod_{i=q_0+1}^{q}\prod_{p=1}^{P}\prod_{j=1}^{n_p} \frac{(\nu_j^{(p)}\mu_i^{(p)})^{x_{ij}^{(p)}}}{x_{ij}^{(p)}!} e^{-\nu_j^{(p)}\mu_i^{(p)}}\right] \tag{S11}
$$

Under this specification, the full conditionals required for the implementation correspond to

$$\pi(\mu_i^{(p)}|\cdots) \propto \frac{\left(\mu_i^{(p)}\right)^{\sum_{j=1}^{n_p} x_{ij}^{(p)}}}{\prod_{j=1}^{n_p}(\phi_j^{(p)}\nu_j^{(p)}\mu_i^{(p)} + 1/\delta_i^{(p)})^{x_{ij}^{(p)}+1/\delta_i^{(p)}}} \times \exp\left\{-\frac{1}{2a_\mu^2}(\log(\mu_i^{(p)}))^2\right\}, \tag{S12}$$

$$\pi(\delta_i^{(p)}|\cdots) \propto \left[\prod_{j_p=1}^{n_p} \frac{\Gamma(x_{ij}^{(p)} + 1/\delta_i^{(p)})}{\Gamma(1/\delta_i^{(p)})} \frac{(1/\delta_i^{(p)})^{1/\delta_i^{(p)}}}{(\phi_j^{(p)}\nu_j^{(p)}\mu_i^{(p)} + 1/\delta_i^{(p)})^{x_{ij}^{(p)}+1/\delta_i^{(p)}}}\right] \times \exp\left\{-\frac{1}{2a_\delta^2}(\log(\delta_i^{(p)}))^2\right\}, \tag{S13}$$

$$\pi(s_j^{(p)}|\cdots) \propto \left(s_j^{(p)}\right)^{a_s-(1/\theta_p)-1} \exp\left\{-\frac{\nu_j^{(p)}}{s_j^{(p)}\theta_p} - s_j^{(p)} b_s\right\}, \tag{S14}$$

$$\pi(\nu_j^{(p)}|\cdots) \propto \left[\prod_{i=1}^{q_0} \frac{\left(\nu_j^{(p)}\right)^{x_{ij}^{(p)}}}{(\phi_j^{(p)}\nu_j^{(p)}\mu_i^{(p)} + \frac{1}{\delta_i^{(p)}})^{x_{ij}^{(p)}+\frac{1}{\delta_i^{(p)}}}}\right] \left[\prod_{i=q_0+1}^{q} \left(\nu_j^{(p)}\right)^{x_{ij}^{(p)}} e^{-\nu_j^{(p)}\mu_i}\right] \times$$
$$\left(\nu_j^{(p)}\right)^{(1/\theta_p)-1} e^{-\nu_j^{(p)}/(\theta_p s_j^{(p)})}, \tag{S15}$$

$$\pi(\theta_p|\cdots) \propto \frac{\left(\prod_{j_p=1}^{n_p}(\nu_j^{(p)}/s_j^{(p)})\right)^{1/\theta_p}}{\Gamma^{n_p}(1/\theta_p)} \theta_p^{a_\theta-(n_p/\theta_p)-1} e^{-(1/\theta_p)\sum_{j_p=1}^{n_p}(\nu_j^{(p)}/s_j^{(p)})-b_\theta\theta_p} \tag{S16}$$

$$\pi(\Phi_p|\cdots) \propto \frac{\prod_{i=1}^{q_0}\left(\phi_j^{(p)}\right)^{\sum_{j_p=1}^{n_p} x_{ij}^{(p)}}}{\prod_{i=1}^{q_0}\prod_{j_p=1}^{n_p}(\phi_j^{(p)}\nu_j^{(p)}\mu_i^{(p)} + 1/\delta_i^{(p)})^{x_{ij}^{(p)}+1/\delta_i^{(p)}}} \times \pi(\Phi_p), \text{ with } \pi(\Phi_p) \text{ as in (S9)}, \tag{S17}$$

for $i = 1, \ldots, q_0$, $j = 1, \ldots, n_p$ and $p = 1, \ldots, P$. To sample from the posterior distribution of all model parameters, we use Gaussian Random walks for these full conditionals (S12)-(S16) and Dirichlet proposals for the full conditional in (S17).

Our implementation is freely available as an R package (R Core Team, 2014), using a combination of R and C++ functions through the Rcpp library (Eddelbuettel et al., 2011). This can be found in https://github.com/catavallejos/BASiCS, released under the GPL license.

S6.4 Post-hoc offset correction of global shifts in mRNA content between groups

To ensure identifiability of all model parameters, we introduce the identifiability restriction

$$\frac{1}{n_p}\sum_{j=1}^{n_p}\phi_j^{(p)} = 1, \quad \text{for } p = 1, \ldots, P. \tag{S18}$$

This restriction does only apply to cells within each group. As a consequence, if they exist, global shifts in cellular mRNA content between groups (e.g. if all mRNAs where present at twice the level in one population related to another) are absorbed by the $\mu_i^{(p)}$'s. To correct this bias, we adopt the 2-step strategy described below.

(i) **Estimation step.** Model parameters are estimated under the identifiability restriction in (S18), using the MCMC algorithm described in Section S6.3. For each parameter, this algorithm generates a sample of N random draws from the associated posterior distribution. In particular, for each $\mu_i^{(p)}$ and $\phi_j^{(p)}$, we denote these samples by $\{\mu_i^{(p)1}, \ldots, \mu_i^{(p)N}\}$ and $\{\phi_j^{(p)1}, \ldots, \phi_j^{(p)N}\}$, respectively.

(ii) **Offset correction step.** Once the model has been fitted, global shifts in input mRNA content are treated as a fixed *offset* and corrected post-hoc. For this purpose, we use the sum of overall expression rates $\sum_{i=1}^{q_0} \mu_i^{(p)}$ (intrinsic genes only) as a proxy for the total mRNA content within each group. Without loss of generality, we use the first group of cells as a reference population and define population-specific offset effects as

$$\Lambda_p = \left(\sum_{i=1}^{q_0} \mu_i^{(p)} \right) \bigg/ \left(\sum_{i=1}^{q_0} \mu_i^{(1)} \right), \quad p = 1, \ldots, P \tag{S19}$$

To estimate these quantities, we firstly create MCMC samples for each Λ_p ($p = 1, \ldots, P$), denoted by $\{\Lambda_p^{(1)}, \ldots, \Lambda_p^{(N)}\}$ with $\Lambda_p^{(m)} = \sum_{i=1}^{q_0} \mu_i^{(p)m}$. Secondly, for each population p ($p = 1, \ldots, P$), we estimate these offset effects as the posterior medians of each Λ_p, i.e.

$$\hat{\Lambda}_p = \underset{m=1,\ldots,M}{\text{median}} \left\{ \Lambda_p^{(m)} \right\} \tag{S20}$$

Finally, offset corrected MCMC samples for each $\mu_i^{(p)}$ and $\phi_j^{(p)}$ are generated as

$$\hat{\mu}_{ip}^{(p)m} = \mu_i^{(p)m} / \hat{\Lambda}_p \quad \hat{\phi}_j^{(p)m} = \phi_j^{(p)m} \hat{\Lambda}_p, \tag{S21}$$

for $m = 1, \ldots, M$, $i = 1, \ldots, q_0$, $j = 1, \ldots, n_p$ and $p = 1, \ldots, P$. The test to compare expression patterns between groups of cells must be conducted using these *offset corrected* chains. Otherwise, comparisons that are based on means will be distorted (although comparisons based on over-dispersion parameters are unaffected by this correction).

S6.5 A probabilistic approach to quantify evidence of changes in expression patterns

A probabilistic approach is adopted, assessing changes in expression patterns (mean and over-dispersion) through a simple and intuitive scale of evidence. Our strategy is flexible and can be combined with a variety of decision rules. In particular, here we focus on highlighting genes whose absolute LFC in overall expression and biological over-dispersion between the populations exceeds minimum tolerance thresholds τ_0 and ω_0, respectively ($\tau_0, \omega_0 \geq 0$), set *a priori*. For a

given probability threshold $\alpha_{_D}$ ($0.5 < \alpha_{_D} < 1$), a gene i is identified to exhibit a change in biological over-dispersion between populations p and p' if

$$\pi^D_{ipp'}(\omega_0) \equiv \mathrm{P}(|\log(\delta_i^{(p)}/\delta_i^{(p')})| > \omega_0|\{\text{data}\}) > \alpha_{_D}, \quad i = 1, \ldots q_0. \tag{S22}$$

An empirical estimate of this tail posterior probability can be easily obtained using an MCMC sample from the posterior distribution of the $\delta_i^{(p)}$'s. In fact, this quantity can be estimated as

$$\hat{\pi}^D_{ipp'}(\omega_0) = \frac{1}{N}\sum_{m=1}^{N} \mathbf{I}\left(|\log\left(\delta_i^{(p)m}/\delta_i^{(p')m}\right)| > \omega_0\right), \tag{S23}$$

where $\delta_i^{(p)m}$ denotes the m-th posterior sample from $\delta_i^{(p)}$ and where $\mathbf{I}(A)$ is an indicator function equal to 1 if the event A is true and 0 otherwise. If $\omega_0 \to 0$, $\pi_i^D(\omega_0) \to 1$ becoming uninformative to detect changes in biological over-dispersion. As in Bochkina and Richardson (2007), in the limiting case where $\tau_0 = 0$, we define

$$\pi^D_{ipp'}(0) = 2\max\left\{\tilde{\pi}^D_{ipp'}, 1 - \tilde{\pi}^D_{ipp'}\right\} - 1 \;\text{ with }\; \tilde{\pi}^D_{ipp'} = \mathrm{P}(\log(\delta_i^{(p)}/\delta_i^{(p')}) > 0|\{\text{data}\}). \tag{S24}$$

In line with (S23), the latter can be estimated as

$$\hat{\tilde{\pi}}^D_{ipp'} = \frac{1}{N}\sum_{m=1}^{N} \mathbf{I}\left(\log\left(\delta_i^{(p)m}/\delta_i^{(p')m}\right) > 0\right). \tag{S25}$$

Analogous expression are used for the detection of genes whose overall expression rate changes between the populations. In such a case, we replace $\log(\delta_i^{(p)}/\delta_i^{(p')})$, ω_0 and α_D by $\log(\mu_i^{(p)}/\mu_i^{(p')})$, τ_0 and α_M, respectively.

References

Bochkina, N. and S. Richardson (2007). Tail posterior probability for inference in pairwise and multiclass gene expression data. *Biometrics 63*(4), 1117–1125.

Buettner, F., K. N. Natarajan, F. P. Casale, V. Proserpio, A. Scialdone, F. J. Theis, S. A. Teichmann, J. C. Marioni, and O. Stegle (2015). Computational analysis of cell-to-cell heterogeneity in single-cell RNA-sequencing data reveals hidden subpopulations of cells. *Nature Biotechnology 33*, 155–160.

Darzynkiewicz, Z., H. Crissman, F. Traganos, and J. Steinkamp (1982). Cell heterogeneity during the cell cycle. *Journal of Cellular Physiology 113*(3), 465–474.

Eddelbuettel, D., R. François, J. Allaire, J. Chambers, D. Bates, and K. Ushey (2011). Rcpp: Seamless R and C++ integration. *Journal of Statistical Software 40*(8), 1–18.

Finak, G., A. McDavid, M. Yajima, J. Deng, V. Gersuk, A. K. Shalek, C. K. Slichter, H. W. Miller, M. J. McElrath, M. Prlic, et al. (2015). Mast: a flexible statistical framework for assessing transcriptional changes and characterizing heterogeneity in single-cell rna sequencing data. *Genome biology 16*(1), 1–13.

Grün, D., L. Kester, and A. van Oudenaarden (2014). Validation of noise models for single-cell transcriptomics. *Nature Methods 11*(6), 637–640.

Islam, S., A. Zeisel, S. Joost, G. La Manno, P. Zajac, M. Kasper, P. Lönnerberg, and S. Linnarsson (2014). Quantitative single-cell RNA-seq with unique molecular identifiers. *Nature Methods 11*(2), 163–166.

Jiang, L., F. Schlesinger, C. A. Davis, Y. Zhang, R. Li, M. Salit, T. R. Gingeras, and B. Oliver (2011). Synthetic spike-in standards for RNA-seq experiments. *Genome Research 21*(9), 1543–1551.

Kharchenko, P. V., L. Silberstein, and D. T. Scadden (2014). Bayesian approach to single-cell differential expression analysis. *Nature Methods 11*(7), 740–742.

Love, M. I., W. Huber, and S. Anders (2014). Moderated estimation of fold change and dispersion for RNA-seq data with DESeq2. *Genome Biology 15*(12), 550.

McCarthy, D. J., Y. Chen, and G. K. Smyth (2012). Differential expression analysis of multifactor RNA-Seq experiments with respect to biological variation. *Nucleic acids research*, gks042.

R Core Team (2014). *R: A Language and Environment for Statistical Computing*. Vienna, Austria: R Foundation for Statistical Computing.

Roberts, G. O. and J. S. Rosenthal (2009). Examples of adaptive MCMC. *Journal of Computational and Graphical Statistics 18*(2), 349–367.

Robinson, M. D., D. J. McCarthy, and G. K. Smyth (2010). edgeR: a Bioconductor package for differential expression analysis of digital gene expression data. *Bioinformatics 26*(1), 139–140.

Vallejos, C. A., J. C. Marioni, and S. Richardson (2015). BASiCS: Bayesian analysis of single-cell sequencing data. *PLoS Computational Biology 11*(6), e1004333.
