# Supplementary material for: Beyond comparisons of means: understanding changes in gene expression at the single-cell level
Source: Genome Biol. 2016 Apr 15;17:70. doi: 10.1186/s13059-016-0930-3 (PMC4832562; doi:10.1186/s13059-016-0930-3)

Comparison of single-cells versus pool-and-split samples

Catalina A. Vallejos, Sylvia Richardson and John C. Marioni

2016-03-09

To demonstrate the efficacy of our method, we use the control experiment described in Grun et al (2014), where single mouse embryonic stem cells (mESCs) are compared against *pool-and-split* samples, consisting of pooled RNA from thousands of mESCs split into single-cell equivalent volumes. Such a controlled setting provides a situation where substantial changes in overall expression are not expected as, on average, the overall expression of single cells should match the levels measured on pool-and-split samples. Additionally, the design of pool-and-split samples removes biological variation, leading to a homogenous set of samples. Hence, pool-and-split samples are expected to show a genuine reduction in biological cell-to-cell heterogeneity when compared to single-cells.

In this document, we provide the R code used to perform the analysis described in

> Vallejos C.A., Richardson S. and Marioni J.C. (2016)
> Beyond comparisons of means: understanding changes in gene expression at the single-cell level

To start the analysis, the following data must be dowloaded and stored in `data.path` directory.

- The expression counts: 'GSE54695_data_transcript_counts.txt' (source: http://www.ncbi.nlm.nih.gov/geo/query/acc.cgi?acc=GSE54695).
- The concentrations in the ERCC mix: 'cms_095046.txt'. The column names of this file have been modified to be readable from R (source: https://tools.thermofisher.com/content/sfs/manuals/cms_095046.txt)

Additionally, the following R libraries must be loaded before performing the analysis

```
library(BASiCS) # To run the analysis
packageVersion("BASiCS")
```

```
## [1] '0.5.3'
```

```
library(data.table) # For fast loading and processing of large datasets
packageVersion("data.table")
```

```
## [1] '1.9.6'
```

Preparing the data

Loading the data

```
data = fread(file.path(data.path,"GSE54695_data_transcript_counts.txt"))
Gene.Ids = data$GENENAME
Cell.Ids = names(data)[-1]
```

```r
RawCounts = cbind(subset(data, select = Cell.Ids[grep("SC_2i", Cell.Ids)]),
                  subset(data, select = Cell.Ids[grep("RNA_2i", Cell.Ids)]))

Cell.Colour =  c( rep("lightpink3",length(grep("SC_2i", Cell.Ids))),
                  rep("darkolivegreen3",length(grep("RNA_2i", Cell.Ids))) )
Batch = c(rep(1, 40), rep(2, 40), rep(1, 40), rep(2, 40))
```

The input data contains 12535 genes and 160 cells.

Transforming the data into UMI counts

```r
# Function provided by Jong Kyoung Kim (EMBL-EBI)
UMICount <- function(MoleculeCount, UMILength)
{
  # MoleculeCount is the normalized count
  M = 4^UMILength
  UMICount = M*(1-exp(-MoleculeCount/M))
  return(UMICount)
}

CountsUMI = round(UMICount(RawCounts, 4))
```

Quality control: filtering cells

Before running the analysis it is important to filter out samples with poor quality.

```r
Pou5f1.per.cell <- as.numeric(CountsUMI[which(Gene.Ids == "Pou5f1"),])
counts.per.cell <- colSums(CountsUMI)
genes.per.cell <- apply(CountsUMI, 2, function(x) sum( x>0 ))
ercc.per.cell <- colSums(CountsUMI[grep("ERCC", Gene.Ids),])
par(mfrow = c(3,1))
par(cex.lab = 1.5, cex.axis = 1.5, cex.main = 2)
plot(Pou5f1.per.cell[order(Pou5f1.per.cell)],
     col = Cell.Colour[order(Pou5f1.per.cell)],
     bty = "n", xlab = "Cells", ylab = "Pou5f1 counts",
     main = "Pou5f1 counts per cell", cex = 1.5, pch = 16)
abline(h = 10, lty = 2)
abline(v = 10, lty = 2)
legend('topleft', c("SC_2i", "RNA_2i"), col = unique(Cell.Colour)[2:1], pch = 16, cex = 2)
plot(ercc.per.cell[order(Pou5f1.per.cell)], pch = 16,
     col = Cell.Colour[order(Pou5f1.per.cell)],
     bty = "n", xlab = "Cells", ylab = "ERCC counts",
     main = "ERCC counts per cell", cex = 1.5)
abline(h = 100, lty = 2)
abline(v = 10, lty = 2)
plot(counts.per.cell[order(Pou5f1.per.cell)], pch = 16,
     col = Cell.Colour[order(Pou5f1.per.cell)], log = "y",
     bty = "n", xlab = "Cells", ylab = "Total counts",
     main = "Total counts per cell", cex = 1.5)
abline(h = 1e4, lty = 2)
abline(v = 10, lty = 2)
```

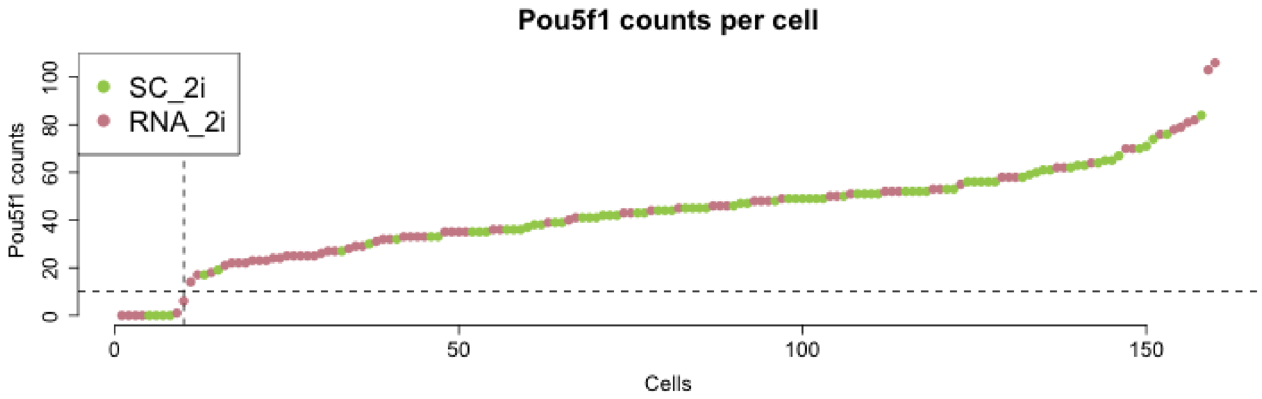

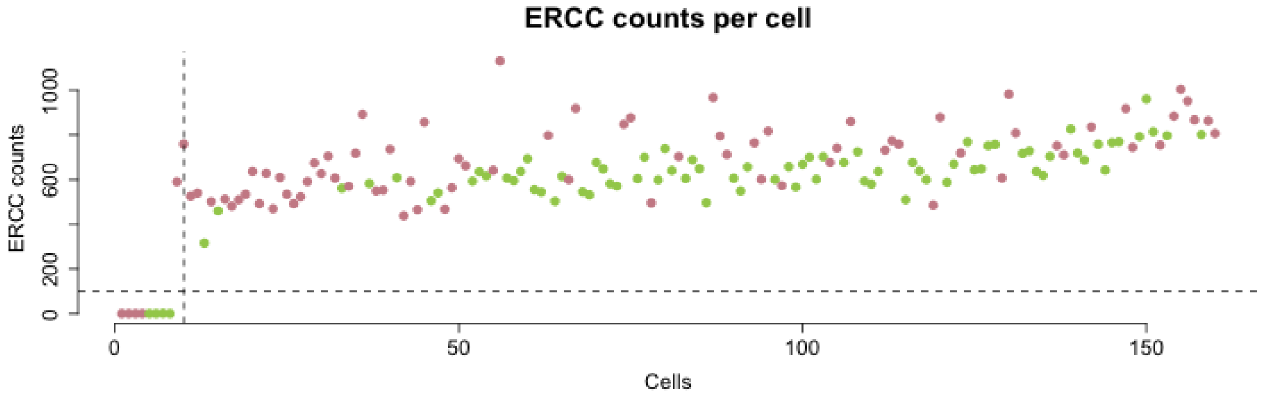

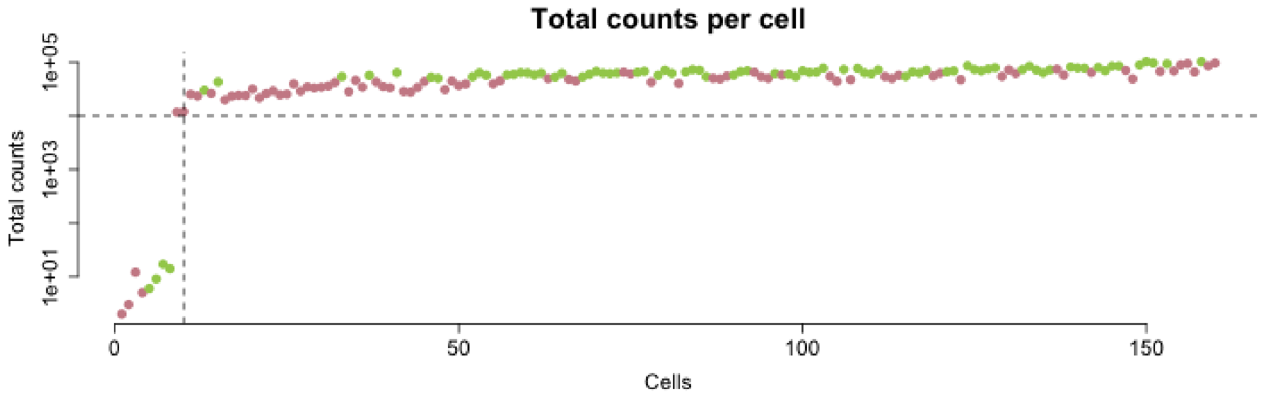


```
CountsUMI_1 <- CountsUMI[,Pou5f1.per.cell >= 10, with = FALSE]
Cell.Colour_1 <- Cell.Colour[Pou5f1.per.cell >= 10]
Batch_1 <- Batch[Pou5f1.per.cell >= 10]
```

Following Grun et al (2014), we discarded those cells where less than 10 transcripts of *Pou5f1* were detected (because poor sequencing efficiency or due to potential undergoing differentiation). We also notice that most of these discarded cells also exhibit particularly low total counts (especially for the ERCCs). After this filter, **current processed data contains 12535 genes and 150 cells (74 and 76 single cells and pool-and-split samples, respectively).**

Filtering of transcripts (removing the low signal genes)

To remove lowly expressed genes that have less biological relevant, our analysis only includes transcripts with at least 50 counts in total across all cells.

```
CountsUMI_2 = CountsUMI_1[rowSums(CountsUMI_1) >= 50, ]
Gene.Ids_2 = Gene.Ids[rowSums(CountsUMI_1) >= 50]
```

After this filter, **current processed data contains 9378 (9343 and 35 intrinsic and spike-in genes, respectively).**

BASiCS analysis

The input dataset

To perform comparisons between two groups of cells, the current implementation of `BASiCS` requires the creation of two separate `BASiCS_Data` objects to contain separate information regarding each group. Two `BASiCS_Data` must be combined into one `BASiCS_D_Data` to perform comparisons between the corresponding groups of cells (using the function `CombineBASiCS_Data`). Similarly, two `BASiCS_Chain` object can be combined into a `BASiCS_D_Chain` object using the function `CombineBASiCS_Chain`.

Spike-in genes information

In addition to the matrix of counts, the creation of `BASiCS_Data` and `BASiCS_D_Data` objects requires information regarding the number of spike-in molecules that are theoretically added to each cell. These quantities can be calculated based on the concentrations of ERCC molecules in the mix and the dilution factors used for library preparation. These are calculated below.

```
SpikeInfo <- fread(file.path(data.path,"cms_095046.txt"))
SpikeInfo <- SpikeInfo[SpikeInfo$ERCC_ID %in% Gene.Ids_2[grep("ERCC", Gene.Ids_2)],]
SpikeInfo$MoleculesPerCell <- SpikeInfo$concentration_in_Mix_1 * (1e-18) *
                                             (6.022e23) * (1/2500000)

# To confirm with the 3.3% capture indicated by Grun et al
SpikeOut <- data.table("ERCC_ID" = Gene.Ids_2[grep("ERCC", Gene.Ids_2)],
                       "ERCC_MeanCount" = rowMeans(CountsUMI_2[grep("ERCC", Gene.Ids_2),]))
SpikeOut = merge(SpikeInfo, SpikeOut, by = "ERCC_ID")
SpikeInfoFilter = subset(SpikeInfo, select = c(ERCC_ID, MoleculesPerCell))

# Creating indicator of technical genes
Tech = grepl("ERCC", Gene.Ids_2)
```

Overall, a 3.55 % of the added ERCC molecules where captured during the experiment.

Separating expression counts for each condition

A final step before creating the required `BASiCS_Data` objects is to separate the matrix of counts according to the grouping of cells.

```r
CountsUMI_SC = as.matrix(CountsUMI_2)[, grep("SC_2i",colnames(CountsUMI_2))]
CountsUMI_RNA = as.matrix(CountsUMI_2)[, grep("RNA_2i",colnames(CountsUMI_2))]
rownames(CountsUMI_SC) <- Gene.Ids_2
rownames(CountsUMI_RNA) <- Gene.Ids_2
```

Creating the input object

```r
Data.SC = newBASiCS_Data(Counts = CountsUMI_SC,
                         Tech = Tech,
                         SpikeInfo = SpikeInfoFilter,
                         BatchInfo = Batch_1[grep("SC_2i",colnames(CountsUMI_2))])
```

```
## An object of class BASiCS_Data
##  Dataset contains 9378 genes (9343 biological and 35 technical) and 74 cells.
##  Elements (slots): Counts, Tech, SpikeInput, GeneNames and BatchInfo.
##  The data contains 2 batches.
##
## NOTICE: BASiCS requires a pre-filtered dataset
##     - You must remove poor quality cells before creating the BASiCS data object
##     - We recommend to pre-filter very lowly expressed transcripts before creating the object.
##       Inclusion criteria may vary for each data. For example, remove transcripts
##           - with very low total counts across of all of the samples
##           - that are only expressed in a few cells
##             (by default genes expressed in only 1 cell are not accepted)
##           - with very low total counts across the samples where the transcript is expressed
##
##  BASiCS_Filter can be used for this purpose
```

```r
Data.RNA = newBASiCS_Data(Counts = CountsUMI_RNA,
                         Tech = Tech,
                         SpikeInfo = SpikeInfoFilter,
                         BatchInfo = Batch_1[grep("RNA_2i",colnames(CountsUMI_2))])
```

```
## An object of class BASiCS_Data
##  Dataset contains 9378 genes (9343 biological and 35 technical) and 76 cells.
##  Elements (slots): Counts, Tech, SpikeInput, GeneNames and BatchInfo.
##  The data contains 2 batches.
##
## NOTICE: BASiCS requires a pre-filtered dataset
##     - You must remove poor quality cells before creating the BASiCS data object
##     - We recommend to pre-filter very lowly expressed transcripts before creating the object.
##       Inclusion criteria may vary for each data. For example, remove transcripts
##           - with very low total counts across of all of the samples
##           - that are only expressed in a few cells
##             (by default genes expressed in only 1 cell are not accepted)
##           - with very low total counts across the samples where the transcript is expressed
##
##  BASiCS_Filter can be used for this purpose
```

```
Data = CombineBASiCS_Data(Data.SC, Data.RNA)
```

```
##
## NOTICE: BASiCS requires a pre-filtered dataset
##     - You must remove poor quality cells before creating the BASiCS data object
##     - We recommend to pre-filter very lowly expressed transcripts before creating the object.
##       Inclusion criteria may vary for each data. For example, remove transcripts
##           - with very low total counts across of all cells
##           - that are only expressed in few cells
##             (by default genes expressed in only 1 cell are not accepted)
##           - with very low total counts across the cells where the transcript is expressed
##
##  BASiCS_Filter can be used for this purpose
##
## An object of class BASiCS_D_Data
##  Dataset contains 9378 genes (9343 biological and 35 technical) and 150 cells.
##         - 74 cells in the test sample, with2batch(es)
##         - 76 cells in the reference sample, with2batch(es)
##  Elements (slots): CountsTest, CountsRef, Tech, SpikeInputTest, SpikeInputRef, BatchInfoTest, BatchIn
```

Fitting the BASiCS model

To run the MCMC algorithm, we use the function `BASiCS_MCMC`. As a default, we used `a2.mu = 0.5` and `a2.delta = 0.5`.

```
N = 40000; Thin = 20; Burn = 20000
RunName.SC = paste0("Grun_Split2i_SC_Batch",N)
RunName.RNA = paste0("Grun_Split2i_RNA_Batch",N)

MCMC_OutputTest <- BASiCS_MCMC(Data.SC, N = N, Thin = Thin, Burn = Burn,
                              PrintProgress = TRUE, StoreChains = FALSE,
                              StoreDir = chains.path, RunName = RunName.SC)

MCMC_OutputRef <- BASiCS_MCMC(Data.RNA, N = N, Thin = Thin, Burn = Burn,
                              PrintProgress = TRUE, StoreChains = FALSE,
                              StoreDir = chains.path, RunName = RunName.RNA)
```

Loading pre-computed chains

This report shows the results of pre-computed chains as defined by the previous chunk of code.

```
ChainMuTest = as.matrix(fread(file.path(chains.path, "chain_mu_Grun_Split2i_SC_Batch40000.txt")))
ChainMuRef = as.matrix(fread(file.path(chains.path, "chain_mu_Grun_Split2i_RNA_Batch40000.txt")))
ChainDeltaTest = as.matrix(fread(file.path(chains.path, "chain_delta_Grun_Split2i_SC_Batch40000.txt")))
ChainDeltaRef = as.matrix(fread(file.path(chains.path, "chain_delta_Grun_Split2i_RNA_Batch40000.txt")))
ChainPhiTest = as.matrix(fread(file.path(chains.path, "chain_phi_Grun_Split2i_SC_Batch40000.txt")))
ChainPhiRef = as.matrix(fread(file.path(chains.path, "chain_phi_Grun_Split2i_RNA_Batch40000.txt")))
ChainSTest = as.matrix(fread(file.path(chains.path, "chain_s_Grun_Split2i_SC_Batch40000.txt")))
ChainSRef = as.matrix(fread(file.path(chains.path, "chain_s_Grun_Split2i_RNA_Batch40000.txt")))
ChainNuTest = as.matrix(fread(file.path(chains.path, "chain_nu_Grun_Split2i_SC_Batch40000.txt")))
ChainNuRef = as.matrix(fread(file.path(chains.path, "chain_nu_Grun_Split2i_RNA_Batch40000.txt")))
```

```
ChainThetaTest = as.matrix(fread(file.path(chains.path, "chain_theta_Grun_Split2i_SC_Batch40000.txt")))
ChainThetaRef = as.matrix(fread(file.path(chains.path, "chain_theta_Grun_Split2i_RNA_Batch40000.txt")))

MCMC_OutputTest = newBASiCS_Chain(mu = ChainMuTest,
                                  delta = ChainDeltaTest,
                                  phi = ChainPhiTest,
                                  s = ChainSTest,
                                  nu = ChainNuTest,
                                  theta = ChainThetaTest)
```

```
## An object of class BASiCS_Chain
##  1000 MCMC samples.
##  Dataset contains 9343 biological genes and 74 cells (2 batches).
##  Elements (slots): mu, delta, phi, s, nu and theta.
```

```
MCMC_OutputRef = newBASiCS_Chain(mu = ChainMuRef,
                                  delta = ChainDeltaRef,
                                  phi = ChainPhiRef,
                                  s = ChainSRef,
                                  nu = ChainNuRef,
                                  theta = ChainThetaRef)
```

```
## An object of class BASiCS_Chain
##  1000 MCMC samples.
##  Dataset contains 9343 biological genes and 76 cells (2 batches).
##  Elements (slots): mu, delta, phi, s, nu and theta.
```

```
MCMC_Output <- CombineBASiCS_Chain(MCMC_OutputTest, MCMC_OutputRef)
```

```
## An object of class BASiCS_D_Chain
##  1000 MCMC samples.
##  Dataset contains 9343 biological genes and 150 cells (in total across both samples).
##  Offset = 1.
##  Elements (slots): muTest, muRef, deltaTest, omegaRef, phi, s, nu, thetaTest, thetaRef and offset.
```

Convergence diagnostics

To assess convergence of the chain, the convergence diagnostics provided by the package `coda` can be used. Additionally, a visual inspection can be performed using traceplot. As an illustration, here we display traceplots for medians across groups of parameters (e.g. the median across all $\mu[i1]$'s). It can be seen that the mixing of the chains related to ϕ_j's and ν_j's mixes less well. Nonetheless, their values are still concentrated on a very small range.

```
nTest = ncol(Data@CountsTest)
nRef = ncol(Data@CountsRef)
n = nTest + nRef
par(mgp = c(5,1,0)); par(mar = c(7,9,4,0.5)); par(mfrow = c(6,2))
par(cex.lab = 2, cex.axis = 1.5)
plot(apply(MCMC_Output@muTest, 1, median), type = "l",
     ylab = expression(paste("Median of ",mu[i1])), xlab = "Iteration")
plot(apply(MCMC_Output@muRef, 1, median), type = "l",
```

```
     ylab = expression(paste("Median of ",mu[i2])), xlab = "Iteration")
plot(apply(MCMC_Output@deltaTest, 1, median), type = "l",
     ylab = expression(paste("Median of ",delta[i1])), xlab = "Iteration")
plot(apply(MCMC_Output@deltaRef, 1, median), type = "l",
     ylab = expression(paste("Median of ",delta[i2])), xlab = "Iteration")
plot(apply(MCMC_Output@phi[,1:nTest], 1, median), type = "l",
     ylab = expression(paste("Median of ",phi[j[1]])), xlab = "Iteration")
plot(apply(MCMC_Output@phi[,(nTest+1):n], 1, median), type = "l",
     ylab = expression(paste("Median of ",phi[j[2]])), xlab = "Iteration")
plot(apply(MCMC_Output@s[,1:nTest], 1, median), type = "l",
     ylab = expression(paste("Median of ",s[j[1]])), xlab = "Iteration")
plot(apply(MCMC_Output@s[,(nTest+1):n], 1, median), type = "l",
     ylab = expression(paste("Median of ",s[j[2]])), xlab = "Iteration")
plot(apply(MCMC_Output@nu[,1:nTest], 1, median), type = "l",
     ylab = expression(paste("Median of ",nu[j[1]])), xlab = "Iteration")
plot(apply(MCMC_Output@nu[,(nTest+1):n], 1, median), type = "l",
     ylab = expression(paste("Median of ",nu[j[2]])), xlab = "Iteration")
plot(MCMC_Output@thetaTest[,1], type = "l",
     ylab = expression(theta[1]), xlab = "Iteration")
plot(MCMC_Output@thetaRef[,1], type = "l",
     ylab = expression(theta[2]), xlab = "Iteration")
```

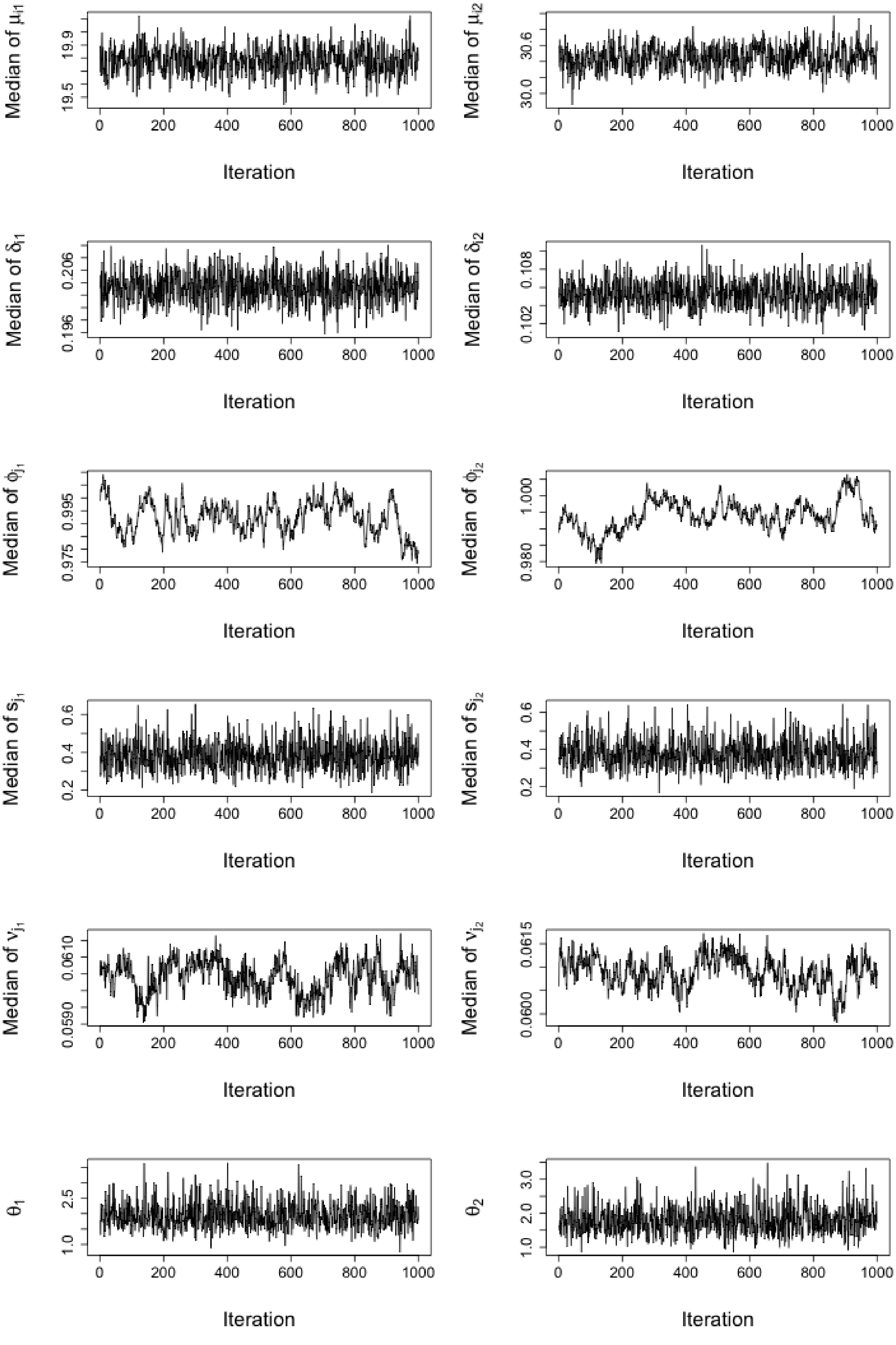
Median of μ_{j1}
19.5
19.9
Median of μ_{j2}
30.0
30.6
Median of δ_{j1}
0.196
0.200
0.206
Median of δ_{j2}
0.102
0.105
0.108
Median of ϕ_{j1}
0.975
0.985
0.995
Median of ϕ_{j2}
0.980
0.990
1.000
Median of s_{j1}
0.2
0.4
0.6
Median of s_{j2}
0.2
0.4
0.6
Median of v_{j1}
0.0590
0.0600
0.0610
Median of v_{j2}
0.0600
0.0615
θ_1
1.0
2.5
θ_2
1.0
2.0
3.0
0
200
400
600
800
1000
Iteration

We also provide additional information to assess the convergence of model parameters that are gene-specific, which are the key parameters in our model and define the results of the comparisons between cell types (randomly selected genes only)

Traceplots and autocorrelation plots for overall expression parameters (test group)

```
par(mgp = c(5,1,0)); par(mar = c(7,9,4,0.5)); par(mfrow = c(4,2))
par(cex.lab = 2, cex.axis = 1.5)
genesel = sample(1:ncol(MCMC_Output@deltaTest), 1)
plot(MCMC_Output@muTest[,genesel], type = "l", main = Data@GeneNames[genesel],
     ylab = expression(paste("Median of ",mu[i1])), xlab = "Iteration")
acf(MCMC_Output@muTest[,genesel], main = Data@GeneNames[genesel])
genesel = sample(1:ncol(MCMC_Output@deltaTest), 1)
plot(MCMC_Output@muTest[,genesel], type = "l", main = Data@GeneNames[genesel],
     ylab = expression(paste("Median of ",mu[i1])), xlab = "Iteration")
acf(MCMC_Output@muTest[,genesel], main = Data@GeneNames[genesel])
genesel = sample(1:ncol(MCMC_Output@deltaTest), 1)
plot(MCMC_Output@muTest[,genesel], type = "l", main = Data@GeneNames[genesel],
     ylab = expression(paste("Median of ",mu[i1])), xlab = "Iteration")
acf(MCMC_Output@muTest[,genesel], main = Data@GeneNames[genesel])
genesel = sample(1:ncol(MCMC_Output@deltaTest), 1)
plot(MCMC_Output@muTest[,genesel], type = "l", main = Data@GeneNames[genesel],
     ylab = expression(paste("Median of ",mu[i1])), xlab = "Iteration")
acf(MCMC_Output@muTest[,genesel], main = Data@GeneNames[genesel])
```

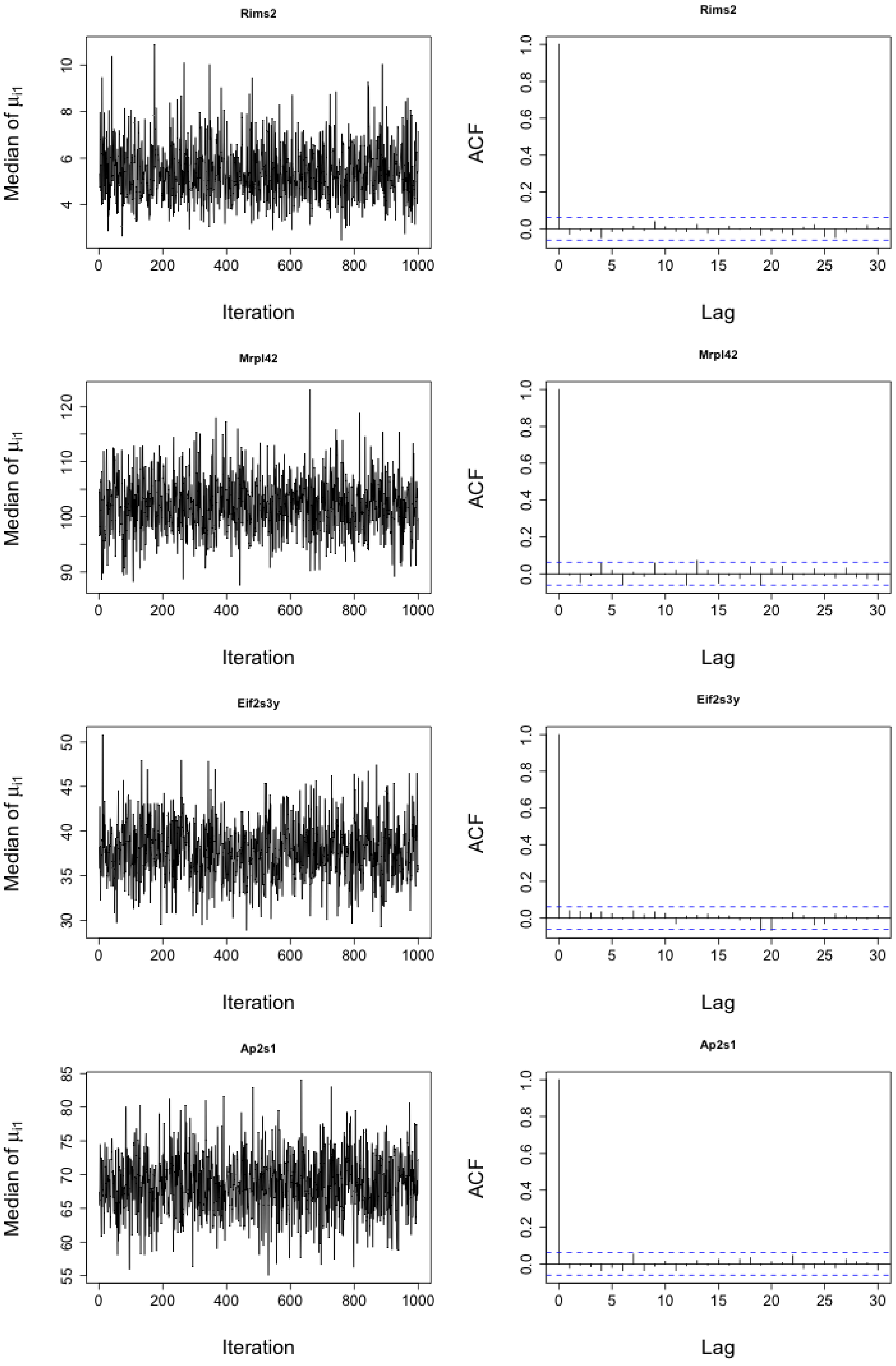
Rims2
Median of μ_{i1}
Iteration
Rims2
ACF
Lag
Mrpl42
Median of μ_{i1}
Iteration
Mrpl42
ACF
Lag
Eif2s3y
Median of μ_{i1}
Iteration
Eif2s3y
ACF
Lag
Ap2s1
Median of μ_{i1}
Iteration
Ap2s1
ACF
Lag

Traceplots and autocorrelation plots for overall expression parameters (ref group)

```
par(mgp = c(5,1,0)); par(mar = c(7,9,4,0.5)); par(mfrow = c(4,2))
par(cex.lab = 2, cex.axis = 1.5)
genesel = sample(1:ncol(MCMC_Output@deltaRef), 1)
plot(MCMC_Output@muRef[,genesel], type = "l", main = Data@GeneNames[genesel],
     ylab = expression(paste("Median of ",mu[i2])), xlab = "Iteration")
acf(MCMC_Output@muRef[,genesel], main = Data@GeneNames[genesel])
genesel = sample(1:ncol(MCMC_Output@deltaRef), 1)
plot(MCMC_Output@muRef[,genesel], type = "l", main = Data@GeneNames[genesel],
     ylab = expression(paste("Median of ",mu[i2])), xlab = "Iteration")
acf(MCMC_Output@muRef[,genesel], main = Data@GeneNames[genesel])
genesel = sample(1:ncol(MCMC_Output@deltaRef), 1)
plot(MCMC_Output@muRef[,genesel], type = "l", main = Data@GeneNames[genesel],
     ylab = expression(paste("Median of ",mu[i2])), xlab = "Iteration")
acf(MCMC_Output@muRef[,genesel], main = Data@GeneNames[genesel])
genesel = sample(1:ncol(MCMC_Output@deltaRef), 1)
plot(MCMC_Output@muRef[,genesel], type = "l", main = Data@GeneNames[genesel],
     ylab = expression(paste("Median of ",mu[i2])), xlab = "Iteration")
acf(MCMC_Output@muRef[,genesel], main = Data@GeneNames[genesel])
```

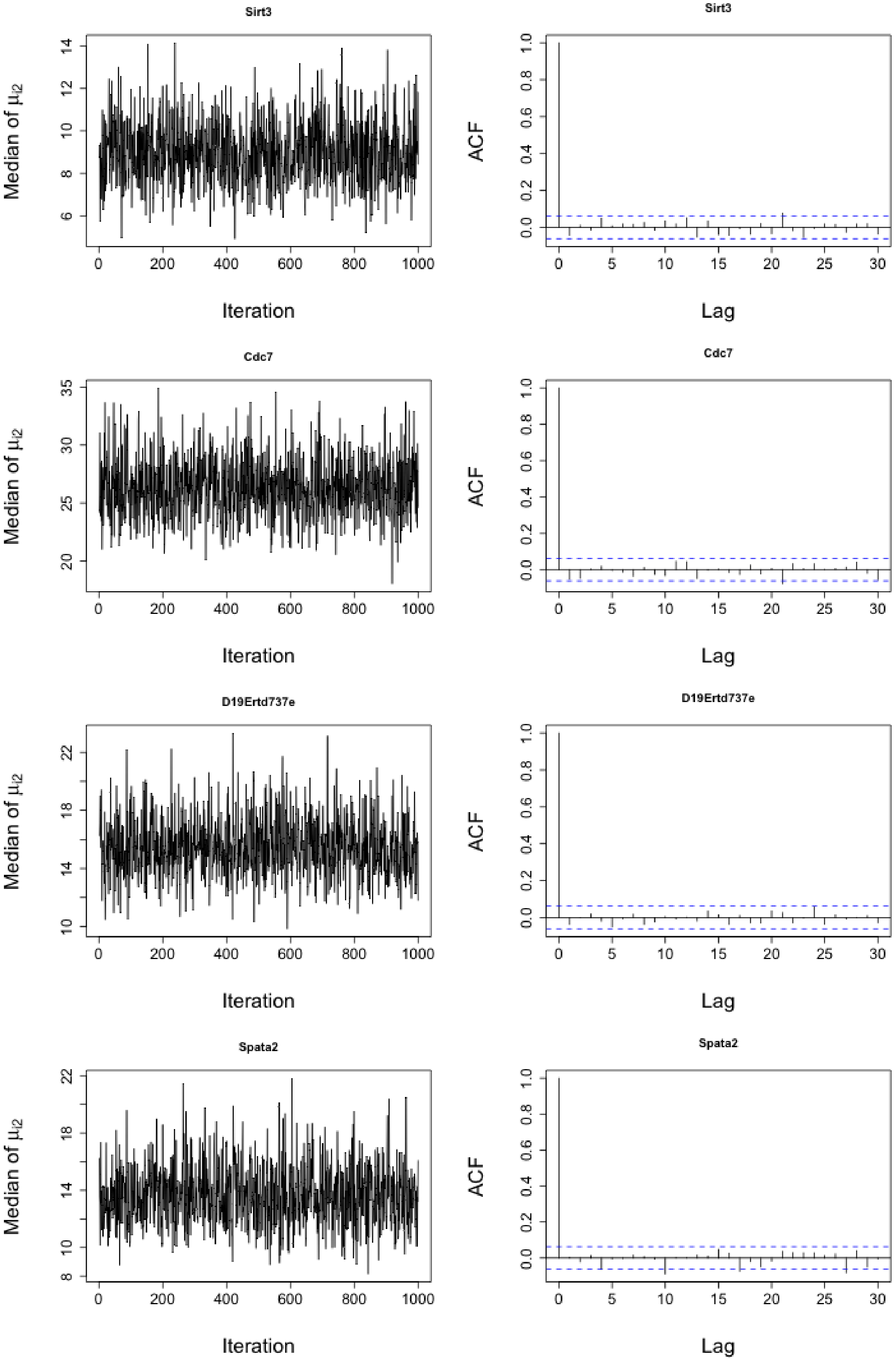
Sirt3
Median of μ_{i2}
Iteration
ACF
Lag
Cdc7
Median of μ_{i2}
Iteration
ACF
Lag
D19Ertd737e
Median of μ_{i2}
Iteration
ACF
Lag
Spata2
Median of μ_{i2}
Iteration
ACF
Lag

Traceplots and autocorrelation plots for dispersion parameters (test group)

```
par(mgp = c(5,1,0)); par(mar = c(7,9,4,0.5)); par(mfrow = c(4,2))
par(cex.lab = 2, cex.axis = 1.5)
genesel = sample(1:ncol(MCMC_Output@deltaTest), 1)
plot(MCMC_Output@deltaTest[,genesel], type = "l", main = Data@GeneNames[genesel],
     ylab = expression(paste("Median of ",delta[i1])), xlab = "Iteration")
acf(MCMC_Output@deltaTest[,genesel], main = Data@GeneNames[genesel])
genesel = sample(1:ncol(MCMC_Output@deltaTest), 1)
plot(MCMC_Output@deltaTest[,genesel], type = "l", main = Data@GeneNames[genesel],
     ylab = expression(paste("Median of ",delta[i1])), xlab = "Iteration")
acf(MCMC_Output@deltaTest[,genesel], main = Data@GeneNames[genesel])
genesel = sample(1:ncol(MCMC_Output@deltaTest), 1)
plot(MCMC_Output@deltaTest[,genesel], type = "l", main = Data@GeneNames[genesel],
     ylab = expression(paste("Median of ",delta[i1])), xlab = "Iteration")
acf(MCMC_Output@deltaTest[,genesel], main = Data@GeneNames[genesel])
genesel = sample(1:ncol(MCMC_Output@deltaTest), 1)
plot(MCMC_Output@deltaTest[,genesel], type = "l", main = Data@GeneNames[genesel],
     ylab = expression(paste("Median of ",delta[i1])), xlab = "Iteration")
acf(MCMC_Output@deltaTest[,genesel], main = Data@GeneNames[genesel])
```

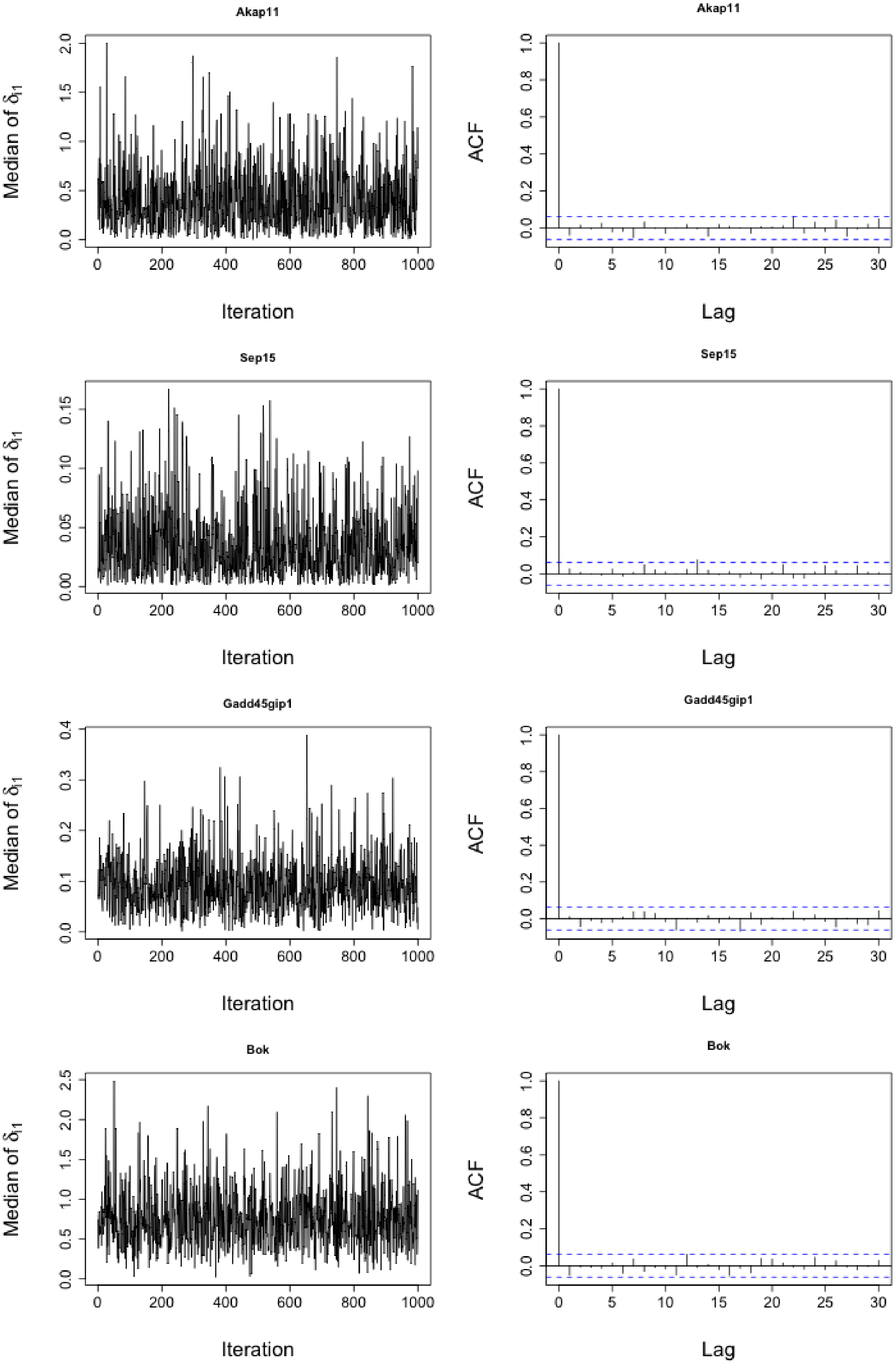
Akap11
Median of δ11
Iteration
ACF
Lag
Sep15
Median of δ11
Iteration
ACF
Lag
Gadd45gip1
Median of δ11
Iteration
ACF
Lag
Bok
Median of δ11
Iteration
ACF
Lag

Traceplots and autocorrelation plots for dispersion parameters (ref group)

```
par(mgp = c(5,1,0)); par(mar = c(7,9,4,0.5)); par(mfrow = c(4,2))
par(cex.lab = 2, cex.axis = 1.5)
genesel = sample(1:ncol(MCMC_Output@deltaRef), 1)
plot(MCMC_Output@deltaRef[,genesel], type = "l", main = Data@GeneNames[genesel],
     ylab = expression(paste("Median of ",delta[i2])), xlab = "Iteration")
acf(MCMC_Output@deltaRef[,genesel], main = Data@GeneNames[genesel])
genesel = sample(1:ncol(MCMC_Output@deltaRef), 1)
plot(MCMC_Output@deltaRef[,genesel], type = "l", main = Data@GeneNames[genesel],
     ylab = expression(paste("Median of ",delta[i2])), xlab = "Iteration")
acf(MCMC_Output@deltaRef[,genesel], main = Data@GeneNames[genesel])
genesel = sample(1:ncol(MCMC_Output@deltaRef), 1)
plot(MCMC_Output@deltaRef[,genesel], type = "l", main = Data@GeneNames[genesel],
     ylab = expression(paste("Median of ",delta[i2])), xlab = "Iteration")
acf(MCMC_Output@deltaRef[,genesel], main = Data@GeneNames[genesel])
genesel = sample(1:ncol(MCMC_Output@deltaRef), 1)
plot(MCMC_Output@deltaRef[,genesel], type = "l", main = Data@GeneNames[genesel],
     ylab = expression(paste("Median of ",delta[i2])), xlab = "Iteration")
acf(MCMC_Output@deltaRef[,genesel], main = Data@GeneNames[genesel])
```

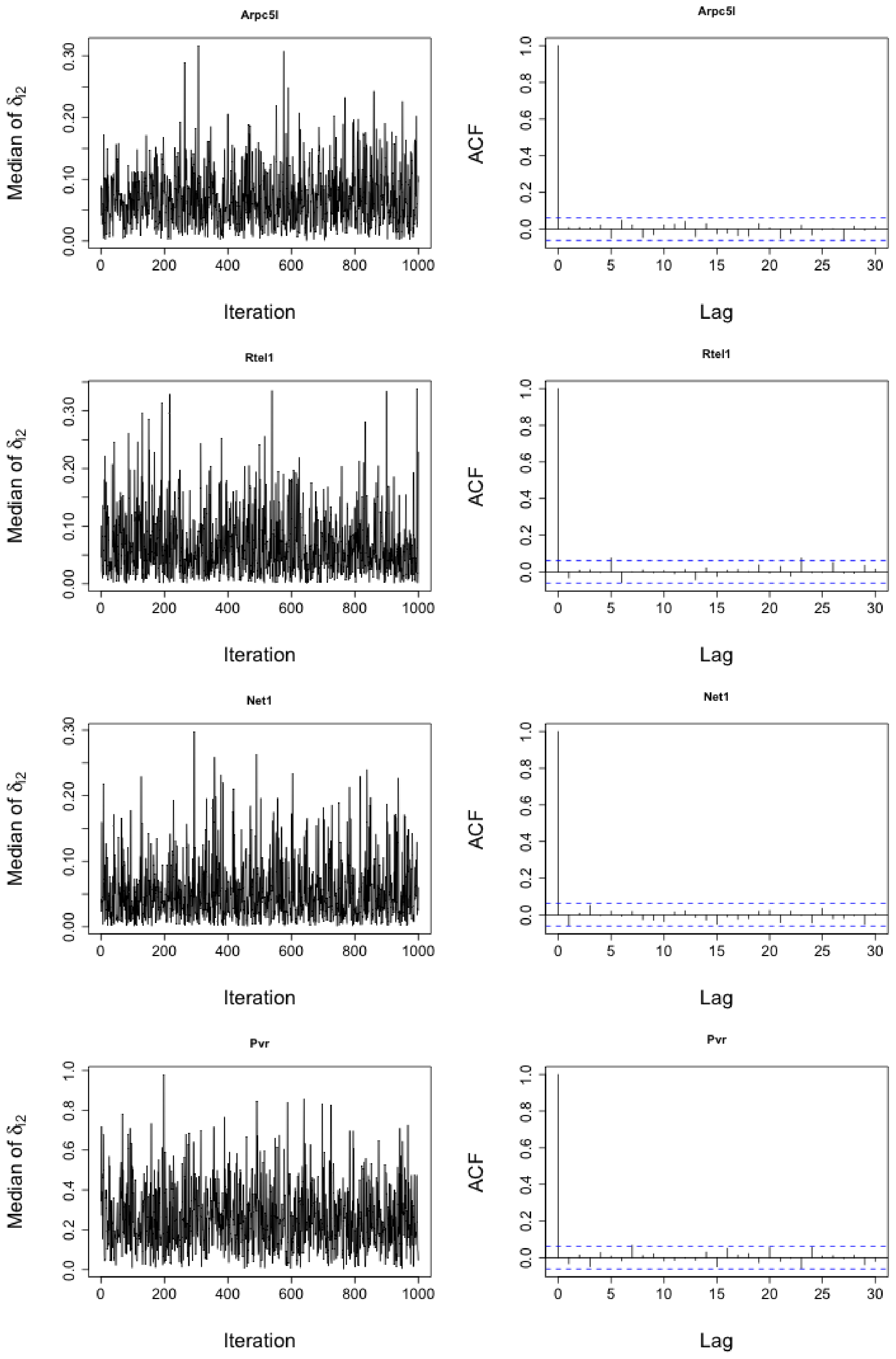
Arpc5l
Median of δi2
Iteration
Arpc5l
ACF
Lag
Rtel1
Median of δi2
Iteration
Rtel1
ACF
Lag
Net1
Median of δi2
Iteration
Net1
ACF
Lag
Pvr
Median of δi2
Iteration
Pvr
ACF
Lag

Additionally, we run the Geweke convergence diagnostic (see library `coda` for gene-specific parameters). As it can be seen bellow, most of the associated Z scores are small, with just a few cases lying outside the (-2,2) interval (not surprisingly due to the large number of parameters). For the genes with extreme Z score values, we also provide traceplots to illustrate that the evidence againts convergence is very weak (if any).

```
library(coda)
MuTestMCMC = mcmc(MCMC_Output@muTest)
MuRefMCMC = mcmc(MCMC_Output@muRef)
DeltaTestMCMC = mcmc(MCMC_Output@deltaTest)
DeltaRefMCMC = mcmc(MCMC_Output@deltaRef)

MuTestMCMC.geweke = geweke.diag(MuTestMCMC)
MuRefMCMC.geweke = geweke.diag(MuRefMCMC)
DeltaTestMCMC.geweke = geweke.diag(DeltaTestMCMC)
DeltaRefMCMC.geweke = geweke.diag(DeltaRefMCMC)

summary(cbind(MuTestMCMC.geweke$z, MuRefMCMC.geweke$z,
              DeltaTestMCMC.geweke$z, DeltaRefMCMC.geweke$z))
```

```
##        V1                  V2                 V3
##  Min.   :-4.74271   Min.   :-5.3355   Min.   :-4.10456
##  1st Qu.:-0.66584   1st Qu.:-0.8545   1st Qu.:-0.72011
##  Median : 0.02752   Median :-0.1550   Median :-0.02314
##  Mean   : 0.01053   Mean   :-0.1622   Mean   :-0.05360
##  3rd Qu.: 0.70734   3rd Qu.: 0.5396   3rd Qu.: 0.63920
##  Max.   : 6.01439   Max.   : 4.3149   Max.   : 4.85610
##        V4
##  Min.   :-5.15131
##  1st Qu.:-0.71665
##  Median :-0.01561
##  Mean   :-0.05246
##  3rd Qu.: 0.64568
##  Max.   : 4.36629
```

```
par(mgp = c(5,1,0)); par(mar = c(7,9,4,0.5)); par(mfrow = c(2,2))
par(cex.lab = 2, cex.axis = 1.5)
genesel = which(abs(MuTestMCMC.geweke$z) ==  max(abs(MuTestMCMC.geweke$z)) )
plot(MCMC_Output@muTest[,genesel], type = "l", main = Data@GeneNames[genesel],
     ylab = expression(paste("Median of ",mu[i1])), xlab = "Iteration")
genesel = which(abs(MuRefMCMC.geweke$z) ==  max(abs(MuRefMCMC.geweke$z)) )
plot(MCMC_Output@muRef[,genesel], type = "l", main = Data@GeneNames[genesel],
     ylab = expression(paste("Median of ",mu[i2])), xlab = "Iteration")
genesel = which(abs(DeltaTestMCMC.geweke$z) ==  max(abs(DeltaTestMCMC.geweke$z)) )
plot(MCMC_Output@deltaTest[,genesel], type = "l", main = Data@GeneNames[genesel],
     ylab = expression(paste("Median of ",delta[i1])), xlab = "Iteration")
genesel = which(abs(DeltaRefMCMC.geweke$z) ==  max(abs(DeltaRefMCMC.geweke$z)) )
plot(MCMC_Output@deltaRef[,genesel], type = "l", main = Data@GeneNames[genesel],
     ylab = expression(paste("Median of ",delta[i2])), xlab = "Iteration")
```

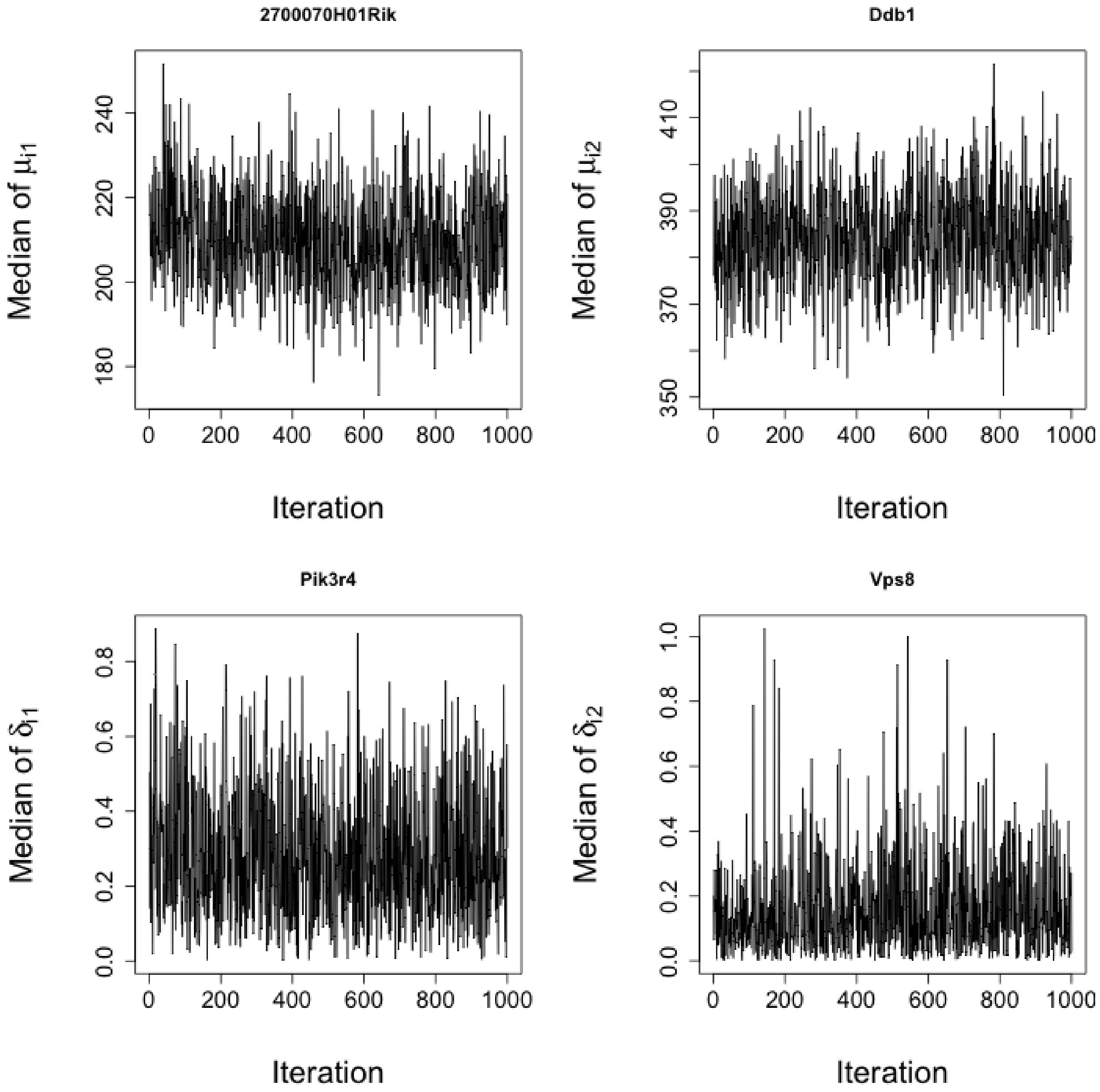

Finally, we also run additional MCMC chains with different starting values. All chains led to virtually the same results. Hence, we conclude we have strong evidence to support that the chain has reached its stationary distribution (not shown).

Comparison of noise parameters per group and batch

This figure suggests batch 1 is affected by stronger noise.

```
boxplot(cbind(ChainThetaTest, ChainThetaRef),
        col = rep(unique(Cell.Colour_1), 2, each = 2),
        frame = F, ylab = "Noise parameter")
legend('top', c("Single-cells", "Pool-and-split"), col = unique(Cell.Colour_1), pch = 15)
```

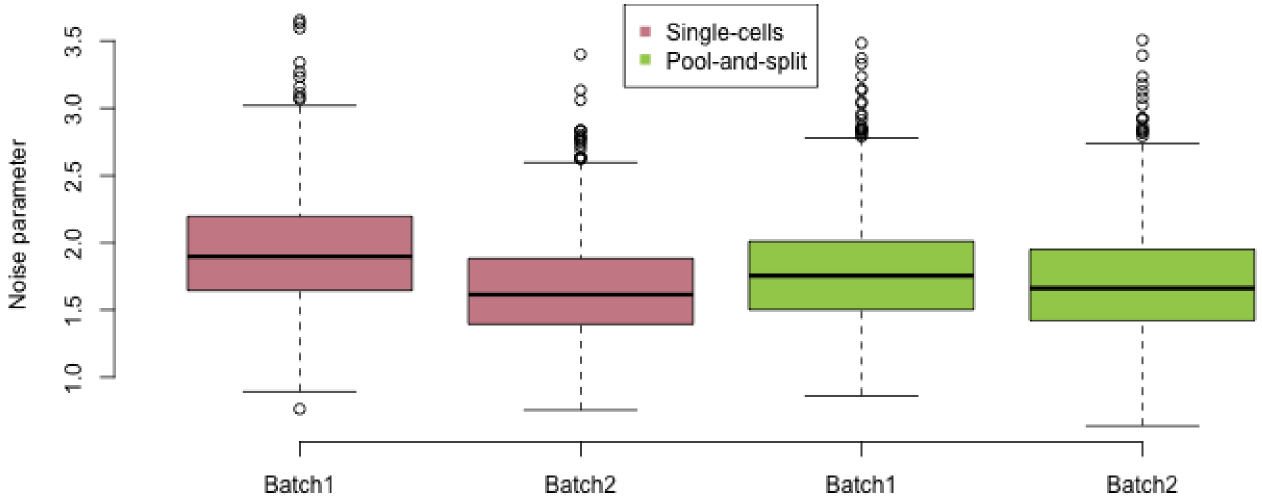

Offset correction

Once the model has been fitted, possible offset effects are corrected using the following function.

```
OffSetCorrection <- function(MCMC_Output)
{
  median(rowSums(MCMC_Output@muRef)/rowSums(MCMC_Output@muTest))
}
```

```
OffSet = OffSetCorrection(MCMC_Output)
OffSet
```

```
## [1] 1.472231
```

Hereafter, results are displayed based on offset corrected chains.

```
MCMC_Output2 <- newBASiCS_D_Chain(muTest = ChainMuTest,
                                  muRef = ChainMuRef / OffSet,
                                  deltaTest = ChainDeltaTest,
                                  deltaRef = ChainDeltaRef,
                                  phi = cbind(ChainPhiTest, ChainPhiRef * OffSet),
                                  s = cbind(ChainSTest, ChainSRef),
                                  nu = cbind(ChainNuTest, ChainNuRef),
                                  thetaTest = ChainThetaTest,
                                  thetaRef = ChainThetaRef)
```

```
## An object of class BASiCS_D_Chain
##  1000 MCMC samples.
##  Dataset contains 9343 biological genes and 150 cells (in total across both samples).
##  Offset = 1.
##  Elements (slots): muTest, muRef, deltaTest, omegaRef, phi, s, nu, thetaTest, thetaRef and offset.
```

Differential expression analysis (mean and over-dispersion)

To visualise gene-wide behaviour of log-fold change estimates, we create the following plots.

```
MedianMuRef = apply(MCMC_Output2@muRef, 2, median)[!Data@Tech]
MedianMuTest = apply(MCMC_Output2@muTest, 2, median)[!Data@Tech]
MuBase=(MedianMuRef * nRef + MedianMuTest * nTest)/n

ChainTau = log(MCMC_Output2@muTest / MCMC_Output2@muRef)
ChainOmega = log(MCMC_Output2@deltaTest / MCMC_Output2@deltaRef)
MedianTau = apply(ChainTau, 2, median)
MedianOmega = apply(ChainOmega, 2, median)

par(mfrow = c(1,2))
par(mar = c(5, 6, 5, 2) + 0.1)
par(cex.axis = 1.5)

plot(MuBase, MedianTau, pch = 16,
     col = rgb(col2rgb("grey")[1],
               col2rgb("grey")[2],
               col2rgb("grey")[3],50,maxColorValue=255),
     bty = "n", log = "x",
     xlab = "Average expression rate", cex.lab = 2,
     ylab = "LFC in overall expression")
abline(h = 0, lty = 2)

plot(MuBase, MedianOmega, pch = 16,
     col = rgb(col2rgb("grey")[1],
               col2rgb("grey")[2],
               col2rgb("grey")[3],50,maxColorValue=255),
     bty = "n", log = "x",
     xlab = "Average expression rate", cex.lab = 2,
     ylab = "LFC in over-dispersion")
abline(h = 0, lty = 2)
```

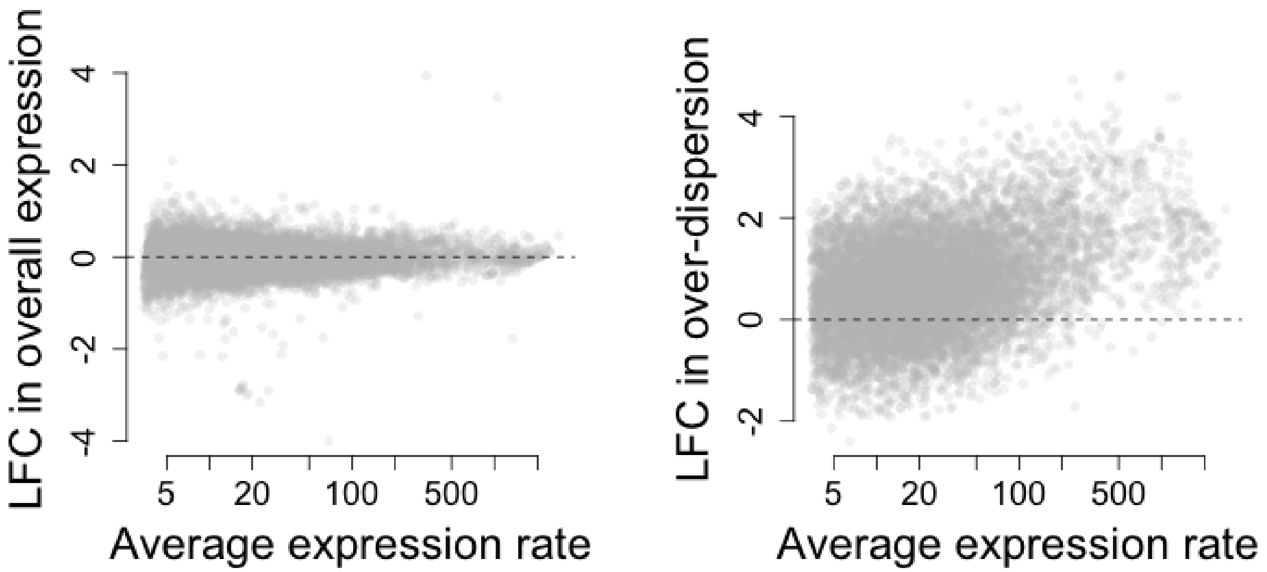

```
Test40 <- BASiCS_D_TestDE(Data, MCMC_Output2, GeneNames = Data@GeneNames[!Data@Tech],
                          EpsilonM = 0.4, EpsilonD = 0.4,
                          EFDR_M = 0.05, EFDR_D = 0.05,
                          OrderVariable = "GeneIndex",
                          GroupLabelRef = "P&S", GroupLabelTest = "SC")
```

```
## -------------------------------------------------------------------------
## 554  genes with a change on the overall expression:
## - Higher expression in  SC group: 252
## - Higher expression in  P&S group: 302
## - Fold change tolerance =  40 %
## - Evidence threshold =  0.85725
## - EFDR =  5 %
## - EFNR =  18.67 %
## -------------------------------------------------------------------------
##
## -------------------------------------------------------------------------
## 2388  genes with a change on the cell-to-cell biological over dispersion:
## - Higher over dispersion in  SC group: 2317
## - Higher over dispersion in  P&S group: 71
## - Fold change tolerance =  40 %
## - Evidence threshold =  0.87825
## - EFDR =  5.03 %
## - EFNR =  76.23 %
## -------------------------------------------------------------------------
```

```
Test0 <- BASiCS_D_TestDE(Data, MCMC_Output2, GeneNames = Data@GeneNames[!Data@Tech],
                          EpsilonM = 0, EpsilonD = 0,
                          EFDR_M = 0.05, EFDR_D = 0.05,
                          OrderVariable = "GeneIndex",
                          GroupLabelRef = "P&S", GroupLabelTest = "SC")
```

```
## -------------------------------------------------------------------------
## 4711  genes with a change on the overall expression:
## - Higher expression in  SC group: 1914
## - Higher expression in  P&S group: 2797
## - Fold change tolerance =  0 %
## - Evidence threshold =  0.79675
## - EFDR =  5.02 %
## - EFNR =  43.19 %
## -------------------------------------------------------------------------
##
## -------------------------------------------------------------------------
## 2274  genes with a change on the cell-to-cell biological over dispersion:
## - Higher over dispersion in  SC group: 2213
## - Higher over dispersion in  P&S group: 61
## - Fold change tolerance =  0 %
## - Evidence threshold =  0.84275
## - EFDR =  5.04 %
## - EFNR =  41.83 %
## -------------------------------------------------------------------------
```

```
par(mfrow = c(2,2))
par(mar = c(5, 6, 4, 2) + 0.1, oma = c(0,0,3,0))
par(cex.axis = 1.5, cex.lab = 2)
MedianTauAux0 = ifelse(abs(Test0$Table$ExpLogFC) > 1.5,
                      1.5 * sign(Test0$Table$ExpLogFC), Test0$Table$ExpLogFC)
pchAux = ifelse(abs(Test0$Table$ExpLogFC) > 1.5, 4, 16)
plot(MedianTauAux0, Test0$Table$ProbDiffExp, bty = "n",
     main = expression(paste(tau[0], "= 0")), cex.main = 2.5,
     xlab = "LFC in overall expression",
     ylab = "Posterior probability",
     pch = 16, ylim = c(0, 1),
     col = rgb(col2rgb("grey")[1],
               col2rgb("grey")[2],
               col2rgb("grey")[3],50,maxColorValue=255),
     xlim = c(-1.5, 1.5))
points(MedianTauAux0[Test0$Table$ResultDiffExp == "SC+"],
       Test0$Table$ProbDiffExp[Test0$Table$ResultDiffExp == "SC+"],
       pch = 16,
       col = rgb(col2rgb("lightpink3")[1],
               col2rgb("lightpink3")[2],
               col2rgb("lightpink3")[3],50,maxColorValue=255))
points(MedianTauAux0[Test0$Table$ResultDiffExp == "P&S+"],
       Test0$Table$ProbDiffExp[Test0$Table$ResultDiffExp == "P&S+"],
       pch = 16,
       col = rgb(col2rgb("darkolivegreen3")[1],
               col2rgb("darkolivegreen3")[2],
               col2rgb("darkolivegreen3")[3],50,maxColorValue=255))
abline(h = Test0$DiffExpSummary$EviThreshold, lty = 2, lwd = 3)

plot(Test0$Table$OverDispLogFC, Test0$Table$ProbDiffOverDisp, bty = "n",
     main = expression(paste(omega[0], "= 0")), cex.main = 2.5,
     xlab = "LFC in over-dispersion",
     ylab = "Posterior probability",
     pch = 16, ylim = c(0, 1),
     col = rgb(col2rgb("grey")[1],
               col2rgb("grey")[2],
               col2rgb("grey")[3],50,maxColorValue=255),
     xlim = c(-4, 4))
points(Test0$Table$OverDispLogFC[Test0$Table$ResultDiffOverDisp == "SC+"],
       Test0$Table$ProbDiffOverDisp[Test0$Table$ResultDiffOverDisp == "SC+"],
       pch = 16,
       col = rgb(col2rgb("lightpink3")[1],
               col2rgb("lightpink3")[2],
               col2rgb("lightpink3")[3],50,maxColorValue=255))
points(Test0$Table$OverDispLogFC[Test0$Table$ResultDiffOverDisp == "P&S+"],
       Test0$Table$ProbDiffOverDisp[Test0$Table$ResultDiffOverDisp == "P&S+"],
       pch = 16,
       col = rgb(col2rgb("darkolivegreen3")[1],
               col2rgb("darkolivegreen3")[2],
               col2rgb("darkolivegreen3")[3],50,maxColorValue=255))
abline(h = Test0$DiffOverDispSummary$EviThreshold, lty = 2, lwd = 3)

MedianTauAux40 = ifelse(abs(Test40$Table$ExpLogFC) > 1.5,
```

```
                       1.5 * sign(Test40$Table$ExpLogFC), Test40$Table$ExpLogFC)
plot(MedianTauAux40, Test40$Table$ProbDiffExp, bty = "n",
     main = expression(paste(tau[0], "= 0.4")), cex.main = 2.5,
     xlab = "LFC in overall expression",
     ylab = "Posterior probability",
     pch = 16, ylim = c(0, 1),
     col = rgb(col2rgb("grey")[1],
               col2rgb("grey")[2],
               col2rgb("grey")[3],50,maxColorValue=255),
     xlim = c(-1.5, 1.5))
points(MedianTauAux40[Test40$Table$ResultDiffExp == "SC+"],
       Test40$Table$ProbDiffExp[Test40$Table$ResultDiffExp == "SC+"],
       pch = 16,
       col = rgb(col2rgb("lightpink3")[1],
               col2rgb("lightpink3")[2],
               col2rgb("lightpink3")[3],50,maxColorValue=255))
points(MedianTauAux40[Test40$Table$ResultDiffExp == "P&S+"],
       Test40$Table$ProbDiffExp[Test40$Table$ResultDiffExp == "P&S+"],
       pch = 16,
       col = rgb(col2rgb("darkolivegreen3")[1],
               col2rgb("darkolivegreen3")[2],
               col2rgb("darkolivegreen3")[3],50,maxColorValue=255))
abline(h = Test40$DiffExpSummary$EviThreshold, lty = 2, lwd = 3)

plot(Test40$Table$OverDispLogFC, Test40$Table$ProbDiffOverDisp, bty = "n",
     main = expression(paste(omega[0], "= 0.4")), cex.main = 2.5,
     xlab = "LFC in over-dispersion",
     ylab = "Posterior probability",
     pch = 16, ylim = c(0, 1),
     col = rgb(col2rgb("grey")[1],
               col2rgb("grey")[2],
               col2rgb("grey")[3],50,maxColorValue=255),
     xlim = c(-4, 4))
points(Test40$Table$OverDispLogFC[Test40$Table$ResultDiffOverDisp == "SC+"],
       Test40$Table$ProbDiffOverDisp[Test40$Table$ResultDiffOverDisp == "SC+"],
       pch = 16,
       col = rgb(col2rgb("lightpink3")[1],
               col2rgb("lightpink3")[2],
               col2rgb("lightpink3")[3],50,maxColorValue=255))
points(Test40$Table$OverDispLogFC[Test40$Table$ResultDiffOverDisp == "P&S+"],
       Test40$Table$ProbDiffOverDisp[Test40$Table$ResultDiffOverDisp == "P&S+"],
       pch = 16,
       col = rgb(col2rgb("darkolivegreen3")[1],
               col2rgb("darkolivegreen3")[2],
               col2rgb("darkolivegreen3")[3],50,maxColorValue=255))
abline(h = Test40$DiffOverDispSummary$EviThreshold, lty = 2, lwd = 3)

title("(a)", outer=TRUE, cex.main = 2.5)
```

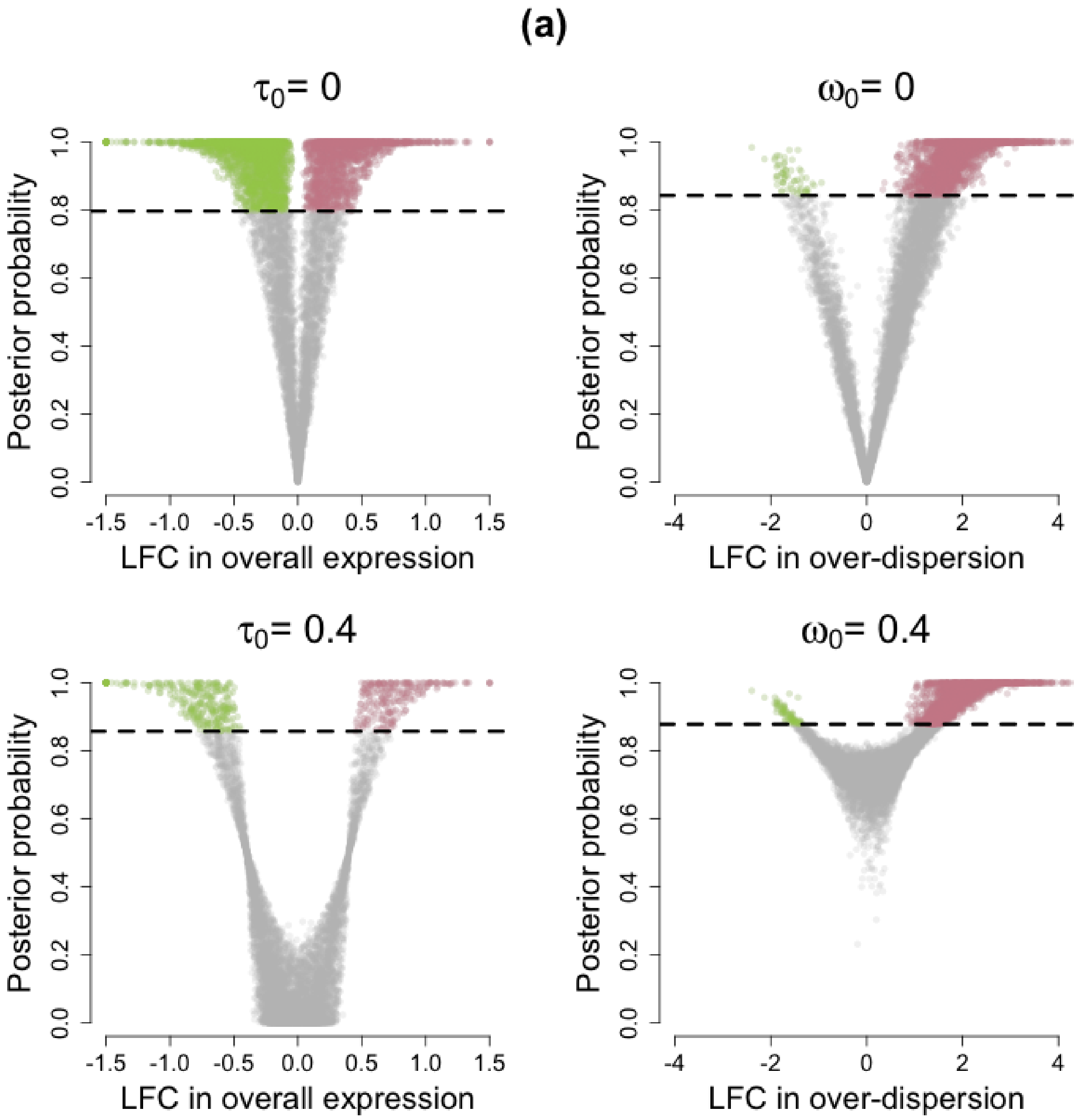


```
Result0 = paste0(Test0$Table$ResultDiffExp,Test0$Table$ResultDiffOverDisp)

NamesBarplot = c(names(table(Result0)))
ColourBarplot = c(rep(unique(Cell.Colour_1)[1], 3),
                  rep("grey", 3),
                  rep(unique(Cell.Colour_1)[2], 3))

par(mfrow = c(2,1))
par(mar = c(4, 4, 4, 2) + 0.1, oma = c(0,0,3,0))
#par(mgp = c(4, 1, 0))
par(cex.main = 2, cex.axis = 1.5, cex.lab = 1.5)
```

```
barplot(table(Test0$Table$ResultDiffOverDisp,
              Test0$Table$ResultDiffExp)[c(3,1,2),c(3,1,2)] + 1,
        col = unique(ColourBarplot), beside = TRUE, cex.names = 1.7,
        ylim = c(1, 35000), xlab = "Changes in overall expression",
        main = expression(paste(tau[0], "= 0, ", omega[0], "= 0")),
        ylab = "Number of genes", axes = FALSE, log = "y",
        names.arg = c("SC +", "No diff.", "P&S +"))
text(x = c(1.3, 2.5, 3.5, 5.3, 6.5, 7.5, 9.3, 10.5, 11.5),
     y = 3 * as.vector(table(Test0$Table$ResultDiffOverDisp,
                               Test0$Table$ResultDiffExp)[c(3,1,2),c(3,1,2)]),
     as.vector(table(Test0$Table$ResultDiffOverDisp,
                               Test0$Table$ResultDiffExp)[c(3,1,2),c(3,1,2)]),
     cex = 1.5, col = "black")


barplot(table(Test40$Table$ResultDiffOverDisp,
              Test40$Table$ResultDiffExp)[c(3,1,2),c(3,1,2)] + 1,
        col = unique(ColourBarplot), beside = TRUE, cex.names = 1.7,
        ylim = c(1, 35000), xlab = "Changes in overall expression",
        main = expression(paste(tau[0], "= 0.4, ", omega[0], "= 0.4")),
        ylab = "Number of genes", axes = FALSE, log = "y",
        names.arg = c("SC +", "No diff.", "P&S +"))
text(x = c(1.5, 2.5, 3.5, 5.2, 6.5, 7.5, 9.3, 10.5, 11.5),
     y = pmax(5, 3 * as.vector(table(Test40$Table$ResultDiffOverDisp,
                               Test40$Table$ResultDiffExp)[c(3,1,2),c(3,1,2)])),
     as.vector(table(Test40$Table$ResultDiffOverDisp,
                               Test40$Table$ResultDiffExp)[c(3,1,2),c(3,1,2)]),
     cex = 1.5, col = "black")

title("(b)", outer=TRUE, cex.main = 2)
```

(b)

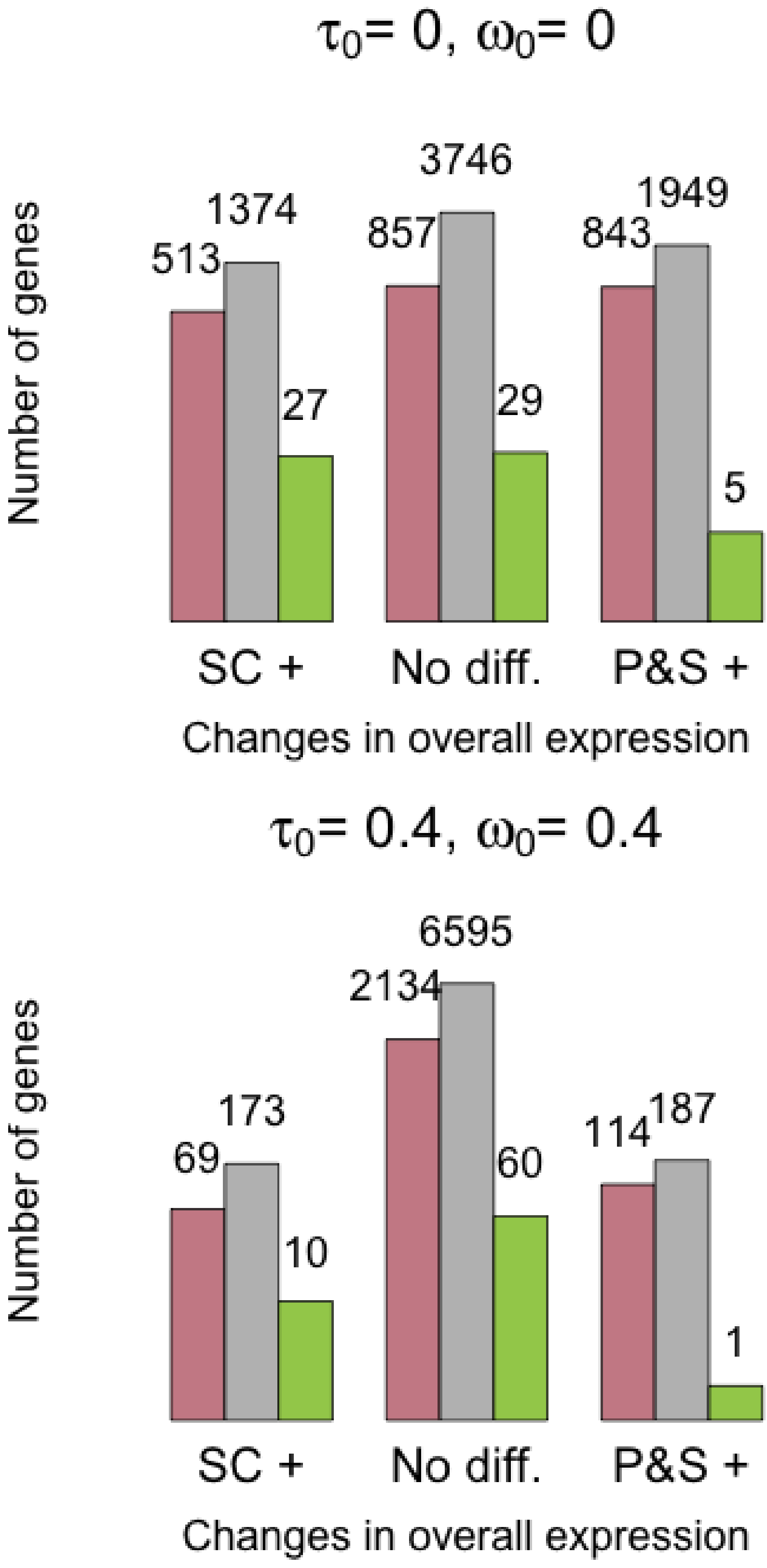

τ0= 0, ω0= 0
Number of genes
513
1374
27
857
3746
29
843
1949
5
SC +
No diff.
P&S +
Changes in overall expression
τ0= 0.4, ω0= 0.4
Number of genes
69
173
10
2134
6595
60
114
187
1
SC +
No diff.
P&S +
Changes in overall expression

Alternative approaches for identifying changes in mean

Preparing the data required to perform alternative analyses.

```
CountsBio <- cbind(counts(Data, type = "biological", samples = "test"),
                   counts(Data, type = "biological", samples = "reference"))
```

```
## Samples in the test group
## Samples in the reference group
```

```
CountsBio <- apply(CountsBio, 2, function(x) {storage.mode(x) <- 'integer'; x})
CountsTech <- cbind(counts(Data, type = "technical", samples = "test"),
                   counts(Data, type = "technical", samples = "reference"))
```

```
## Samples in the test group
## Samples in the reference group
```

```
sg <- factor(gsub("(SC|RNA).*", "\\1", colnames(CountsBio)), levels = c("SC", "RNA"))
names(sg) <- colnames(CountsBio)
table(sg)
```

```
## sg
##  SC RNA
##  74  76
```

```
colData = data.frame("Group" = sg, "Batch" = factor(Batch_1))

RPMBio=1000000*CountsBio/colSums(CountsBio)
```

In all cases, analysis was run once using this code and results were stored. This allows faster compilation of the vignette.

DESeq2

```
library(DESeq2)
packageVersion("DESeq2")
```

```
## [1] '1.10.1'
```

```
dds <- DESeqDataSetFromMatrix(countData = CountsBio,
                              colData = colData,
                              design = ~ Group + Batch)
dds <- DESeq(dds)

# tau0 = 0
res0 <- results(dds, contrast = c("Group", "SC", "RNA"), alpha=0.05)
# tau0 = 0.4
res40 <- results(dds, contrast = c("Group", "SC", "RNA"),
```

```
                 lfcThreshold=log2(1.5), altHypothesis="greaterAbs", alpha=0.05)

write.table(res0, file.path(results.path, "DESeq2results0.txt"),
            col.names = T, row.names = T)
write.table(res40, file.path(results.path, "DESeq2results40.txt"),
            col.names = T, row.names = T)
```

```
res0 = DESeqResults(DataFrame(read.table(file.path(results.path,
                                                   "DESeq2results0.txt"), header = T)))
res40 = DESeqResults(DataFrame(read.table(file.path(results.path,
                                                     "DESeq2results40.txt"), header = T)))

summary(res0, alpha = 0.05)
```

```
##
## out of 9343 with nonzero total read count
## adjusted p-value < 0.05
## LFC > 0 (up)      : 857, 9.2%
## LFC < 0 (down)    : 1157, 12%
## outliers [1]      : 0, 0%
## low counts [2]    : 0, 0%
## (mean count < 0)
## [1] see 'cooksCutoff' argument of ?results
## [2] see 'independentFiltering' argument of ?results
```

```
summary(res40, alpha = 0.05)
```

```
##
## out of 9343 with nonzero total read count
## adjusted p-value < 0.05
## LFC > 0 (up)      : 26, 0.28%
## LFC < 0 (down)    : 34, 0.36%
## outliers [1]      : 0, 0%
## low counts [2]    : 0, 0%
## (mean count < 0)
## [1] see 'cooksCutoff' argument of ?results
## [2] see 'independentFiltering' argument of ?results
```

```
par(mfrow = c(1,2))
plotMA(res0, main="DESeq2", ylim=c(-2,2), alpha = 0.05)
plotMA(res40, main="DESeq2", ylim=c(-2,2), alpha = 0.05)
```

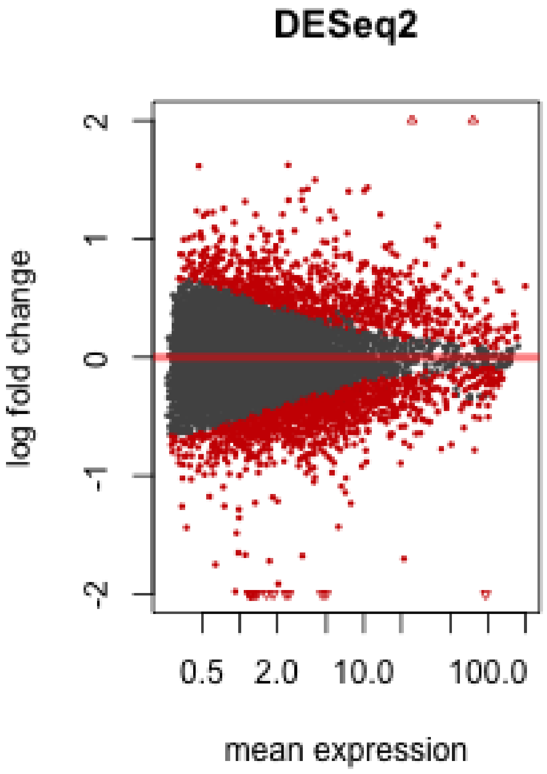

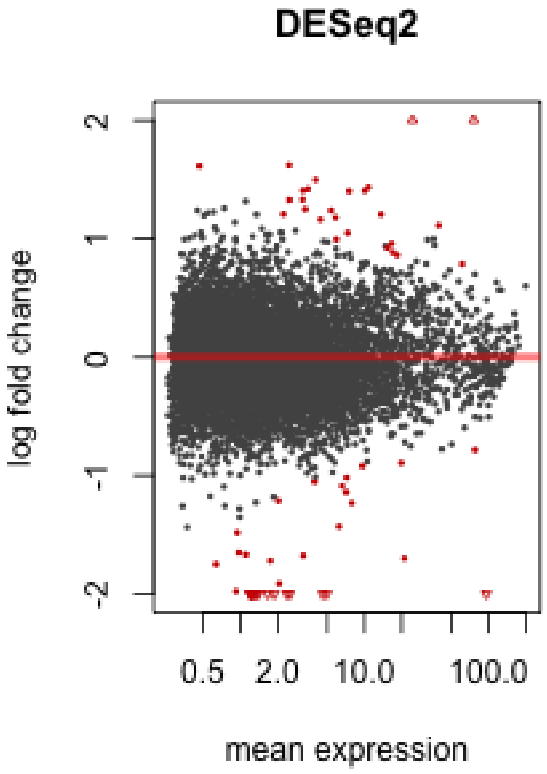


```
ResultsDESeq2_0 = cbind.data.frame("GeneNames" = row.names(res0),
                                   "LFC" = - res0$log2FoldChange * log(2),
                                   "Evidence" = res0$padj,
                                   "Result" = ifelse(res0$padj < 0.05,
                                                     ifelse(-res0$log2FoldChange > 0,
                                                            "SC+", "P&S+"), "NoDiff"))

ResultsDESeq2_40 = cbind.data.frame("GeneNames" = row.names(res40),
                                   "LFC" = - res40$log2FoldChange * log(2),
                                   "Evidence" = res40$padj,
                                   "Result" = ifelse(res40$padj < 0.05,
                                                     ifelse(-res40$log2FoldChange > 0,
                                                            "SC+", "P&S+"), "NoDiff"))
```

SCDE

```
library(scde)
packageVersion("scde")
```

```
## [1] '1.99.1'
```

```
o.ifm <- scde.error.models(counts = CountsBio,
                             groups = sg,
                             n.cores = 4,
                             threshold.segmentation = TRUE,
                             save.crossfit.plots = FALSE,
```

```r
                              save.model.plots = FALSE,
                              verbose = 1)
write.table(o.ifm, file.path(results.path, "o.ifm.txt"), col.names = T, row.names = T)

o.ifm <- read.table(file.path(results.path, "o.ifm.txt"), header = T)
row.names(o.ifm) <- read.table(file.path(results.path, "row.names.o.ifm.txt"))[,1]

head(o.ifm)

# filter out cells that don't show positive correlation with
# the expected expression magnitudes (very poor fits)
valid.cells <- o.ifm$corr.a > 0
table(valid.cells)

o.ifm <- o.ifm[valid.cells, ]

# estimate gene expression prior
o.prior <- scde.expression.prior(models = o.ifm, counts = CountsBio,
                                  length.out = 400, show.plot = FALSE)

# define two groups of cells
groups <- factor(gsub("(SC|RNA).*", "\\1", rownames(o.ifm)), levels = c("SC", "RNA"))
names(groups) <- row.names(o.ifm)
# run differential expression tests on all genes.
ediff <- scde.expression.difference(o.ifm, CountsBio,
                                     o.prior, groups = groups,
                                     n.randomizations  =  100,
                                     n.cores  =  3, verbose  =  1,
                                     batch = colData$Batch)

head(ediff$batch.adjusted[order(abs(ediff$batch.adjusted$cZ), decreasing  =  TRUE), ])

write.table(ediff$batch.adjusted,
            file = file.path(results.path,"SCDEresultsBatchAdjusted.txt"),
            row.names = TRUE, col.names = TRUE, sep = "\t", quote = FALSE)

write.table(ediff$results,
            file = file.path(results.path,"SCDEresults.txt"),
            row.names = TRUE, col.names = TRUE, sep = "\t", quote = FALSE)

write.table(ediff$batch.effect,
            file = file.path(results.path,"SCDEresultsBatchEffect.txt"),
            row.names = TRUE, col.names = TRUE, sep = "\t", quote = FALSE)
```

```r
ediff <- read.table(file.path(results.path,"SCDEresultsBatchAdjusted.txt"), header = T)
# adjusted p-value
ediff$adj.p.value = 2*(1-pnorm(abs(ediff$cZ)))

# Results
table(ediff$adj.p.value < 0.05)
```

```
##
## FALSE  TRUE
```

```
## 9142 201
```

```
ResultsSCDE = cbind.data.frame("GeneNames" = row.names(ediff),
                               "LFC" = ediff$ce * log(2),
                               "Evidence" = ediff$adj.p.value,
                               "Result" = ifelse(ediff$adj.p.value < 0.05,
                                                 ifelse(ediff$Z > 0,
                                                        "SC+", "P&S+"), "NoDiff"))
```

edgeR

```
library(edgeR)
packageVersion("edgeR")
```

```
## [1] '3.12.0'
```

```
cds <- DGEList( CountsBio , group = colData$Group )
cds <- calcNormFactors( cds )
design = model.matrix(~colData$Group + colData$Batch)

cds <- estimateGLMCommonDisp( cds, design, verbose = TRUE )
```

```
## Disp = 0.04653 , BCV = 0.2157
```

```
# This is the recommendation, it doesn't work because we have too many samples
# Instead we use prior.df = 10 as in edgeR vignette, batch effect example
#cds <- estimateTagwiseDisp( cds , prior.n = 50 / (ncol(CountsBio) - 2) )
cds <- estimateGLMTrendedDisp(cds, design)
cds <- estimateGLMTagwiseDisp(cds, design, prior.df=10)

fit <- glmFit(cds, design)

# Using different test because of message: Zero log2-FC threshold detected.
# Switch to glmLRT() instead
tr0 = glmLRT(fit, coef = 2)
tr40 <- glmTreat(fit, coef = 2, lfc = log2(1.5))

edgeR0 = topTags(tr0, n = Inf, adjust.method = "BH")$table
edgeR40 = topTags(tr40, n = Inf, adjust.method = "BH")$table

summary(de0 <- decideTestsDGE(tr0), adjust.method="BH", p.value=0.05, lfc=0)
```

```
##    [,1]
## -1  631
## 0  6779
## 1  1933
```

```
summary(de40 <- decideTestsDGE(tr40), adjust.method="BH", p.value=0.05, lfc=log2(1.5))
```

```
##     [,1]
## -1    49
## 0   9182
## 1    112
```

```
ResultsedgeR_0 = cbind.data.frame("GeneNames" = row.names(edgeR0),
                                  "LFC" = - edgeR0$logFC * log(2),
                                  "Evidence" = edgeR0$FDR,
                                  "Result" = ifelse(edgeR0$FDR < 0.05,
                                                    ifelse(-edgeR0$logFC > 0,
                                                           "SC+", "P&S+"), "NoDiff"))

ResultsedgeR_40 = cbind.data.frame("GeneNames" = row.names(edgeR40),
                                   "LFC" = - edgeR40$logFC * log(2),
                                   "Evidence" = edgeR40$FDR,
                                   "Result" = ifelse(edgeR40$FDR < 0.05,
                                                     ifelse(-edgeR40$logFC > 0,
                                                            "SC+", "P&S+"), "NoDiff"))

par(mfrow = c(1,2))
plotSmear(tr0, de.tags=rownames(cds)[as.logical(de0)], main = "tau0 = 0")
plotSmear(tr40, de.tags=rownames(cds)[as.logical(de40)], main = "tau0 = 0.4")
```

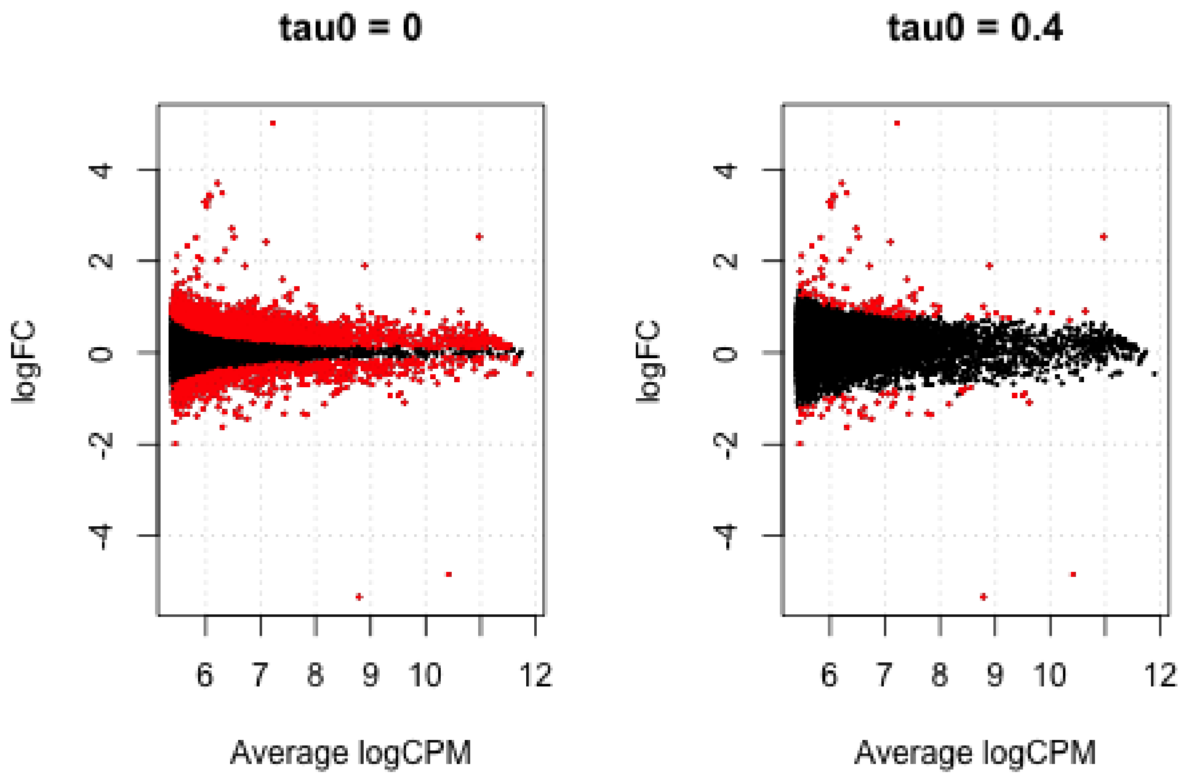

MAST

```
#library(devtools)
#install_github("RGLab/MAST")
```

```r
library(MAST)
packageVersion("MAST")
```

```
## [1] '0.933'
```

```r
ngeneson <- apply(t(log(RPMBio+1)),1,function(x)mean(x>0))
ngenesonS <- apply(t(log(CountsTech+1)),1,function(x)mean(x>0))
dataMAST <- FromMatrix('SingleCellAssay', t(log(RPMBio+1)),
                       fData = data.frame("primerid" = rownames(CountsBio)),
                       cData = data.frame("wellKey" = colnames(CountsBio),
                                          "Population" = colData$Group,
                                          "ngeneson" = ngeneson,
                                          "cngeneson" = ngeneson - mean(ngeneson),
                                          "ngenesonS" = ngenesonS,
                                          "cngenesonS" = ngenesonS - mean(ngenesonS),
                                          "Batch" = colData$Batch))

zlm.output <- zlm.SingleCellAssay(~ Population + cngeneson + Batch, dataMAST,
                                  method='bayesglm',
                                  ebayes = TRUE,
                                  ebayesControl = list(method = "MLE", model = "H1"))

zlm.lr <- lrTest(zlm.output, 'Population')

# BH correction
adj.p.cont = p.adjust(zlm.lr[,'cont','Pr(>Chisq)'], "BH")
adj.p.disc = p.adjust(zlm.lr[,'disc','Pr(>Chisq)'], "BH")
adj.p.hurdle = p.adjust(zlm.lr[,'hurdle','Pr(>Chisq)'], "BH")

write.table(adj.p.cont, file.path(results.path, "adj.p.cont.txt"),
            row.names = T, col.names = T)
write.table(adj.p.disc, file.path(results.path, "adj.p.disc.txt"),
            row.names = T, col.names = T)
write.table(adj.p.hurdle, file.path(results.path, "adj.p.hurdle.txt"),
            row.names = T, col.names = T)
```

```r
adj.p.cont = read.table(file.path(results.path, "adj.p.cont.txt"))
adj.p.disc = read.table(file.path(results.path, "adj.p.disc.txt"))
adj.p.hurdle = read.table(file.path(results.path, "adj.p.hurdle.txt"))

table(adj.p.cont < 0.05)
```

```
##
## FALSE  TRUE
##  9053   290
```

```r
table(adj.p.disc < 0.05)
```

```
##
## FALSE  TRUE
##  9253    90
```

```r
table(adj.p.hurdle < 0.05)
```

```
##
## FALSE  TRUE
##  8987   356
```

```r
ResultsMAST = cbind.data.frame("GeneNames" = row.names(adj.p.cont),
                           "EvidenceCont" = adj.p.cont$x,
                           "EvidenceDisc" = adj.p.disc$x,
                           "EvidenceHurdle" = adj.p.hurdle$x,
                           "ResultsCont" = ifelse(adj.p.cont$x < 0.05, "Diff", "NoDiff"),
                           "ResultsDisc" = ifelse(adj.p.disc$x < 0.05, "Diff", "NoDiff"),
                           "ResultsHurdle" = ifelse(adj.p.hurdle$x < 0.05, "Diff", "NoDiff"))
```

```r
zlm.outputNotCDR <- zlm.SingleCellAssay(~ Population + Batch, dataMAST,
                                  method='bayesglm',
                                  ebayes = TRUE,
                                  ebayesControl = list(method = "MLE", model = "H1"))
show(zlm.outputNotCDR)
summary(zlm.outputNotCDR)

zlm.lrNotCDR <- lrTest(zlm.outputNotCDR, 'Population')

# BH correction
adj.p.contNotCDR = p.adjust(zlm.lrNotCDR[,'cont','Pr(>Chisq)'], "BH")
adj.p.discNotCDR = p.adjust(zlm.lrNotCDR[,'disc','Pr(>Chisq)'], "BH")
adj.p.hurdleNotCDR = p.adjust(zlm.lrNotCDR[,'hurdle','Pr(>Chisq)'], "BH")

write.table(adj.p.contNotCDR, file.path(results.path, "adj.p.contNotCDR.txt"),
            row.names = T, col.names = T)
write.table(adj.p.discNotCDR, file.path(results.path, "adj.p.discNotCDR.txt"),
            row.names = T, col.names = T)
write.table(adj.p.hurdleNotCDR, file.path(results.path, "adj.p.hurdleNotCDR.txt"),
            row.names = T, col.names = T)
```

```r
adj.p.contNotCDR = read.table(file.path(results.path, "adj.p.contNotCDR.txt"))
adj.p.discNotCDR = read.table(file.path(results.path, "adj.p.discNotCDR.txt"))
adj.p.hurdleNotCDR = read.table(file.path(results.path, "adj.p.hurdleNotCDR.txt"))

table(adj.p.contNotCDR < 0.05)
```

```
##
## FALSE  TRUE
##  6662  2681
```

```r
table(adj.p.discNotCDR < 0.05)
```

```
##
## FALSE  TRUE
##  5845  3498
```

```r
table(adj.p.hurdleNotCDR < 0.05)
```

```
##
## FALSE  TRUE
##  4220  5123
```

```r
ResultsMASTNotCDR = cbind.data.frame("GeneNames" = row.names(adj.p.contNotCDR),
                            "EvidenceCont" = adj.p.contNotCDR$x,
                            "EvidenceDisc" = adj.p.discNotCDR$x,
                            "EvidenceHurdle" = adj.p.hurdleNotCDR$x,
                            "ResultsCont" = ifelse(adj.p.contNotCDR$x < 0.05, "Diff", "NoDiff"),
                            "ResultsDisc" = ifelse(adj.p.discNotCDR$x < 0.05, "Diff", "NoDiff"),
                            "ResultsHurdle" = ifelse(adj.p.hurdleNotCDR$x < 0.05, "Diff", "NoDiff"))
```

```r
GenesDE = c(sum(Test0$Table$ResultDiffExp != "NoDiff"), # 1
                sum(Test40$Table$ResultDiffExp != "NoDiff"), # 2
                sum(ResultsDESeq2_0$Result != "NoDiff"), # 3
                sum(ResultsDESeq2_40$Result != "NoDiff"), # 4
                sum(ResultsedgeR_0$Result != "NoDiff"), # 5
                sum(ResultsedgeR_40$Result != "NoDiff"), # 6
                sum(ResultsSCDE$Result != "NoDiff"), # 7
                sum(ResultsMAST$ResultsHurdle != "NoDiff"), # 8
                sum(ResultsMASTNotCDR$ResultsHurdle != "NoDiff")) # 9

rainbowcols <- rainbow(9, s = 0.3)

bplt <- barplot(GenesDE, col = rainbowcols, names.arg = 1:length(GenesDE), ylim = c(0, 5700),
                ylab = "No. of detected genes", xlab = "Method", cex.lab = 1.5)
text(x = bplt, y = GenesDE + 250, GenesDE, cex = 1.5)
```

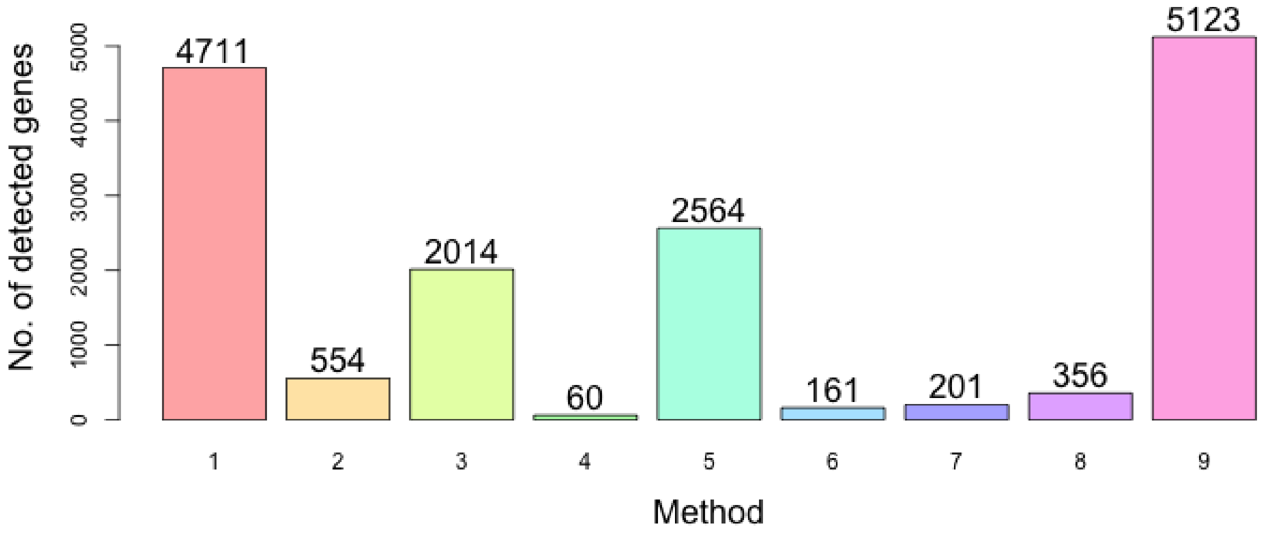

Supplement: Supplementary file 2 — Data analysis (part 1). R code used to analyze the single cells vs pool-and-split samples data set. (PDF 3952 kb) [file 13059_2016_930_MOESM2_ESM.pdf]
