# Supplementary material for: Beyond comparisons of means: understanding changes in gene expression at the single-cell level
Source: Genome Biol. 2016 Apr 15;17:70. doi: 10.1186/s13059-016-0930-3 (PMC4832562; doi:10.1186/s13059-016-0930-3)
Supplement: Supplementary file 3 — Data analysis (part 2). R code used to analyze the cell-cycle data set. (PDF 3051 kb) [file 13059_2016_930_MOESM3_ESM.pdf]

Comparison of mESCs between cell cycle phases

Catalina A. Vallejos, Sylvia Richardson and John C. Marioni

20 December 2015

Our second example shows the analysis of the mESC dataset presented in Buettner et al (2015), which contains cells for which the cell cycle phase is known (G1, S and G2M). To start the analysis, the following data must be dowloaded and stored in `data.path` directory.

- Expression counts. Files 'G1_dec5_83c_counttable.txt', 'G2M_dec5_89c_counttable.txt' and 'S_dec6_73c_counttable.txt'.

Additionally, the following R libraries must be loaded before performing the analysis

```
library(BASiCS) # To run the analysis
packageVersion("BASiCS")
```

```
## [1] '0.5.3'
```

```
library(data.table) # For fast loading and processing of large datasets
packageVersion("data.table")
```

```
## [1] '1.9.6'
```

Data pre-processing

Loading the data

```
# Reading the expression counts
dataG1=fread(file.path(data.path,"G1_dec5_83c_counttable.txt"))
dataS=fread(file.path(data.path,"S_dec6_73c_counttable.txt"))
dataG2M=fread(file.path(data.path,"G2M_dec5_89c_counttable.txt"))

# Genes id
genes.id=dataG1$EnsemblGeneID

# Removing information that is not required
dataG1[,EnsemblTranscriptID:=NULL]; dataG1[,AssociatedGeneName:=NULL]; dataG1[,GeneLength:=NULL]
dataS[,EnsemblTranscriptID:=NULL]; dataS[,AssociatedGeneName:=NULL]; dataS[,GeneLength:=NULL]
dataG2M[,EnsemblTranscriptID:=NULL]; dataG2M[,AssociatedGeneName:=NULL]; dataG2M[,GeneLength:=NULL]

# Creating an idicator of spike-in genes
TechAux=rep(F,times=length(genes.id))
TechAux[grep("ERCC",genes.id)]=T
table(TechAux) # 38293 endogenous genes,   92 spike-in genes
```

Filtering cells

Firstly, we remove the same poor quality control samples as in Buettner et al (2015). The indexes of these cells are contained in the vectors `RemoveG1`, `RemoveS` and `RemoveG2M`. Additionally, as in Buettner et al (2015), we filter cells based on the ratio between endogenous reads and total mapped reads.

```
RatioG1=colSums(dataG1[!TechAux,-1,with=F])/colSums(dataG1[,-1,with=F])
RatioS=colSums(dataS[!TechAux,-1,with=F])/colSums(dataS[,-1,with=F])
RatioG2M=colSums(dataG2M[!TechAux,-1,with=F])/colSums(dataG2M[,-1,with=F])

IncludeG1=which(abs(RatioG1-median(RatioG1)) < mad(RatioG1))
IncludeS=which(abs(RatioS-median(RatioS)) < mad(RatioS))
IncludeG2M=which(abs(RatioG2M-median(RatioG2M)) < mad(RatioG2M))

# Merging all three datasets into one.
setkey(dataG1,EnsemblGeneID); setkey(dataG2M,EnsemblGeneID); setkey(dataS,EnsemblGeneID);
Counts=merge(dataG1[,c(1,IncludeG1+1),with=F],
             dataG2M[,c(1,IncludeG2M+1),with=F],all=T)
setkey(Counts,EnsemblGeneID);
Counts=merge(Counts,dataS[,c(1,IncludeS+1),with=F],all=T)
Counts=Counts[,EnsemblGeneID:=NULL]
Counts=as.matrix(Counts)
```

Filtering of transcripts (removing the low signal genes)

For the analysis, we only include transcripts with

- More than 20 RPM (on average), across all cells

```
CountsRPM=1000000*Counts/colSums(Counts)

# Filtering of genes
Include = which(rowMeans(CountsRPM)>20)
CountsFilter <- Counts[ Include, ]
Genes.ids=genes.id[Include]
Tech=rep(F,times=dim(CountsFilter)[1])
Tech[grep("ERCC",Genes.ids)]=T
rownames(CountsFilter) <- Genes.ids
```

The input data contains 5687 genes and 182 cells.

BASiCS analysis

The input dataset

Spike-in genes information

```r
# Creating indicator of technical genes
Tech=grepl("ERCC",Genes.ids)
table(Tech)
```

```
## Tech
## FALSE  TRUE
##  5634    53
```

```r
# Input number of molecules for spike-in genes
SpikesInfo=read.table(file.path(data.path,"ERCC_controls.txt"),header=T)
Spikes.ids=Genes.ids[grep("ERCC",Genes.ids)]
SpikesMolecules=as.data.table(SpikesInfo[SpikesInfo$ERCC_ID %in% Spikes.ids,])
SpikesMolecules=SpikesMolecules[order(ERCC_ID),]
SpikesMolecules=as.data.frame(SpikesMolecules)
```

Re-ordering of genes

```r
Counts=rbind(CountsFilter[!Tech,],CountsFilter[Tech,])
Genes.ids = c(Genes.ids[!Tech], Genes.ids[Tech])
Tech=c(Tech[!Tech],Tech[Tech])
```

Separating expression counts for each condition

```r
Counts.G1 <- Counts[, grep("G1_dec", colnames(Counts))]
Counts.S <- Counts[, grep("S_dec", colnames(Counts))]
Counts.G2M <- Counts[, grep("G2M_dec", colnames(Counts))]

Cell.Colour <- c(rep("lightpink3",ncol(Counts.G1)),
                 rep("darkolivegreen3",ncol(Counts.S)),
                 rep("darkgoldenrod1", ncol(Counts.G2M)))

Counts <- cbind(Counts.G1, Counts.S, Counts.G2M)
```

Final processed data contains 59 cells in the G1 group, 58 cells in the S group and 65 cells in the G2M group.

Creating the input object

To use BASiCS, we need to create a `BASiCSDV_Data` object containing the expression counts, a vector of spike-in gene indicators (`TRUE/FALSE`) and the input number of mRNA molecules for each spike-in gene.

```r
Data.G1 = newBASiCS_Data(Counts = Counts.G1,
                         Tech = Tech,
                         SpikeInfo = SpikesMolecules)
```

```
## An object of class BASiCS_Data
##  Dataset contains 5687 genes (5634 biological and 53 technical) and 59 cells.
##  Elements (slots): Counts, Tech, SpikeInput, GeneNames and BatchInfo.
```

```
##  The data contains 1 batch.
##
## NOTICE: BASiCS requires a pre-filtered dataset
##     - You must remove poor quality cells before creating the BASiCS data object
##     - We recommend to pre-filter very lowly expressed transcripts before creating the object.
##       Inclusion criteria may vary for each data. For example, remove transcripts
##           - with very low total counts across of all of the samples
##           - that are only expressed in a few cells
##             (by default genes expressed in only 1 cell are not accepted)
##           - with very low total counts across the samples where the transcript is expressed
##
##  BASiCS_Filter can be used for this purpose
```

```
Data.S = newBASiCS_Data(Counts = Counts.S,
                         Tech = Tech,
                         SpikeInfo = SpikesMolecules)
```

```
## An object of class BASiCS_Data
##  Dataset contains 5687 genes (5634 biological and 53 technical) and 58 cells.
##  Elements (slots): Counts, Tech, SpikeInput, GeneNames and BatchInfo.
##  The data contains 1 batch.
##
## NOTICE: BASiCS requires a pre-filtered dataset
##     - You must remove poor quality cells before creating the BASiCS data object
##     - We recommend to pre-filter very lowly expressed transcripts before creating the object.
##       Inclusion criteria may vary for each data. For example, remove transcripts
##           - with very low total counts across of all of the samples
##           - that are only expressed in a few cells
##             (by default genes expressed in only 1 cell are not accepted)
##           - with very low total counts across the samples where the transcript is expressed
##
##  BASiCS_Filter can be used for this purpose
```

```
Data.G2M = newBASiCS_Data(Counts = Counts.G2M,
                         Tech = Tech,
                         SpikeInfo = SpikesMolecules)
```

```
## An object of class BASiCS_Data
##  Dataset contains 5687 genes (5634 biological and 53 technical) and 65 cells.
##  Elements (slots): Counts, Tech, SpikeInput, GeneNames and BatchInfo.
##  The data contains 1 batch.
##
## NOTICE: BASiCS requires a pre-filtered dataset
##     - You must remove poor quality cells before creating the BASiCS data object
##     - We recommend to pre-filter very lowly expressed transcripts before creating the object.
##       Inclusion criteria may vary for each data. For example, remove transcripts
##           - with very low total counts across of all of the samples
##           - that are only expressed in a few cells
##             (by default genes expressed in only 1 cell are not accepted)
##           - with very low total counts across the samples where the transcript is expressed
##
##  BASiCS_Filter can be used for this purpose
```

Fitting the BASiCS model

To run the MCMC algorithm, we use the function `BASiCS_MCMC`.

```
N = 20000; Thin = 10; Burn = 10000
RunNameG1 = paste0("CellCycle_G1_",N)
RunNameS = paste0("CellCycle_S_",N)
RunNameG2M = paste0("CellCycle_G2M_",N)

MCMC_Output.G1 <- BASiCS_MCMC(Data.G1, N = N, Thin = Thin, Burn = Burn,
                              PrintProgress = TRUE, StoreChains = TRUE,
                              StoreDir = chains.path, RunName = RunNameG1)
MCMC_Output.S <- BASiCS_MCMC(Data.S, N = N, Thin = Thin, Burn = Burn,
                              PrintProgress = TRUE, StoreChains = TRUE,
                              StoreDir = chains.path, RunName = RunNameS)
MCMC_Output.G2M <- BASiCS_MCMC(Data.G2M, N = N, Thin = Thin, Burn = Burn,
                              PrintProgress = TRUE, StoreChains = TRUE,
                              StoreDir = chains.path, RunName = RunNameG2M)
```

Loading pre-computed chains

Loading pre-computed chains for which `a2.mu = 0.5` and `a2.delta = 0.5`.

```
ChainMuG1 = as.matrix(fread(file.path(chains.path, "chain_mu_CellCycle_G1_20000.txt")))
ChainMuS = as.matrix(fread(file.path(chains.path, "chain_mu_CellCycle_S_20000.txt")))
ChainMuG2M = as.matrix(fread(file.path(chains.path, "chain_mu_CellCycle_G2M_20000.txt")))

ChainDeltaG1 = as.matrix(fread(file.path(chains.path, "chain_delta_CellCycle_G1_20000.txt")))
ChainDeltaS = as.matrix(fread(file.path(chains.path, "chain_delta_CellCycle_S_20000.txt")))
ChainDeltaG2M = as.matrix(fread(file.path(chains.path, "chain_delta_CellCycle_G2M_20000.txt")))

ChainPhiG1 = as.matrix(fread(file.path(chains.path, "chain_phi_CellCycle_G1_20000.txt")))
ChainPhiS = as.matrix(fread(file.path(chains.path, "chain_phi_CellCycle_S_20000.txt")))
ChainPhiG2M = as.matrix(fread(file.path(chains.path, "chain_phi_CellCycle_G2M_20000.txt")))

ChainSG1 = as.matrix(fread(file.path(chains.path, "chain_s_CellCycle_G1_20000.txt")))
ChainSS = as.matrix(fread(file.path(chains.path, "chain_s_CellCycle_S_20000.txt")))
ChainSG2M = as.matrix(fread(file.path(chains.path, "chain_s_CellCycle_G2M_20000.txt")))

ChainNuG1 = as.matrix(fread(file.path(chains.path, "chain_nu_CellCycle_G1_20000.txt")))
ChainNuS = as.matrix(fread(file.path(chains.path, "chain_nu_CellCycle_S_20000.txt")))
ChainNuG2M = as.matrix(fread(file.path(chains.path, "chain_nu_CellCycle_G2M_20000.txt")))

ChainThetaG1 = fread(file.path(chains.path, "chain_theta_CellCycle_G1_20000.txt"))$Batch1
ChainThetaS = fread(file.path(chains.path, "chain_theta_CellCycle_S_20000.txt"))$Batch1
ChainThetaG2M = fread(file.path(chains.path, "chain_theta_CellCycle_G2M_20000.txt"))$Batch1

MCMC_Output1 <- newBASiCS_Chain(mu = ChainMuG1,
                               delta = ChainDeltaG1,
                               phi = ChainPhiG1,
                               nu = ChainNuG1,
                               s = ChainSG1,
                               theta = as.matrix(ChainThetaG1))
```

```
## An object of class BASiCS_Chain
##  1000 MCMC samples.
##  Dataset contains 5634 biological genes and 59 cells (1 batch).
##  Elements (slots): mu, delta, phi, s, nu and theta.
```

```
MCMC_Output2 <- newBASiCS_Chain(mu = ChainMuS,
                                delta = ChainDeltaS,
                                phi = ChainPhiS,
                                nu = ChainNuS,
                                s = ChainSS,
                                theta = as.matrix(ChainThetaS))
```

```
## An object of class BASiCS_Chain
##  1000 MCMC samples.
##  Dataset contains 5634 biological genes and 58 cells (1 batch).
##  Elements (slots): mu, delta, phi, s, nu and theta.
```

```
MCMC_Output3 <- newBASiCS_Chain(mu = ChainMuG2M,
                                delta = ChainDeltaG2M,
                                phi = ChainPhiG2M,
                                nu = ChainNuG2M,
                                s = ChainSG2M,
                                theta = as.matrix(ChainThetaG2M))
```

```
## An object of class BASiCS_Chain
##  1000 MCMC samples.
##  Dataset contains 5634 biological genes and 65 cells (1 batch).
##  Elements (slots): mu, delta, phi, s, nu and theta.
```

```
## Not the right thing to do, but helpful for some things
MCMC_Output <- newBASiCS_Chain(mu = cbind(ChainMuG1, ChainMuS, ChainMuG2M),
                                delta = cbind(ChainDeltaG1, ChainDeltaS, ChainDeltaG2M),
                                phi = cbind(ChainPhiG1, ChainPhiS, ChainPhiG2M),
                                nu = cbind(ChainNuG1, ChainNuS, ChainNuG2M),
                                s = cbind(ChainSG1, ChainSS, ChainSG2M),
                                theta = cbind(ChainThetaG1, ChainThetaS, ChainThetaG2M))
```

```
## An object of class BASiCS_Chain
##  1000 MCMC samples.
##  Dataset contains 16902 biological genes and 182 cells (3 batches).
##  Elements (slots): mu, delta, phi, s, nu and theta.
```

Convergence diagnostics

To assess convergence of the chain, the convergence diagnostics provided by the package `coda` can be used. Additionally, a visual inspection is provided by traceplots. First, for some selected parameters.

```
par(mgp = c(5,1,0)); par(mar = c(7,9,4,0.5)); par(mfrow = c(6,3))
par(cex.lab = 2, cex.axis = 1.5)
plot(apply(ChainMuG1,1,median), type = "l", cex.lab = 2,
     ylab = expression(paste("Median of ",mu[i1])), xlab = "Iteration")
```

```r
plot(apply(ChainMuS,1,median), type = "l", cex.lab = 2,
     ylab = expression(paste("Median of ",mu[i2])), xlab = "Iteration")
plot(apply(ChainMuG2M,1,median), type = "l", cex.lab = 2,
     ylab = expression(paste("Median of ",mu[i3])), xlab = "Iteration")

plot(apply(ChainDeltaG1,1,median), type = "l", cex.lab = 2,
     ylab = expression(paste("Median of ",delta[i1])), xlab = "Iteration")
plot(apply(ChainDeltaS,1,median), type = "l", cex.lab = 2,
     ylab = expression(paste("Median of ",delta[i2])), xlab = "Iteration")
plot(apply(ChainDeltaG2M,1,median), type = "l", cex.lab = 2,
     ylab = expression(paste("Median of ",delta[i3])), xlab = "Iteration")

plot(apply(ChainPhiG1,1,median), type = "l", cex.lab = 2,
     ylab = expression(paste("Median of ",phi[j[1]])), xlab = "Iteration")
plot(apply(ChainPhiS,1,median), type = "l", cex.lab = 2,
     ylab = expression(paste("Median of ",phi[j[2]])), xlab = "Iteration")
plot(apply(ChainPhiG2M,1,median), type = "l", cex.lab = 2,
     ylab = expression(paste("Median of ",phi[j[3]])), xlab = "Iteration")

plot(apply(ChainSG1,1,median), type = "l", cex.lab = 2,
     ylab = expression(paste("Median of ",s[j[1]])), xlab = "Iteration")
plot(apply(ChainSS,1,median), type = "l", cex.lab = 2,
     ylab = expression(paste("Median of ",s[j[2]])), xlab = "Iteration")
plot(apply(ChainSG2M,1,median), type = "l", cex.lab = 2,
     ylab = expression(paste("Median of ",s[j[3]])), xlab = "Iteration")

plot(apply(ChainNuG1,1,median), type = "l", cex.lab = 2,
     ylab = expression(paste("Median of ",nu[j[1]])), xlab = "Iteration")
plot(apply(ChainNuS,1,median), type = "l", cex.lab = 2,
     ylab = expression(paste("Median of ",nu[j[2]])), xlab = "Iteration")
plot(apply(ChainNuG2M,1,median), type = "l", cex.lab = 2,
     ylab = expression(paste("Median of ",nu[j[3]])), xlab = "Iteration")

plot(ChainThetaG1, type = "l", cex.lab = 2,
     ylab = expression(theta[1]), xlab = "Iteration")
plot(ChainThetaS, type = "l", cex.lab = 2,
     ylab = expression(theta[2]), xlab = "Iteration")
plot(ChainThetaG2M, type = "l", cex.lab = 2,
     ylab = expression(theta[3]), xlab = "Iteration")
```

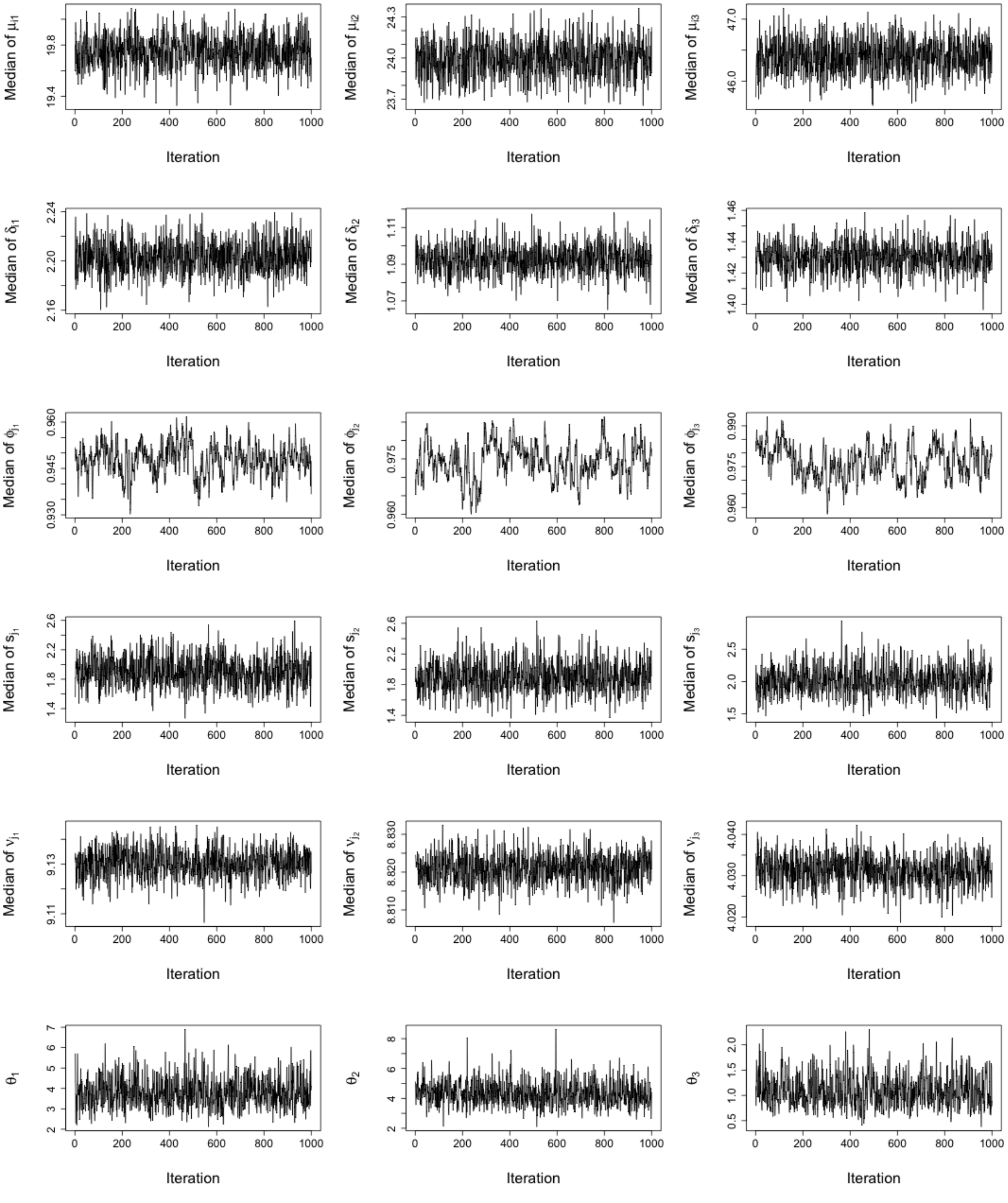

We also provide additional information to assess the convergence of model parameters that are gene-specific, which are the key parameters in our model and define the results of the comparisons between cell types (randomly selected genes only).

Traceplots and autocorrelation plots for overall expression parameters (G1 cells)

```
par(mgp = c(5,1,0)); par(mar = c(7,9,4,0.5)); par(mfrow = c(4,2))
par(cex.lab = 2, cex.axis = 1.5)
genesel = sample(1:ncol(ChainDeltaG1), 1)
plot(ChainMuG1[,genesel], type = "l", main = Data.G1@GeneNames[genesel],
     ylab = expression(paste("Median of ",mu[i1])), xlab = "Iteration")
acf(ChainMuG1[,genesel], main = Data.G1@GeneNames[genesel])
genesel = sample(1:ncol(ChainDeltaG1), 1)
plot(ChainMuG1[,genesel], type = "l", main = Data.G1@GeneNames[genesel],
     ylab = expression(paste("Median of ",mu[i1])), xlab = "Iteration")
acf(ChainMuG1[,genesel], main = Data.G1@GeneNames[genesel])
genesel = sample(1:ncol(ChainDeltaG1), 1)
plot(ChainMuG1[,genesel], type = "l", main = Data.G1@GeneNames[genesel],
     ylab = expression(paste("Median of ",mu[i1])), xlab = "Iteration")
acf(ChainMuG1[,genesel], main = Data.G1@GeneNames[genesel])
genesel = sample(1:ncol(ChainDeltaG1), 1)
plot(ChainMuG1[,genesel], type = "l", main = Data.G1@GeneNames[genesel],
     ylab = expression(paste("Median of ",mu[i1])), xlab = "Iteration")
acf(ChainMuG1[,genesel], main = Data.G1@GeneNames[genesel])
```

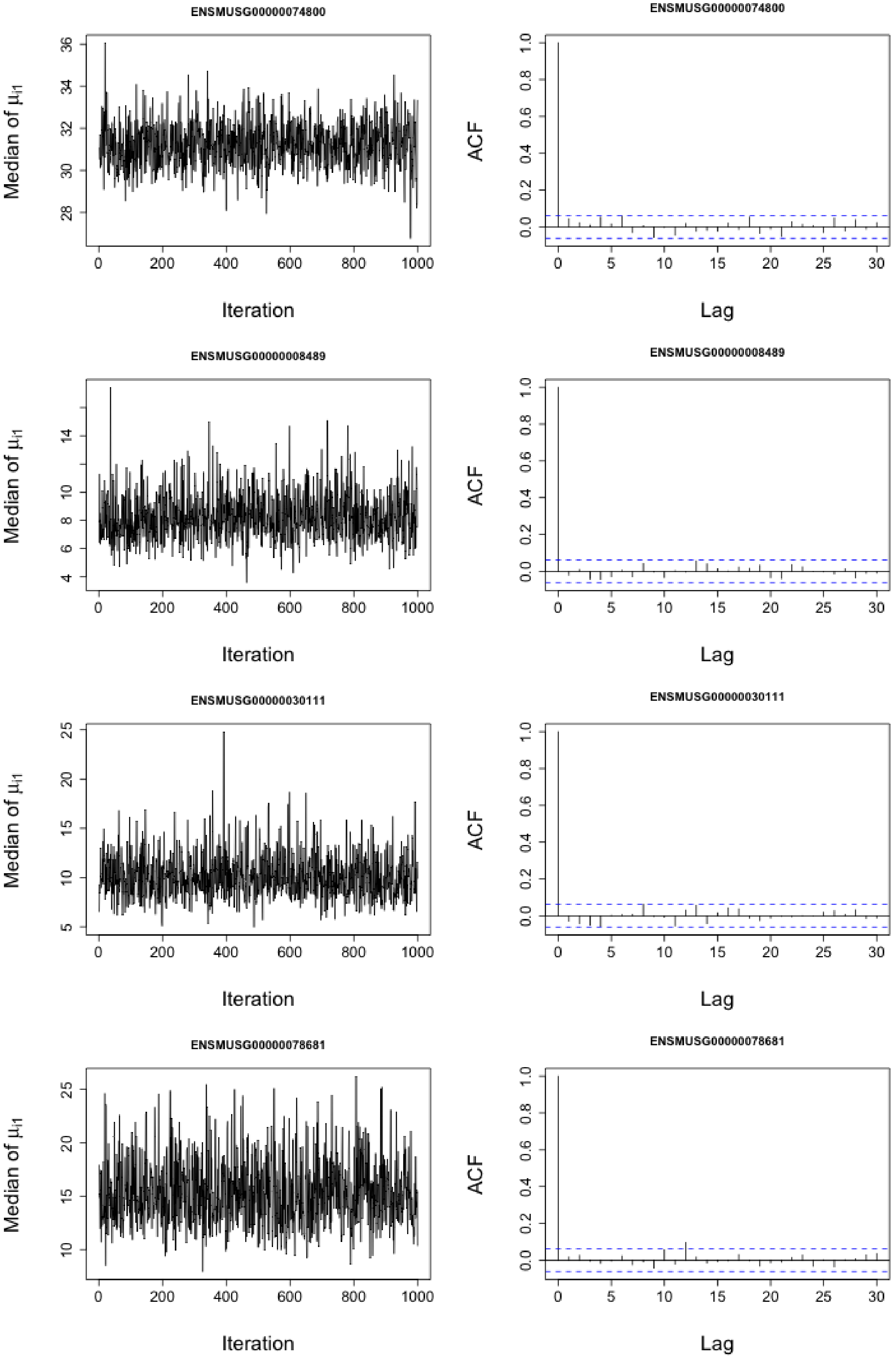
ENSMUSG00000074800
Median of μ_{i1}
Iteration
ENSMUSG00000074800
ACF
Lag
ENSMUSG00000008489
Median of μ_{i1}
Iteration
ENSMUSG00000008489
ACF
Lag
ENSMUSG00000030111
Median of μ_{i1}
Iteration
ENSMUSG00000030111
ACF
Lag
ENSMUSG00000078681
Median of μ_{i1}
Iteration
ENSMUSG00000078681
ACF
Lag

Traceplots and autocorrelation plots for overall expression parameters (S cells)

```
par(mgp = c(5,1,0)); par(mar = c(7,9,4,0.5)); par(mfrow = c(4,2))
par(cex.lab = 2, cex.axis = 1.5)
genesel = sample(1:ncol(ChainDeltaS), 1)
plot(ChainMuS[,genesel], type = "l", main = Data.S@GeneNames[genesel],
     ylab = expression(paste("Median of ",mu[i1])), xlab = "Iteration")
acf(ChainMuS[,genesel], main = Data.S@GeneNames[genesel])
genesel = sample(1:ncol(ChainDeltaS), 1)
plot(ChainMuS[,genesel], type = "l", main = Data.S@GeneNames[genesel],
     ylab = expression(paste("Median of ",mu[i1])), xlab = "Iteration")
acf(ChainMuS[,genesel], main = Data.S@GeneNames[genesel])
genesel = sample(1:ncol(ChainDeltaS), 1)
plot(ChainMuS[,genesel], type = "l", main = Data.S@GeneNames[genesel],
     ylab = expression(paste("Median of ",mu[i1])), xlab = "Iteration")
acf(ChainMuS[,genesel], main = Data.S@GeneNames[genesel])
genesel = sample(1:ncol(ChainDeltaS), 1)
plot(ChainMuS[,genesel], type = "l", main = Data.S@GeneNames[genesel],
     ylab = expression(paste("Median of ",mu[i1])), xlab = "Iteration")
acf(ChainMuS[,genesel], main = Data.S@GeneNames[genesel])
```

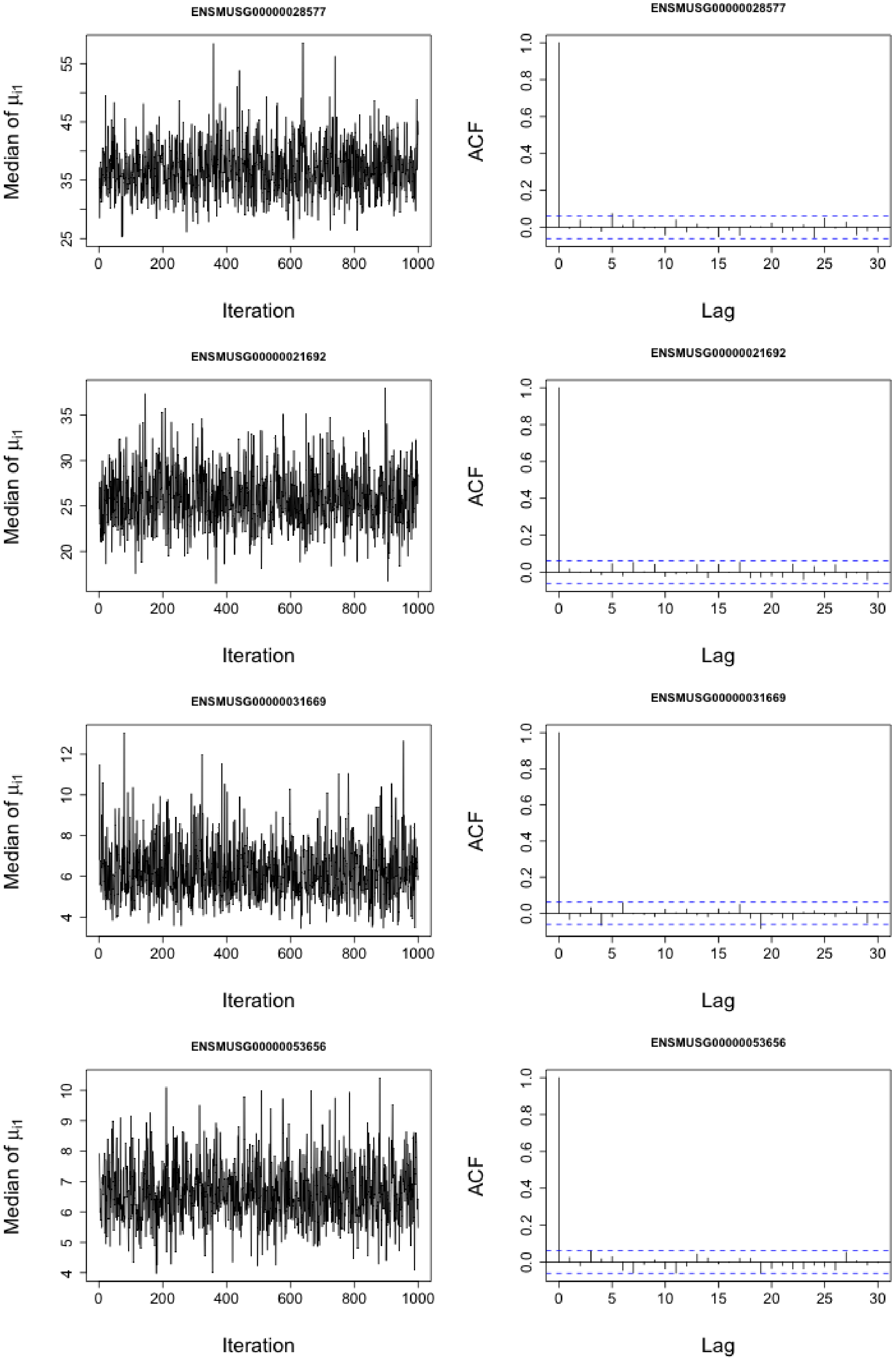
ENSMUSG00000028577
Median of μ_{i1}
Iteration
ACF
Lag
ENSMUSG00000021692
Median of μ_{i1}
Iteration
ACF
Lag
ENSMUSG00000031669
Median of μ_{i1}
Iteration
ACF
Lag
ENSMUSG00000053656
Median of μ_{i1}
Iteration
ACF
Lag

Traceplots and autocorrelation plots for overall expression parameters (G1 cells)

```
par(mgp = c(5,1,0)); par(mar = c(7,9,4,0.5)); par(mfrow = c(4,2))
par(cex.lab = 2, cex.axis = 1.5)
genesel = sample(1:ncol(ChainDeltaG2M), 1)
plot(ChainMuG2M[,genesel], type = "l", main = Data.G2M@GeneNames[genesel],
     ylab = expression(paste("Median of ",mu[i1])), xlab = "Iteration")
acf(ChainMuG2M[,genesel], main = Data.G2M@GeneNames[genesel])
genesel = sample(1:ncol(ChainDeltaG2M), 1)
plot(ChainMuG2M[,genesel], type = "l", main = Data.G2M@GeneNames[genesel],
     ylab = expression(paste("Median of ",mu[i1])), xlab = "Iteration")
acf(ChainMuG2M[,genesel], main = Data.G2M@GeneNames[genesel])
genesel = sample(1:ncol(ChainDeltaG2M), 1)
plot(ChainMuG2M[,genesel], type = "l", main = Data.G2M@GeneNames[genesel],
     ylab = expression(paste("Median of ",mu[i1])), xlab = "Iteration")
acf(ChainMuG2M[,genesel], main = Data.G2M@GeneNames[genesel])
genesel = sample(1:ncol(ChainDeltaG2M), 1)
plot(ChainMuG2M[,genesel], type = "l", main = Data.G2M@GeneNames[genesel],
     ylab = expression(paste("Median of ",mu[i1])), xlab = "Iteration")
acf(ChainMuG2M[,genesel], main = Data.G2M@GeneNames[genesel])
```

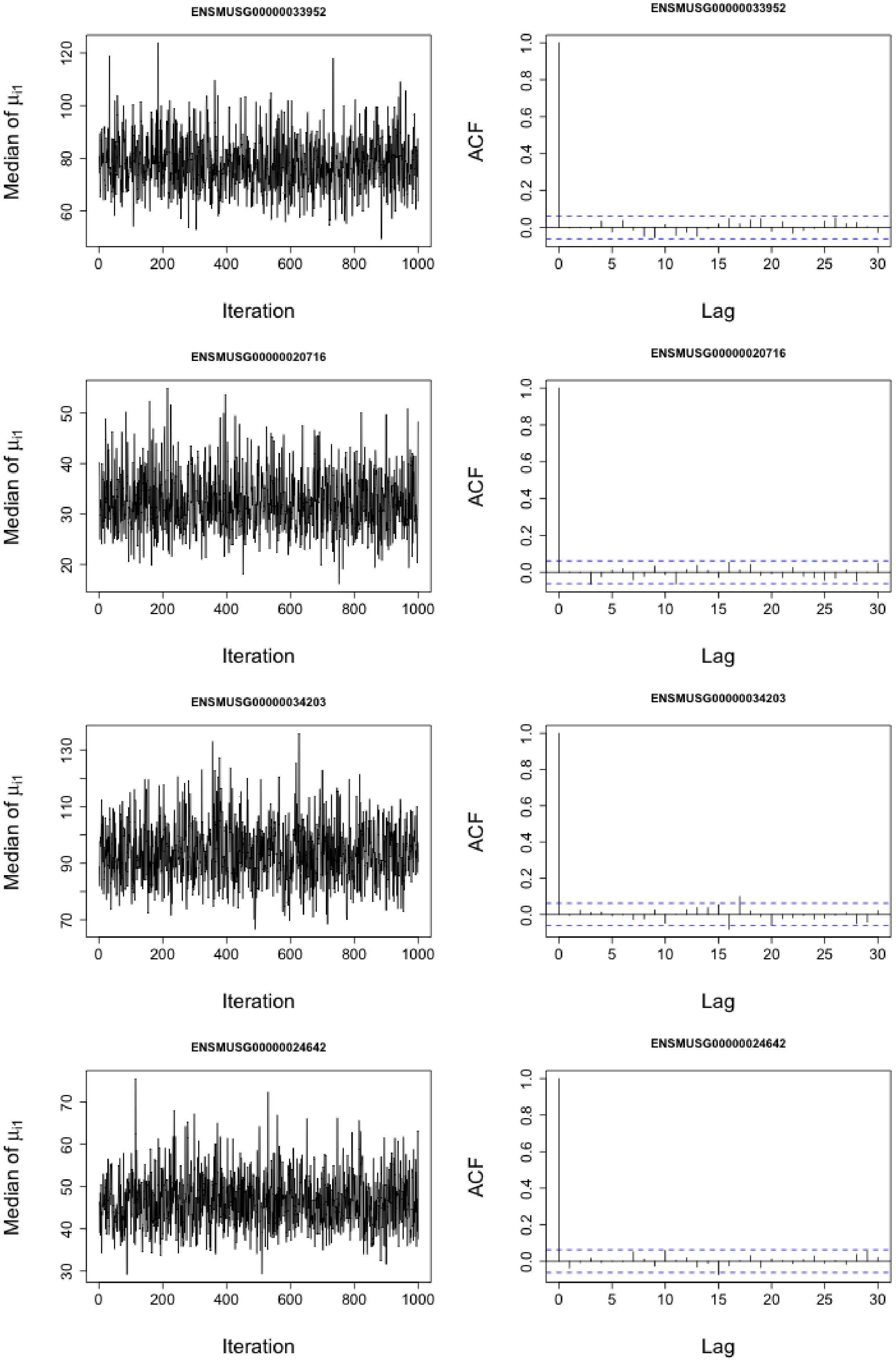
ENSMUSG00000033952
Median of μ_{i1}
Iteration
ENSMUSG00000033952
ACF
Lag
ENSMUSG00000020716
Median of μ_{i1}
Iteration
ENSMUSG00000020716
ACF
Lag
ENSMUSG00000034203
Median of μ_{i1}
Iteration
ENSMUSG00000034203
ACF
Lag
ENSMUSG00000024642
Median of μ_{i1}
Iteration
ENSMUSG00000024642
ACF
Lag

Traceplots and autocorrelation plots for dispersion parameters (G1 cells)

```
par(mgp = c(5,1,0)); par(mar = c(7,9,4,0.5)); par(mfrow = c(4,2))
par(cex.lab = 2, cex.axis = 1.5)
genesel = sample(1:ncol(ChainDeltaG1), 1)
plot(ChainDeltaG1[,genesel], type = "l", main = Data.G1@GeneNames[genesel],
     ylab = expression(paste("Median of ",delta[i1])), xlab = "Iteration")
acf(ChainDeltaG1[,genesel], main = Data.G1@GeneNames[genesel])
genesel = sample(1:ncol(ChainDeltaG1), 1)
plot(ChainDeltaG1[,genesel], type = "l", main = Data.G1@GeneNames[genesel],
     ylab = expression(paste("Median of ",delta[i1])), xlab = "Iteration")
acf(ChainDeltaG1[,genesel], main = Data.G1@GeneNames[genesel])
genesel = sample(1:ncol(ChainDeltaG1), 1)
plot(ChainDeltaG1[,genesel], type = "l", main = Data.G1@GeneNames[genesel],
     ylab = expression(paste("Median of ",delta[i1])), xlab = "Iteration")
acf(ChainDeltaG1[,genesel], main = Data.G1@GeneNames[genesel])
genesel = sample(1:ncol(ChainDeltaG1), 1)
plot(ChainDeltaG1[,genesel], type = "l", main = Data.G1@GeneNames[genesel],
     ylab = expression(paste("Median of ",delta[i1])), xlab = "Iteration")
acf(ChainDeltaG1[,genesel], main = Data.G1@GeneNames[genesel])
```

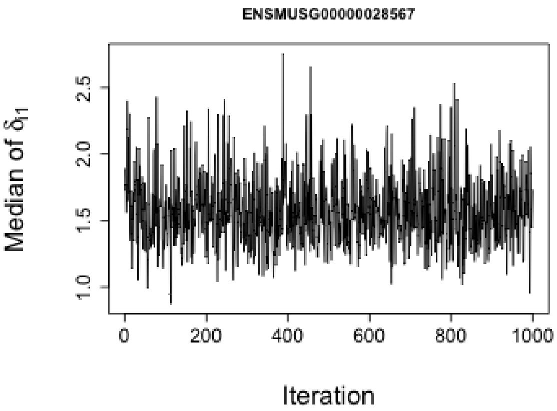
ENSMUSG00000028567
Median of δ_{11}
Iteration

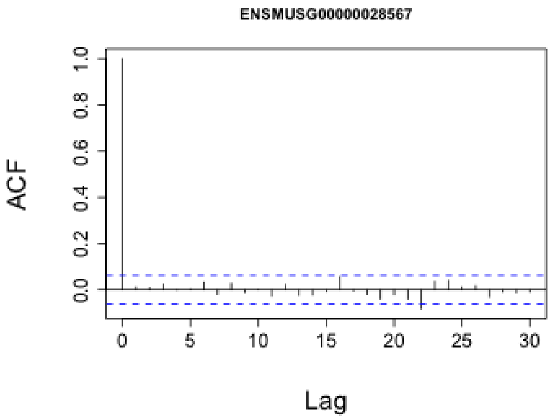
ENSMUSG00000028567
ACF
Lag

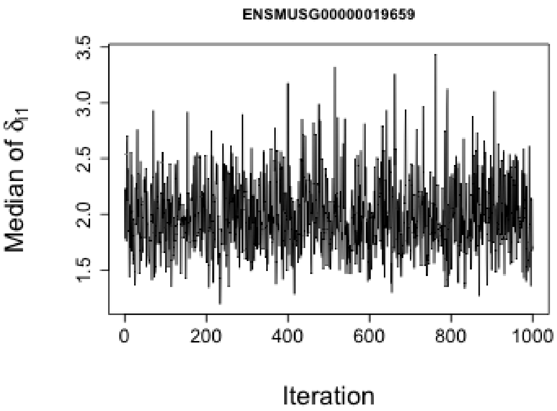
ENSMUSG00000019659
Median of δ_{11}
Iteration

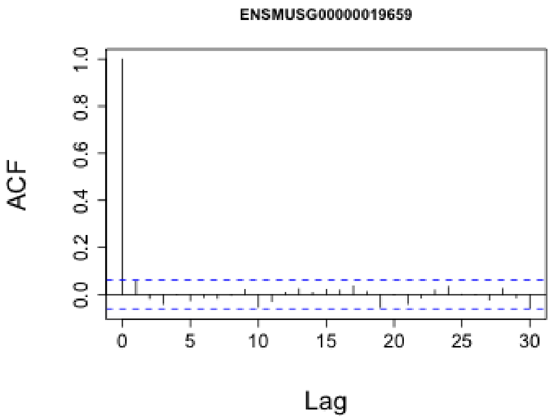
ENSMUSG00000019659
ACF
Lag

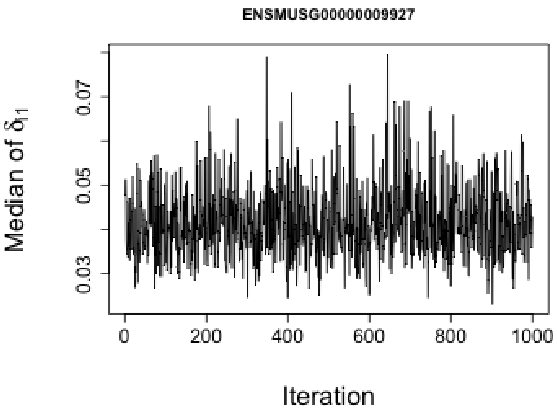
ENSMUSG00000009927
Median of δ_{11}
Iteration

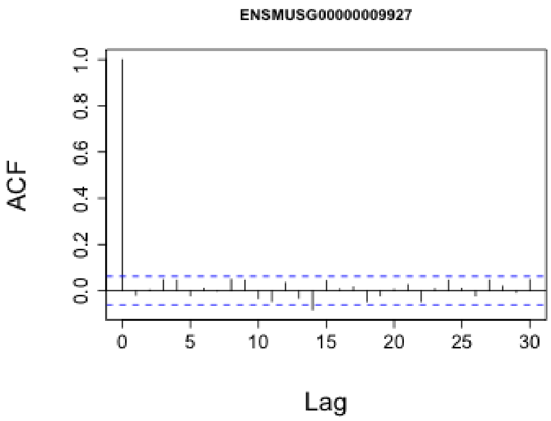
ENSMUSG00000009927
ACF
Lag

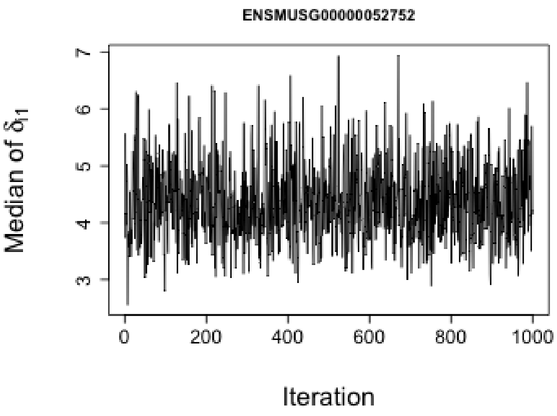
ENSMUSG00000052752
Median of δ_{11}
Iteration

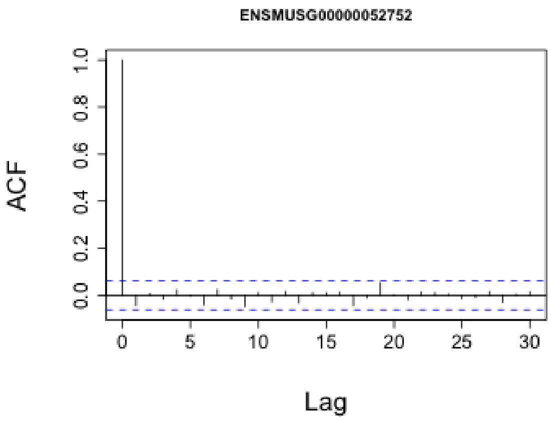
ENSMUSG00000052752
ACF
Lag

Traceplots and autocorrelation plots for dispersion parameters (S cells)

```
par(mgp = c(5,1,0)); par(mar = c(7,9,4,0.5)); par(mfrow = c(4,2))
par(cex.lab = 2, cex.axis = 1.5)
genesel = sample(1:ncol(ChainDeltaS), 1)
plot(ChainDeltaS[,genesel], type = "l", main = Data.S@GeneNames[genesel],
     ylab = expression(paste("Median of ",delta[i1])), xlab = "Iteration")
acf(ChainDeltaS[,genesel], main = Data.S@GeneNames[genesel])
genesel = sample(1:ncol(ChainDeltaS), 1)
plot(ChainDeltaS[,genesel], type = "l", main = Data.S@GeneNames[genesel],
     ylab = expression(paste("Median of ",delta[i1])), xlab = "Iteration")
acf(ChainDeltaS[,genesel], main = Data.S@GeneNames[genesel])
genesel = sample(1:ncol(ChainDeltaS), 1)
plot(ChainDeltaS[,genesel], type = "l", main = Data.S@GeneNames[genesel],
     ylab = expression(paste("Median of ",delta[i1])), xlab = "Iteration")
acf(ChainDeltaS[,genesel], main = Data.S@GeneNames[genesel])
genesel = sample(1:ncol(ChainDeltaS), 1)
plot(ChainDeltaS[,genesel], type = "l", main = Data.S@GeneNames[genesel],
     ylab = expression(paste("Median of ",delta[i1])), xlab = "Iteration")
acf(ChainDeltaS[,genesel], main = Data.S@GeneNames[genesel])
```

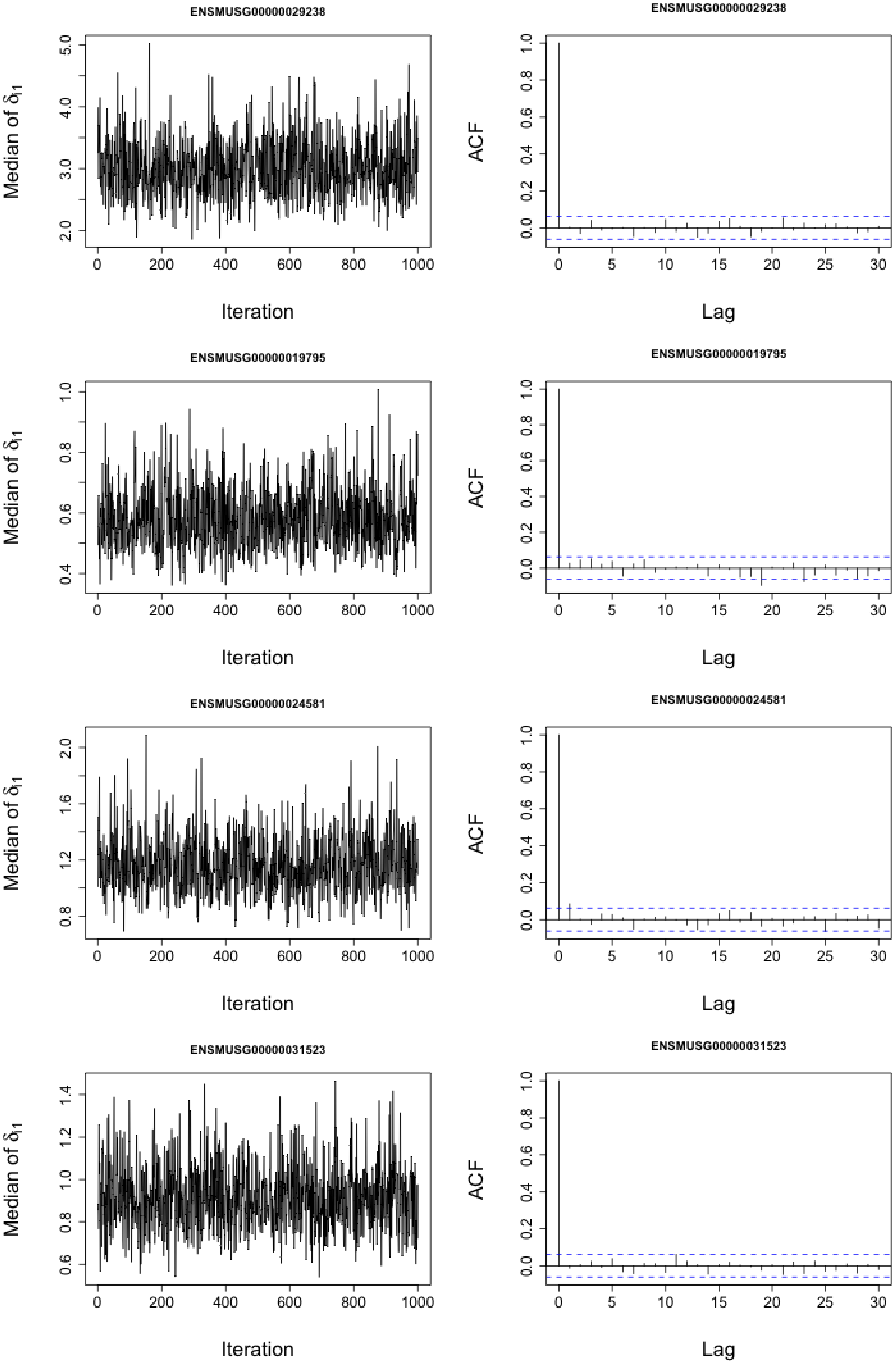
ENSMUSG00000029238
Median of δ11
Iteration
ENSMUSG00000029238
ACF
Lag
ENSMUSG00000019795
Median of δ11
Iteration
ENSMUSG00000019795
ACF
Lag
ENSMUSG00000024581
Median of δ11
Iteration
ENSMUSG00000024581
ACF
Lag
ENSMUSG00000031523
Median of δ11
Iteration
ENSMUSG00000031523
ACF
Lag

Traceplots and autocorrelation plots for dispersion parameters (G1 cells)

```
par(mgp = c(5,1,0)); par(mar = c(7,9,4,0.5)); par(mfrow = c(4,2))
par(cex.lab = 2, cex.axis = 1.5)
genesel = sample(1:ncol(ChainDeltaG2M), 1)
plot(ChainDeltaG2M[,genesel], type = "l", main = Data.G2M@GeneNames[genesel],
     ylab = expression(paste("Median of ",delta[i1])), xlab = "Iteration")
acf(ChainDeltaG2M[,genesel], main = Data.G2M@GeneNames[genesel])
genesel = sample(1:ncol(ChainDeltaG2M), 1)
plot(ChainDeltaG2M[,genesel], type = "l", main = Data.G2M@GeneNames[genesel],
     ylab = expression(paste("Median of ",delta[i1])), xlab = "Iteration")
acf(ChainDeltaG2M[,genesel], main = Data.G2M@GeneNames[genesel])
genesel = sample(1:ncol(ChainDeltaG2M), 1)
plot(ChainDeltaG2M[,genesel], type = "l", main = Data.G2M@GeneNames[genesel],
     ylab = expression(paste("Median of ",delta[i1])), xlab = "Iteration")
acf(ChainDeltaG2M[,genesel], main = Data.G2M@GeneNames[genesel])
genesel = sample(1:ncol(ChainDeltaG2M), 1)
plot(ChainDeltaG2M[,genesel], type = "l", main = Data.G2M@GeneNames[genesel],
     ylab = expression(paste("Median of ",delta[i1])), xlab = "Iteration")
acf(ChainDeltaG2M[,genesel], main = Data.G2M@GeneNames[genesel])
```

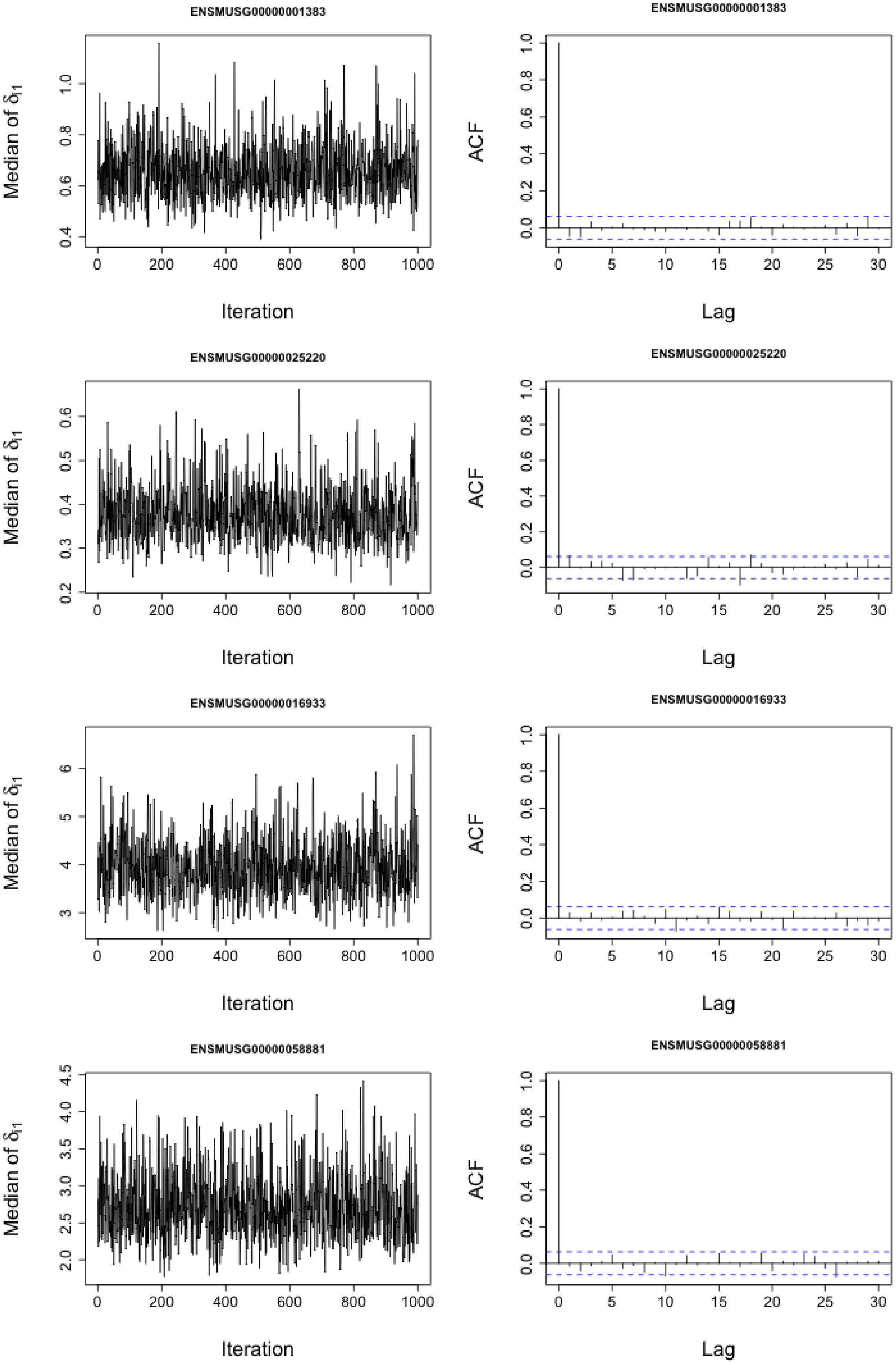
ENSMUSG00000001383
Median of δ_{11}
Iteration
ENSMUSG00000001383
ACF
Lag
ENSMUSG00000025220
Median of δ_{11}
Iteration
ENSMUSG00000025220
ACF
Lag
ENSMUSG00000016933
Median of δ_{11}
Iteration
ENSMUSG00000016933
ACF
Lag
ENSMUSG00000058881
Median of δ_{11}
Iteration
ENSMUSG00000058881
ACF
Lag

Additionally, we run the Geweke convergence diagnostic (see library `coda` for gene-specific parameters). As it can be seen bellow, most of the associated Z scores are small, with just a few cases lying outside the (-2,2) interval (not surprisingly due to the large number of parameters). For the genes with extreme Z score values, we also provide traceplots to illustrate that the evidence againts convergence is very weak (if any).

```
library(coda)

ChainMuG1MCMC = mcmc(ChainMuG1)
ChainMuSMCMC = mcmc(ChainMuS)
ChainMuG2MMCMC = mcmc(ChainMuG2M)
ChainDeltaG1MCMC = mcmc(ChainDeltaG1)
ChainDeltaSMCMC = mcmc(ChainDeltaS)
ChainDeltaG2MMCMC = mcmc(ChainDeltaG2M)

ChainMuG1.geweke = geweke.diag(ChainMuG1MCMC)
ChainMuS.geweke = geweke.diag(ChainMuSMCMC)
ChainMuG2M.geweke = geweke.diag(ChainMuG2MMCMC)
ChainDeltaG1.geweke = geweke.diag(ChainDeltaG1MCMC)
ChainDeltaS.geweke = geweke.diag(ChainDeltaSMCMC)
ChainDeltaG2M.geweke = geweke.diag(ChainDeltaG2MMCMC)

summary(cbind(ChainMuG1.geweke$z, ChainMuS.geweke$z, ChainMuG2M.geweke$z,
              ChainDeltaG1.geweke$z, ChainDeltaS.geweke$z, ChainDeltaG2M.geweke$z))
```

```
##        V1                   V2                  V3
##  Min.   :-4.405887   Min.   :-4.05001   Min.   :-4.69788
##  1st Qu.:-0.702119   1st Qu.:-0.71170   1st Qu.:-0.72711
##  Median :-0.004102   Median :-0.02837   Median :-0.01154
##  Mean   :-0.021931   Mean   :-0.03645   Mean   :-0.02674
##  3rd Qu.: 0.693513   3rd Qu.: 0.65325   3rd Qu.: 0.65515
##  Max.   : 4.629556   Max.   : 4.57364   Max.   : 4.65154
##        V4                   V5                  V6
##  Min.   :-4.374696   Min.   :-4.18620   Min.   :-5.431570
##  1st Qu.:-0.712819   1st Qu.:-0.71078   1st Qu.:-0.680263
##  Median : 0.004688   Median :-0.03536   Median : 0.005217
##  Mean   :-0.016182   Mean   :-0.03227   Mean   :-0.006212
##  3rd Qu.: 0.674419   3rd Qu.: 0.67720   3rd Qu.: 0.689929
##  Max.   : 4.500239   Max.   : 5.34821   Max.   : 3.728605
```

```
par(mgp = c(5,1,0)); par(mar = c(7,9,4,0.5)); par(mfrow = c(2,3))
par(cex.lab = 2, cex.axis = 1.5)
genesel = which(abs(ChainMuG1.geweke$z) ==  max(abs(ChainMuG1.geweke$z)) )
plot(ChainMuG1[,genesel], type = "l", main = Data.G1@GeneNames[genesel],
     ylab = expression(paste("Median of ",mu[i1])), xlab = "Iteration")
genesel = which(abs(ChainMuS.geweke$z) ==  max(abs(ChainMuS.geweke$z)) )
plot(ChainMuS[,genesel], type = "l", main = Data.S@GeneNames[genesel],
     ylab = expression(paste("Median of ",mu[i1])), xlab = "Iteration")
genesel = which(abs(ChainMuG2M.geweke$z) ==  max(abs(ChainMuG2M.geweke$z)) )
plot(ChainMuG2M[,genesel], type = "l", main = Data.G2M@GeneNames[genesel],
     ylab = expression(paste("Median of ",mu[i1])), xlab = "Iteration")

genesel = which(abs(ChainDeltaG1.geweke$z) ==  max(abs(ChainDeltaG1.geweke$z)) )
plot(ChainDeltaG1[,genesel], type = "l", main = Data.G1@GeneNames[genesel],
```

```
     ylab = expression(paste("Median of ",delta[i1])), xlab = "Iteration")
genesel = which(abs(ChainDeltaS.geweke$z) ==  max(abs(ChainDeltaS.geweke$z)) )
plot(ChainDeltaS[,genesel], type = "l", main = Data.S@GeneNames[genesel],
     ylab = expression(paste("Median of ",delta[i1])), xlab = "Iteration")
genesel = which(abs(ChainDeltaG2M.geweke$z) ==  max(abs(ChainDeltaG2M.geweke$z)) )
plot(ChainDeltaG2M[,genesel], type = "l", main = Data.G2M@GeneNames[genesel],
     ylab = expression(paste("Median of ",delta[i1])), xlab = "Iteration")
```

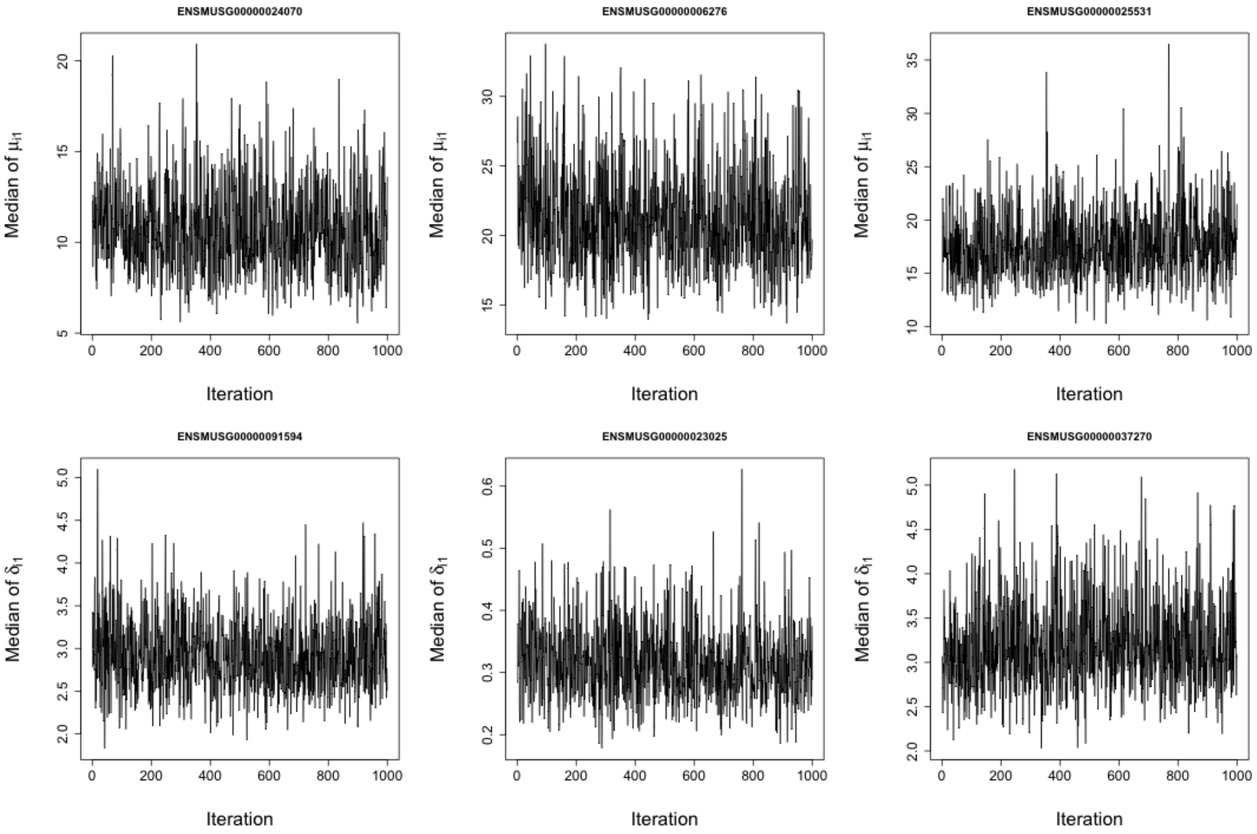

Finally, we also run additional MCMC chains with different starting values. All chains led to virtually the same results. Hence, we conclude we have strong evidence to support that the chain has reached its stationary distribution (not shown).

Offset effect removal

```
OffSetCorrection <- function(Chain1, Chain2, Chain3)
{
  ModelOffSet21 = median(rowSums(Chain2@mu)/rowSums(Chain1@mu))
  ModelOffSet31 = median(rowSums(Chain3@mu)/rowSums(Chain1@mu))
  cat(paste("Model offset 2 vs 1:", ModelOffSet21, "\n"))
  cat(paste("Model offset 3 vs 1:", ModelOffSet31, "\n"))

  list("OffSet21" = ModelOffSet21,
       "OffSet31" = ModelOffSet31)
}
```

```
OffSetComparison <- function(Data1, Data2, Data3,
                             Chain1, Chain2, Chain3)
{

  # Posterior medians without correction
  MedianMu1 = apply(Chain1@mu, 2, median)
  MedianMu2 = apply(Chain2@mu, 2, median)
  MedianMu3 = apply(Chain3@mu, 2, median)

  MedianDelta1 = apply(Chain1@delta, 2, median)
  MedianDelta2 = apply(Chain2@delta, 2, median)
  MedianDelta3 = apply(Chain3@delta, 2, median)

  n1 = ncol(Chain1@phi); n2 = ncol(Chain2@phi); n3 = ncol(Chain3@phi)
  n = n1+n2+n3

  MuBase = (MedianMu1 * n1 + MedianMu2 * n2 + MedianMu3 * n3)/n
  DeltaBase = (MedianDelta1 * n1 + MedianDelta2 * n2 + MedianDelta3 * n3)/n

  # Log-fold change chains and estimates
  ChainTau12 = log(Chain1@mu / Chain2@mu)
  ChainTau13 = log(Chain1@mu / Chain3@mu)
  ChainTau23 = log(Chain2@mu / Chain3@mu)
  ChainOmega12 = log(Chain1@delta / Chain2@delta)
  ChainOmega13 = log(Chain1@delta / Chain3@delta)
  ChainOmega23 = log(Chain2@delta / Chain3@delta)

  MedianTau12 = apply(ChainTau12, 2, median)
  MedianTau13 = apply(ChainTau13, 2, median)
  MedianTau23 = apply(ChainTau23, 2, median)
  MedianOmega12 = apply(ChainOmega12, 2, median)
  MedianOmega13 = apply(ChainOmega13, 2, median)
  MedianOmega23 = apply(ChainOmega23, 2, median)

  ModelOffSet21 = median(rowSums(Chain2@mu)/rowSums(Chain1@mu))
  ModelOffSet31 = median(rowSums(Chain3@mu)/rowSums(Chain1@mu))

  par(mfrow = c(2,3))
  par(cex.lab = 3, cex.main = 3, cex.axis = 3)
  par(mar = c(7, 10, 4, 2) + 0.1)
  par(mgp=c(5,2,0))
  par(lwd = 4)


  plot(MuBase, MedianTau12, pch = 16, col = rgb(190,190,190,50,maxColorValue=255),
       bty = "n", log = "x",
     xlab = "Expression rate", cex.lab = 2,
     ylab = expression(paste("LFC in overall expression (",log(hat(mu)[i]^{(G1)}/hat(mu)[i]^{(S)}), ")"
     main = "G1 vs S")
  abline(h = c(0, -log(ModelOffSet21)), lty = c(1,2),
         col = c("black", "blue"))
  legend('bottomright', c("Offset"),
         col = c("blue"), lty = 2, cex = 3, bty = "n")
```

```
  plot(MuBase, MedianTau13, pch = 16, col = rgb(190,190,190,50,maxColorValue=255),
       bty = "n", log = "x",
     xlab = "Expression rate", cex.lab = 2,
     ylab = expression(paste("LFC in overall expression (",log(hat(mu)[i]^{(G1)}/hat(mu)[i]^{(G2M)}), "
     main = "G1 vs G2M")
  abline(h = c(0, -log(ModelOffSet31)), lty = 1:2,
         col = c("black", "blue"))

  plot(MuBase, MedianTau23, pch = 16, col = rgb(190,190,190,50,maxColorValue=255),
       bty = "n", log = "x",
     xlab = "Expression rate", cex.lab = 2,
     ylab = expression(paste("LFC in overall expression (",log(hat(mu)[i]^{(S)}/hat(mu)[i]^{(G2M)}), ")
     main = "S vs G2M")
  abline(h = c(0, -log(ModelOffSet31) + log(ModelOffSet21)), lty = 1:2,
         col = c("black", "blue"))

  Group <- c(rep(1, times = ncol(Data1@Counts)),
             rep(2, times = ncol(Data2@Counts)),
             rep(3, times = ncol(Data3@Counts)))

  MedianPhi1 <- apply(Chain1@phi, 2, median)
  MedianPhi2 <- apply(Chain2@phi, 2, median)
  MedianPhi3 <- apply(Chain3@phi, 2, median)

  boxplot(c(MedianPhi1, MedianPhi2, MedianPhi3) ~ Group,
        names = c("G1", "S", "G2M"),
        col = unique(Cell.Colour),
        ylab = expression(paste("mRNA content (", hat(phi)[j]^{(p)},")")),
        main = "Before offset correction",
        frame = FALSE)

  boxplot(c(MedianPhi1, MedianPhi2 * ModelOffSet21,
            MedianPhi3 * ModelOffSet31) ~ Group,
        names = c("G1", "S", "G2M"),
        col = unique(Cell.Colour),
        ylab = expression(paste("mRNA content (", hat(phi)[j]^{(p)},")")),
        main = "After offset correction",
        frame = FALSE)

#  plot(c(0, 1), c(0, 1), ann = F, bty = 'n', type = 'n',
#       xaxt = 'n', yaxt = 'n')
#  text(x = 0.5, y = 0.5, paste("Offset S vs G1:", round(ModelOffSet21, 2),
#                               ".\n",
#                               "Offset G2M vs G1:", round(ModelOffSet31, 2),
#                               ".\n",
#                               "Offset G2M vs S:", round(ModelOffSet31/ModelOffSet21, 2),
#                               ".\n"),
#       cex = 2.7, col = "black")
}
```

```
OffSet = OffSetCorrection(MCMC_Output1, MCMC_Output2, MCMC_Output3)
```

```
## Model offset 2 vs 1: 1.13830480959853
```

```
## Model offset 3 vs 1: 2.33066673306469
```

```
OffSetComparison(Data.G1, Data.S, Data.G2M,
                 MCMC_Output1, MCMC_Output2, MCMC_Output3)
```

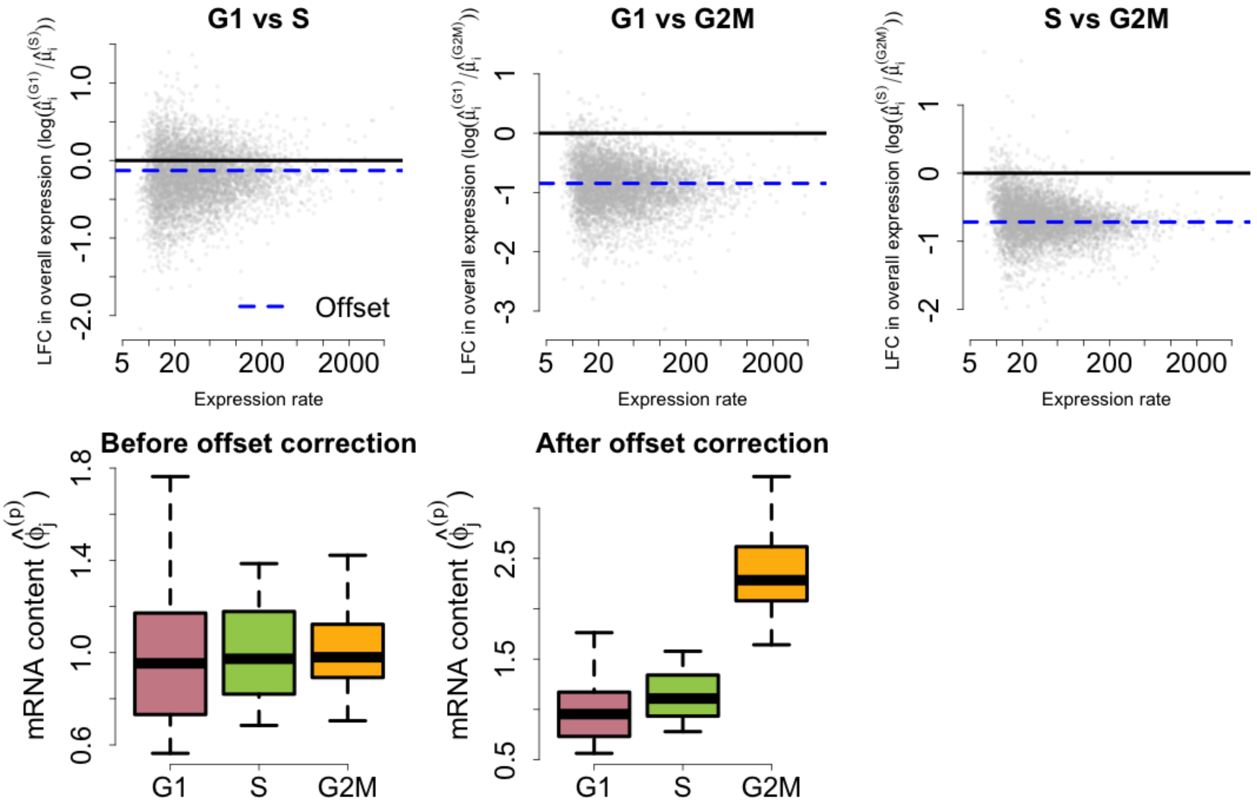


```
MCMC_Output1_1 <- newBASiCS_Chain(mu = ChainMuG1,
                                  delta = ChainDeltaG1,
                                  phi = ChainPhiG1,
                                  nu = ChainNuG1,
                                  s = ChainSG1,
                                  theta = as.matrix(ChainThetaG1))
```

```
## An object of class BASiCS_Chain
##  1000 MCMC samples.
##  Dataset contains 5634 biological genes and 59 cells (1 batch).
##  Elements (slots): mu, delta, phi, s, nu and theta.
```

```
MCMC_Output2_1 <- newBASiCS_Chain(mu = ChainMuS / OffSet$OffSet21,
                                   delta = ChainDeltaS,
                                   phi = ChainPhiS * OffSet$OffSet21,
                                   nu = ChainNuS,
                                   s = ChainSS,
                                   theta = as.matrix(ChainThetaS))
```

```
## An object of class BASiCS_Chain
##  1000 MCMC samples.
##  Dataset contains 5634 biological genes and 58 cells (1 batch).
##  Elements (slots): mu, delta, phi, s, nu and theta.
```

```
MCMC_Output3_1 <- newBASiCS_Chain(mu = ChainMuG2M / OffSet$OffSet31,
                                  delta = ChainDeltaG2M,
                                  phi = ChainPhiG2M * OffSet$OffSet31,
                                  nu = ChainNuG2M,
                                  s = ChainSG2M,
                                  theta = as.matrix(ChainThetaG2M))
```

```
## An object of class BASiCS_Chain
##  1000 MCMC samples.
##  Dataset contains 5634 biological genes and 65 cells (1 batch).
##  Elements (slots): mu, delta, phi, s, nu and theta.
```

```
MCMC_Output_1 <- newBASiCS_Chain(mu = cbind(ChainMuG1, ChainMuS / OffSet$OffSet21,
                                            ChainMuG2M / OffSet$OffSet31),
                               delta = cbind(ChainDeltaG1, ChainDeltaS, ChainDeltaG2M),
                               phi = cbind(ChainPhiG1, ChainPhiS * OffSet$OffSet21,
                                           ChainPhiG2M * OffSet$OffSet31),
                               nu = cbind(ChainNuG1, ChainNuS, ChainNuG2M),
                               s = cbind(ChainSG1, ChainSS, ChainSG2M),
                               theta = cbind(ChainThetaG1, ChainThetaS, ChainThetaG2M))
```

```
## An object of class BASiCS_Chain
##  1000 MCMC samples.
##  Dataset contains 16902 biological genes and 182 cells (3 batches).
##  Elements (slots): mu, delta, phi, s, nu and theta.
```

Summarizing the model fit To summarize the results, the function `Summary` calculates posterior medians and the High Posterior Density (HPD) intervals for each model parameter. As a default option, HPD intervals contain 0.95 probability.

```
MCMC_Summary <- Summary(MCMC_Output_1)

MCMC_Summary1 <- Summary(MCMC_Output1_1)
MCMC_Summary2 <- Summary(MCMC_Output2_1)
MCMC_Summary3 <- Summary(MCMC_Output3_1)
```

To display posterior medians of δ_i (the parameters controlling the strength of the biological cell-to-cell expression heterogeneity of a gene i across the population of cells under study) againts overall gene-specific expression levels μ_i use:

```
par(mfrow = c(3,3))
par(mar = c(7, 10, 4, 4) + 0.1, oma=c(0,0,3,0))
par(mgp = c(5, 2, 0))
par(cex.lab = 2.5, cex.axis = 2)

lim1 = c(min(apply(MCMC_Output1_1@mu, 2, median),
             apply(MCMC_Output2_1@mu, 2, median),
             apply(MCMC_Output3_1@mu, 2, median)),
         max(apply(MCMC_Output1_1@mu, 2, median),
             apply(MCMC_Output2_1@mu, 2, median),
             apply(MCMC_Output3_1@mu, 2, median)))
```

```
lim2 = c(min(apply(MCMC_Output1_1@delta, 2, median),
              apply(MCMC_Output2_1@delta, 2, median),
              apply(MCMC_Output3_1@delta, 2, median)),
          max(apply(MCMC_Output1_1@delta, 2, median),
              apply(MCMC_Output2_1@delta, 2, median),
              apply(MCMC_Output3_1@delta, 2, median)))

plot(c(0, 1), c(0, 1), ann = F, bty = 'n', type = 'n',
       xaxt = 'n', yaxt = 'n', bg = unique(Cell.Colour)[1])
rect(0, 0, 1, 1, col = unique(Cell.Colour)[1])
text(x = 0.5, y = 0.5, paste("G1"), cex = 10, col = "white", lwd = 2)

plot(apply(MCMC_Output2_1@mu, 2, median),
     apply(MCMC_Output1_1@mu, 2, median), pch = 16, col = rgb(190,190,190,50,maxColorValue=255),
     bty = "n", log = "xy",
     xlab = expression(paste("Overall expression S cells (",hat(mu)[i]^{(S)},")")),
     ylab = expression(paste("Overall expression G1 cells (",hat(mu)[i]^{(G1)},")")),
     xlim = lim1, ylim = lim1, cex = 2)
abline(a = 0, b = 1, lty = 2, lwd = 3)

aux = sum(MCMC_Summary2@mu[,1] < MCMC_Summary1@mu[,1]) / length(MCMC_Summary2@mu[,1])

text(x = lim1[1]+5, y = lim1[2], paste0(round(100*aux,1),"%"), cex = 3, col = "blue")
text(x = lim1[2]-5000, y = lim1[1]+1, paste0(100-round(100*aux,1),"%"), cex = 3, col = "blue")

plot(apply(MCMC_Output3_1@mu, 2, median),
     apply(MCMC_Output1_1@mu, 2, median), pch = 16, col = rgb(190,190,190,50,maxColorValue=255),
     bty = "n", log = "xy",
     xlab = expression(paste("Overall expression G2M cells (",hat(mu)[i]^{(G2M)},")")),
     ylab = expression(paste("Overall expression G1 cells (",hat(mu)[i]^{(G1)},")")),
     xlim = lim1, ylim = lim1, cex = 2)
abline(a = 0, b = 1, lty = 2, lwd = 3)

aux = sum(apply(MCMC_Output3_1@mu, 2, median) <
                      apply(MCMC_Output1_1@mu, 2, median)) /
              length(apply(MCMC_Output1_1@mu, 2, median))

text(x = lim1[1]+5, y = lim1[2], paste0(round(100*aux,1),"%"), cex = 3, col = "blue")
text(x = lim1[2]-5000, y = lim1[1]+1, paste0(100-round(100*aux,1),"%"), cex = 3, col = "blue")


plot(apply(MCMC_Output1_1@delta, 2, median),
     apply(MCMC_Output2_1@delta, 2, median), pch = 16, col = rgb(190,190,190,50,maxColorValue=255),
     bty = "n", log = "xy",
     xlab = expression(paste("Over-dispersion G1 cells (",hat(delta)[i]^{(G1)},")")),
     ylab = expression(paste("Over-dispersion S cells (",hat(delta)[i]^{(S)},")")),
     xlim = lim2, ylim = lim2, cex = 2)
abline(a = 0, b = 1, lty = 2, lwd = 3)

aux = sum(apply(MCMC_Output1_1@delta, 2, median) <
                      apply(MCMC_Output2_1@delta, 2, median)) /
              length(apply(MCMC_Output1_1@mu, 2, median))
```

```r
text(x = lim2[1]+.05, y = lim2[2], paste0(round(100*aux,1),"%"), cex = 3, col = "blue")
text(x = lim2[2]-10, y = lim2[1]+.025, paste0(100-round(100*aux,1),"%"), cex = 3, col = "blue")


plot(c(0, 1), c(0, 1), ann = F, bty = 'n', type = 'n',
       xaxt = 'n', yaxt = 'n')
rect(0, 0, 1, 1, col = unique(Cell.Colour)[2])
text(x = 0.5, y = 0.5, paste("S"), cex = 10, col = "white", lwd = 2)

plot(apply(MCMC_Output3_1@mu, 2, median),
     apply(MCMC_Output2_1@mu, 2, median), pch = 16, col = rgb(190,190,190,50,maxColorValue=255),
     bty = "n", log = "xy",
     xlab = expression(paste("Overall expression G2M cells (",hat(mu)[i]^{(G2M)},")")),
     ylab = expression(paste("Overall expression S cells (",hat(mu)[i]^{(S)},")")),
     xlim = lim1, ylim = lim1, cex = 2)
abline(a = 0, b = 1, lty = 2, lwd = 3)

aux = round(100*sum(apply(MCMC_Output3_1@mu, 2, median) <
                      apply(MCMC_Output2_1@mu, 2, median)) /
              length(apply(MCMC_Output1_1@mu, 2, median)),1)

text(x = lim1[1]+5, y = lim1[2], paste0(aux,"%"), cex = 3, col = "blue")
text(x = lim1[2]-5000, y = lim1[1]+1, paste0(100-aux,"%"), cex = 3, col = "blue")

plot(apply(MCMC_Output1@delta, 2, median),
     apply(MCMC_Output3@delta, 2, median), pch = 16, col = rgb(190,190,190,50,maxColorValue=255),
     bty = "n", log = "xy",
     xlab = expression(paste("Over-dispersion G1 cells (",hat(delta)[i]^{(G1)},")")),
     ylab = expression(paste("Over-dispersion G2M cells (",hat(delta)[i]^{(G2M)},")")),
     xlim = lim2, ylim = lim2, cex = 2)
abline(a = 0, b = 1, lty = 2, lwd = 3)

aux = round(100*sum(apply(MCMC_Output1@delta, 2, median) <
                      apply(MCMC_Output3@delta, 2, median)) /
              length(apply(MCMC_Output1_1@mu, 2, median)),1)

text(x = lim2[1]+.05, y = lim2[2], paste0(aux,"%"), cex = 3, col = "blue")
text(x = lim2[2]-10, y = lim2[1]+.025, paste0(100-aux,"%"), cex = 3, col = "blue")

plot(apply(MCMC_Output2@delta, 2, median),
     apply(MCMC_Output3@delta, 2, median), pch = 16, col = rgb(190,190,190,50,maxColorValue=255),
     bty = "n", log = "xy",
     xlab = expression(paste("Over-dispersion S cells (",hat(delta)[i]^{(S)},")")),
     ylab = expression(paste("Over-dispersion G2M cells (",hat(delta)[i]^{(G2M)},")")),
     xlim = lim2, ylim = lim2, cex = 2)
abline(a = 0, b = 1, lty = 2, lwd = 3)

aux = round(100*sum(apply(MCMC_Output2@delta, 2, median) <
                      apply(MCMC_Output3@delta, 2, median)) /
              length(apply(MCMC_Output1_1@mu, 2, median)),1)

text(x = lim2[1]+.05, y = lim2[2], paste0(aux,"%"), cex = 3, col = "blue")
text(x = lim2[2]-10, y = lim2[1]+.025, paste0(100-aux,"%"), cex = 3, col = "blue")
```

```
plot(c(0, 1), c(0, 1), ann = F, bty = 'n', type = 'n',
       xaxt = 'n', yaxt = 'n')
rect(0, 0, 1, 1, col = unique(Cell.Colour)[3])
text(x = 0.5, y = 0.5, paste("G2M"), cex = 10, col = "white", lwd = 2)

title("(d)", outer=TRUE, cex.main = 3)
```

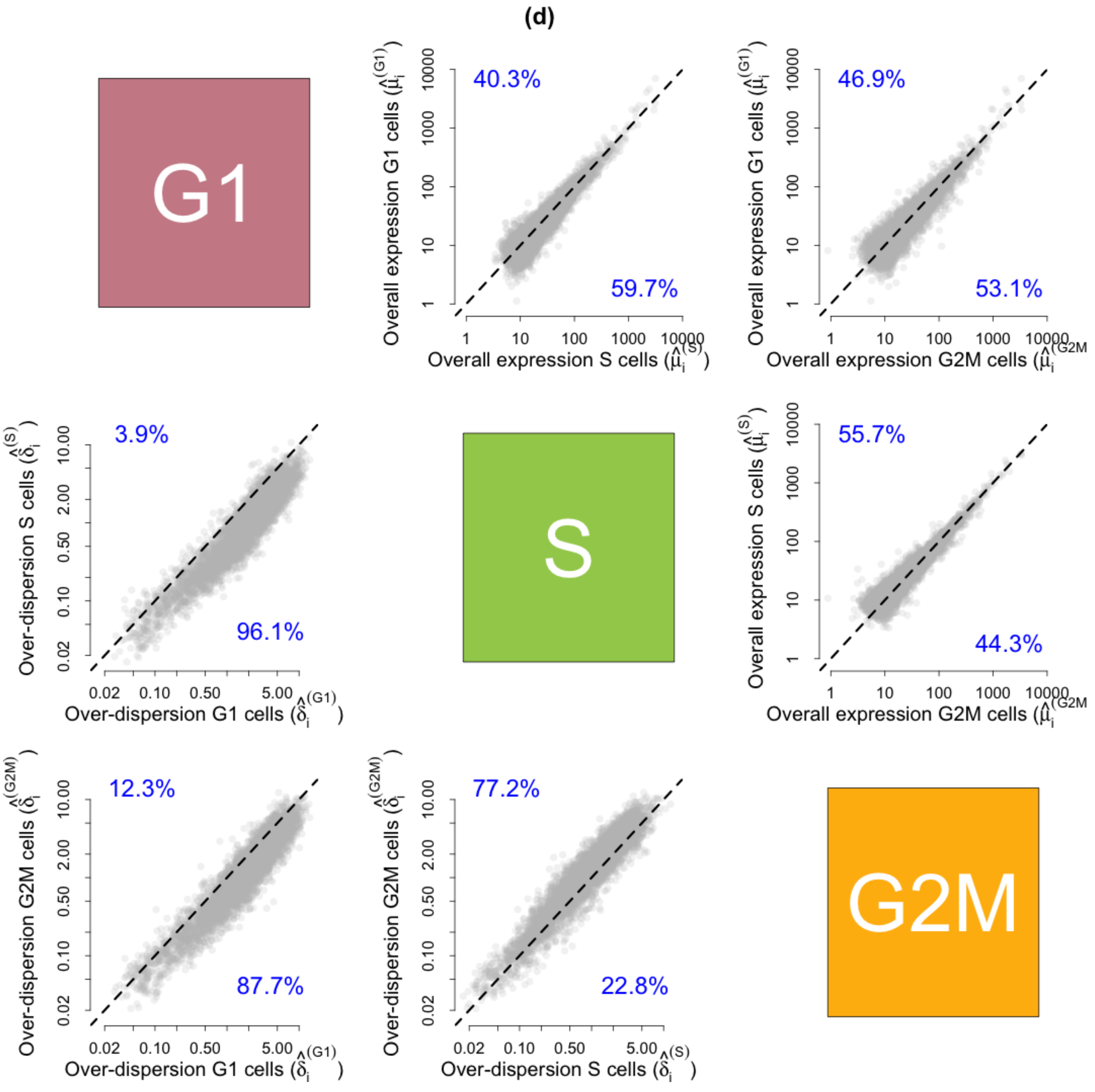


```
par(mfrow = c(1,3))
par(cex.lab = 3, cex.main = 3, cex.axis = 3)
par(mar = c(7, 10, 4, 2) + 0.1)
par(mgp=c(5,2,0))
par(lwd = 4)
```

```r
GroupCells <- c(rep(1, times = ncol(Counts.G1)),
           rep(2, times = ncol(Counts.S)),
           rep(3, times = ncol(Counts.G2M)))

boxplot(displaySummaryBASiCS(MCMC_Summary, Param = "phi")[,1] ~ GroupCells, main = "(a)",
        names = c("G1", "S", "G2M"), col = unique(Cell.Colour), frame = F,
        ylab = expression(paste("mRNA content (",hat(phi)[j]^{(p)},")")))

GroupGenes <- c(rep(1, times = sum(!Tech)),
                rep(2, times = sum(!Tech)),
                rep(3, times = sum(!Tech)))

boxplot(displaySummaryBASiCS(MCMC_Summary, Param = "mu")[,1] ~ GroupGenes, main = "(b)",
        names = c("G1", "S", "G2M"), col = unique(Cell.Colour), log = "y", frame = FALSE,
        ylab = expression(paste("Overall expression (",hat(mu)[i]^{(p)},")")))

boxplot(displaySummaryBASiCS(MCMC_Summary, Param = "delta")[,1] ~ GroupGenes, main = "(c)",
        names = c("G1", "S", "G2M"), col = unique(Cell.Colour), frame = FALSE,
        ylab = expression(paste("Biological over-dispersion (",hat(delta)[i]^{(p)},")")))
```

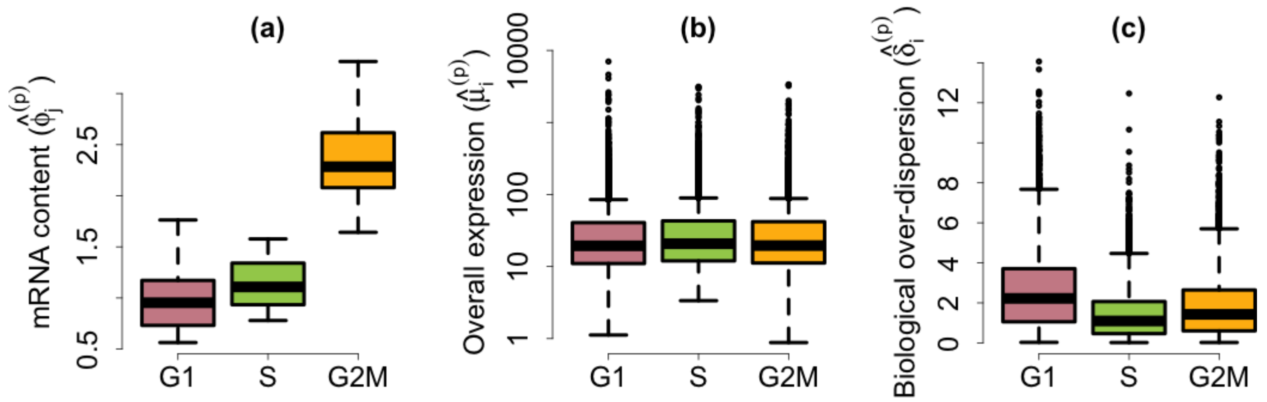

```r
library(gplots) # To construct heatmaps
```

```
##
## Attaching package: 'gplots'

## The following object is masked from 'package:stats':
##
##     lowess
```

```r
HeatmapFormat <- function(x, GenesSel = NULL, Colv = FALSE, Rowv= FALSE, ...)
{
  if(is.null(GenesSel)) {GenesSel = rownames(x)}
  heatmap_colour_scale<-colorRampPalette(c("#313695", "#74add1", "#ffffbf","#fdae61","#d73027"))(15)
  xsel = x[which(rownames(x) %in% GenesSel), ]
  heatmap.2(log(xsel+1), dendrogram = "none", trace = "none",
            Colv = Colv, Rowv= Rowv, margin=c(6,25), col= heatmap_colour_scale, ...)

}
```

Comparison mu vs delta

Differential expression (mean and over-dispersion)

```
Data12 <- CombineBASiCS_Data(Data.G1, Data.S)
```

```
##
## NOTICE: BASiCS requires a pre-filtered dataset
##     - You must remove poor quality cells before creating the BASiCS data object
##     - We recommend to pre-filter very lowly expressed transcripts before creating the object.
##       Inclusion criteria may vary for each data. For example, remove transcripts
##           - with very low total counts across of all cells
##           - that are only expressed in few cells
##             (by default genes expressed in only 1 cell are not accepted)
##           - with very low total counts across the cells where the transcript is expressed
##
##  BASiCS_Filter can be used for this purpose
##
## An object of class BASiCS_D_Data
##  Dataset contains 5687 genes (5634 biological and 53 technical) and 117 cells.
##        - 59 cells in the test sample, with1batch(es)
##        - 58 cells in the reference sample, with1batch(es)
##  Elements (slots): CountsTest, CountsRef, Tech, SpikeInputTest, SpikeInputRef, BatchInfoTest, BatchIn
```

```
Data13 <- CombineBASiCS_Data(Data.G1, Data.G2M)
```

```
##
## NOTICE: BASiCS requires a pre-filtered dataset
##     - You must remove poor quality cells before creating the BASiCS data object
##     - We recommend to pre-filter very lowly expressed transcripts before creating the object.
##       Inclusion criteria may vary for each data. For example, remove transcripts
##           - with very low total counts across of all cells
##           - that are only expressed in few cells
##             (by default genes expressed in only 1 cell are not accepted)
##           - with very low total counts across the cells where the transcript is expressed
##
##  BASiCS_Filter can be used for this purpose
##
## An object of class BASiCS_D_Data
##  Dataset contains 5687 genes (5634 biological and 53 technical) and 124 cells.
##        - 59 cells in the test sample, with1batch(es)
##        - 65 cells in the reference sample, with1batch(es)
##  Elements (slots): CountsTest, CountsRef, Tech, SpikeInputTest, SpikeInputRef, BatchInfoTest, BatchIn
```

```
Data23 <- CombineBASiCS_Data(Data.S, Data.G2M)
```

```
##
## NOTICE: BASiCS requires a pre-filtered dataset
```

```
##     - You must remove poor quality cells before creating the BASiCS data object
##     - We recommend to pre-filter very lowly expressed transcripts before creating the object.
##       Inclusion criteria may vary for each data. For example, remove transcripts
##           - with very low total counts across of all cells
##           - that are only expressed in few cells
##             (by default genes expressed in only 1 cell are not accepted)
##           - with very low total counts across the cells where the transcript is expressed
##
##  BASiCS_Filter can be used for this purpose
##
## An object of class BASiCS_D_Data
##  Dataset contains 5687 genes (5634 biological and 53 technical) and 123 cells.
##         - 58 cells in the test sample, with1batch(es)
##         - 65 cells in the reference sample, with1batch(es)
##  Elements (slots): CountsTest, CountsRef, Tech, SpikeInputTest, SpikeInputRef, BatchInfoTest, BatchIn
```

```
MCMC_Output12 <- CombineBASiCS_Chain(MCMC_Output1_1, MCMC_Output2_1)
```

```
## An object of class BASiCS_D_Chain
##  1000 MCMC samples.
##  Dataset contains 5634 biological genes and 117 cells (in total across both samples).
##  Offset = 1.
##  Elements (slots): muTest, muRef, deltaTest, omegaRef, phi, s, nu, thetaTest, thetaRef and offset.
```

```
MCMC_Output13 <- CombineBASiCS_Chain(MCMC_Output1_1, MCMC_Output3_1)
```

```
## An object of class BASiCS_D_Chain
##  1000 MCMC samples.
##  Dataset contains 5634 biological genes and 124 cells (in total across both samples).
##  Offset = 1.
##  Elements (slots): muTest, muRef, deltaTest, omegaRef, phi, s, nu, thetaTest, thetaRef and offset.
```

```
MCMC_Output23 <- CombineBASiCS_Chain(MCMC_Output2_1, MCMC_Output3_1)
```

```
## An object of class BASiCS_D_Chain
##  1000 MCMC samples.
##  Dataset contains 5634 biological genes and 123 cells (in total across both samples).
##  Offset = 1.
##  Elements (slots): muTest, muRef, deltaTest, omegaRef, phi, s, nu, thetaTest, thetaRef and offset.
```

```
Test12_0 <- BASiCS_D_TestDE(Data12, MCMC_Output12,
                             GeneNames = Genes.ids[!Data12@Tech],
                             EpsilonM = 0, EpsilonD = 0,
                             EFDR_M = 0.05, EFDR_D = 0.05,
                             OrderVariable = "GeneIndex",
                             GroupLabelRef = "S", GroupLabelTest = "G1",
                             OffSet = FALSE)
```

```
## ------------------------------------------------------------------------
## 2307  genes with a change on the overall expression:
## - Higher expression in  G1 group: 882
```

```
## - Higher expression in  S group: 1425
## - Fold change tolerance =  0 %
## - Evidence threshold =  0.82675
## - EFDR =  4.96 %
## - EFNR =  45.02 %
## ---------------------------------------------------------------------
##
## ---------------------------------------------------------------------
## 5228  genes with a change on the cell-to-cell biological over dispersion:
## - Higher over dispersion in  G1 group: 5142
## - Higher over dispersion in  S group: 86
## - Fold change tolerance =  0 %
## - Evidence threshold =  0.50075
## - EFDR =  4.9 %
## - EFNR =  27.79 %
## ---------------------------------------------------------------------
```

```
Test12_40 <- BASiCS_D_TestDE(Data12, MCMC_Output12,
                             GeneNames = Genes.ids[!Data12@Tech],
                             EpsilonM = 0.4, EpsilonD = 0.4,
                             EFDR_M = 0.05, EFDR_D = 0.05,
                             OrderVariable = "GeneIndex",
                             GroupLabelRef = "S", GroupLabelTest = "G1",
                             OffSet = FALSE)
```

```
## ---------------------------------------------------------------------
## 343  genes with a change on the overall expression:
## - Higher expression in  G1 group: 110
## - Higher expression in  S group: 233
## - Fold change tolerance =  40 %
## - Evidence threshold =  0.88025
## - EFDR =  5.01 %
## - EFNR =  26.88 %
## ---------------------------------------------------------------------
##
## ---------------------------------------------------------------------
## 3197  genes with a change on the cell-to-cell biological over dispersion:
## - Higher over dispersion in  G1 group: 3189
## - Higher over dispersion in  S group: 8
## - Fold change tolerance =  40 %
## - Evidence threshold =  0.8015
## - EFDR =  5 %
## - EFNR =  45.55 %
## ---------------------------------------------------------------------
```

```
Test13_0 <- BASiCS_D_TestDE(Data13, MCMC_Output13,
                             GeneNames = Genes.ids[!Data13@Tech],
                             EpsilonM = 0, EpsilonD = 0,
                             EFDR_M = 0.05, EFDR_D = 0.05,
                             OrderVariable = "GeneIndex",
                             GroupLabelRef = "G2M", GroupLabelTest = "G1",
                             OffSet = FALSE)
```

```
## ---------------------------------------------------------------------
```

```
## 2613  genes with a change on the overall expression:
## - Higher expression in  G1 group: 1206
## - Higher expression in  G2M group: 1407
## - Fold change tolerance =  0 %
## - Evidence threshold =  0.81475
## - EFDR =  5 %
## - EFNR =  43.81 %
## ----------------------------------------------------------------------
##
## ----------------------------------------------------------------------
## 3872  genes with a change on the cell-to-cell biological over dispersion:
## - Higher over dispersion in  G1 group: 3706
## - Higher over dispersion in  G2M group: 166
## - Fold change tolerance =  0 %
## - Evidence threshold =  0.74075
## - EFDR =  5 %
## - EFNR =  40.88 %
## ----------------------------------------------------------------------
```

```
Test13_40 <- BASiCS_D_TestDE(Data13, MCMC_Output13,
                               GeneNames = Genes.ids[!Data12@Tech],
                             EpsilonM = .4, EpsilonD = .4,
                             EFDR_M = 0.05, EFDR_D = 0.05,
                             OrderVariable = "GeneIndex",
                             GroupLabelRef = "G2M", GroupLabelTest = "G1",
                             OffSet = FALSE)
```

```
## ----------------------------------------------------------------------
## 545  genes with a change on the overall expression:
## - Higher expression in  G1 group: 229
## - Higher expression in  G2M group: 316
## - Fold change tolerance =  40 %
## - Evidence threshold =  0.85925
## - EFDR =  5.03 %
## - EFNR =  27.73 %
## ----------------------------------------------------------------------
##
## ----------------------------------------------------------------------
## 1550  genes with a change on the cell-to-cell biological over dispersion:
## - Higher over dispersion in  G1 group: 1535
## - Higher over dispersion in  G2M group: 15
## - Fold change tolerance =  40 %
## - Evidence threshold =  0.84725
## - EFDR =  4.99 %
## - EFNR =  37.84 %
## ----------------------------------------------------------------------
```

```
Test23_0 <- BASiCS_D_TestDE(Data23, MCMC_Output23,
                             GeneNames = Genes.ids[!Data23@Tech],
                             EpsilonM = 0, EpsilonD = 0,
                             EFDR_M = 0.05, EFDR_D = 0.05,
                             OrderVariable = "GeneIndex",
                             GroupLabelRef = "G2M", GroupLabelTest = "S",
                             OffSet = FALSE)
```

```
## ---------------------------------------------------------------------
## 2038  genes with a change on the overall expression:
## - Higher expression in  S group: 1166
## - Higher expression in  G2M group: 872
## - Fold change tolerance =  0 %
## - Evidence threshold =  0.83875
## - EFDR =  5.02 %
## - EFNR =  45.06 %
## ---------------------------------------------------------------------
##
## ---------------------------------------------------------------------
## 2676  genes with a change on the cell-to-cell biological over dispersion:
## - Higher over dispersion in  S group: 323
## - Higher over dispersion in  G2M group: 2353
## - Fold change tolerance =  0 %
## - Evidence threshold =  0.82275
## - EFDR =  5.02 %
## - EFNR =  46.92 %
## ---------------------------------------------------------------------
```

```
Test23_40 <- BASiCS_D_TestDE(Data23, MCMC_Output23,
                               GeneNames = Genes.ids[!Data23@Tech],
                             EpsilonM = .4, EpsilonD = .4,
                             EFDR_M = 0.05, EFDR_D = 0.05,
                             OrderVariable = "GeneIndex",
                             GroupLabelRef = "G2M", GroupLabelTest = "S",
                             OffSet = FALSE)
```

```
## ---------------------------------------------------------------------
## 209  genes with a change on the overall expression:
## - Higher expression in  S group: 126
## - Higher expression in  G2M group: 83
## - Fold change tolerance =  40 %
## - Evidence threshold =  0.88525
## - EFDR =  4.99 %
## - EFNR =  20.77 %
## ---------------------------------------------------------------------
##
## ---------------------------------------------------------------------
## 593  genes with a change on the cell-to-cell biological over dispersion:
## - Higher over dispersion in  S group: 62
## - Higher over dispersion in  G2M group: 531
## - Fold change tolerance =  40 %
## - Evidence threshold =  0.87925
## - EFDR =  4.99 %
## - EFNR =  35.81 %
## ---------------------------------------------------------------------
```

```
ColourBarplot12 = c(rep(unique(Cell.Colour)[1], 3),
                  rep("grey", 3),
                  rep(unique(Cell.Colour)[2], 3))

ColourBarplot13 = c(rep(unique(Cell.Colour)[1], 3),
```

```
                  rep("grey", 3),
                  rep(unique(Cell.Colour)[3], 3))

ColourBarplot23 = c(rep(unique(Cell.Colour)[2], 3),
                  rep("grey", 3),
                  rep(unique(Cell.Colour)[3], 3))

par(mfrow = c(1,3))
par(mar = c(6, 7, 4, 5) + 0.1)
par(mgp = c(5, 1, 0))
par(cex.main = 3.5, cex.axis = 2, cex.lab = 2.5)

barplot(table(Test12_40$Table$ResultDiffOverDisp,
              Test12_40$Table$ResultDiffExp) + 1,
        col = unique(ColourBarplot12), beside = TRUE, cex.names = 2.5,
        ylim = c(1, 15000), log = "y", xlab = "Changes in overall expression",
        main = expression(paste(tau[0], "= 0.4, ", omega[0], "= 0.4")),
        ylab = "Number of genes", axes = FALSE,
        names.arg = c("G1 +", "No diff.", "S +"))

text(x = c(1.5, 2.5, 3.5, 5.3, 6.7, 7.5, 9.5, 10.5, 11.5),
     y = pmax(6, 1.9 * as.vector(table(Test12_40$Table$ResultDiffOverDisp,
                         Test12_40$Table$ResultDiffExp))),
     as.vector(table(Test12_40$Table$ResultDiffOverDisp,
                     Test12_40$Table$ResultDiffExp)),
     cex = 2, col = "black")

barplot(table(Test13_40$Table$ResultDiffOverDisp,
              Test13_40$Table$ResultDiffExp)[c(1,3,2), c(1,3,2)] + 1,
        col = unique(ColourBarplot13), beside = TRUE, cex.names = 2.5,
        ylim = c(1, 15000), log = "y", xlab = "Changes in overall expression",
        main = expression(paste(tau[0], "= 0.4, ", omega[0], "= 0.4")),
        ylab = "Number of genes", axes = FALSE,
        names.arg = c("G1 +", "No diff.", "G2M +"))

text(x = c(1.5, 2.5, 3.5, 5.1, 6.7, 7.5, 9.5, 10.5, 11.5),
     y = pmax(6, 1.9 * as.vector(table(Test13_40$Table$ResultDiffOverDisp,
                         Test13_40$Table$ResultDiffExp)[c(1,3,2), c(1,3,2)])),
     as.vector(table(Test13_40$Table$ResultDiffOverDisp,
                     Test13_40$Table$ResultDiffExp)[c(1,3,2), c(1,3,2)]),
     cex = 2, col = "black")

barplot(table(Test23_40$Table$ResultDiffOverDisp,
              Test23_40$Table$ResultDiffExp)[c(3,2,1), c(3,2,1)] + 1,
        col = unique(ColourBarplot23), beside = TRUE, cex.names = 2.5,
        ylim = c(1, 15000), log = "y", xlab = "Changes in overall expression",
        main = expression(paste(tau[0], "= 0.4, ", omega[0], "= 0.4")),
        ylab = "Number of genes", axes = FALSE,
        names.arg = c("S +", "No diff.", "G2M +"))

text(x = c(1.5, 2.5, 3.5, 5.3, 6.5, 7.7, 9.5, 10.5, 11.5),
     y = pmax(6, 1.9 * as.vector(table(Test23_40$Table$ResultDiffOverDisp,
                         Test23_40$Table$ResultDiffExp)[c(3,2,1), c(3,2,1)])),
```

```
    as.vector(table(Test23_40$Table$ResultDiffOverDisp,
                    Test23_40$Table$ResultDiffExp)[c(3,2,1), c(3,2,1)]),
    cex = 2, col = "black")
```

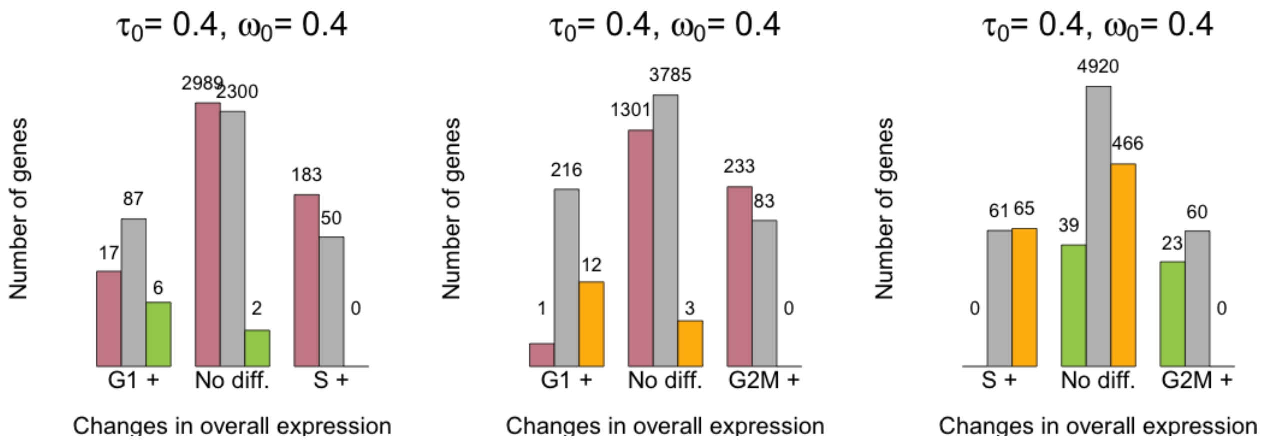


```
par(mfrow = c(1,3))
par(mar = c(6, 7, 4, 5) + 0.1)
par(mgp = c(5, 1, 0))
par(cex.main = 3.5, cex.axis = 2, cex.lab = 2.5)
plot(MCMC_Summary1, Param = "mu", Param2 = "delta", log = "xy",
     col = unique(Cell.Colour)[1], main = "G1 cells",
     ylab = "Over-dispersion", xlab = "Overall expression")
plot(MCMC_Summary2, Param = "mu", Param2 = "delta", log = "xy",
     col = unique(Cell.Colour)[2], main = "S cells",
     ylab = "Over-dispersion", xlab = "Overall expression")
plot(MCMC_Summary3, Param = "mu", Param2 = "delta", log = "xy",
     col = unique(Cell.Colour)[3], main = "G2M cells",
     ylab = "Over-dispersion", xlab = "Overall expression")
```

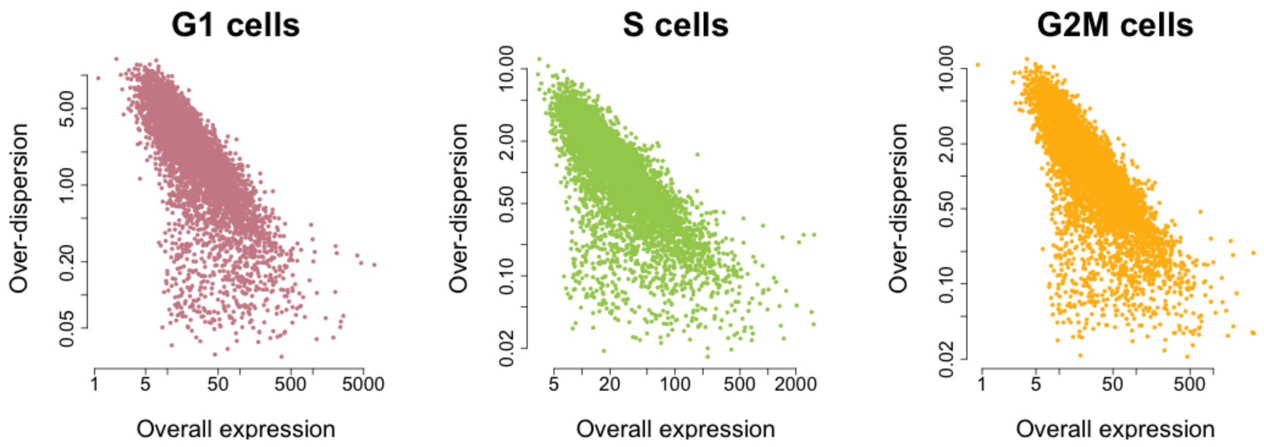


```
par(mfrow = c(1,3))
par(mar = c(6, 7, 4, 5) + 0.1)
par(mgp = c(5, 1, 0))
par(cex.main = 3.5, cex.axis = 2, cex.lab = 2.5)
plot(Test12_40$Table$ExpLogFC, Test12_40$Table$OverDispLogFC,
     bty = "n", col = 8, pch = 16,
```

```
     ylab = "LFC in over-dispersion",
     xlab = "LFC in expression",
     main = "G1 vs S cells",
     ylim = c(-3,3), xlim = c(-3,3))

plot(Test13_40$Table$ExpLogFC, Test13_40$Table$OverDispLogFC,
     bty = "n", col = 8, pch = 16,
     ylab = "LFC in over-dispersion",
     xlab = "LFC in expression",
     main = "G1 vs G2M cells",
     ylim = c(-3,3), xlim = c(-3,3))

plot(Test23_40$Table$ExpLogFC, Test23_40$Table$OverDispLogFC,
     bty = "n", col = 8, pch = 16,
     ylab = "LFC in over-dispersion",
     xlab = "LFC in expression",
     main = "S vs G2M cells",
     ylim = c(-3,3), xlim = c(-3,3))
```

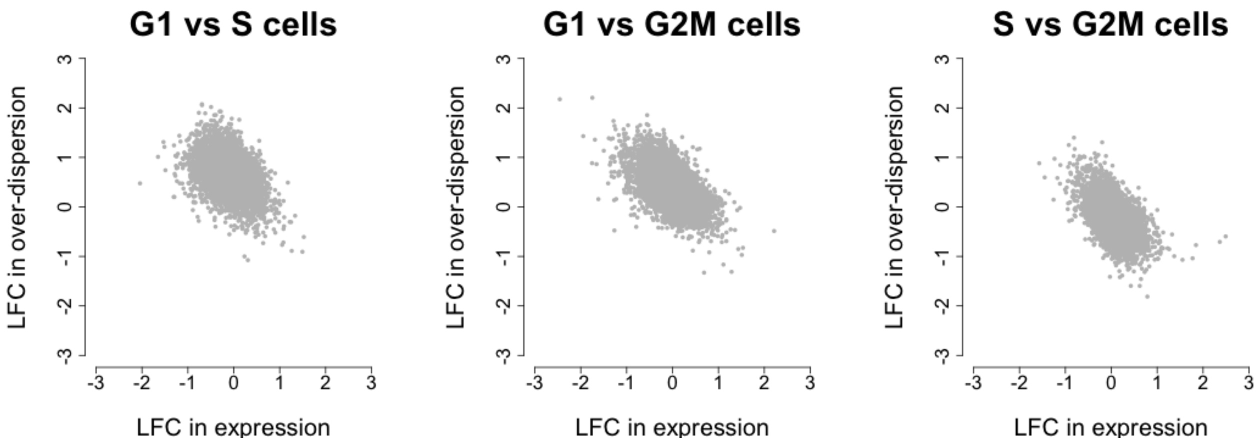


```
DC.G1 = BASiCS_DenoisedCounts(Data = Data.G1, Chain = MCMC_Output1_1)[!Data.G1@Tech,]
DC.S = BASiCS_DenoisedCounts(Data = Data.S, Chain = MCMC_Output2_1)[!Data.S@Tech,]
DC.G2M = BASiCS_DenoisedCounts(Data = Data.G2M, Chain = MCMC_Output3_1)[!Data.G2M@Tech,]

DC.all = cbind(DC.G1, DC.S, DC.G2M)

# Heatmaps for differentially expressed genes with LFC above 1.5 (in log2 scale)
Test12_40$Table = Test12_40$Table[order(as.numeric(row.names(Test12_40$Table))),]
sum(!(rownames(MCMC_Summary1@mu) == Data.G1@GeneNames[!Data.G1@Tech]))
```

```
## [1] 0
```

```
Test13_40$Table = Test13_40$Table[order(as.numeric(row.names(Test13_40$Table))),]
sum(!(rownames(DC.all) == Data.G1@GeneNames[!Data.G1@Tech]))
```

```
## [1] 0
```

```
Test23_40$Table = Test23_40$Table[order(as.numeric(row.names(Test23_40$Table))),]
sum(!(rownames(DC.all) == Data.G1@GeneNames[!Data.G1@Tech]))
```

```
## [1] 0
```

```
HeatmapFormat(cbind(DC.G1, DC.S)[order(Test12_40$Table$ExpLogFC),],
              GenesSel = Test12_40$Table$GeneNames[Test12_40$Table$ResultDiffExp != "NoDiff" ],
              ColSideColors = Cell.Colour[!grepl("G2M", colnames(DC.all))],
              main = "Differentially expressed genes (tau0 = 0.4)", Rowv = TRUE)
legend('topright', c("G1", "S"), pch = 15, col = unique(Cell.Colour)[1:2])
```

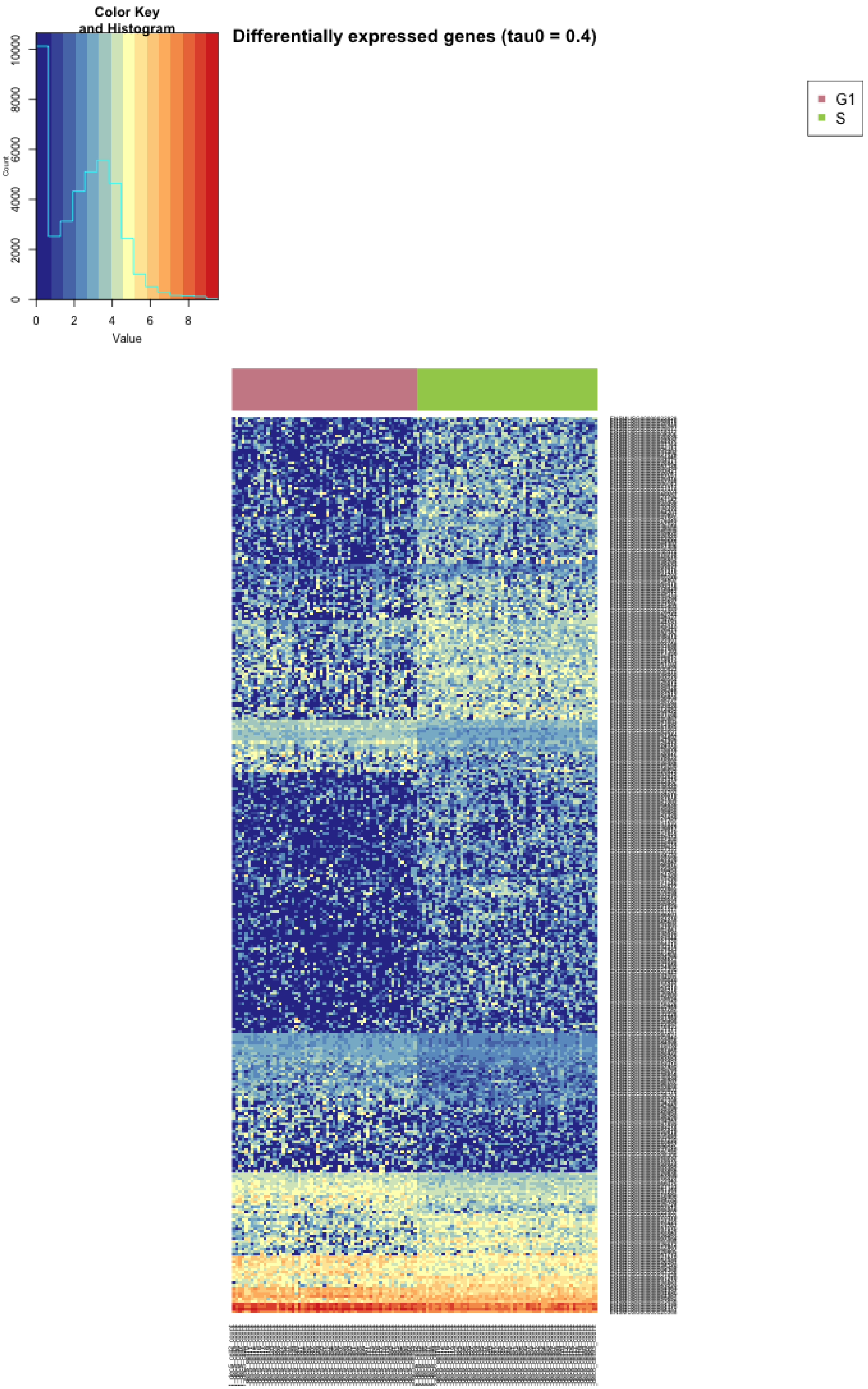

Color Key
and Histogram
Count
Value
Differentially expressed genes (tau0 = 0.4)
G1
S

```
HeatmapFormat(cbind(DC.G1, DC.G2M)[order(Test13_40$Table$ExpLogFC),],
              GenesSel = Test13_40$Table$GeneNames[Test13_40$Table$ResultDiffExp != "NoDiff"],
              ColSideColors = Cell.Colour[!grepl("S", colnames(DC.all))],
              main = "Differentially expressed genes (tau0 = 0.4)", Rowv = TRUE)
legend('topright', c("G1", "G2M"), pch = 15, col = unique(Cell.Colour)[c(1,3)])
```

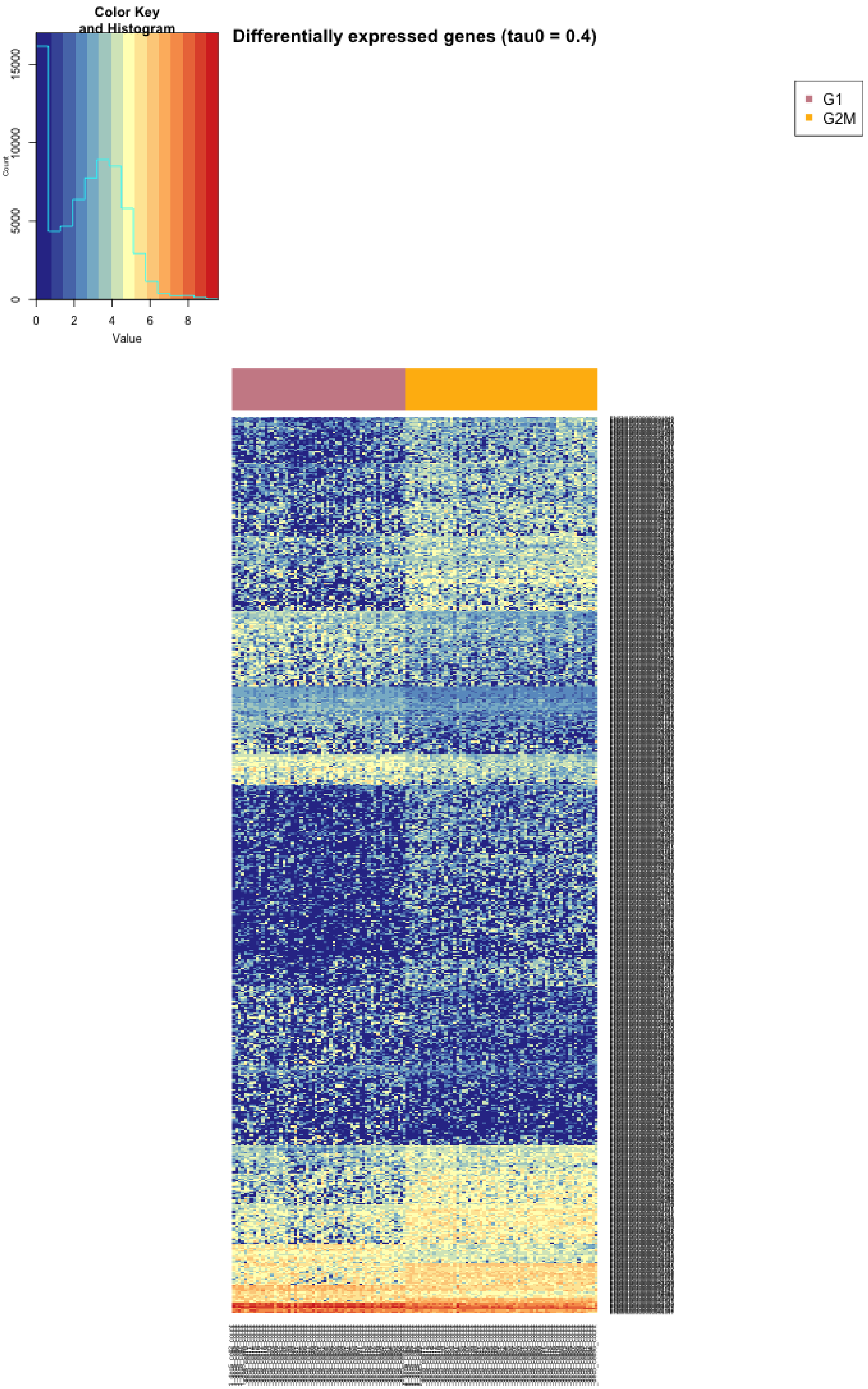
Color Key
and Histogram
Count
Value
Differentially expressed genes (tau0 = 0.4)
G1
G2M

```r
HeatmapFormat(cbind(DC.S, DC.G2M)[order(Test23_40$Table$ExpLogFC),],
              GenesSel = Test23_40$Table$GeneNames[Test23_40$Table$ResultDiffExp != "NoDiff"],
              ColSideColors = Cell.Colour[!grepl("G1", colnames(DC.all))],
              main = "Differentially expressed genes (tau0 = 0.4)", Rowv = TRUE)
legend('topright', c("S", "G2M"), pch = 15, col = unique(Cell.Colour)[c(2,3)])
```

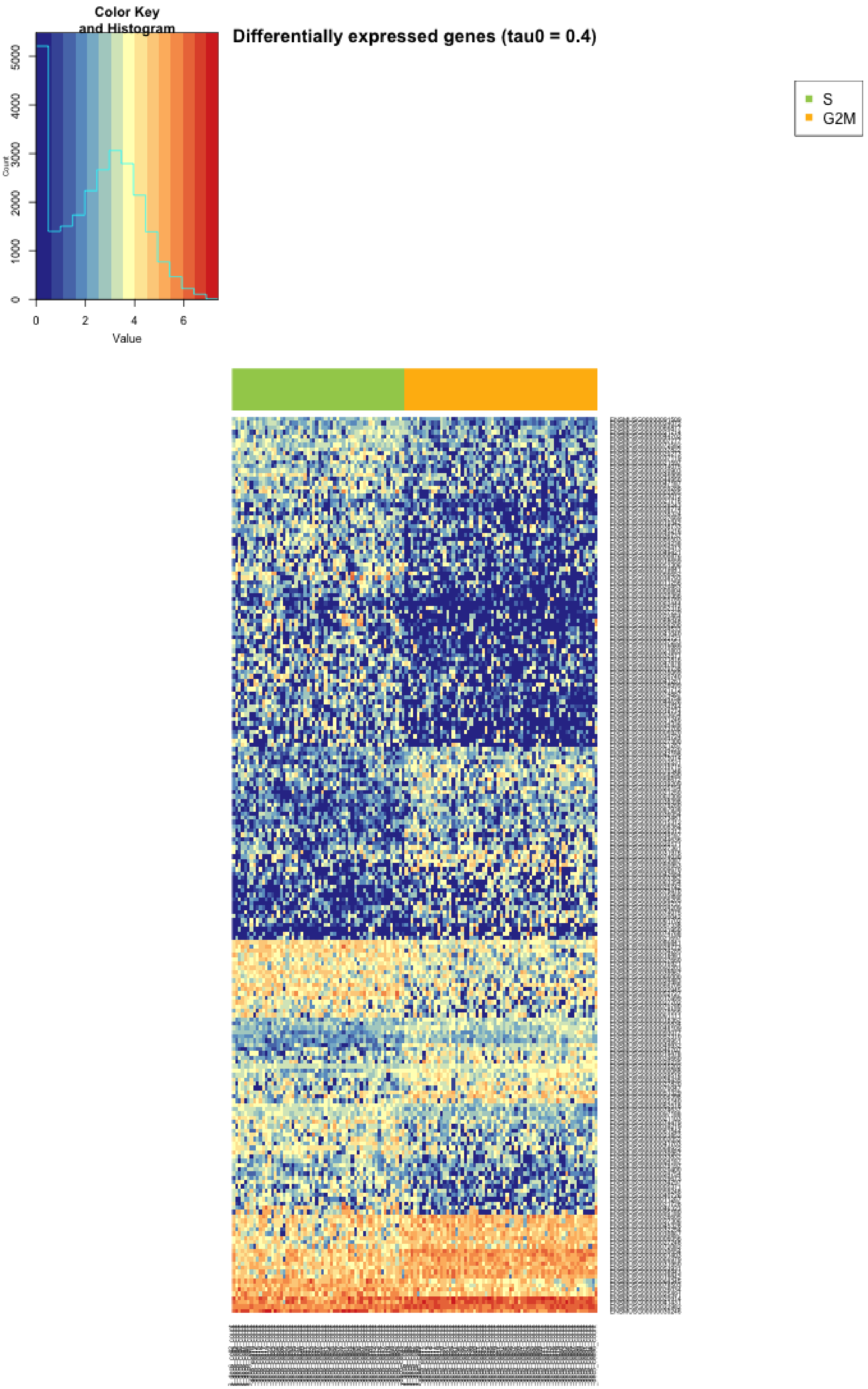
Color Key
and Histogram
Count
Value
Differentially expressed genes (tau0 = 0.4)
S
G2M

GO enrichment analysis

Finally, we perform a GO enrichment analysis of those genes identified as differentially expressed (mean and over-dispersion). For this, we first transform gene names into the required format.

```
# GO enrichment analysis ####

# Transforming ENSMUSG to gene names (using Biomart as a source)
GenesTransform = fread(file.path(data.path,"mart_export.txt"), sep = ",")
setkey(GenesTransform, "EnsemblGeneID")
GenesTransform1 = unique(GenesTransform)

GenesTransform2 = subset(GenesTransform1, EnsemblGeneID %in% Genes.ids)
setnames(GenesTransform2 , names(GenesTransform2),
         c("GeneNames", "EnsemblTranscriptID", "AssociatedGeneName"))
sum(!Tech) - nrow(GenesTransform2)
```

```
## [1] 21
```

```
# The following 21 ENSMUSG ids were not found in ensembl.org Biomark
#Genes.ids[which(!(Genes.ids[!Tech] %in% GenesTransform2$EnsemblGeneID) )]

Test12_0_DT = merge(as.data.table(Test12_0$Table), GenesTransform2,
                    by = "GeneNames", all.x = TRUE)
Test13_0_DT = merge(as.data.table(Test13_0$Table), GenesTransform2,
                    by = "GeneNames", all.x = TRUE)
Test23_0_DT = merge(as.data.table(Test23_0$Table), GenesTransform2,
                    by = "GeneNames", all.x = TRUE)

Test12_40_DT = merge(as.data.table(Test12_40$Table), GenesTransform2,
                    by = "GeneNames", all.x = TRUE)
Test13_40_DT = merge(as.data.table(Test13_40$Table), GenesTransform2,
                    by = "GeneNames", all.x = TRUE)
Test23_40_DT = merge(as.data.table(Test23_40$Table), GenesTransform2,
                    by = "GeneNames", all.x = TRUE)
```

The following function is used to perform the enrichment analysis. It have been adapted from the code provided by Brennecke et al (2013)

```
require( topGO ) # To perform GO enrichment analysis
require( org.Mm.eg.db ) # To perform GO enrichment analysis (map of Musmusculus genes)
require(GO.db) # To perform GO enrichment analysis

annFUN.symbol <- function (whichOnto, feasibleGenes = NULL, mapping)
  annFUN.org(whichOnto, feasibleGenes, mapping, ID = 'symbol')
topGOAnalysis <- function( geneIDs, inUniverse, inSelection ) {
  f <- function( ont ) {
    alg <- factor( as.integer( inSelection[inUniverse] ) )
    names(alg) <- geneIDs[inUniverse]
    tgd <- new( "topGOdata", ontology=ont, allGenes = alg, nodeSize=5,
```

```
                annot=annFUN.symbol, mapping="org.Mm.eg.db" )
    resultTopGO <- runTest(tgd, algorithm = "elim", statistic = "Fisher" )
    GenTable( tgd, resultTopGO, topNodes=15 ) }

  sapply( c( "MF", "BP", "CC" ), f, simplify=FALSE )
}
```

Differentially expressed genes (mean)

```
goEnrichDE12_40<-topGOAnalysis(Test12_40_DT$AssociatedGeneName[!is.na(Test12_40_DT$AssociatedGeneName)]
                           rep(T,sum(!is.na(Test12_40_DT$AssociatedGeneName))),
                           Test12_40_DT$ResultDiffExp != "NoDiff")


goEnrichDE13_40<-topGOAnalysis(Test13_40_DT$AssociatedGeneName[!is.na(Test13_40_DT$AssociatedGeneName)]
                           rep(T,sum(!is.na(Test13_40_DT$AssociatedGeneName))),
                           Test13_40_DT$ResultDiffExp != "NoDiff")

goEnrichDE23_40<-topGOAnalysis(Test23_40_DT$AssociatedGeneName[!is.na(Test23_40_DT$AssociatedGeneName)]
                           rep(T,sum(!is.na(Test23_40_DT$AssociatedGeneName))),
                           Test23_40_DT$ResultDiffExp != "NoDiff")
```

```
# Changes in mean between G1 and S phases
goEnrichDE12_40$BP
```

```
##         GO.ID                                        Term Annotated
## 1  GO:0070527                         platelet aggregation        19
## 2  GO:0042772 DNA damage response, signal transduction...         5
## 3  GO:0000920              cell separation after cytokinesis        11
## 4  GO:0021522    spinal cord motor neuron differentiation         6
## 5  GO:0050881                      musculoskeletal movement         6
## 6  GO:0006878                cellular copper ion homeostasis         6
## 7  GO:0070306                lens fiber cell differentiation         6
## 8  GO:0050885 neuromuscular process controlling balanc...        20
## 9  GO:0007040                         lysosome organization        20
## 10 GO:0071248                 cellular response to metal ion        21
## 11 GO:0030218                    erythrocyte differentiation        41
## 12 GO:0015936                   coenzyme A metabolic process         8
## 13 GO:0006900                               membrane budding        32
## 14 GO:0006814                          sodium ion transport        32
## 15 GO:0010824        regulation of centrosome duplication        18
##    Significant Expected result1
## 1            7     1.11   6e-05
## 2            3     0.29  0.0018
## 3            4     0.64  0.0027
## 4            3     0.35  0.0035
## 5            3     0.35  0.0035
## 6            3     0.35  0.0035
## 7            3     0.35  0.0035
## 8            5     1.17  0.0049
## 9            5     1.17  0.0049
```

```
## 10           5     1.23  0.0062
## 11           7     2.40  0.0088
## 12           3     0.47  0.0089
## 13           6     1.87  0.0094
## 14           6     1.87  0.0094
## 15           4     1.05  0.0182
```

```r
# Changes in mean between G1 and G2M phases
goEnrichDE13_40$BP
```

```
##         GO.ID                                        Term Annotated
## 1  GO:0061640          cytoskeleton-dependent cytokinesis        25
## 2  GO:0000712 resolution of meiotic recombination inte...         7
## 3  GO:0007052                mitotic spindle organization        45
## 4  GO:0021695               cerebellar cortex development        17
## 5  GO:0016579                    protein deubiquitination        54
## 6  GO:0006376                  mRNA splice site selection        19
## 7  GO:0021537                    telencephalon development        65
## 8  GO:1903792        negative regulation of anion transport         5
## 9  GO:0051231                           spindle elongation          5
## 10 GO:0061041                  regulation of wound healing        20
## 11 GO:0007067                     mitotic nuclear division       255
## 12 GO:0051301                                cell division       338
## 13 GO:0008299                isoprenoid biosynthetic process       10
## 14 GO:0043409          negative regulation of MAPK cascade        48
## 15 GO:0000041               transition metal ion transport        28
##    Significant Expected result1
## 1            8     2.36  0.0015
## 2            4     0.66  0.0022
## 3           11     4.25  0.0025
## 4            6     1.61  0.0035
## 5           12     5.10  0.0038
## 6            6     1.80  0.0064
## 7           13     6.14  0.0068
## 8            3     0.47  0.0073
## 9            3     0.47  0.0073
## 10           6     1.89  0.0085
## 11          36    24.10  0.0085
## 12          50    31.95  0.0093
## 13           4     0.95  0.0104
## 14          10     4.54  0.0126
## 15           7     2.65  0.0132
```

```r
# Changes in mean between S and G2M phases
goEnrichDE23_40$BP
```

```
##         GO.ID                                        Term Annotated
## 1  GO:0031145 anaphase-promoting complex-dependent pro...        42
## 2  GO:0051304                         chromosome separation        52
## 3  GO:0051340                   regulation of ligase activity        45
## 4  GO:0051438 regulation of ubiquitin-protein transfer...        46
## 5  GO:0043968                         histone H2A acetylation        12
## 6  GO:0030071 regulation of mitotic metaphase/anaphase...        37
```

```
## 7  GO:1902099 regulation of metaphase/anaphase transit...        37
## 8  GO:0045682        regulation of epidermis development        13
## 9  GO:0045471                          response to ethanol        13
## 10 GO:0071715                          icosanoid transport         5
## 11 GO:1901571             fatty acid derivative transport         5
## 12 GO:0032309                          icosanoid secretion         5
## 13 GO:0007091 metaphase/anaphase transition of mitotic...        39
## 14 GO:0044784 metaphase/anaphase transition of cell cy...        39
## 15 GO:0010038                        response to metal ion        54
##    Significant Expected result1
## 1            7     1.52 0.00068
## 2            7     1.89 0.00248
## 3            6     1.63 0.00527
## 4            6     1.67 0.00588
## 5            3     0.44 0.00811
## 6            5     1.34 0.01010
## 7            5     1.34 0.01010
## 8            3     0.47 0.01026
## 9            3     0.47 0.01026
## 10           2     0.18 0.01217
## 11           2     0.18 0.01217
## 12           2     0.18 0.01217
## 13           5     1.41 0.01259
## 14           5     1.41 0.01259
## 15           6     1.96 0.01277
```

Differentially expressed genes (over-dispersion only)

```
goEnrichDoDnotDE12_G1_40<-topGOAnalysis(Test12_40_DT$AssociatedGeneName[!is.na(Test12_40_DT$AssociatedGe
                           rep(T,sum(!is.na(Test12_40_DT$AssociatedGeneName))),
                           paste0(Test12_40_DT$ResultDiffExp,
                                 Test12_40_DT$ResultDiffOverDisp) == "NoDiffG1+")

goEnrichDoDnotDE12_S_40<-topGOAnalysis(Test12_40_DT$AssociatedGeneName[!is.na(Test12_40_DT$AssociatedGen
                           rep(T,sum(!is.na(Test12_40_DT$AssociatedGeneName))),
                           paste0(Test12_40_DT$ResultDiffExp,
                                 Test12_40_DT$ResultDiffOverDisp) == "NoDiffS+")

goEnrichDoDnotDE13_G1_40<-topGOAnalysis(Test13_40_DT$AssociatedGeneName[!is.na(Test13_40_DT$AssociatedGe
                           rep(T,sum(!is.na(Test13_40_DT$AssociatedGeneName))),
                           paste0(Test13_40_DT$ResultDiffExp,
                                 Test13_40_DT$ResultDiffOverDisp) == "NoDiffG1+")

goEnrichDoDnotDE13_G2M_40<-topGOAnalysis(Test13_40_DT$AssociatedGeneName[!is.na(Test13_40_DT$Associated(
                           rep(T,sum(!is.na(Test13_40_DT$AssociatedGeneName))),
                           paste0(Test13_40_DT$ResultDiffExp,
                                 Test13_40_DT$ResultDiffOverDisp) == "NoDiffG2M+")

goEnrichDoDnotDE23_S_40<-topGOAnalysis(Test23_40_DT$AssociatedGeneName[!is.na(Test23_40_DT$AssociatedGen
                           rep(T,sum(!is.na(Test23_40_DT$AssociatedGeneName))),
                           paste0(Test23_40_DT$ResultDiffExp,
                                 Test23_40_DT$ResultDiffOverDisp) == "NoDiffS+")
```

```
goEnrichDoDnotDE23_G2M_40<-topGOAnalysis(Test23_40_DT$AssociatedGeneName[!is.na(Test23_40_DT$Associated
                           rep(T,sum(!is.na(Test23_40_DT$AssociatedGeneName))),
                           paste0(Test23_40_DT$ResultDiffExp,
                                 Test23_40_DT$ResultDiffOverDisp) == "NoDiffG2M+")
```

```
# Change in over-dispersion between G1 and S (G1+)
goEnrichDoDnotDE12_G1_40$BP
```

```
##         GO.ID                                        Term Annotated
## 1  GO:0032101 regulation of response to external stimu...       179
## 2  GO:0006511 ubiquitin-dependent protein catabolic pr...       287
## 3  GO:0009060                         aerobic respiration        39
## 4  GO:0009108                coenzyme biosynthetic process        39
## 5  GO:0018022                  peptidyl-lysine methylation        55
## 6  GO:0042559 pteridine-containing compound biosynthet...         8
## 7  GO:0009148 pyrimidine nucleoside triphosphate biosy...         8
## 8  GO:0031347               regulation of defense response       115
## 9  GO:0007623                             circadian rhythm        57
## 10 GO:0006734                       NADH metabolic process        15
## 11 GO:0009894              regulation of catabolic process       306
## 12 GO:0045638 negative regulation of myeloid cell diff...        21
## 13 GO:0043648          dicarboxylic acid metabolic process        46
## 14 GO:0008366                             axon ensheathment        32
## 15 GO:0007272                        ensheathment of neurons        32
##    Significant Expected result1
## 1          115    96.19  0.0025
## 2          177   154.23  0.0032
## 3           29    20.96  0.0066
## 4           29    20.96  0.0066
## 5           39    29.56  0.0068
## 6            8     4.30  0.0069
## 7            8     4.30  0.0069
## 8           75    61.80  0.0077
## 9           40    30.63  0.0082
## 10          13     8.06  0.0082
## 11         185   164.44  0.0087
## 12          17    11.29  0.0092
## 13          33    24.72  0.0095
## 14          24    17.20  0.0111
## 15          24    17.20  0.0111
```

```
# Change in over-dispersion between G1 and S (S+)
goEnrichDoDnotDE12_S_40$BP
```

```
##         GO.ID                                        Term Annotated
## 1  GO:0045777       positive regulation of blood pressure         9
## 2  GO:0022600                    digestive system process        13
## 3  GO:0003073 regulation of systemic arterial blood pr...        14
## 4  GO:0032941                          secretion by tissue        17
## 5  GO:0007589                         body fluid secretion        19
## 6  GO:0010923 negative regulation of phosphatase activ...        23
## 7  GO:0071901 negative regulation of protein serine/th...        40
```

```
## 8  GO:0051048           negative regulation of secretion        43
## 9  GO:0046777                protein autophosphorylation        63
## 10 GO:0044057               regulation of system process        76
## 11 GO:0051241 negative regulation of multicellular org...       215
## 12 GO:0006811                              ion transport       263
## 13 GO:0051239 regulation of multicellular organismal p...       565
## 14 GO:0035556           intracellular signal transduction       611
## 15 GO:0007165                        signal transduction      1033
##    Significant Expected result1
## 1            1     0.00  0.0017
## 2            1     0.00  0.0024
## 3            1     0.00  0.0026
## 4            1     0.00  0.0032
## 5            1     0.00  0.0036
## 6            1     0.00  0.0043
## 7            1     0.01  0.0075
## 8            1     0.01  0.0081
## 9            1     0.01  0.0118
## 10           1     0.01  0.0143
## 11           1     0.04  0.0404
## 12           1     0.05  0.0494
## 13           1     0.11  0.1062
## 14           1     0.11  0.1148
## 15           1     0.19  0.1941
```

```
# Change in over-dispersion between G1 and G2M (G1+)
goEnrichDoDnotDE13_G1_40$BP
```

```
##         GO.ID                                        Term Annotated
## 1  GO:0003081 regulation of systemic arterial blood pr...         6
## 2  GO:0042100                        B cell proliferation        15
## 3  GO:0051928 positive regulation of calcium ion trans...        13
## 4  GO:0002768 immune response-regulating cell surface ...        25
## 5  GO:0060071 Wnt signaling pathway, planar cell polar...         9
## 6  GO:0051497 negative regulation of stress fiber asse...         9
## 7  GO:0090263 positive regulation of canonical Wnt sig...        17
## 8  GO:0048515                    spermatid differentiation        38
## 9  GO:2001237 negative regulation of extrinsic apoptot...        35
## 10 GO:0006342                         chromatin silencing        29
## 11 GO:0002931                        response to ischemia         7
## 12 GO:0052547             regulation of peptidase activity        94
## 13 GO:2000095 regulation of Wnt signaling pathway, pla...         5
## 14 GO:0006415                   translational termination         5
## 15 GO:0046782           regulation of viral transcription        18
##    Significant Expected result1
## 1            6     1.40 0.00016
## 2            9     3.49 0.00245
## 3            8     3.03 0.00347
## 4           12     5.82 0.00579
## 5            6     2.10 0.00684
## 6            6     2.10 0.00684
## 7            9     3.96 0.00752
## 8           16     8.85 0.00755
## 9           15     8.15 0.00790
```

```
## 10          13     6.75 0.00842
## 11           5     1.63 0.00930
## 12          32    21.88 0.01103
## 13           4     1.16 0.01191
## 14           4     1.16 0.01191
## 15           9     4.19 0.01197
```

```
# Change in over-dispersion between G1 and G2M (G2M+)
goEnrichDoDnotDE13_G2M_40$BP
```

```
##         GO.ID                                       Term Annotated
## 1  GO:0006662            glycerol ether metabolic process         5
## 2  GO:0046826 negative regulation of protein export fr...         5
## 3  GO:1903206 negative regulation of hydrogen peroxide...         7
## 4  GO:0033158 regulation of protein import into nucleu...        11
## 5  GO:0043388          positive regulation of DNA binding        15
## 6  GO:0033138 positive regulation of peptidyl-serine p...        21
## 7  GO:0045454                      cell redox homeostasis        34
## 8  GO:0006790            sulfur compound metabolic process        83
## 9  GO:0006457                             protein folding        97
## 10 GO:0009314                       response to radiation       154
## 11 GO:0019725                        cellular homeostasis       177
## 12 GO:0000122 negative regulation of transcription fro...       250
## 13 GO:0009628                 response to abiotic stimulus       272
## 14 GO:0055114                  oxidation-reduction process       327
## 15 GO:0042592                         homeostatic process       380
##    Significant Expected result1
## 1            1     0.00  0.0019
## 2            1     0.00  0.0019
## 3            1     0.00  0.0026
## 4            1     0.00  0.0041
## 5            1     0.01  0.0056
## 6            1     0.01  0.0079
## 7            1     0.01  0.0127
## 8            1     0.03  0.0310
## 9            1     0.04  0.0361
## 10           1     0.06  0.0570
## 11           1     0.07  0.0654
## 12           1     0.09  0.0918
## 13           1     0.10  0.0996
## 14           1     0.12  0.1191
## 15           1     0.14  0.1377
```

```
# Change in over-dispersion between S and G2M (S+)
goEnrichDoDnotDE23_S_40$BP
```

```
##         GO.ID                                       Term Annotated
## 1  GO:0000281                         mitotic cytokinesis        19
## 2  GO:0098813              nuclear chromosome segregation       122
## 3  GO:0051052           regulation of DNA metabolic process       133
## 4  GO:0000280                            nuclear division       313
## 5  GO:1902589       single-organism organelle organization      1055
## 6  GO:0007067                    mitotic nuclear division       255
```

```
## 7  GO:0031060           regulation of histone methylation        34
## 8  GO:0006281                                      DNA repair       258
## 9  GO:0044728          DNA methylation or demethylation        35
## 10 GO:0006304                                DNA modification        37
## 11 GO:0031032           actomyosin structure organization        45
## 12 GO:0033683    nucleotide-excision repair, DNA incision         5
## 13 GO:0034086    maintenance of sister chromatid cohesion         5
## 14 GO:0006390 transcription from mitochondrial promote...         5
## 15 GO:0071971              extracellular exosome assembly         5
##    Significant Expected result1
## 1            2     0.11  0.0056
## 2            4     0.73  0.0058
## 3            4     0.80  0.0078
## 4            6     1.88  0.0097
## 5           12     6.34  0.0153
## 6            5     1.53  0.0169
## 7            2     0.20  0.0174
## 8            5     1.55  0.0177
## 9            2     0.21  0.0184
## 10           2     0.22  0.0205
## 11           2     0.27  0.0295
## 12           1     0.03  0.0297
## 13           1     0.03  0.0297
## 14           1     0.03  0.0297
## 15           1     0.03  0.0297
```

```
# Change in over-dispersion between S and G2M (G2M+)
goEnrichDoDnotDE23_G2M_40$BP
```

```
##         GO.ID                                        Term Annotated
## 1  GO:0006984                 ER-nucleus signaling pathway        17
## 2  GO:0019233                    sensory perception of pain        13
## 3  GO:0042177 negative regulation of protein catabolic...        68
## 4  GO:0051588    regulation of neurotransmitter transport         9
## 5  GO:0018065                      protein-cofactor linkage         5
## 6  GO:0046839               phospholipid dephosphorylation        15
## 7  GO:0042149     cellular response to glucose starvation        15
## 8  GO:0044724 single-organism carbohydrate catabolic p...        42
## 9  GO:1903362 regulation of cellular protein catabolic...       151
## 10 GO:0005977                    glycogen metabolic process        16
## 11 GO:0051186                    cofactor metabolic process       135
## 12 GO:0031330 negative regulation of cellular cataboli...        75
## 13 GO:0043153 entrainment of circadian clock by photop...         6
## 14 GO:0072574                      hepatocyte proliferation         6
## 15 GO:0006283 transcription-coupled nucleotide-excisio...         6
##    Significant Expected result1
## 1            6     1.43  0.0019
## 2            5     1.10  0.0030
## 3           13     5.74  0.0039
## 4            4     0.76  0.0045
## 5            3     0.42  0.0052
## 6            5     1.27  0.0062
## 7            5     1.27  0.0062
## 8            9     3.54  0.0072
```

```
## 9           22    12.74  0.0075
## 10           5     1.35  0.0083
## 11          20    11.39  0.0088
## 12          13     6.33  0.0091
## 13           3     0.51  0.0098
## 14           3     0.51  0.0098
## 15           3     0.51  0.0098
```

Alternative approaches for detecting changes in mean

Preparing the data required to perform alternative analyses.

```
CountsBio12 <- cbind(counts(Data.G1, type = "biological"), counts(Data.S, type = "biological"))
CountsBio13 <- cbind(counts(Data.G1, type = "biological"), counts(Data.G2M, type = "biological"))
CountsBio23 <- cbind(counts(Data.S, type = "biological"), counts(Data.G2M, type = "biological"))

# Required to run SCDE
CountsBio12  <- apply(CountsBio12 , 2, function(x) {storage.mode(x) <- 'integer'; x})
CountsBio13  <- apply(CountsBio13 , 2, function(x) {storage.mode(x) <- 'integer'; x})
CountsBio23  <- apply(CountsBio23 , 2, function(x) {storage.mode(x) <- 'integer'; x})
CountsTech12 <- cbind(counts(Data.G1, type = "technical"),counts(Data.S, type = "technical"))
CountsTech13 <- cbind(counts(Data.G1, type = "technical"),counts(Data.G2M, type = "technical"))
CountsTech23 <- cbind(counts(Data.S, type = "technical"),counts(Data.G2M, type = "technical"))

sg12 <- factor(gsub("(G1|S).*", "\\1", colnames(CountsBio12)), levels = c("G1", "S"))
sg13 <- factor(gsub("(G1|G2M).*", "\\1", colnames(CountsBio13)), levels = c("G1", "G2M"))
sg23 <- factor(gsub("(S|G2M).*", "\\1", colnames(CountsBio23)), levels = c("S", "G2M"))

names(sg12) <- colnames(CountsBio12)
names(sg13) <- colnames(CountsBio13)
names(sg23) <- colnames(CountsBio23)

colData12 = data.frame("Group" = sg12)
colData13 = data.frame("Group" = sg13)
colData23 = data.frame("Group" = sg23)

RPMBio12 = 1000000 * CountsBio12 / colSums(CountsBio12)
RPMBio13 = 1000000 * CountsBio13 / colSums(CountsBio13)
RPMBio23 = 1000000 * CountsBio23 / colSums(CountsBio23)
```

In all cases, analysis was run once using this code and results were stored. This allows faster compilation of the vignette.

DESeq2

```
library(DESeq2)
packageVersion("DESeq2")
```

```
## [1] '1.10.1'
```

```
dds12 <- DESeqDataSetFromMatrix(countData = CountsBio12,
                                colData = colData12,
                                design = ~ Group)
dds13 <- DESeqDataSetFromMatrix(countData = CountsBio13,
                                colData = colData13,
                                design = ~ Group)
dds23 <- DESeqDataSetFromMatrix(countData = CountsBio23,
                                colData = colData23,
                                design = ~ Group)
dds12 <- DESeq(dds12)
#estimating size factors
#estimating dispersions
#gene-wise dispersion estimates
#mean-dispersion relationship
#final dispersion estimates
#fitting model and testing
#-- replacing outliers and refitting for 77 genes
#-- DESeq argument 'minReplicatesForReplace' = 7
#-- original counts are preserved in counts(dds)
#estimating dispersions
#fitting model and testing

dds13 <- DESeq(dds13)
#estimating size factors
#estimating dispersions
#gene-wise dispersion estimates
#mean-dispersion relationship
#final dispersion estimates
#fitting model and testing
#-- replacing outliers and refitting for 82 genes
#-- DESeq argument 'minReplicatesForReplace' = 7
#-- original counts are preserved in counts(dds)
#estimating dispersions
#fitting model and testing

dds23 <- DESeq(dds23)
#estimating size factors
#estimating dispersions
#gene-wise dispersion estimates
#mean-dispersion relationship
#final dispersion estimates
#fitting model and testing
#-- replacing outliers and refitting for 7 genes
#-- DESeq argument 'minReplicatesForReplace' = 7
#-- original counts are preserved in counts(dds)
#estimating dispersions
#fitting model and testing

# tau0 = 0
res12_0 <- results(dds12, contrast = c("Group", "G1", "S"), alpha=0.05)
res13_0 <- results(dds13, contrast = c("Group", "G1", "G2M"), alpha=0.05)
res23_0 <- results(dds23, contrast = c("Group", "S", "G2M"), alpha=0.05)
```

```r
# tau0 = 0.4
res12_40 <- results(dds12, contrast = c("Group", "G1", "S"),
                lfcThreshold=log2(1.5), altHypothesis="greaterAbs", alpha=0.05)
res13_40 <- results(dds13, contrast = c("Group", "G1", "G2M"),
                lfcThreshold=log2(1.5), altHypothesis="greaterAbs", alpha=0.05)
res23_40 <- results(dds23, contrast = c("Group", "S", "G2M"),
                lfcThreshold=log2(1.5), altHypothesis="greaterAbs", alpha=0.05)

write.table(res12_0, file.path(results.path, "DESeq2results12_0.txt"),
            col.names = T, row.names = T)
write.table(res13_0, file.path(results.path, "DESeq2results13_0.txt"),
            col.names = T, row.names = T)
write.table(res23_0, file.path(results.path, "DESeq2results23_0.txt"),
            col.names = T, row.names = T)
write.table(res12_40, file.path(results.path, "DESeq2results12_40.txt"),
            col.names = T, row.names = T)
write.table(res13_40, file.path(results.path, "DESeq2results13_40.txt"),
            col.names = T, row.names = T)
write.table(res23_40, file.path(results.path, "DESeq2results23_40.txt"),
            col.names = T, row.names = T)
```

```r
res12_0 = DESeqResults(DataFrame(read.table(file.path(results.path,
                                                  "DESeq2results12_0.txt"), header = T)))
res13_0 = DESeqResults(DataFrame(read.table(file.path(results.path,
                                                  "DESeq2results13_0.txt"), header = T)))
res23_0 = DESeqResults(DataFrame(read.table(file.path(results.path,
                                                  "DESeq2results23_0.txt"), header = T)))
res12_40 = DESeqResults(DataFrame(read.table(file.path(results.path,
                                                   "DESeq2results12_40.txt"), header = T)))
res13_40 = DESeqResults(DataFrame(read.table(file.path(results.path,
                                                   "DESeq2results13_40.txt"), header = T)))
res23_40 = DESeqResults(DataFrame(read.table(file.path(results.path,
                                                   "DESeq2results23_40.txt"), header = T)))

summary(res12_0, alpha = 0.05)
```

```
##
## out of 5634 with nonzero total read count
## adjusted p-value < 0.05
## LFC > 0 (up)      : 332, 5.9%
## LFC < 0 (down)    : 152, 2.7%
## outliers [1]      : 0, 0%
## low counts [2]    : 0, 0%
## (mean count < 0)
## [1] see 'cooksCutoff' argument of ?results
## [2] see 'independentFiltering' argument of ?results
```

```r
summary(res13_0, alpha = 0.05)
```

```
##
## out of 5634 with nonzero total read count
## adjusted p-value < 0.05
```

```
## LFC > 0 (up)       : 422, 7.5%
## LFC < 0 (down)     : 308, 5.5%
## outliers [1]       : 0, 0%
## low counts [2]     : 0, 0%
## (mean count < 0)
## [1] see 'cooksCutoff' argument of ?results
## [2] see 'independentFiltering' argument of ?results
```

```
summary(res23_0, alpha = 0.05)
```

```
##
## out of 5634 with nonzero total read count
## adjusted p-value < 0.05
## LFC > 0 (up)       : 119, 2.1%
## LFC < 0 (down)     : 186, 3.3%
## outliers [1]       : 0, 0%
## low counts [2]     : 0, 0%
## (mean count < 0)
## [1] see 'cooksCutoff' argument of ?results
## [2] see 'independentFiltering' argument of ?results
```

```
summary(res12_40, alpha = 0.05)
```

```
##
## out of 5634 with nonzero total read count
## adjusted p-value < 0.05
## LFC > 0 (up)       : 11, 0.2%
## LFC < 0 (down)     : 0, 0%
## outliers [1]       : 0, 0%
## low counts [2]     : 0, 0%
## (mean count < 0)
## [1] see 'cooksCutoff' argument of ?results
## [2] see 'independentFiltering' argument of ?results
```

```
summary(res13_40, alpha = 0.05)
```

```
##
## out of 5634 with nonzero total read count
## adjusted p-value < 0.05
## LFC > 0 (up)       : 20, 0.35%
## LFC < 0 (down)     : 13, 0.23%
## outliers [1]       : 0, 0%
## low counts [2]     : 0, 0%
## (mean count < 0)
## [1] see 'cooksCutoff' argument of ?results
## [2] see 'independentFiltering' argument of ?results
```

```
summary(res23_40, alpha = 0.05)
```

```
##
## out of 5634 with nonzero total read count
```

```
## adjusted p-value < 0.05
## LFC > 0 (up)       : 1, 0.018%
## LFC < 0 (down)     : 2, 0.035%
## outliers [1]       : 0, 0%
## low counts [2]     : 0, 0%
## (mean count < 0)
## [1] see 'cooksCutoff' argument of ?results
## [2] see 'independentFiltering' argument of ?results
```

```
ResultsDESeq212_0 = cbind.data.frame("GeneNames" = row.names(res12_0),
                                   "LFC" = res12_0$log2FoldChange * log(2),
                                   "Evidence" = res12_0$padj,
                                   "Result" = ifelse(res12_0$padj < 0.05,
                                                     ifelse(res12_0$log2FoldChange > 0,
                                                            "G1+", "S+"), "NoDiff"))
ResultsDESeq213_0 = cbind.data.frame("GeneNames" = row.names(res13_0),
                                   "LFC" = res13_0$log2FoldChange * log(2),
                                   "Evidence" = res13_0$padj,
                                   "Result" = ifelse(res13_0$padj < 0.05,
                                                     ifelse(res13_0$log2FoldChange > 0,
                                                            "G1+", "G2M+"), "NoDiff"))
ResultsDESeq223_0 = cbind.data.frame("GeneNames" = row.names(res23_0),
                                   "LFC" = res23_0$log2FoldChange * log(2),
                                   "Evidence" = res23_0$padj,
                                   "Result" = ifelse(res23_0$padj < 0.05,
                                                     ifelse(res23_0$log2FoldChange > 0,
                                                            "S+", "G2M+"), "NoDiff"))

ResultsDESeq212_40 = cbind.data.frame("GeneNames" = row.names(res12_40),
                                   "LFC" = res12_40$log2FoldChange * log(2),
                                   "Evidence" = res12_40$padj,
                                   "Result" = ifelse(res12_40$padj < 0.05,
                                                     ifelse(res12_40$log2FoldChange > 0,
                                                            "G1+", "S+"), "NoDiff"))
ResultsDESeq213_40 = cbind.data.frame("GeneNames" = row.names(res13_40),
                                   "LFC" = res13_40$log2FoldChange * log(2),
                                   "Evidence" = res13_40$padj,
                                   "Result" = ifelse(res13_40$padj < 0.05,
                                                     ifelse(res13_40$log2FoldChange > 0,
                                                            "G1+", "G2M+"), "NoDiff"))
ResultsDESeq223_40 = cbind.data.frame("GeneNames" = row.names(res23_40),
                                   "LFC" = res23_40$log2FoldChange * log(2),
                                   "Evidence" = res23_40$padj,
                                   "Result" = ifelse(res23_40$padj < 0.05,
                                                     ifelse(res23_40$log2FoldChange > 0,
                                                            "S+", "G2M+"), "NoDiff"))
```

SCDE

```
library(scde)
packageVersion("scde")
```

```
## [1] '1.99.1'
```

```
o.ifm12 <- scde.error.models(counts = CountsBio12,
                              groups = sg12,
                              n.cores = 3,
                              threshold.segmentation = TRUE,
                              save.crossfit.plots = FALSE,
                              save.model.plots = FALSE,
                              verbose = 1)
o.ifm13 <- scde.error.models(counts = CountsBio13,
                              groups = sg13,
                              n.cores = 3,
                              threshold.segmentation = TRUE,
                              save.crossfit.plots = FALSE,
                              save.model.plots = FALSE,
                              verbose = 1)
o.ifm23 <- scde.error.models(counts = CountsBio23,
                              groups = sg23,
                              n.cores = 3,
                              threshold.segmentation = TRUE,
                              save.crossfit.plots = FALSE,
                              save.model.plots = FALSE,
                              verbose = 1)
write.table(o.ifm12, file.path(results.path, "o.ifm12.txt"), col.names = T, row.names = T)
write.table(o.ifm13, file.path(results.path, "o.ifm13.txt"), col.names = T, row.names = T)
write.table(o.ifm23, file.path(results.path, "o.ifm23.txt"), col.names = T, row.names = T)

o.ifm12 <- read.table(file.path(results.path, "o.ifm12.txt"), header = T)
o.ifm13 <- read.table(file.path(results.path, "o.ifm13.txt"), header = T)
o.ifm23 <- read.table(file.path(results.path, "o.ifm23.txt"), header = T)

# filter out cells that don't show positive correlation with
# the expected expression magnitudes (very poor fits)
valid.cells12 <- o.ifm12$corr.a > 0
valid.cells13 <- o.ifm13$corr.a > 0
valid.cells23 <- o.ifm23$corr.a > 0
table(valid.cells12)
table(valid.cells13)
table(valid.cells23)

o.ifm12 <- o.ifm12[valid.cells12, ]
o.ifm13 <- o.ifm13[valid.cells13, ]
o.ifm23 <- o.ifm23[valid.cells23, ]

# estimate gene expression prior
o.prior12 <- scde.expression.prior(models = o.ifm12, counts = CountsBio12,
                                    length.out = 400, show.plot = FALSE)
o.prior13 <- scde.expression.prior(models = o.ifm13, counts = CountsBio13,
                                    length.out = 400, show.plot = FALSE)
o.prior23 <- scde.expression.prior(models = o.ifm23, counts = CountsBio23,
                                    length.out = 400, show.plot = FALSE)

# define two groups of cells
groups12 <- factor(gsub("(G1|S).*", "\\1", rownames(o.ifm12)), levels = c("G1", "S"))
```

```r
groups13 <- factor(gsub("(G1|G2M).*", "\\1", rownames(o.ifm13)), levels = c("G1", "G2M"))
groups23 <- factor(gsub("(S|G2M).*", "\\1", rownames(o.ifm23)), levels = c("S", "G2M"))
names(groups12) <- row.names(o.ifm12)
names(groups13) <- row.names(o.ifm13)
names(groups23) <- row.names(o.ifm23)
# run differential expression tests on all genes.
ediff12 <- scde.expression.difference(o.ifm12, CountsBio12,
                                    o.prior12, groups = groups12,
                                    n.randomizations  =  100,
                                    n.cores  =  3, verbose  =  1)
ediff13 <- scde.expression.difference(o.ifm13, CountsBio13,
                                    o.prior13, groups = groups13,
                                    n.randomizations  =  100,
                                    n.cores  =  3, verbose  =  1)
ediff23 <- scde.expression.difference(o.ifm23, CountsBio23,
                                    o.prior23, groups = groups23,
                                    n.randomizations  =  100,
                                    n.cores  =  3, verbose  =  1)

write.table(ediff12,
            file = file.path(results.path,"SCDEresults12.txt"),
            row.names = TRUE, col.names = TRUE, sep = "\t", quote = FALSE)
write.table(ediff13,
            file = file.path(results.path,"SCDEresults13.txt"),
            row.names = TRUE, col.names = TRUE, sep = "\t", quote = FALSE)
write.table(ediff23,
            file = file.path(results.path,"SCDEresults23.txt"),
            row.names = TRUE, col.names = TRUE, sep = "\t", quote = FALSE)
```

```r
ediff12 <- read.table(file.path(results.path,"SCDEresults12.txt"), header = T)
ediff13 <- read.table(file.path(results.path,"SCDEresults13.txt"), header = T)
ediff23 <- read.table(file.path(results.path,"SCDEresults23.txt"), header = T)
# adjusted p-value
ediff12$adj.p.value = 2*(1-pnorm(abs(ediff12$cZ)))
ediff13$adj.p.value = 2*(1-pnorm(abs(ediff13$cZ)))
ediff23$adj.p.value = 2*(1-pnorm(abs(ediff23$cZ)))

# Results
table(ediff12$adj.p.value < 0.05)
```

```
##
## FALSE  TRUE
##  5469   165
```

```r
table(ediff13$adj.p.value < 0.05)
```

```
##
## FALSE  TRUE
##  5378   256
```

```
table(ediff23$adj.p.value < 0.05)
```

```
##
## FALSE  TRUE
##  5572    62
```

```
ResultsSCDE12 = cbind.data.frame("GeneNames" = row.names(ediff12),
                                 "LFC" = ediff12$ce * log(2),
                                 "Evidence" = ediff12$adj.p.value,
                                 "Result" = ifelse(ediff12$adj.p.value < 0.05,
                                                   ifelse(ediff12$Z > 0,
                                                          "G1+", "S+"), "NoDiff"))

ResultsSCDE13 = cbind.data.frame("GeneNames" = row.names(ediff13),
                                 "LFC" = ediff13$ce * log(2),
                                 "Evidence" = ediff13$adj.p.value,
                                 "Result" = ifelse(ediff13$adj.p.value < 0.05,
                                                   ifelse(ediff13$Z > 0,
                                                          "G1+", "G2M+"), "NoDiff"))

ResultsSCDE23 = cbind.data.frame("GeneNames" = row.names(ediff23),
                                 "LFC" = ediff23$ce * log(2),
                                 "Evidence" = ediff23$adj.p.value,
                                 "Result" = ifelse(ediff23$adj.p.value < 0.05,
                                                   ifelse(ediff23$Z > 0,
                                                          "S+", "G2M+"), "NoDiff"))
```

edgeR

```
library(edgeR)
packageVersion("edgeR")
```

```
## [1] '3.12.0'
```

```
cds12 <- DGEList( CountsBio12 , group = colData12$Group )
cds13 <- DGEList( CountsBio13 , group = colData13$Group )
cds23 <- DGEList( CountsBio23 , group = colData23$Group )

cds12 <- calcNormFactors( cds12 )
cds13 <- calcNormFactors( cds13 )
cds23 <- calcNormFactors( cds23 )

design12 = model.matrix(~colData12$Group)
design13 = model.matrix(~colData13$Group)
design23 = model.matrix(~colData23$Group)

cds12 <- estimateGLMCommonDisp( cds12, design12, verbose = TRUE )
cds13 <- estimateGLMCommonDisp( cds13, design13, verbose = TRUE )
cds23 <- estimateGLMCommonDisp( cds23, design23, verbose = TRUE )
```

```r
# This is the recommendation, it doesn't work because we have too many samples
# Instead we use prior.df = 10 as in edgeR vignette, batch effect example
#cds <- estimateTagwiseDisp( cds , prior.n = 50 / (ncol(CountsBio) - 2) )
cds12 <- estimateGLMTrendedDisp(cds12, design12)
cds13 <- estimateGLMTrendedDisp(cds13, design13)
cds23 <- estimateGLMTrendedDisp(cds23, design23)

cds12 <- estimateGLMTagwiseDisp(cds12, design12, prior.df=10)
cds13 <- estimateGLMTagwiseDisp(cds13, design13, prior.df=10)
cds23 <- estimateGLMTagwiseDisp(cds23, design23, prior.df=10)

fit12 <- glmFit(cds12, design12)
fit13 <- glmFit(cds13, design13)
fit23 <- glmFit(cds23, design23)

# Using different test because of message: Zero log2-FC threshold detected.
# Switch to glmLRT() instead
tr12_0 = glmLRT(fit12, coef = 2)
tr13_0 = glmLRT(fit13, coef = 2)
tr23_0 = glmLRT(fit23, coef = 2)
tr12_40 <- glmTreat(fit12, coef = 2, lfc = log2(1.5))
tr13_40 <- glmTreat(fit13, coef = 2, lfc = log2(1.5))
tr23_40 <- glmTreat(fit23, coef = 2, lfc = log2(1.5))

edgeR12_0 = topTags(tr12_0, n = Inf, adjust.method = "BH")$table
edgeR13_0 = topTags(tr13_0, n = Inf, adjust.method = "BH")$table
edgeR23_0 = topTags(tr23_0, n = Inf, adjust.method = "BH")$table
edgeR12_40 = topTags(tr12_40, n = Inf, adjust.method = "BH")$table
edgeR13_40 = topTags(tr13_40, n = Inf, adjust.method = "BH")$table
edgeR23_40 = topTags(tr23_40, n = Inf, adjust.method = "BH")$table

#summary(de12_0 <- decideTestsDGE(tr12_0), adjust.method="BH", p.value=0.05, lfc=0)
#summary(de40 <- decideTestsDGE(tr40), adjust.method="BH", p.value=0.05, lfc=log2(1.5))

write.table(edgeR12_0, file.path(results.path, "edgeR12_0.txt"), col.names = T, row.names = T)
write.table(edgeR13_0, file.path(results.path, "edgeR13_0.txt"), col.names = T, row.names = T)
write.table(edgeR23_0, file.path(results.path, "edgeR23_0.txt"), col.names = T, row.names = T)
write.table(edgeR12_40, file.path(results.path, "edgeR12_40.txt"), col.names = T, row.names = T)
write.table(edgeR13_40, file.path(results.path, "edgeR13_40.txt"), col.names = T, row.names = T)
write.table(edgeR23_40, file.path(results.path, "edgeR23_40.txt"), col.names = T, row.names = T)
```

```r
edgeR12_0 = read.table(file.path(results.path, "edgeR12_0.txt"))
edgeR13_0 = read.table(file.path(results.path, "edgeR13_0.txt"))
edgeR23_0 = read.table(file.path(results.path, "edgeR23_0.txt"))
edgeR12_40 = read.table(file.path(results.path, "edgeR12_40.txt"))
edgeR13_40 = read.table(file.path(results.path, "edgeR13_40.txt"))
edgeR23_40 = read.table(file.path(results.path, "edgeR23_40.txt"))


ResultsedgeR12_0 = cbind.data.frame("GeneNames" = row.names(edgeR12_0),
                                   "LFC" = edgeR12_0$logFC * log(2),
                                   "Evidence" = edgeR12_0$FDR,
                                   "Result" = ifelse(edgeR12_0$FDR < 0.05,
```

```
                                                     ifelse(edgeR12_0$logFC > 0,
                                                            "G1+", "S+"), "NoDiff"))

ResultsedgeR12_40 = cbind.data.frame("GeneNames" = row.names(edgeR12_40),
                                   "LFC" = edgeR12_40$logFC * log(2),
                                   "Evidence" = edgeR12_40$FDR,
                                   "Result" = ifelse(edgeR12_40$FDR < 0.05,
                                                     ifelse(edgeR12_40$logFC > 0,
                                                            "G1+", "S+"), "NoDiff"))

ResultsedgeR13_0 = cbind.data.frame("GeneNames" = row.names(edgeR13_0),
                                   "LFC" = edgeR13_0$logFC * log(2),
                                   "Evidence" = edgeR13_0$FDR,
                                   "Result" = ifelse(edgeR13_0$FDR < 0.05,
                                                     ifelse(edgeR13_0$logFC > 0,
                                                            "G1+", "G2M+"), "NoDiff"))

ResultsedgeR13_40 = cbind.data.frame("GeneNames" = row.names(edgeR13_40),
                                   "LFC" = edgeR13_40$logFC * log(2),
                                   "Evidence" = edgeR13_40$FDR,
                                   "Result" = ifelse(edgeR13_40$FDR < 0.05,
                                                     ifelse(edgeR13_40$logFC > 0,
                                                            "G1+", "G2M+"), "NoDiff"))

ResultsedgeR23_0 = cbind.data.frame("GeneNames" = row.names(edgeR23_0),
                                   "LFC" = edgeR23_0$logFC * log(2),
                                   "Evidence" = edgeR23_0$FDR,
                                   "Result" = ifelse(edgeR23_0$FDR < 0.05,
                                                     ifelse(edgeR23_0$logFC > 0,
                                                            "S+", "G2M+"), "NoDiff"))

ResultsedgeR23_40 = cbind.data.frame("GeneNames" = row.names(edgeR23_40),
                                   "LFC" = edgeR23_40$logFC * log(2),
                                   "Evidence" = edgeR23_40$FDR,
                                   "Result" = ifelse(edgeR23_40$FDR < 0.05,
                                                     ifelse(edgeR23_40$logFC > 0,
                                                            "S+", "G2M+"), "NoDiff"))
```

MAST

```
#library(devtools)
#install_github("RGLab/MAST")
library(MAST)
packageVersion("MAST")
```

```
## [1] '0.933'
```

```
ngeneson12 <- apply(t(log(RPMBio12+1)),1,function(x)mean(x>0))
ngeneson13 <- apply(t(log(RPMBio13+1)),1,function(x)mean(x>0))
ngeneson23 <- apply(t(log(RPMBio23+1)),1,function(x)mean(x>0))
```

```r
dataMAST12 <- FromMatrix('SingleCellAssay', t(log(RPMBio12+1)),
                       fData = data.frame("primerid" = rownames(CountsBio12)),
                       cData = data.frame("wellKey" = colnames(CountsBio12),
                                          "Population" = colData12$Group,
                                          "ngeneson" = ngeneson12,
                                          "cngeneson" = ngeneson12 - mean(ngeneson12)))

dataMAST13 <- FromMatrix('SingleCellAssay', t(log(RPMBio13+1)),
                       fData = data.frame("primerid" = rownames(CountsBio13)),
                       cData = data.frame("wellKey" = colnames(CountsBio13),
                                          "Population" = colData13$Group,
                                          "ngeneson" = ngeneson13,
                                          "cngeneson" = ngeneson13 - mean(ngeneson13)))

dataMAST23 <- FromMatrix('SingleCellAssay', t(log(RPMBio23+1)),
                       fData = data.frame("primerid" = rownames(CountsBio23)),
                       cData = data.frame("wellKey" = colnames(CountsBio23),
                                          "Population" = colData23$Group,
                                          "ngeneson" = ngeneson23,
                                          "cngeneson" = ngeneson23 - mean(ngeneson23)))

zlm.output12 <- zlm.SingleCellAssay(~ Population + cngeneson, dataMAST12,
                                  method='bayesglm',
                                  ebayes = TRUE,
                                  ebayesControl = list(method = "MLE", model = "H1"))
zlm.output13 <- zlm.SingleCellAssay(~ Population + cngeneson, dataMAST13,
                                  method='bayesglm',
                                  ebayes = TRUE,
                                  ebayesControl = list(method = "MLE", model = "H1"))
zlm.output23 <- zlm.SingleCellAssay(~ Population + cngeneson, dataMAST23,
                                  method='bayesglm',
                                  ebayes = TRUE,
                                  ebayesControl = list(method = "MLE", model = "H1"))

zlm.lr12 <- lrTest(zlm.output12, 'Population')
zlm.lr13 <- lrTest(zlm.output13, 'Population')
zlm.lr23 <- lrTest(zlm.output23, 'Population')

# BH correction
adj.p.cont12 = p.adjust(zlm.lr12[,'cont','Pr(>Chisq)'], "BH")
adj.p.disc12 = p.adjust(zlm.lr12[,'disc','Pr(>Chisq)'], "BH")
adj.p.hurdle12 = p.adjust(zlm.lr12[,'hurdle','Pr(>Chisq)'], "BH")
adj.p.cont13 = p.adjust(zlm.lr13[,'cont','Pr(>Chisq)'], "BH")
adj.p.disc13 = p.adjust(zlm.lr13[,'disc','Pr(>Chisq)'], "BH")
adj.p.hurdle13 = p.adjust(zlm.lr13[,'hurdle','Pr(>Chisq)'], "BH")
adj.p.cont23 = p.adjust(zlm.lr23[,'cont','Pr(>Chisq)'], "BH")
adj.p.disc23 = p.adjust(zlm.lr23[,'disc','Pr(>Chisq)'], "BH")
adj.p.hurdle23 = p.adjust(zlm.lr23[,'hurdle','Pr(>Chisq)'], "BH")

write.table(adj.p.cont12, file.path(results.path, "adj.p.cont12.txt"),
            row.names = T, col.names = T)
write.table(adj.p.disc12, file.path(results.path, "adj.p.disc12.txt"),
            row.names = T, col.names = T)
```

```r
write.table(adj.p.hurdle12, file.path(results.path, "adj.p.hurdle12.txt"),
            row.names = T, col.names = T)

write.table(adj.p.cont13, file.path(results.path, "adj.p.cont13.txt"),
            row.names = T, col.names = T)
write.table(adj.p.disc13, file.path(results.path, "adj.p.disc13.txt"),
            row.names = T, col.names = T)
write.table(adj.p.hurdle13, file.path(results.path, "adj.p.hurdle13.txt"),
            row.names = T, col.names = T)

write.table(adj.p.cont23, file.path(results.path, "adj.p.cont23.txt"),
            row.names = T, col.names = T)
write.table(adj.p.disc23, file.path(results.path, "adj.p.disc23.txt"),
            row.names = T, col.names = T)
write.table(adj.p.hurdle23, file.path(results.path, "adj.p.hurdle23.txt"),
            row.names = T, col.names = T)
```

```r
adj.p.cont12 = read.table(file.path(results.path, "adj.p.cont12.txt"))
adj.p.disc12 = read.table(file.path(results.path, "adj.p.disc12.txt"))
adj.p.hurdle12 = read.table(file.path(results.path, "adj.p.hurdle12.txt"))
adj.p.cont13 = read.table(file.path(results.path, "adj.p.cont13.txt"))
adj.p.disc13 = read.table(file.path(results.path, "adj.p.disc13.txt"))
adj.p.hurdle13 = read.table(file.path(results.path, "adj.p.hurdle13.txt"))
adj.p.cont23 = read.table(file.path(results.path, "adj.p.cont23.txt"))
adj.p.disc23 = read.table(file.path(results.path, "adj.p.disc23.txt"))
adj.p.hurdle23 = read.table(file.path(results.path, "adj.p.hurdle23.txt"))

table(adj.p.cont12 < 0.05)
```

```
##
## FALSE  TRUE
##  5575    59
```

```r
table(adj.p.disc12 < 0.05)
```

```
##
## FALSE  TRUE
##  5624    10
```

```r
table(adj.p.hurdle12 < 0.05)
```

```
##
## FALSE  TRUE
##  5594    40
```

```r
table(adj.p.cont13 < 0.05)
```

```
##
## FALSE  TRUE
##  5422   212
```

```r
table(adj.p.disc13 < 0.05)
```

```
##
## FALSE  TRUE
##  5580    54
```

```r
table(adj.p.hurdle13 < 0.05)
```

```
##
## FALSE  TRUE
##  5386   248
```

```r
table(adj.p.cont23 < 0.05)
```

```
##
## FALSE  TRUE
##  5514   120
```

```r
table(adj.p.disc23 < 0.05)
```

```
##
## FALSE  TRUE
##  5598    36
```

```r
table(adj.p.hurdle23 < 0.05)
```

```
##
## FALSE  TRUE
##  5497   137
```

```r
ResultsMAST12 = cbind.data.frame("GeneNames" = row.names(adj.p.cont12),
                           "EvidenceCont" = adj.p.cont12$x,
                           "EvidenceDisc" = adj.p.disc12$x,
                           "EvidenceHurdle" = adj.p.hurdle12$x,
                           "ResultsCont" = ifelse(adj.p.cont12$x < 0.05, "Diff", "NoDiff"),
                           "ResultsDisc" = ifelse(adj.p.disc12$x < 0.05, "Diff", "NoDiff"),
                           "ResultsHurdle" = ifelse(adj.p.hurdle12$x < 0.05, "Diff", "NoDiff"))
ResultsMAST13 = cbind.data.frame("GeneNames" = row.names(adj.p.cont13),
                           "EvidenceCont" = adj.p.cont13$x,
                           "EvidenceDisc" = adj.p.disc13$x,
                           "EvidenceHurdle" = adj.p.hurdle13$x,
                           "ResultsCont" = ifelse(adj.p.cont13$x < 0.05, "Diff", "NoDiff"),
                           "ResultsDisc" = ifelse(adj.p.disc13$x < 0.05, "Diff", "NoDiff"),
                           "ResultsHurdle" = ifelse(adj.p.hurdle13$x < 0.05, "Diff", "NoDiff"))
ResultsMAST23 = cbind.data.frame("GeneNames" = row.names(adj.p.cont23),
                           "EvidenceCont" = adj.p.cont23$x,
                           "EvidenceDisc" = adj.p.disc23$x,
                           "EvidenceHurdle" = adj.p.hurdle23$x,
                           "ResultsCont" = ifelse(adj.p.cont23$x < 0.05, "Diff", "NoDiff"),
                           "ResultsDisc" = ifelse(adj.p.disc23$x < 0.05, "Diff", "NoDiff"),
                           "ResultsHurdle" = ifelse(adj.p.hurdle23$x < 0.05, "Diff", "NoDiff"))
```

```
zlm.outputNotCDR12 <- zlm.SingleCellAssay(~ Population, dataMAST12,
                                  method='bayesglm',
                                  ebayes = TRUE,
                                  ebayesControl = list(method = "MLE", model = "H1"))
zlm.outputNotCDR13 <- zlm.SingleCellAssay(~ Population, dataMAST13,
                                  method='bayesglm',
                                  ebayes = TRUE,
                                  ebayesControl = list(method = "MLE", model = "H1"))
zlm.outputNotCDR23 <- zlm.SingleCellAssay(~ Population, dataMAST23,
                                  method='bayesglm',
                                  ebayes = TRUE,
                                  ebayesControl = list(method = "MLE", model = "H1"))

zlm.lrNotCDR12 <- lrTest(zlm.outputNotCDR12, 'Population')
zlm.lrNotCDR13 <- lrTest(zlm.outputNotCDR13, 'Population')
zlm.lrNotCDR23 <- lrTest(zlm.outputNotCDR23, 'Population')

# BH correction
adj.p.contNotCDR12 = p.adjust(zlm.lrNotCDR12[,'cont','Pr(>Chisq)'], "BH")
adj.p.discNotCDR12 = p.adjust(zlm.lrNotCDR12[,'disc','Pr(>Chisq)'], "BH")
adj.p.hurdleNotCDR12 = p.adjust(zlm.lrNotCDR12[,'hurdle','Pr(>Chisq)'], "BH")
adj.p.contNotCDR13 = p.adjust(zlm.lrNotCDR13[,'cont','Pr(>Chisq)'], "BH")
adj.p.discNotCDR13 = p.adjust(zlm.lrNotCDR13[,'disc','Pr(>Chisq)'], "BH")
adj.p.hurdleNotCDR13 = p.adjust(zlm.lrNotCDR13[,'hurdle','Pr(>Chisq)'], "BH")
adj.p.contNotCDR23 = p.adjust(zlm.lrNotCDR23[,'cont','Pr(>Chisq)'], "BH")
adj.p.discNotCDR23 = p.adjust(zlm.lrNotCDR23[,'disc','Pr(>Chisq)'], "BH")
adj.p.hurdleNotCDR23 = p.adjust(zlm.lrNotCDR23[,'hurdle','Pr(>Chisq)'], "BH")

write.table(adj.p.contNotCDR12, file.path(results.path, "adj.p.contNotCDR12.txt"),
            row.names = T, col.names = T)
write.table(adj.p.discNotCDR12, file.path(results.path, "adj.p.discNotCDR12.txt"),
            row.names = T, col.names = T)
write.table(adj.p.hurdleNotCDR12, file.path(results.path, "adj.p.hurdleNotCDR12.txt"),
            row.names = T, col.names = T)

write.table(adj.p.contNotCDR13, file.path(results.path, "adj.p.contNotCDR13.txt"),
            row.names = T, col.names = T)
write.table(adj.p.discNotCDR13, file.path(results.path, "adj.p.discNotCDR13.txt"),
            row.names = T, col.names = T)
write.table(adj.p.hurdleNotCDR13, file.path(results.path, "adj.p.hurdleNotCDR13.txt"),
            row.names = T, col.names = T)

write.table(adj.p.contNotCDR23, file.path(results.path, "adj.p.contNotCDR23.txt"),
            row.names = T, col.names = T)
write.table(adj.p.discNotCDR23, file.path(results.path, "adj.p.discNotCDR23.txt"),
            row.names = T, col.names = T)
write.table(adj.p.hurdleNotCDR23, file.path(results.path, "adj.p.hurdleNotCDR23.txt"),
            row.names = T, col.names = T)
```

```
adj.p.contNotCDR12 = read.table(file.path(results.path, "adj.p.contNotCDR12.txt"))
adj.p.discNotCDR12 = read.table(file.path(results.path, "adj.p.discNotCDR12.txt"))
adj.p.hurdleNotCDR12 = read.table(file.path(results.path, "adj.p.hurdleNotCDR12.txt"))
adj.p.contNotCDR13 = read.table(file.path(results.path, "adj.p.contNotCDR13.txt"))
```

```r
adj.p.discNotCDR13 = read.table(file.path(results.path, "adj.p.discNotCDR13.txt"))
adj.p.hurdleNotCDR13 = read.table(file.path(results.path, "adj.p.hurdleNotCDR13.txt"))
adj.p.contNotCDR23 = read.table(file.path(results.path, "adj.p.contNotCDR23.txt"))
adj.p.discNotCDR23 = read.table(file.path(results.path, "adj.p.discNotCDR23.txt"))
adj.p.hurdleNotCDR23 = read.table(file.path(results.path, "adj.p.hurdleNotCDR23.txt"))

table(adj.p.contNotCDR12 < 0.05)
```

```
##
## FALSE  TRUE
##  3278  2356
```

```r
table(adj.p.discNotCDR12 < 0.05)
```

```
##
## FALSE  TRUE
##  4895   739
```

```r
table(adj.p.hurdleNotCDR12 < 0.05)
```

```
##
## FALSE  TRUE
##  2934  2700
```

```r
table(adj.p.contNotCDR13 < 0.05)
```

```
##
## FALSE  TRUE
##  4314  1320
```

```r
table(adj.p.discNotCDR13 < 0.05)
```

```
##
## FALSE  TRUE
##  5165   469
```

```r
table(adj.p.hurdleNotCDR13 < 0.05)
```

```
##
## FALSE  TRUE
##  4011  1623
```

```r
table(adj.p.contNotCDR23 < 0.05)
```

```
##
## FALSE  TRUE
##  5445   189
```

```r
table(adj.p.discNotCDR23 < 0.05)
```

```
##
## FALSE  TRUE
##  5585    49
```

```r
table(adj.p.hurdleNotCDR23 < 0.05)
```

```
##
## FALSE  TRUE
##  5378   256
```

```r
ResultsMASTNotCDR12 = cbind.data.frame("GeneNames" = row.names(adj.p.contNotCDR12),
                            "EvidenceCont" = adj.p.contNotCDR12$x,
                            "EvidenceDisc" = adj.p.discNotCDR12$x,
                            "EvidenceHurdle" = adj.p.hurdleNotCDR12$x,
                            "ResultsCont" = ifelse(adj.p.contNotCDR12$x < 0.05, "Diff", "NoDiff"),
                            "ResultsDisc" = ifelse(adj.p.discNotCDR12$x < 0.05, "Diff", "NoDiff"),
                            "ResultsHurdle" = ifelse(adj.p.hurdleNotCDR12$x < 0.05, "Diff", "NoDiff"))
ResultsMASTNotCDR13 = cbind.data.frame("GeneNames" = row.names(adj.p.contNotCDR13),
                            "EvidenceCont" = adj.p.contNotCDR13$x,
                            "EvidenceDisc" = adj.p.discNotCDR13$x,
                            "EvidenceHurdle" = adj.p.hurdleNotCDR13$x,
                            "ResultsCont" = ifelse(adj.p.contNotCDR13$x < 0.05, "Diff", "NoDiff"),
                            "ResultsDisc" = ifelse(adj.p.discNotCDR13$x < 0.05, "Diff", "NoDiff"),
                            "ResultsHurdle" = ifelse(adj.p.hurdleNotCDR13$x < 0.05, "Diff", "NoDiff"))
ResultsMASTNotCDR23 = cbind.data.frame("GeneNames" = row.names(adj.p.contNotCDR23),
                            "EvidenceCont" = adj.p.contNotCDR23$x,
                            "EvidenceDisc" = adj.p.discNotCDR23$x,
                            "EvidenceHurdle" = adj.p.hurdleNotCDR23$x,
                            "ResultsCont" = ifelse(adj.p.contNotCDR23$x < 0.05, "Diff", "NoDiff"),
                            "ResultsDisc" = ifelse(adj.p.discNotCDR23$x < 0.05, "Diff", "NoDiff"),
                            "ResultsHurdle" = ifelse(adj.p.hurdleNotCDR23$x < 0.05, "Diff", "NoDiff"))
```

```r
GenesDE12 = c(sum(Test12_0$Table$ResultDiffExp != "NoDiff"), # 1
                sum(Test12_40$Table$ResultDiffExp != "NoDiff"), # 2
                sum(ResultsDESeq212_0$Result != "NoDiff"), # 3
                sum(ResultsDESeq212_40$Result != "NoDiff"), # 4
                sum(ResultsedgeR12_0$Result != "NoDiff"), # 5
                sum(ResultsedgeR12_40$Result != "NoDiff"), # 6
                sum(ResultsSCDE12$Result != "NoDiff"), # 7
                sum(ResultsMAST12$ResultsHurdle != "NoDiff"), # 8
                sum(ResultsMASTNotCDR12$ResultsHurdle != "NoDiff")) # 9

GenesDE13 = c(sum(Test13_0$Table$ResultDiffExp != "NoDiff"), # 1
                sum(Test13_40$Table$ResultDiffExp != "NoDiff"), # 2
                sum(ResultsDESeq213_0$Result != "NoDiff"), # 3
                sum(ResultsDESeq213_40$Result != "NoDiff"), # 4
                sum(ResultsedgeR13_0$Result != "NoDiff"), # 5
                sum(ResultsedgeR13_40$Result != "NoDiff"), # 6
                sum(ResultsSCDE13$Result != "NoDiff"), # 7
                sum(ResultsMAST13$ResultsHurdle != "NoDiff"), # 8
```

```
                sum(ResultsMASTNotCDR13$ResultsHurdle != "NoDiff")) # 9

GenesDE23 = c(sum(Test23_0$Table$ResultDiffExp != "NoDiff"), # 1
                sum(Test23_40$Table$ResultDiffExp != "NoDiff"), # 2
                sum(ResultsDESeq223_0$Result != "NoDiff"), # 3
                sum(ResultsDESeq223_40$Result != "NoDiff"), # 4
                sum(ResultsedgeR23_0$Result != "NoDiff"), # 5
                sum(ResultsedgeR23_40$Result != "NoDiff"), # 6
                sum(ResultsSCDE23$Result != "NoDiff"), # 7
                sum(ResultsMAST23$ResultsHurdle != "NoDiff"), # 8
                sum(ResultsMASTNotCDR23$ResultsHurdle != "NoDiff")) # 9

rainbowcols <- rainbow(9, s = 0.3)

bplt <- barplot(GenesDE12, col = rainbowcols, names.arg = 1:length(GenesDE12), ylim = c(0, 5700),
                ylab = "No. of detected genes", xlab = "Method", cex.lab = 1.5,
                main = "G1 vs S", cex.main = 2)
text(x = bplt, y = GenesDE12 + 250, GenesDE12, cex = 1.5)
```

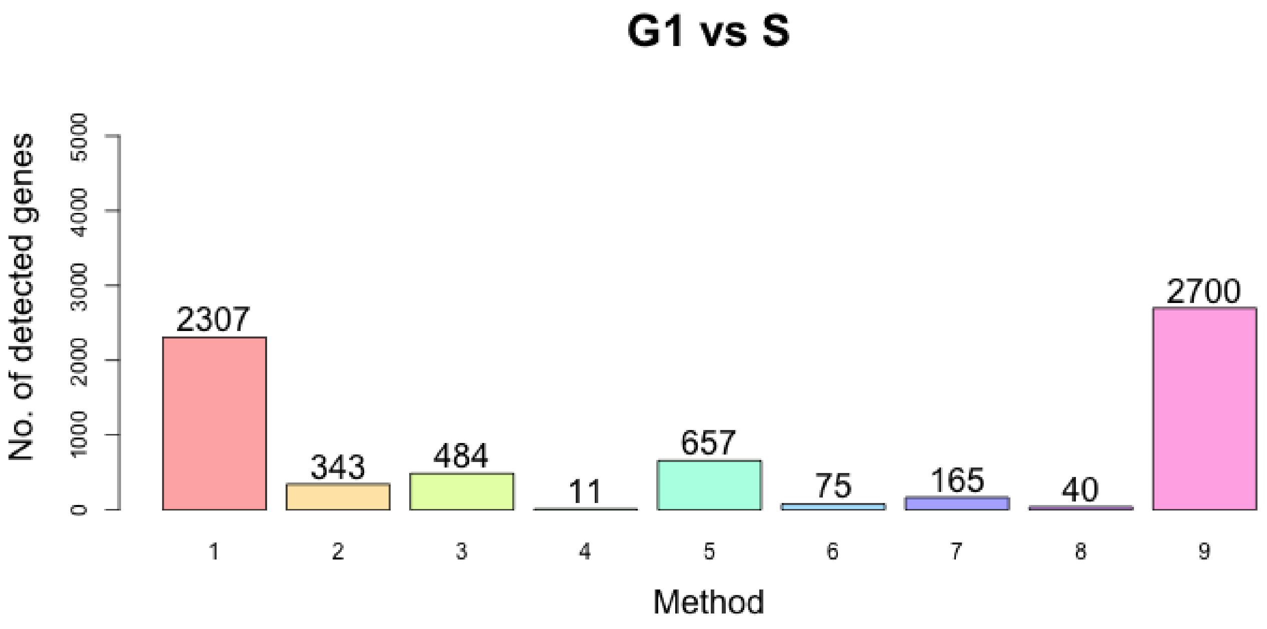


```
bplt <- barplot(GenesDE13, col = rainbowcols, names.arg = 1:length(GenesDE13), ylim = c(0, 5700),
                ylab = "No. of detected genes", xlab = "Method", cex.lab = 1.5,
                main = "G1 vs G2M", cex.main = 2)
text(x = bplt, y = GenesDE13 + 250, GenesDE13, cex = 1.5)
```

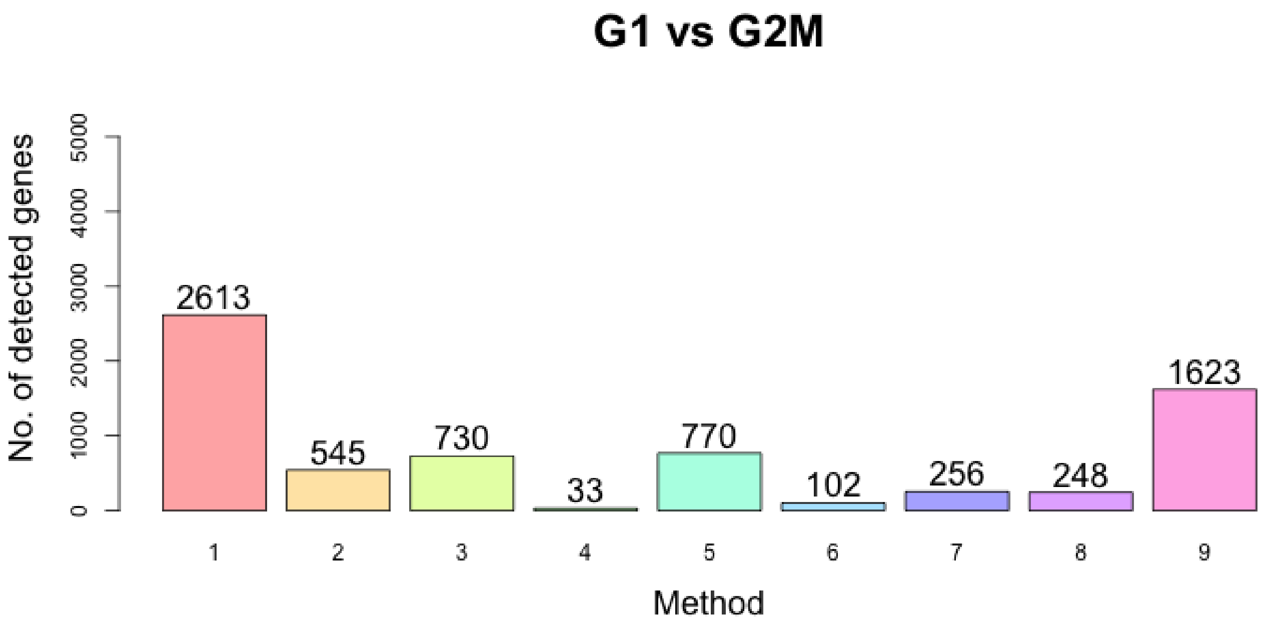


```
bplt <- barplot(GenesDE23, col = rainbowcols, names.arg = 1:length(GenesDE23), ylim = c(0, 5700),
                ylab = "No. of detected genes", xlab = "Method", cex.lab = 1.5,
                main = "S vs G2M", cex.main = 2)
text(x = bplt, y = GenesDE23 + 250, GenesDE23, cex = 1.5)
```

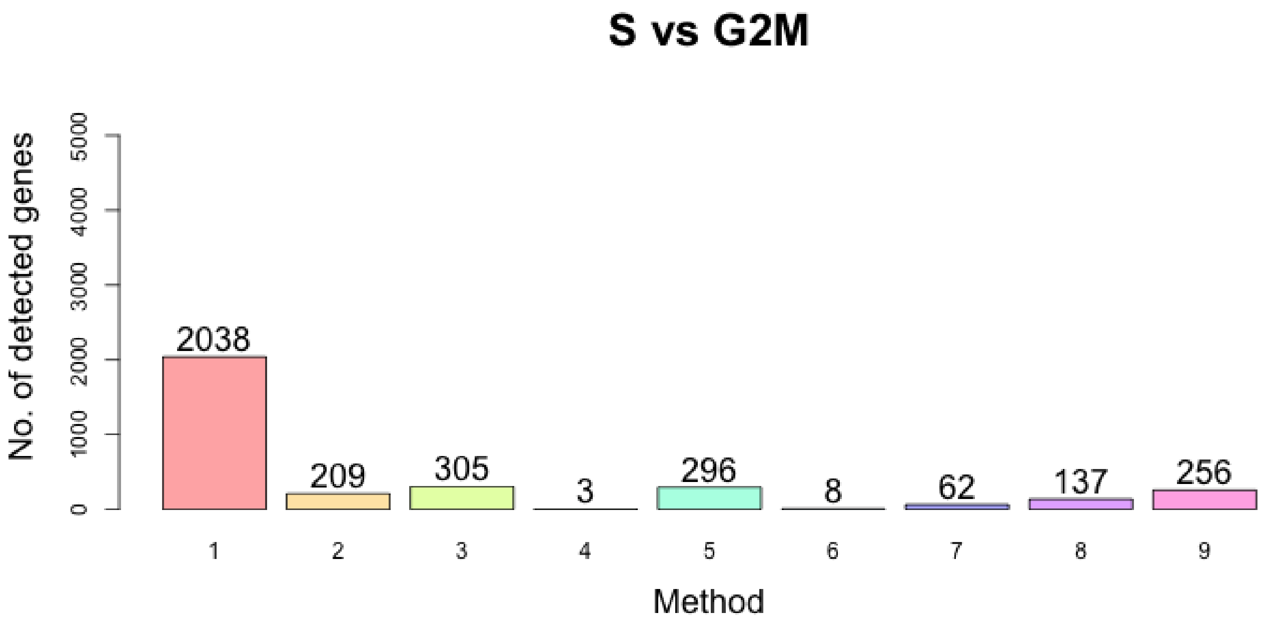

To be more fair, since the definition of mean is different, we consider results regarding the Hurdle model for MAST. The following shows GO enrichment analysis for the genes highlighted to have differences in overall expression according to BASiCS but not by MAST or SCDE.

```
Test12_40_DT_MAST = merge(Test12_40_DT, ResultsMAST12, by = "GeneNames")
Test13_40_DT_MAST = merge(Test13_40_DT, ResultsMAST13, by = "GeneNames")
Test23_40_DT_MAST = merge(Test23_40_DT, ResultsMAST12, by = "GeneNames")

# 317 genes
```

```r
goEnrichDE12_40_MAST<-topGOAnalysis(Test12_40_DT_MAST$AssociatedGeneName[!is.na(Test12_40_DT_MAST$Assoc
                              rep(T,sum(!is.na(Test12_40_DT_MAST$AssociatedGeneName))),
                              Test12_40_DT_MAST$ResultDiffExp != "NoDiff" &
                                Test12_40_DT_MAST$ResultsHurdle == "NoDiff")

# 371 genes
goEnrichDE13_40_MAST<-topGOAnalysis(Test13_40_DT_MAST$AssociatedGeneName[!is.na(Test13_40_DT_MAST$Assoc
                              rep(T,sum(!is.na(Test13_40_DT_MAST$AssociatedGeneName))),
                              Test13_40_DT_MAST$ResultDiffExp != "NoDiff" &
                                Test13_40_DT_MAST$ResultsHurdle == "NoDiff")

# 206 genes
goEnrichDE23_40_MAST<-topGOAnalysis(Test23_40_DT_MAST$AssociatedGeneName[!is.na(Test23_40_DT_MAST$Assoc
                              rep(T,sum(!is.na(Test23_40_DT_MAST$AssociatedGeneName))),
                              Test23_40_DT_MAST$ResultDiffExp != "NoDiff" &
                                Test23_40_DT_MAST$ResultsHurdle == "NoDiff")
```

```
##         GO.ID                                        Term Annotated
## 1  GO:0070527                       platelet aggregation        19
## 2  GO:0042772 DNA damage response, signal transduction...         5
## 3  GO:0021522    spinal cord motor neuron differentiation         6
## 4  GO:0050881                   musculoskeletal movement         6
## 5  GO:0006878              cellular copper ion homeostasis         6
## 6  GO:0070306              lens fiber cell differentiation         6
## 7  GO:0050885 neuromuscular process controlling balanc...        20
## 8  GO:0042592                        homeostatic process       380
## 9  GO:0006814                        sodium ion transport        32
## 10 GO:0015936                 coenzyme A metabolic process         8
## 11 GO:2001238 positive regulation of extrinsic apoptot...        18
## 12 GO:0030193              regulation of blood coagulation        11
## 13 GO:0000920            cell separation after cytokinesis        11
## 14 GO:1900046                   regulation of hemostasis        11
## 15 GO:0043967                     histone H4 acetylation        40
##    Significant Expected result1
## 1            7     1.04 3.9e-05
## 2            3     0.27  0.0015
## 3            3     0.33  0.0029
## 4            3     0.33  0.0029
## 5            3     0.33  0.0029
## 6            3     0.33  0.0029
## 7            5     1.09  0.0037
## 8           35    20.78  0.0059
## 9            6     1.75  0.0069
## 10           3     0.44  0.0074
## 11           4     0.98  0.0146
## 12           3     0.60  0.0192
## 13           3     0.60  0.0192
## 14           3     0.60  0.0192
## 15           6     2.19  0.0202

##         GO.ID                                        Term Annotated
## 1  GO:0007052                 mitotic spindle organization        45
## 2  GO:1903792      negative regulation of anion transport         5
```

```
## 3  GO:0051231                        spindle elongation         5
## 4  GO:0021695             cerebellar cortex development        17
## 5  GO:0070841                   inclusion body assembly        12
## 6  GO:0046717                            acid secretion        12
## 7  GO:0070527                      platelet aggregation        19
## 8  GO:0006310                         DNA recombination       114
## 9  GO:0061041               regulation of wound healing        20
## 10 GO:0051646                mitochondrion localization        13
## 11 GO:0045005 DNA-dependent DNA replication maintenanc...        13
## 12 GO:0045132             meiotic chromosome segregation        39
## 13 GO:0002712      regulation of B cell mediated immunity         8
## 14 GO:0002889 regulation of immunoglobulin mediated im...         8
## 15 GO:0032890        regulation of organic acid transport         8
##    Significant Expected result1
## 1           11     2.95 0.00011
## 2            3     0.33 0.00253
## 3            3     0.33 0.00253
## 4            5     1.11 0.00378
## 5            4     0.79 0.00590
## 6            4     0.79 0.00590
## 7            5     1.25 0.00637
## 8           15     7.48 0.00709
## 9            5     1.31 0.00804
## 10           4     0.85 0.00809
## 11           4     0.85 0.00809
## 12           7     2.56 0.01218
## 13           3     0.52 0.01222
## 14           3     0.52 0.01222
## 15           3     0.52 0.01222
```

```
##         GO.ID                                        Term Annotated
## 1  GO:0051340               regulation of ligase activity        45
## 2  GO:0051438 regulation of ubiquitin-protein transfer...        46
## 3  GO:0043968                     histone H2A acetylation        12
## 4  GO:0030071 regulation of mitotic metaphase/anaphase...        37
## 5  GO:0045682          regulation of epidermis development        13
## 6  GO:0045471                         response to ethanol        13
## 7  GO:0031145 anaphase-promoting complex-dependent pro...        42
## 8  GO:0015711                     organic anion transport        70
## 9  GO:0071715                         icosanoid transport         5
## 10 GO:1901571              fatty acid derivative transport         5
## 11 GO:0032309                         icosanoid secretion         5
## 12 GO:0051439 regulation of ubiquitin-protein ligase a...        26
## 13 GO:1903322 positive regulation of protein modificat...        56
## 14 GO:0001516           prostaglandin biosynthetic process         6
## 15 GO:0046457              prostanoid biosynthetic process         6
##    Significant Expected result1
## 1            6     1.59  0.0046
## 2            6     1.62  0.0052
## 3            3     0.42  0.0075
## 4            5     1.31  0.0091
## 5            3     0.46  0.0095
## 6            3     0.46  0.0095
## 7            7     1.48  0.0111
```

```
## 8            7     2.47  0.0112
## 9            2     0.18  0.0116
## 10           2     0.18  0.0116
## 11           2     0.18  0.0116
## 12           4     0.92  0.0123
## 13           6     1.98  0.0134
## 14           2     0.21  0.0170
## 15           2     0.21  0.0170
```

```
Test12_40_DT_SCDE = merge(Test12_40_DT, ResultsSCDE12, by = "GeneNames")
Test13_40_DT_SCDE = merge(Test13_40_DT, ResultsSCDE13, by = "GeneNames")
Test23_40_DT_SCDE = merge(Test23_40_DT, ResultsSCDE12, by = "GeneNames")

# 237 genes
goEnrichDE12_40_SCDE<-topGOAnalysis(Test12_40_DT_SCDE$AssociatedGeneName[!is.na(Test12_40_DT_SCDE$Associ
                           rep(T,sum(!is.na(Test12_40_DT_SCDE$AssociatedGeneName))),
                           Test12_40_DT_SCDE$ResultDiffExp != "NoDiff" &
                             Test12_40_DT_SCDE$Result == "NoDiff")

# 342 genes
goEnrichDE13_40_SCDE<-topGOAnalysis(Test13_40_DT_SCDE$AssociatedGeneName[!is.na(Test13_40_DT_SCDE$Associ
                           rep(T,sum(!is.na(Test13_40_DT_SCDE$AssociatedGeneName))),
                           Test13_40_DT_SCDE$ResultDiffExp != "NoDiff" &
                             Test13_40_DT_SCDE$Result == "NoDiff")

# 196 genes
goEnrichDE23_40_SCDE<-topGOAnalysis(Test23_40_DT_SCDE$AssociatedGeneName[!is.na(Test23_40_DT_SCDE$Associ
                           rep(T,sum(!is.na(Test23_40_DT_SCDE$AssociatedGeneName))),
                           Test23_40_DT_SCDE$ResultDiffExp != "NoDiff" &
                             Test23_40_DT_SCDE$Result == "NoDiff")
```

```
##         GO.ID                                       Term Annotated
## 1  GO:0070527                       platelet aggregation        19
## 2  GO:0050885 neuromuscular process controlling balanc...        20
## 3  GO:0070306             lens fiber cell differentiation         6
## 4  GO:0050881                    musculoskeletal movement         6
## 5  GO:0006814                        sodium ion transport        32
## 6  GO:0010035             response to inorganic substance       104
## 7  GO:0043967                     histone H4 acetylation        40
## 8  GO:0070887      cellular response to chemical stimulus       522
## 9  GO:0030193             regulation of blood coagulation        11
## 10 GO:0048872              homeostasis of number of cells        88
## 11 GO:0016050                        vesicle organization        90
## 12 GO:0019751                    polyol metabolic process        22
## 13 GO:0070841                     inclusion body assembly        12
## 14 GO:0048731                          system development      1022
## 15 GO:1904062 regulation of cation transmembrane trans...        34
##    Significant Expected result1
## 1            5     0.78 0.00082
## 2            5     0.82 0.00105
## 3            3     0.25 0.00125
## 4            3     0.25 0.00125
## 5            6     1.32 0.00167
## 6           11     4.28 0.00341
```

```
## 7            6     1.65 0.00538
## 8           33    21.48 0.00757
## 9            3     0.45 0.00887
## 10           9     3.62 0.00972
## 11           9     3.70 0.01119
## 12           4     0.91 0.01138
## 13           3     0.49 0.01148
## 14          58    42.06 0.01156
## 15           5     1.40 0.01183

##         GO.ID                                        Term Annotated
## 1  GO:0021695               cerebellar cortex development        17
## 2  GO:1903792      negative regulation of anion transport         5
## 3  GO:0007140                                 male meiosis        17
## 4  GO:0002521                   leukocyte differentiation       105
## 5  GO:0006310                             DNA recombination       114
## 6  GO:0046717                               acid secretion        12
## 7  GO:0016579                    protein deubiquitination        54
## 8  GO:0061041                  regulation of wound healing        20
## 9  GO:0045005 DNA-dependent DNA replication maintenanc...        13
## 10 GO:0071347           cellular response to interleukin-1         7
## 11 GO:0000712 resolution of meiotic recombination inte...         7
## 12 GO:0051651              maintenance of location in cell        40
## 13 GO:0002889 regulation of immunoglobulin mediated im...         8
## 14 GO:0032890          regulation of organic acid transport         8
## 15 GO:0019400                    alditol metabolic process         8
##    Significant Expected result1
## 1            6     1.03 0.00032
## 2            3     0.30 0.00198
## 3            5     1.03 0.00262
## 4           14     6.33 0.00391
## 5           17     6.88 0.00433
## 6            4     0.72 0.00437
## 7            9     3.26 0.00450
## 8            5     1.21 0.00565
## 9            4     0.78 0.00601
## 10           3     0.42 0.00634
## 11           3     0.42 0.00634
## 12           7     2.41 0.00903
## 13           3     0.48 0.00970
## 14           3     0.48 0.00970
## 15           3     0.48 0.00970

##         GO.ID                                        Term Annotated
## 1  GO:0051340                 regulation of ligase activity        45
## 2  GO:0051438 regulation of ubiquitin-protein transfer...        46
## 3  GO:0043968                       histone H2A acetylation        12
## 4  GO:0030071 regulation of mitotic metaphase/anaphase...        37
## 5  GO:0045682           regulation of epidermis development        13
## 6  GO:0015711                       organic anion transport        70
## 7  GO:0031145 anaphase-promoting complex-dependent pro...        42
## 8  GO:1901571               fatty acid derivative transport         5
## 9  GO:0071715                           icosanoid transport         5
## 10 GO:0032309                           icosanoid secretion         5
```

```
## 11 GO:0051439 regulation of ubiquitin-protein ligase a...        26
## 12 GO:1903322 positive regulation of protein modificat...        56
## 13 GO:0006310                            DNA recombination       114
## 14 GO:0051443 positive regulation of ubiquitin-protein...        16
## 15 GO:0051488 activation of anaphase-promoting complex...         6
##    Significant Expected result1
## 1            6     1.54  0.0040
## 2            6     1.57  0.0044
## 3            3     0.41  0.0069
## 4            5     1.27  0.0079
## 5            3     0.44  0.0087
## 6            7     2.39  0.0095
## 7            7     1.44  0.0104
## 8            2     0.17  0.0109
## 9            2     0.17  0.0109
## 10           2     0.17  0.0109
## 11           4     0.89  0.0110
## 12           6     1.92  0.0116
## 13           9     3.90  0.0155
## 14           3     0.55  0.0159
## 15           2     0.21  0.0159
```

```
Test12_40_DT_MAST = merge(Test12_40_DT, ResultsMAST12, by = "GeneNames")
Test13_40_DT_MAST = merge(Test13_40_DT, ResultsMAST13, by = "GeneNames")
Test23_40_DT_MAST = merge(Test23_40_DT, ResultsMAST12, by = "GeneNames")
# Checking order
sum(Test12_40_DT_MAST$GeneNames != rownames(DC.G1))
```

```
## [1] 0
```

```
sum(Test13_40_DT_MAST$GeneNames != rownames(DC.G1))
```

```
## [1] 0
```

```
sum(Test23_40_DT_MAST$GeneNames != rownames(DC.G1))
```

```
## [1] 0
```

```
HeatmapFormat(cbind(DC.G1, DC.S)[order(Test12_40_DT_MAST$ExpLogFC),],
              GenesSel = Test12_40_DT_MAST$GeneNames[Test12_40_DT_MAST$ResultDiffExp != "NoDiff" &
                             Test12_40_DT_MAST$ResultsHurdle == "NoDiff"],
              ColSideColors = Cell.Colour[!grepl("G2M", colnames(DC.all))],
              main = "Detected by BASiCS but not by MAST", Rowv = TRUE)
legend('topright', c("G1", "S"), pch = 15, col = unique(Cell.Colour)[1:2])
```

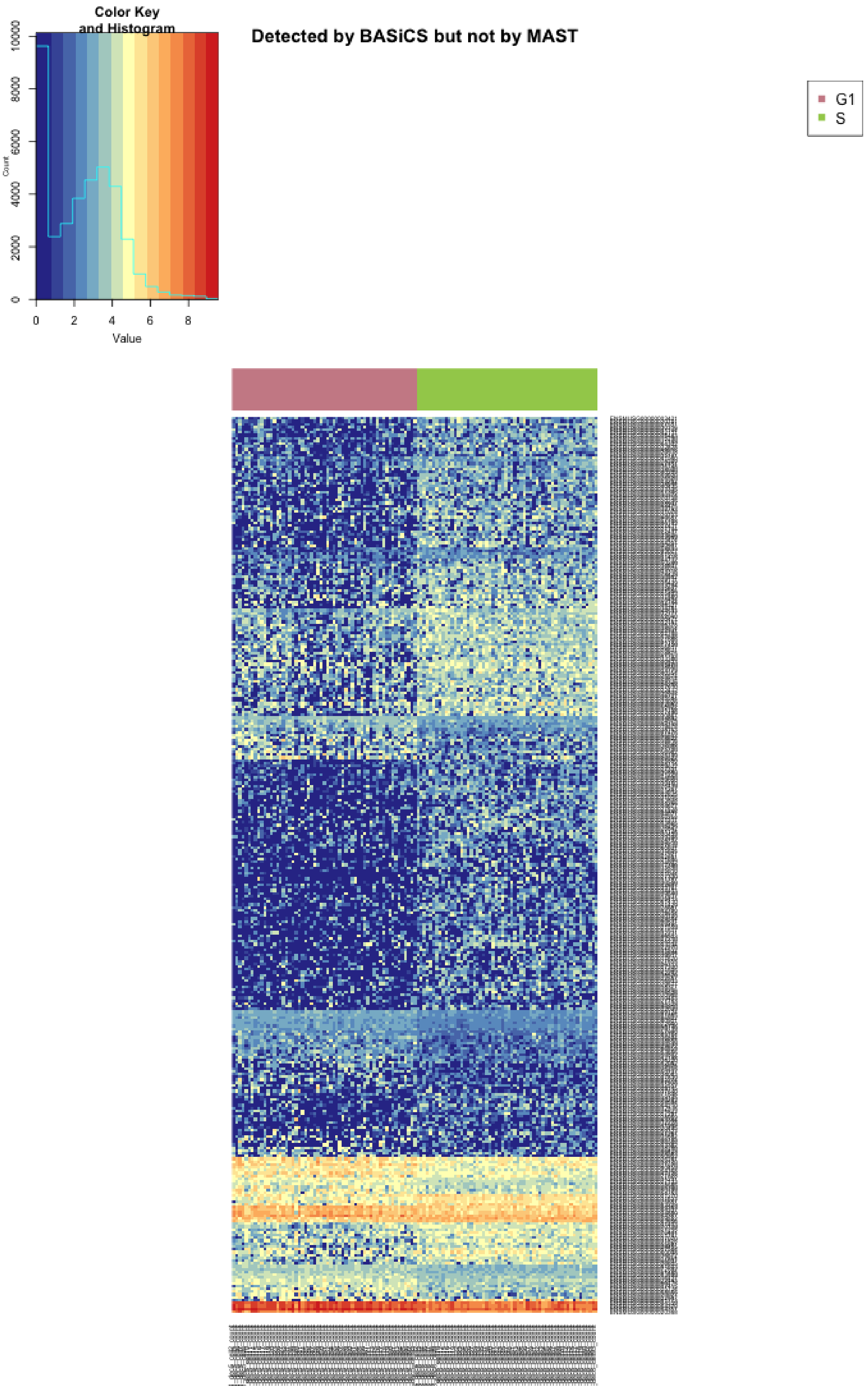
Color Key
and Histogram
Count
Value
Detected by BASiCS but not by MAST
G1
S

```r
HeatmapFormat(cbind(DC.G1, DC.G2M)[order(Test13_40_DT_MAST$ExpLogFC),],
              GenesSel = Test13_40_DT_MAST$GeneNames[Test13_40_DT_MAST$ResultDiffExp != "NoDiff" &
                             Test13_40_DT_MAST$ResultsHurdle == "NoDiff"],
              ColSideColors = Cell.Colour[!grepl("S", colnames(DC.all))],
              main = "Detected by BASiCS but not by MAST", Rowv = TRUE)
legend('topright', c("G1", "G2M"), pch = 15, col = unique(Cell.Colour)[c(1,3)])
```

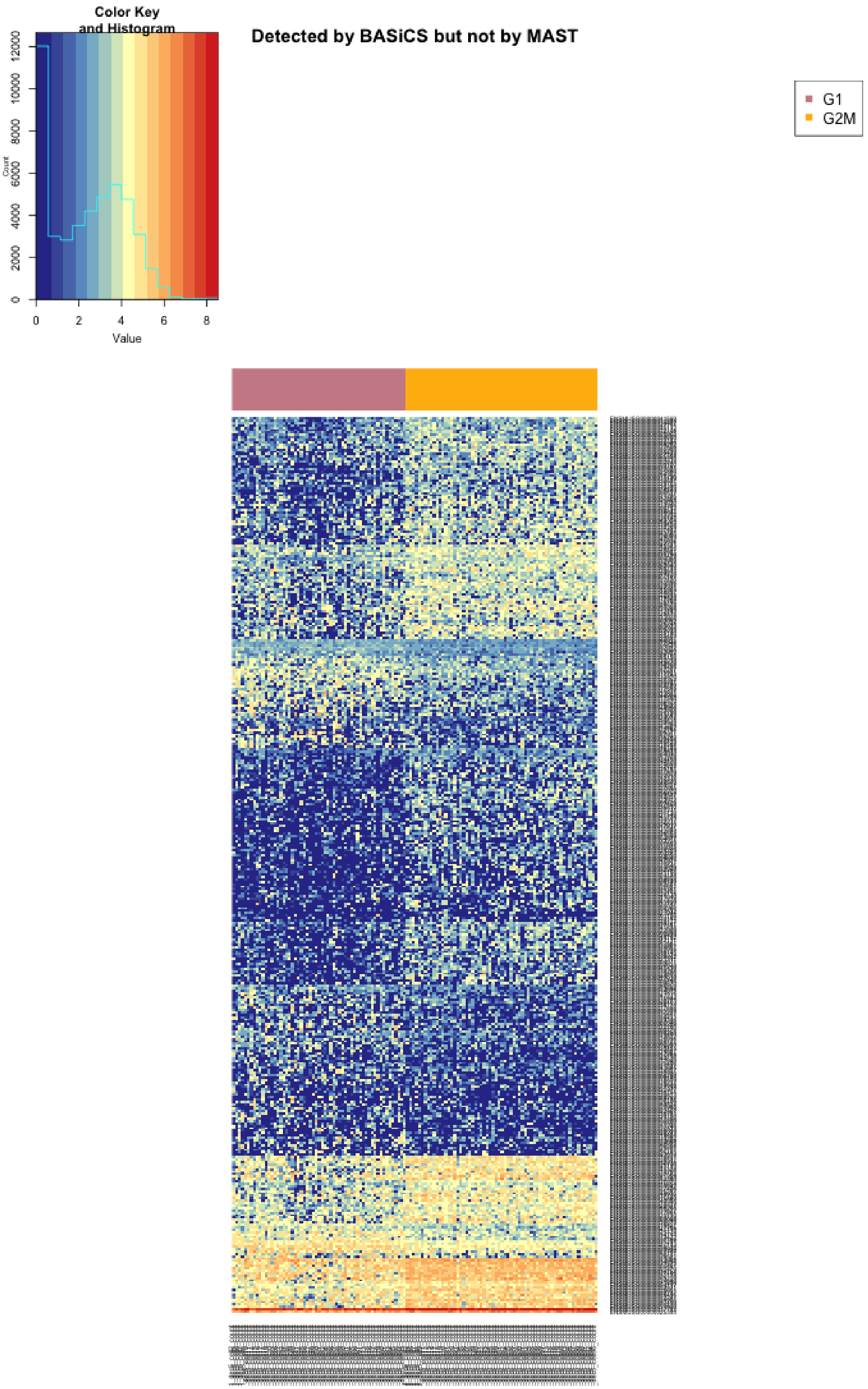
Color Key
and Histogram
Count
Value
Detected by BASiCS but not by MAST
G1
G2M

```
HeatmapFormat(cbind(DC.S, DC.G2M)[order(Test23_40_DT_MAST$ExpLogFC),],
              GenesSel = Test23_40_DT_MAST$GeneNames[Test23_40_DT_MAST$ResultDiffExp != "NoDiff" &
                             Test23_40_DT_MAST$ResultsHurdle == "NoDiff"],
              ColSideColors = Cell.Colour[!grepl("G1", colnames(DC.all))],
              main = "Detected by BASiCS but not by MAST", Rowv = TRUE)
legend('topright', c("S", "G2M"), pch = 15, col = unique(Cell.Colour)[c(2,3)])
```

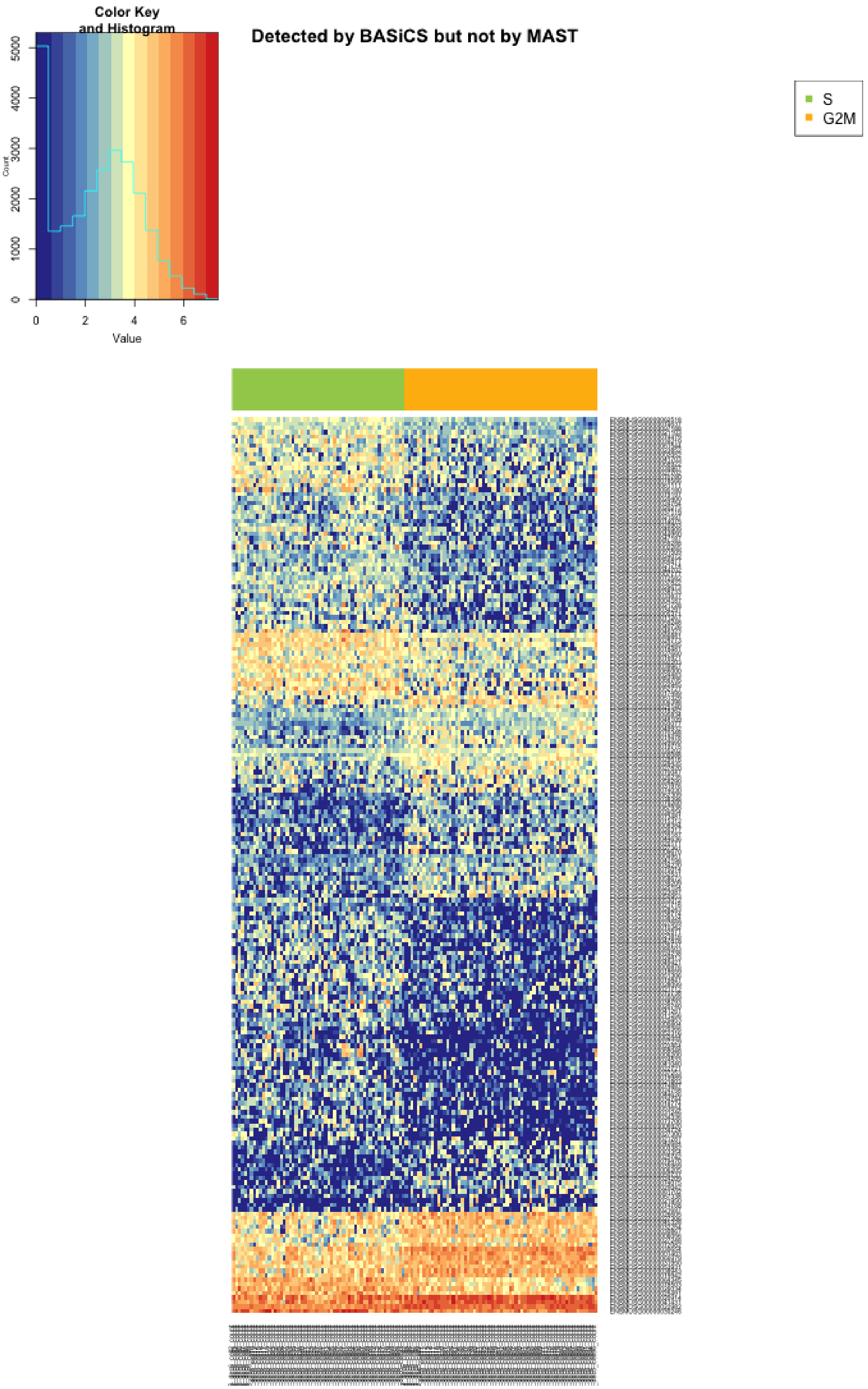
Color Key
and Histogram
Count
Value
Detected by BASiCS but not by MAST
S
G2M

```
HeatmapFormat(cbind(DC.G1, DC.S)[order(Test12_40_DT_MAST$ExpLogFC),],
              GenesSel = Test12_40_DT_MAST$GeneNames[Test12_40_DT_MAST$ResultDiffExp == "NoDiff" &
                             Test12_40_DT_MAST$ResultsHurdle != "NoDiff"],
              ColSideColors = Cell.Colour[!grepl("G2M", colnames(DC.all))],
              main = "Detected by MAST but not by BASiCS", Rowv = TRUE)
legend('topright', c("G1", "S"), pch = 15, col = unique(Cell.Colour)[1:2])
```

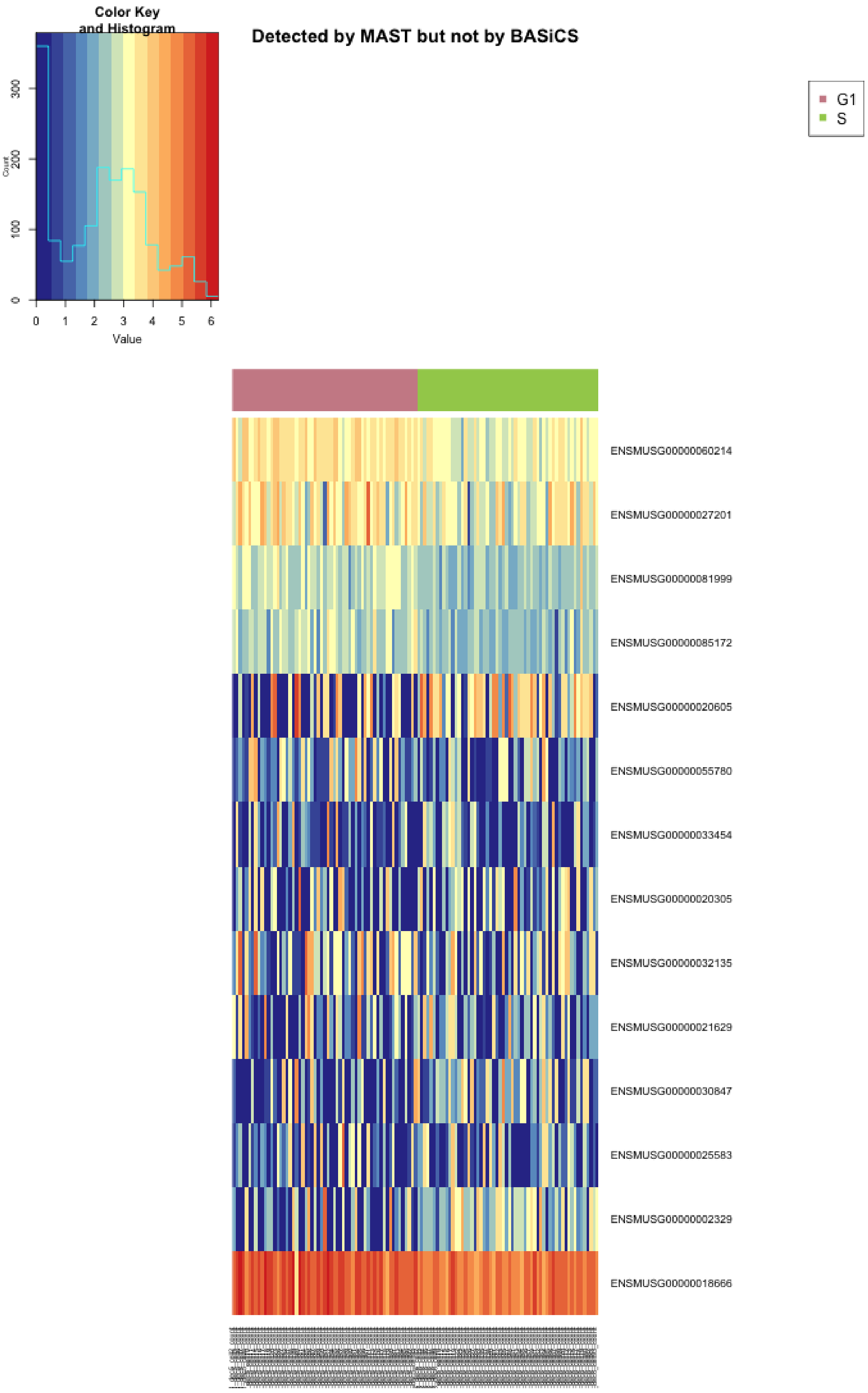
Color Key
and Histogram
Count
0
100
200
300
0 1 2 3 4 5 6
Value
Detected by MAST but not by BASiCS
G1
S
ENSMUSG00000060214
ENSMUSG00000027201
ENSMUSG00000081999
ENSMUSG00000085172
ENSMUSG00000020605
ENSMUSG00000055780
ENSMUSG00000033454
ENSMUSG00000020305
ENSMUSG00000032135
ENSMUSG00000021629
ENSMUSG00000030847
ENSMUSG00000025583
ENSMUSG00000002329
ENSMUSG00000018666

```r
HeatmapFormat(cbind(DC.G1, DC.G2M)[order(Test13_40_DT_MAST$ExpLogFC),],
              GenesSel = Test13_40_DT_MAST$GeneNames[Test13_40_DT_MAST$ResultDiffExp == "NoDiff" &
                             Test13_40_DT_MAST$ResultsHurdle != "NoDiff"],
              ColSideColors = Cell.Colour[!grepl("S", colnames(DC.all))],
              main = "Detected by BASiCS but not by MAST", Rowv = TRUE)
legend('topright', c("G1", "G2M"), pch = 15, col = unique(Cell.Colour)[c(1,3)])
```

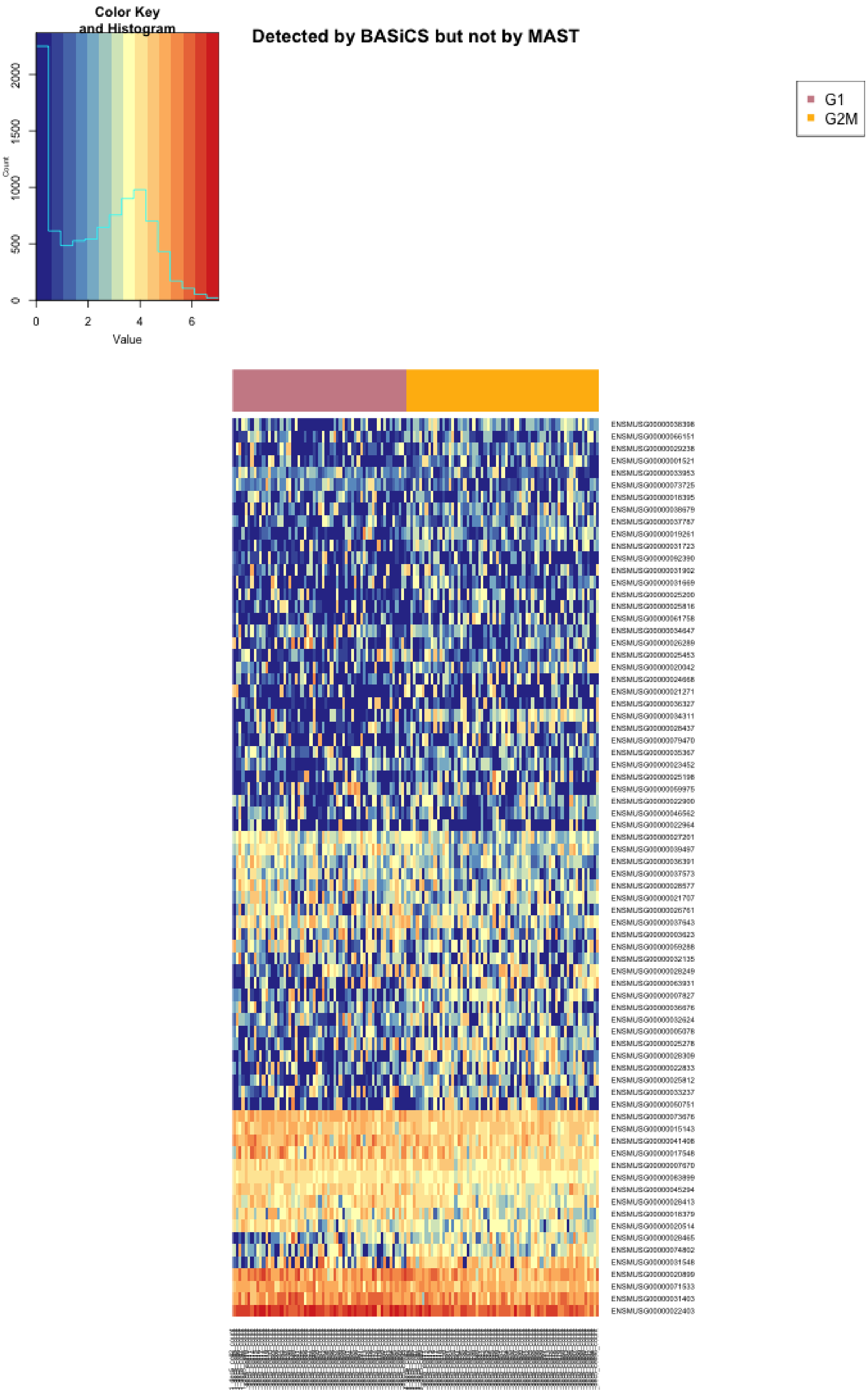
Color Key
and Histogram
Count
Value
Detected by BASiCS but not by MAST
G1
G2M

```r
HeatmapFormat(cbind(DC.S, DC.G2M)[order(Test23_40_DT_MAST$ExpLogFC),],
              GenesSel = Test23_40_DT_MAST$GeneNames[Test23_40_DT_MAST$ResultDiffExp == "NoDiff" &
                             Test23_40_DT_MAST$ResultsHurdle != "NoDiff"],
              ColSideColors = Cell.Colour[!grepl("G1", colnames(DC.all))],
              main = "Detected by BASiCS but not by MAST", Rowv = TRUE)
legend('topright', c("S", "G2M"), pch = 15, col = unique(Cell.Colour)[c(2,3)])
```

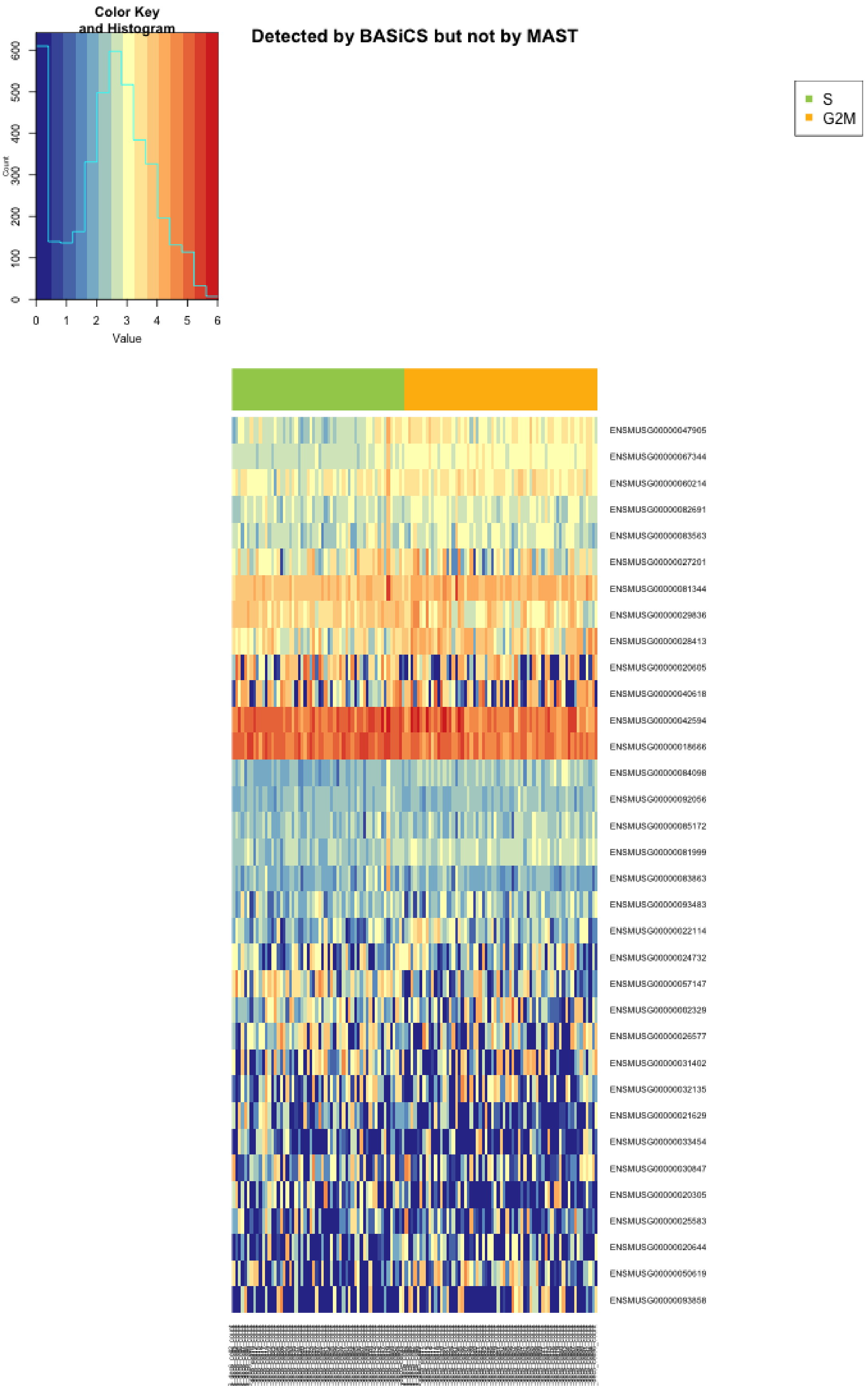
Color Key
and Histogram
Count
0
100
200
300
400
500
600
0
1
2
3
4
5
6
Value
Detected by BASiCS but not by MAST
S
G2M
ENSMUSG00000047905
ENSMUSG00000067344
ENSMUSG00000060214
ENSMUSG00000082691
ENSMUSG00000083563
ENSMUSG00000027201
ENSMUSG00000081344
ENSMUSG00000029836
ENSMUSG00000028413
ENSMUSG00000020605
ENSMUSG00000040618
ENSMUSG00000042594
ENSMUSG00000018666
ENSMUSG00000084098
ENSMUSG00000092056
ENSMUSG00000085172
ENSMUSG00000081999
ENSMUSG00000083863
ENSMUSG00000093483
ENSMUSG00000022114
ENSMUSG00000024732
ENSMUSG00000057147
ENSMUSG00000002329
ENSMUSG00000026577
ENSMUSG00000031402
ENSMUSG00000032135
ENSMUSG00000021629
ENSMUSG00000033454
ENSMUSG00000030847
ENSMUSG00000020305
ENSMUSG00000025583
ENSMUSG00000020644
ENSMUSG00000050619
ENSMUSG00000093858

```r
Test12_40_DT_MASTNotCDR = merge(Test12_40_DT, ResultsMASTNotCDR12, by = "GeneNames")
Test13_40_DT_MASTNotCDR = merge(Test13_40_DT, ResultsMASTNotCDR13, by = "GeneNames")
Test23_40_DT_MASTNotCDR = merge(Test23_40_DT, ResultsMASTNotCDR12, by = "GeneNames")

# Checking order
sum(Test12_40_DT_MASTNotCDR$GeneNames != rownames(DC.G1))
```

```
## [1] 0
```

```r
sum(Test13_40_DT_MASTNotCDR$GeneNames != rownames(DC.G1))
```

```
## [1] 0
```

```r
sum(Test23_40_DT_MASTNotCDR$GeneNames != rownames(DC.G1))
```

```
## [1] 0
```

```r
HeatmapFormat(cbind(DC.G1, DC.S)[order(Test12_40_DT_MASTNotCDR$ExpLogFC),],
              GenesSel = Test12_40_DT_MASTNotCDR$GeneNames[Test12_40_DT_MASTNotCDR$ResultDiffExp != "No
                             Test12_40_DT_MASTNotCDR$ResultsCont == "NoDiff"],
              ColSideColors = Cell.Colour[!grepl("G2M", colnames(DC.all))],
              main = "Detected by BASiCS but not by MAST (no CDR)", Rowv = TRUE)
legend('topright', c("G1", "S"), pch = 15, col = unique(Cell.Colour)[1:2])
```

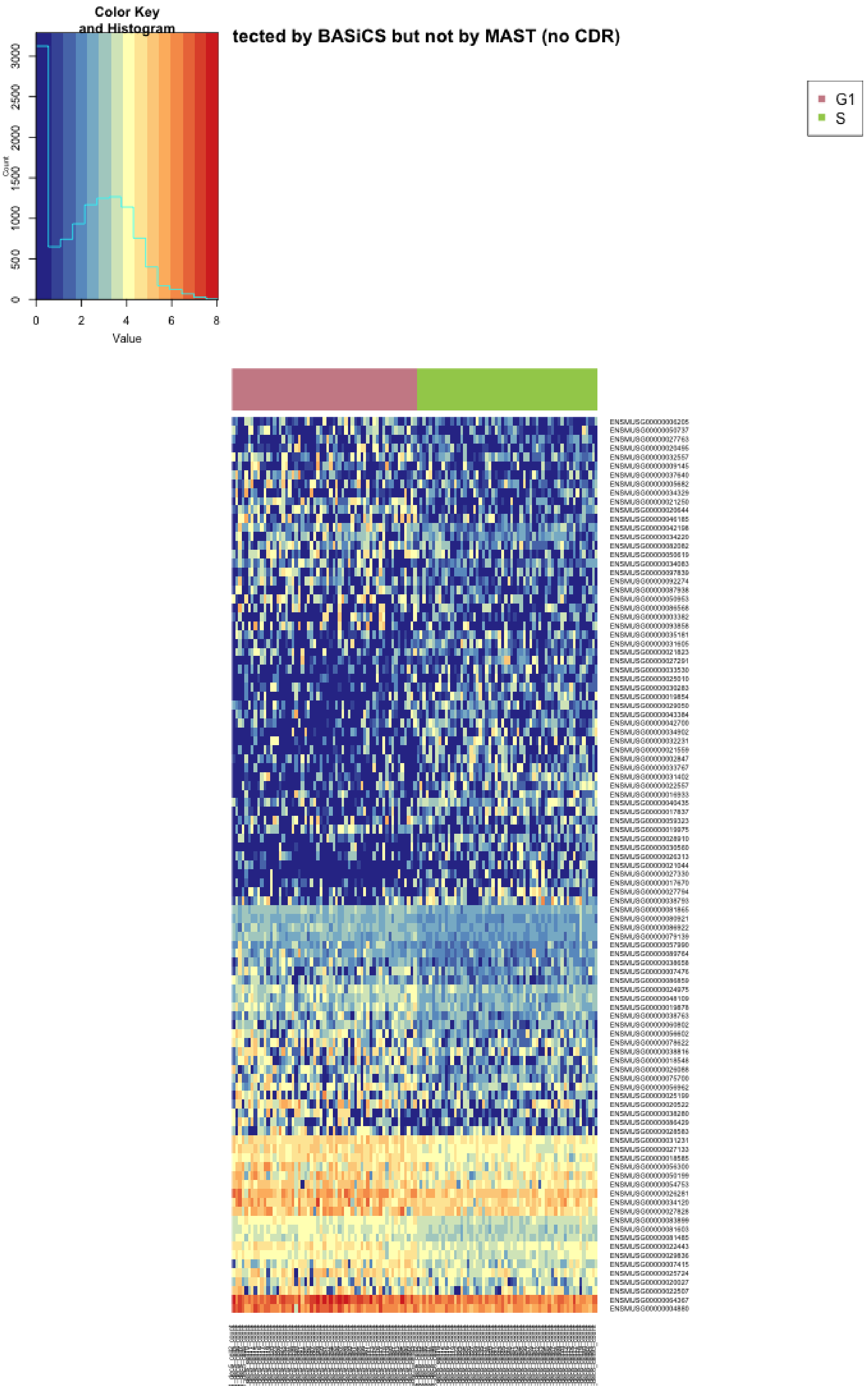
Color Key
and Histogram
Count
Value
tected by BASiCS but not by MAST (no CDR)
G1
S

```r
HeatmapFormat(cbind(DC.G1, DC.G2M)[order(Test13_40_DT_MASTNotCDR$ExpLogFC),],
              GenesSel = Test13_40_DT_MASTNotCDR$GeneNames[Test13_40_DT_MASTNotCDR$ResultDiffExp != "NoD
                             Test13_40_DT_MASTNotCDR$ResultsCont == "NoDiff"],
              ColSideColors = Cell.Colour[!grepl("S", colnames(DC.all))],
              main = "Detected by BASiCS but not by MAST (no CDR)", Rowv = TRUE)
legend('topright', c("G1", "G2M"), pch = 15, col = unique(Cell.Colour)[c(1,3)])
```

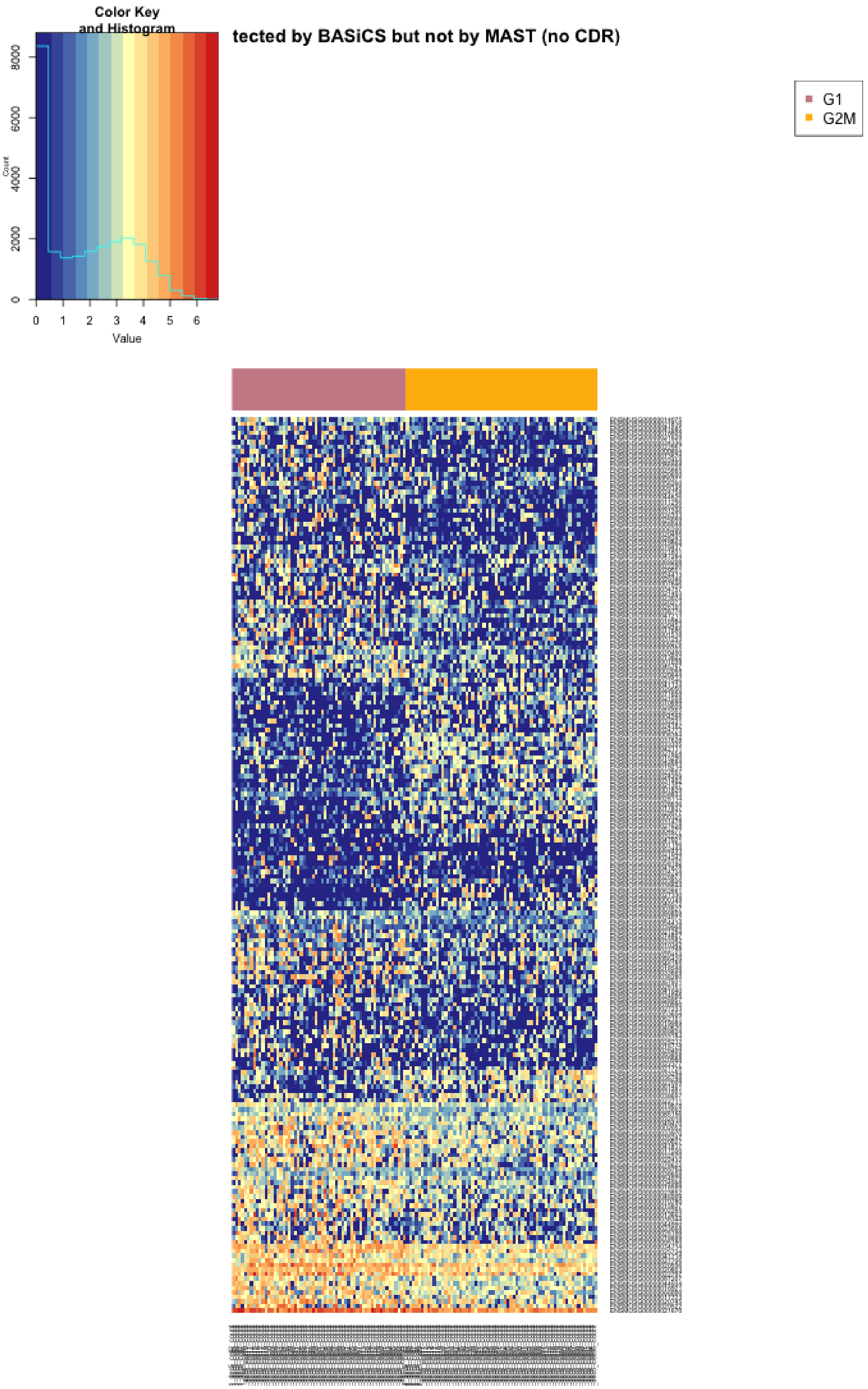

Color Key
and Histogram
Count
Value
0
2000
4000
6000
8000
0 1 2 3 4 5 6
tected by BASiCS but not by MAST (no CDR)
G1
G2M

```
HeatmapFormat(cbind(DC.S, DC.G2M)[order(Test23_40_DT_MASTNotCDR$ExpLogFC),],
              GenesSel = Test23_40_DT_MASTNotCDR$GeneNames[Test23_40_DT_MASTNotCDR$ResultDiffExp != "No[illegible]
                             Test23_40_DT_MASTNotCDR$ResultsCont == "NoDiff"],
              ColSideColors = Cell.Colour[!grepl("G1", colnames(DC.all))],
              main = "Detected by BASiCS but not by MAST (no CDR)", Rowv = TRUE)
legend('topright', c("S", "G2M"), pch = 15, col = unique(Cell.Colour)[c(2,3)])
```

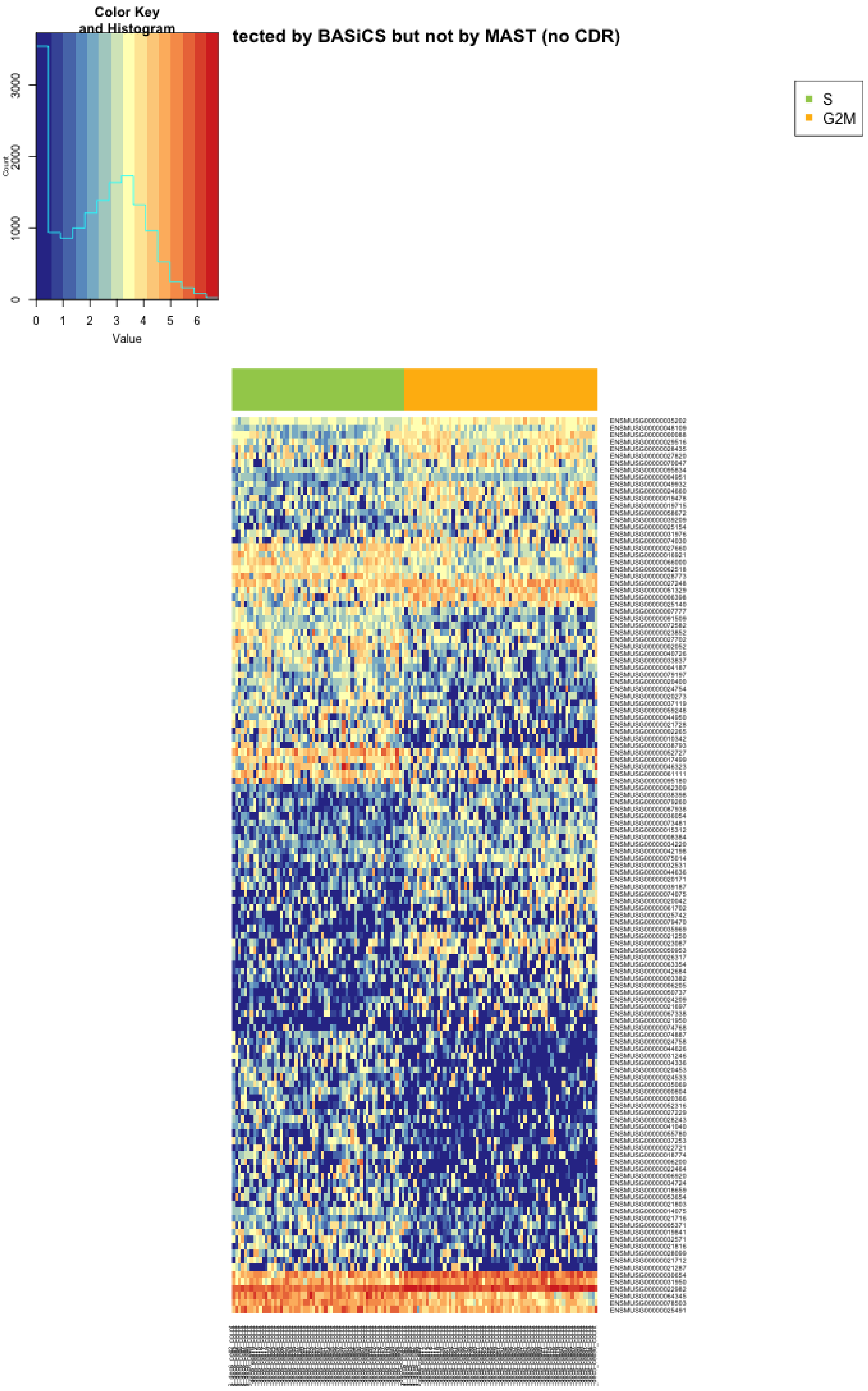
tected by BASiCS but not by MAST (no CDR)
Color Key
and Histogram
Count
0
1000
2000
3000
0 1 2 3 4 5 6
Value
S
G2M

```
HeatmapFormat(cbind(DC.G1, DC.S)[order(Test12_40_DT_MASTNotCDR$ExpLogFC),],
              GenesSel = Test12_40_DT_MASTNotCDR$GeneNames[Test12_40_DT_MASTNotCDR$ResultDiffExp == "NoD
                             Test12_40_DT_MASTNotCDR$ResultsCont != "NoDiff"],
              ColSideColors = Cell.Colour[!grepl("G2M", colnames(DC.all))],
              main = "Detected by MAST(no CDR) but not by BASiCS", Rowv = TRUE)
legend('topright', c("G1", "S"), pch = 15, col = unique(Cell.Colour)[1:2])
```

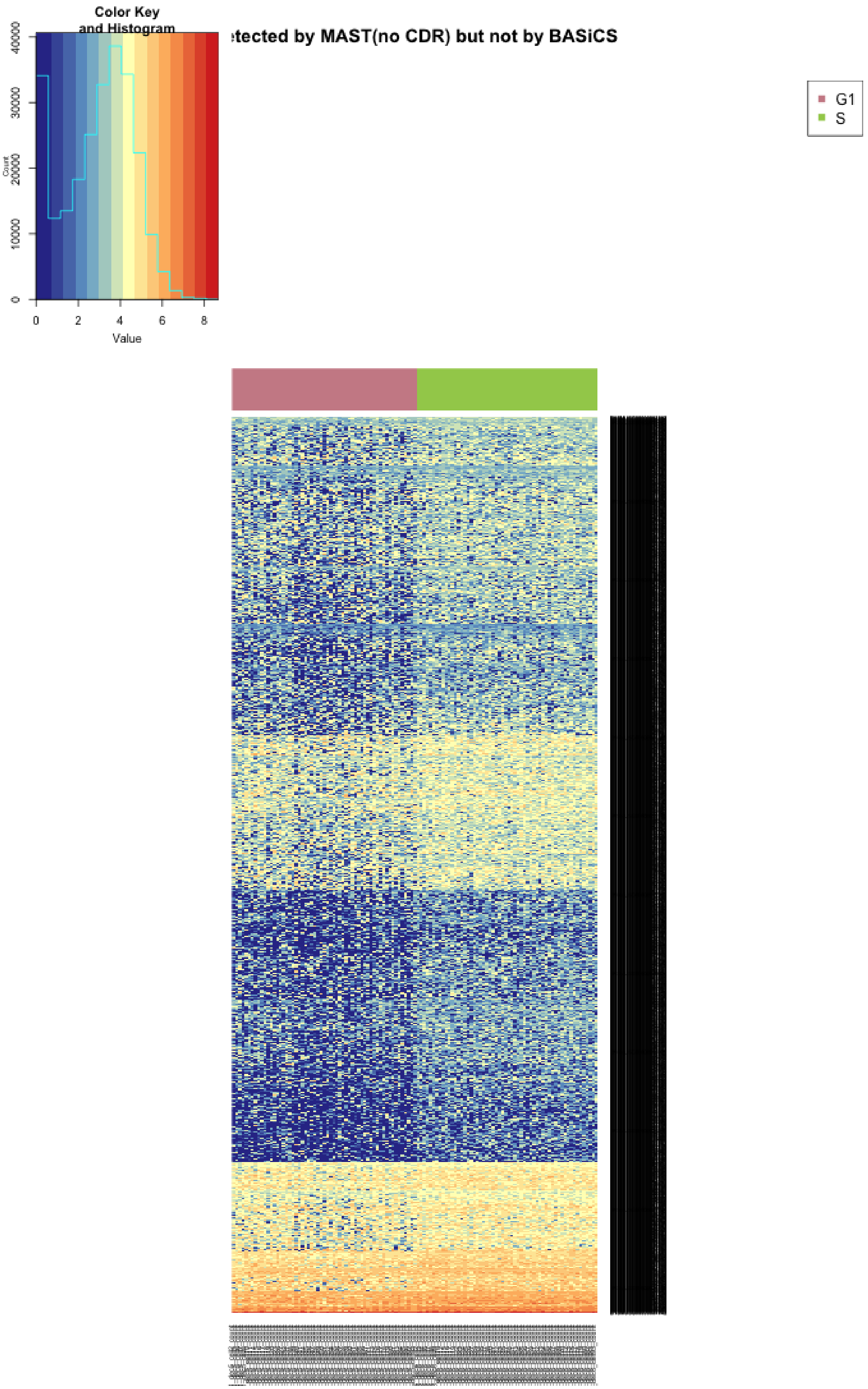
Color Key
and Histogram
Count
Value
etected by MAST(no CDR) but not by BASiCS
G1
S

```
HeatmapFormat(cbind(DC.G1, DC.G2M)[order(Test13_40_DT_MASTNotCDR$ExpLogFC),],
              GenesSel = Test13_40_DT_MASTNotCDR$GeneNames[Test13_40_DT_MASTNotCDR$ResultDiffExp == "NoD
                             Test13_40_DT_MASTNotCDR$ResultsCont != "NoDiff"],
              ColSideColors = Cell.Colour[!grepl("S", colnames(DC.all))],
              main = "Detected by MAST(no CDR) but not by BASiCS", Rowv = TRUE)
legend('topright', c("G1", "G2M"), pch = 15, col = unique(Cell.Colour)[c(1,3)])
```

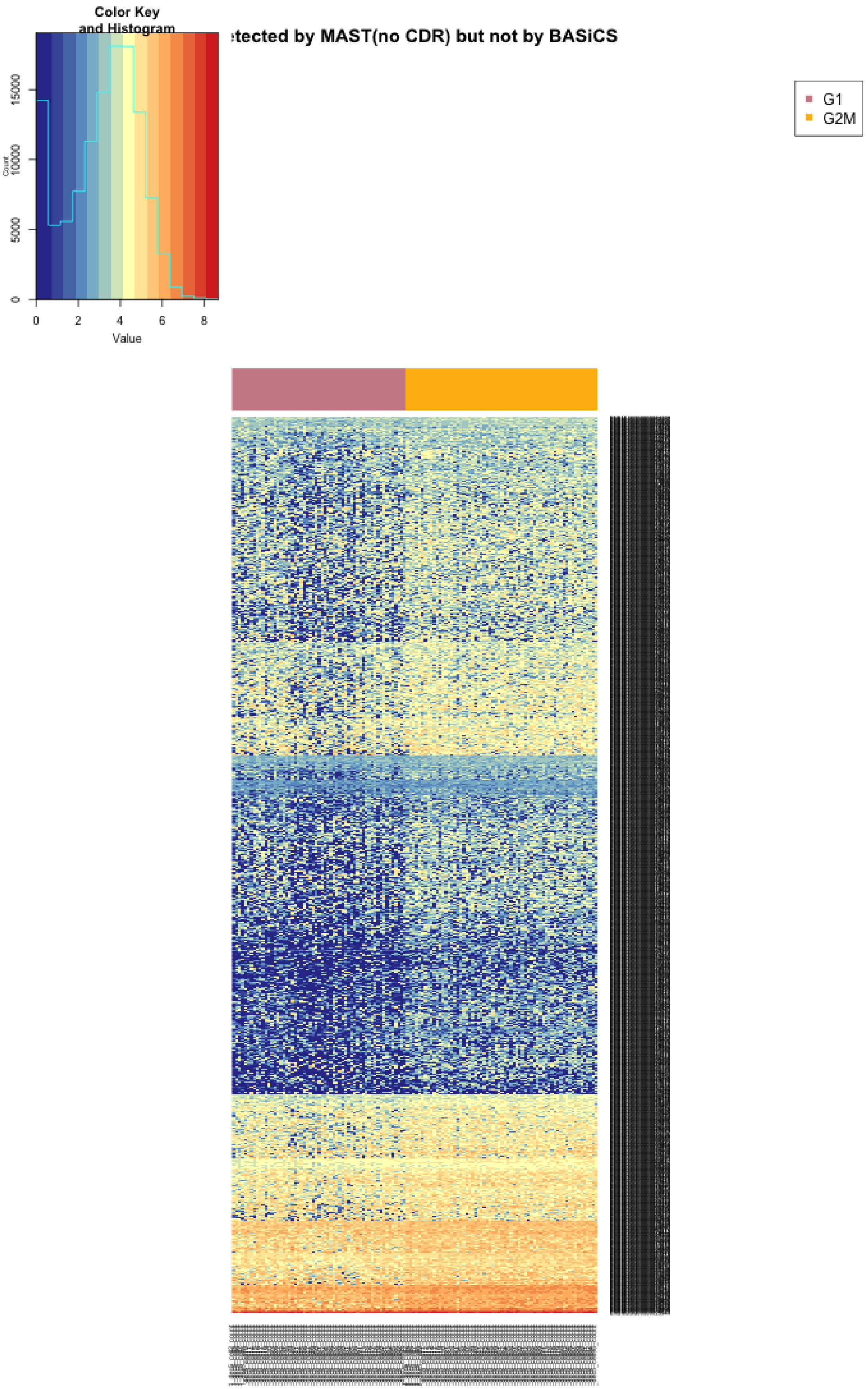
Color Key
and Histogram
Count
15000
10000
5000
0
0
2
4
6
8
Value
:tected by MAST(no CDR) but not by BASiCS
G1
G2M

```r
HeatmapFormat(cbind(DC.S, DC.G2M)[order(Test23_40_DT_MASTNotCDR$ExpLogFC),],
              GenesSel = Test23_40_DT_MASTNotCDR$GeneNames[Test23_40_DT_MASTNotCDR$ResultDiffExp == "No[illegible]
                             Test23_40_DT_MASTNotCDR$ResultsCont != "NoDiff"],
              ColSideColors = Cell.Colour[!grepl("G1", colnames(DC.all))],
              main = "Detected by MAST(no CDR) but not by BASiCS", Rowv = TRUE)
legend('topright', c("S", "G2M"), pch = 15, col = unique(Cell.Colour)[c(2,3)])
```

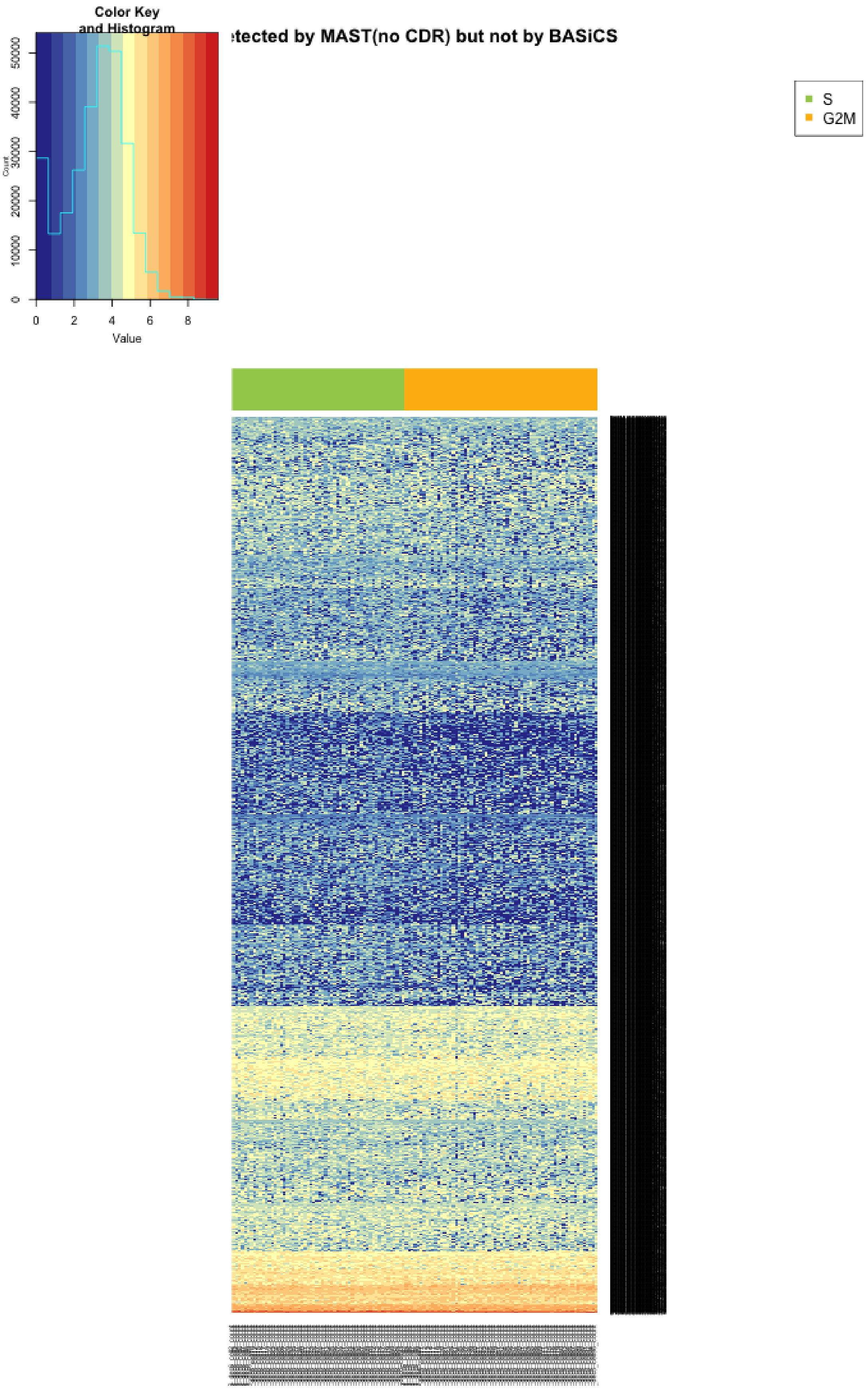
Color Key
and Histogram
Count
Value
0
2
4
6
8
0
10000
20000
30000
40000
50000
etected by MAST(no CDR) but not by BASiCS
S
G2M

```r
Test12_40_DT_SCDE = merge(Test12_40_DT, ResultsSCDE12, by = "GeneNames")
Test13_40_DT_SCDE = merge(Test13_40_DT, ResultsSCDE13, by = "GeneNames")
Test23_40_DT_SCDE = merge(Test23_40_DT, ResultsSCDE12, by = "GeneNames")
# Checking order
sum(Test12_40_DT_SCDE$GeneNames != rownames(DC.G1))
```

```
## [1] 0
```

```r
sum(Test13_40_DT_SCDE$GeneNames != rownames(DC.G1))
```

```
## [1] 0
```

```r
sum(Test23_40_DT_SCDE$GeneNames != rownames(DC.G1))
```

```
## [1] 0
```

```r
HeatmapFormat(cbind(DC.G1, DC.S)[order(Test12_40_DT_SCDE$ExpLogFC),],
              GenesSel = Test12_40_DT_SCDE$GeneNames[Test12_40_DT_SCDE$ResultDiffExp != "NoDiff" &
                             Test12_40_DT_SCDE$Result == "NoDiff"],
              ColSideColors = Cell.Colour[!grepl("G2M", colnames(DC.all))],
              main = "Detected by BASiCS but not by SCDE", Rowv = TRUE)
legend('topright', c("G1", "S"), pch = 15, col = unique(Cell.Colour)[1:2])
```

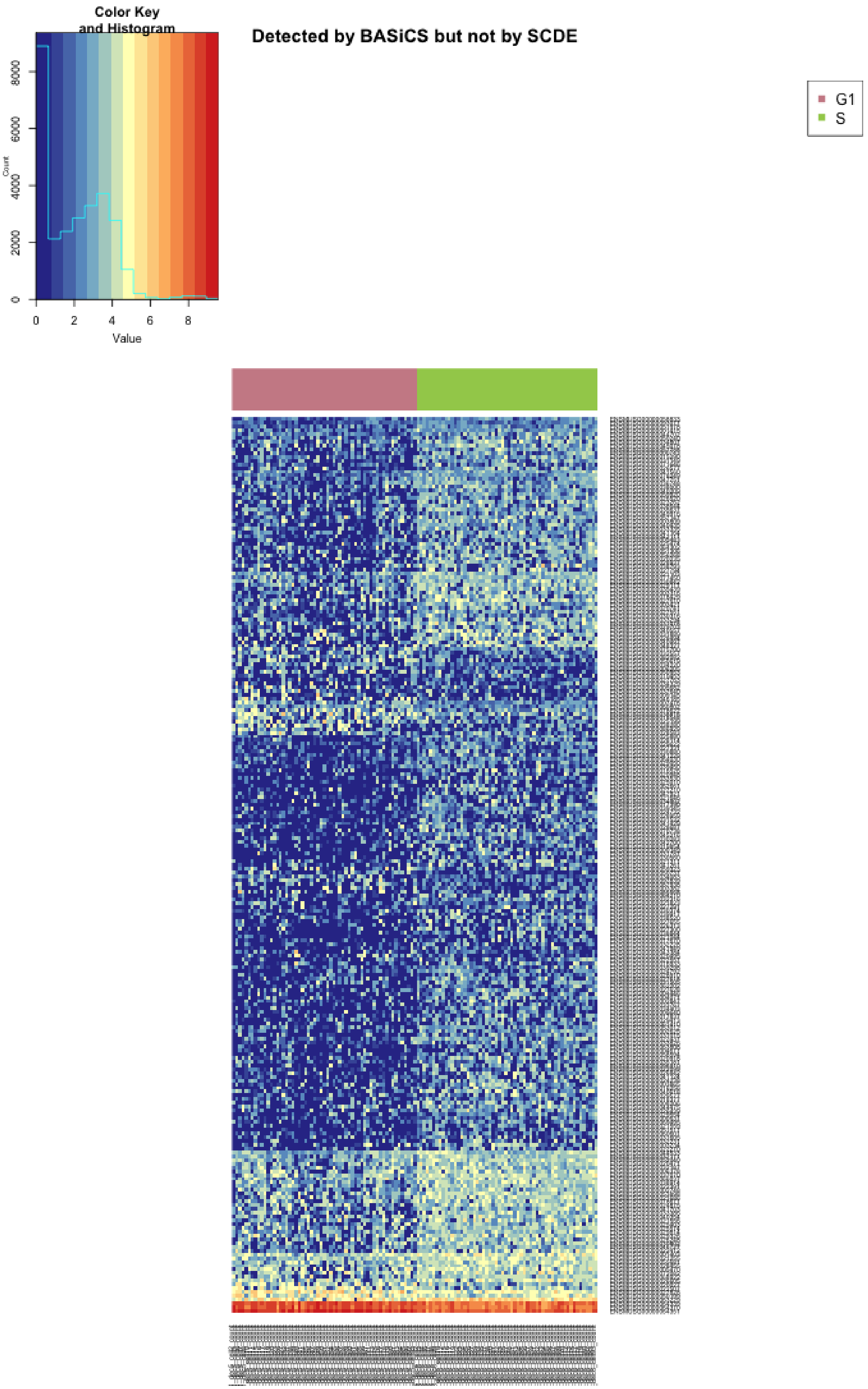
Color Key
and Histogram
Count
Value
Detected by BASiCS but not by SCDE
G1
S

```
HeatmapFormat(cbind(DC.G1, DC.G2M)[order(Test13_40_DT_SCDE$ExpLogFC),],
              GenesSel = Test13_40_DT_SCDE$GeneNames[Test13_40_DT_SCDE$ResultDiffExp != "NoDiff" &
                             Test13_40_DT_SCDE$Result == "NoDiff"],
              ColSideColors = Cell.Colour[!grepl("S", colnames(DC.all))],
              main = "Detected by BASiCS but not by SCDE", Rowv = TRUE)
legend('topright', c("G1", "G2M"), pch = 15, col = unique(Cell.Colour)[c(1,3)])
```

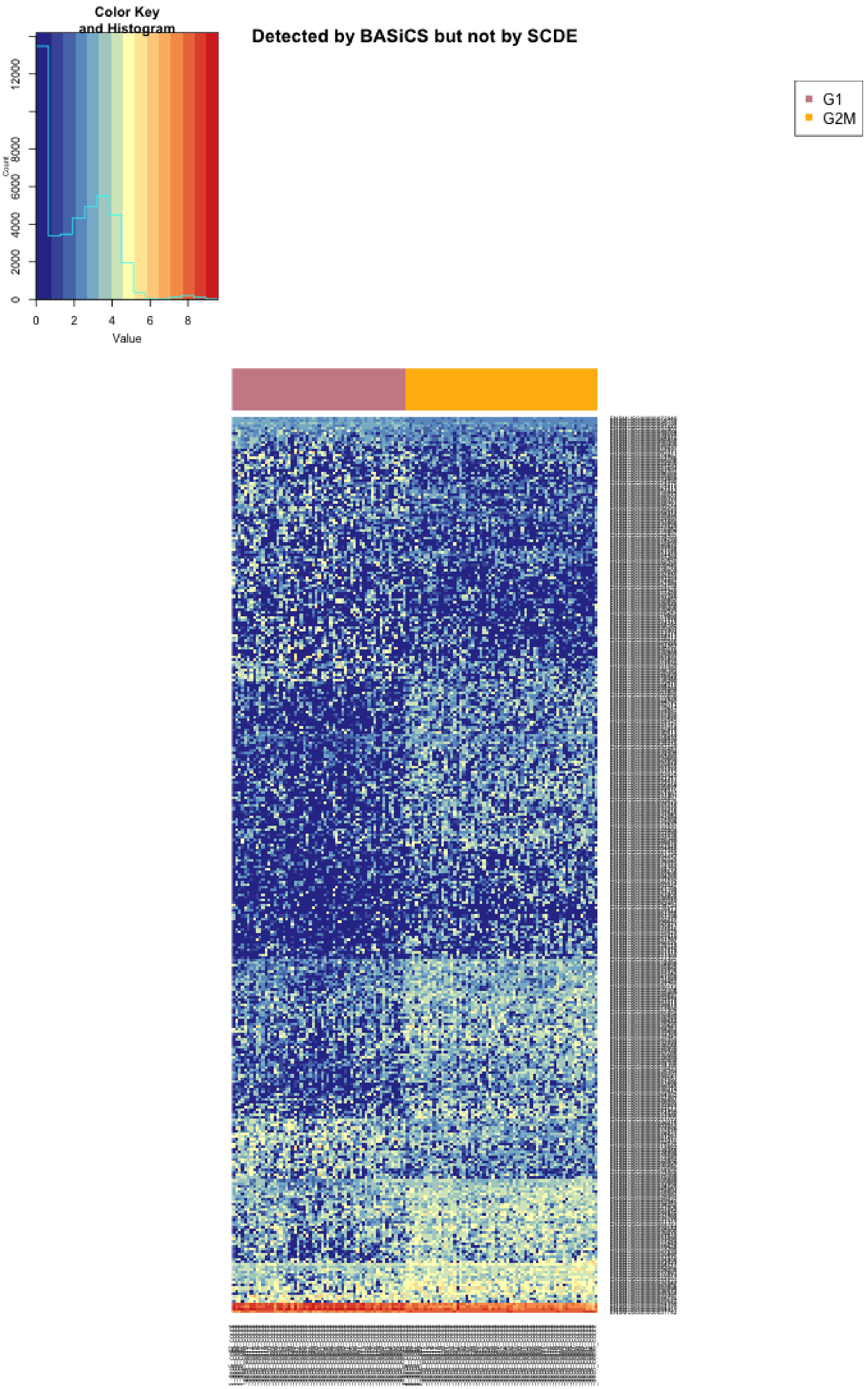
Color Key
and Histogram
Count
Value
Detected by BASiCS but not by SCDE
G1
G2M

```
HeatmapFormat(cbind(DC.S, DC.G2M)[order(Test23_40_DT_SCDE$ExpLogFC),],
              GenesSel = Test23_40_DT_SCDE$GeneNames[Test23_40_DT_SCDE$ResultDiffExp != "NoDiff" &
                             Test23_40_DT_SCDE$Result == "NoDiff"],
              ColSideColors = Cell.Colour[!grepl("G1", colnames(DC.all))],
              main = "Detected by BASiCS but not by SCDE", Rowv = TRUE)
legend('topright', c("S", "G2M"), pch = 15, col = unique(Cell.Colour)[c(2,3)])
```

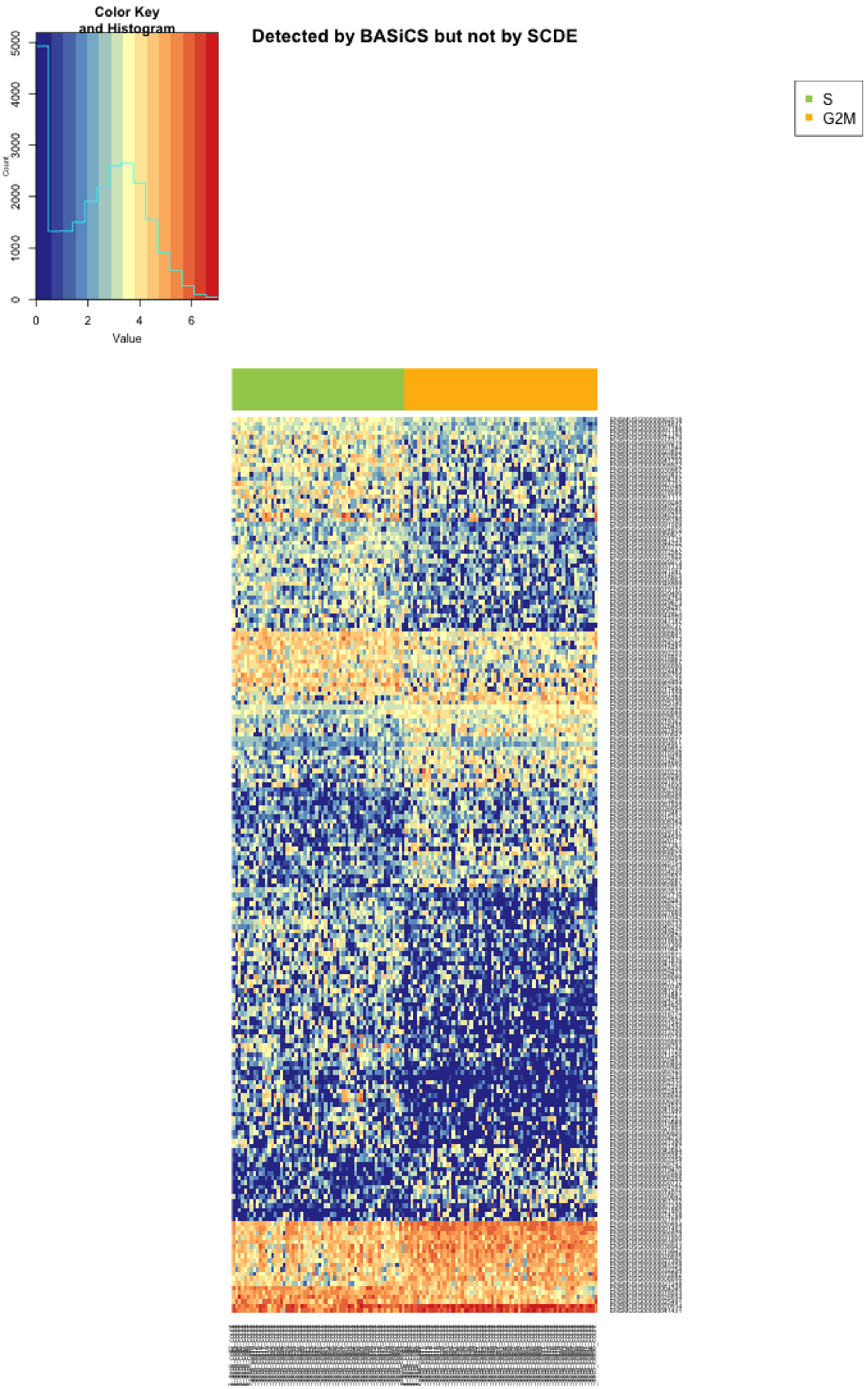
Color Key
and Histogram
Count
0
1000
2000
3000
4000
5000
0
2
4
6
Value
Detected by BASiCS but not by SCDE
S
G2M

```
HeatmapFormat(cbind(DC.G1, DC.S)[order(Test12_40_DT_SCDE$ExpLogFC),],
              GenesSel = Test12_40_DT_SCDE$GeneNames[Test12_40_DT_SCDE$ResultDiffExp == "NoDiff" &
                             Test12_40_DT_SCDE$Result != "NoDiff"],
              ColSideColors = Cell.Colour[!grepl("G2M", colnames(DC.all))],
              main = "Detected by SCDE but not by BASiCS", Rowv = TRUE)
legend('topright', c("G1", "S"), pch = 15, col = unique(Cell.Colour)[1:2])
```

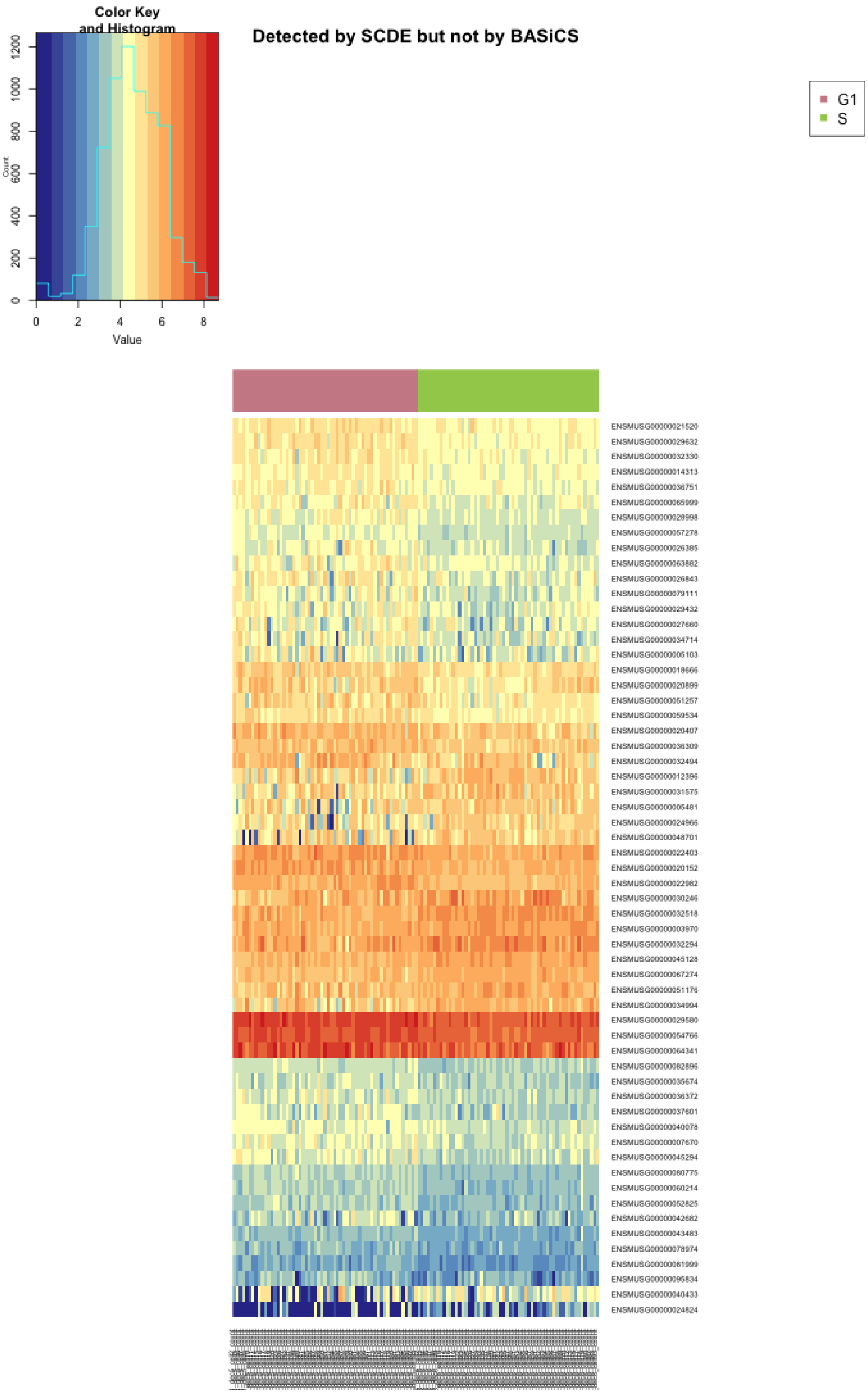
Color Key
and Histogram
Count
Value
Detected by SCDE but not by BASiCS
G1
S

```
HeatmapFormat(cbind(DC.G1, DC.G2M)[order(Test13_40_DT_SCDE$ExpLogFC),],
              GenesSel = Test13_40_DT_SCDE$GeneNames[Test13_40_DT_SCDE$ResultDiffExp == "NoDiff" &
                             Test13_40_DT_SCDE$Result != "NoDiff"],
              ColSideColors = Cell.Colour[!grepl("S", colnames(DC.all))],
              main = "Detected by SCDE but not by BASiCS", Rowv = TRUE)
legend('topright', c("G1", "G2M"), pch = 15, col = unique(Cell.Colour)[c(1,3)])
```

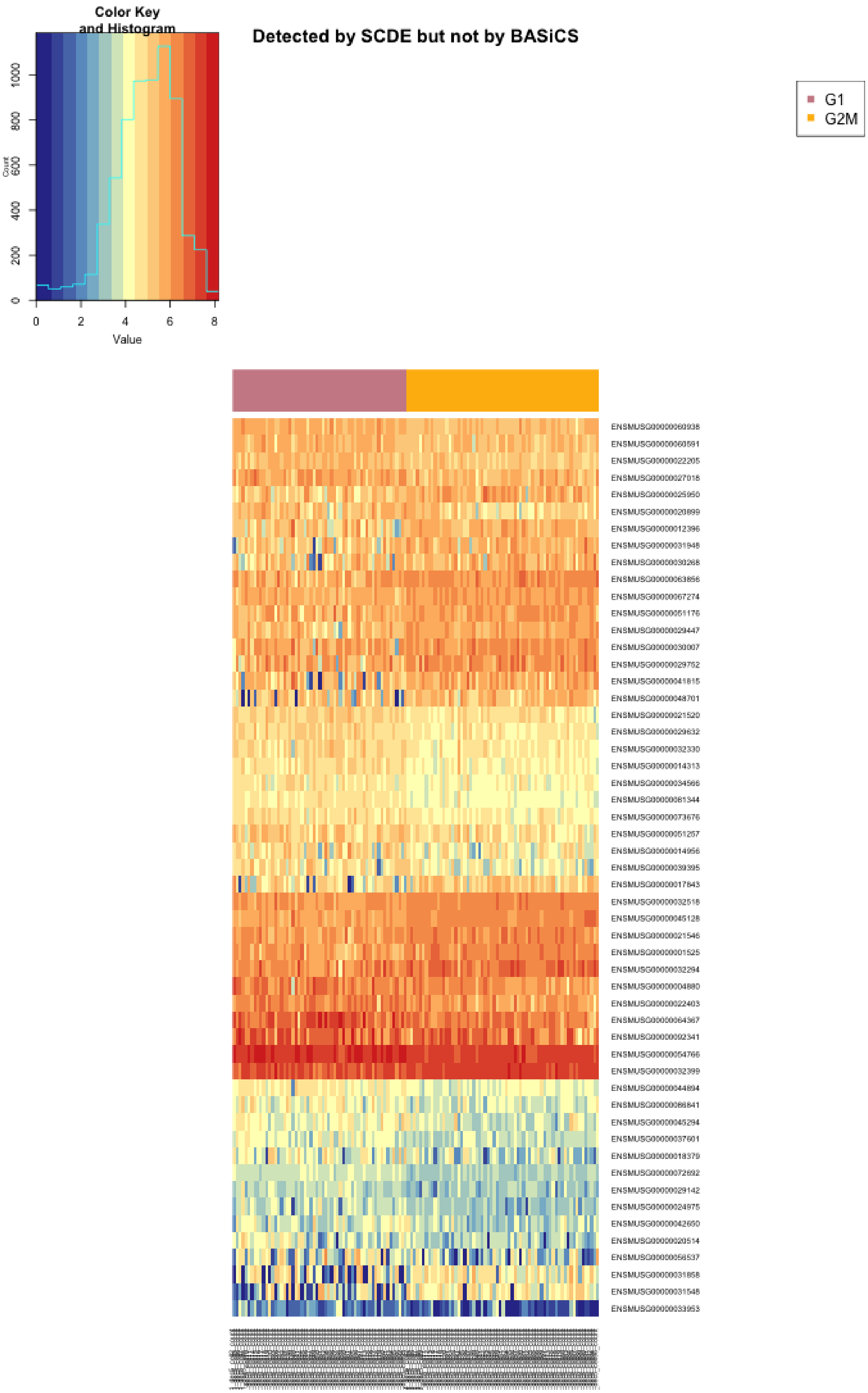
Color Key
and Histogram
Count
Value
Detected by SCDE but not by BASiCS
G1
G2M

```
HeatmapFormat(cbind(DC.S, DC.G2M)[order(Test23_40_DT_SCDE$ExpLogFC),],
              GenesSel = Test23_40_DT_SCDE$GeneNames[Test23_40_DT_SCDE$ResultDiffExp == "NoDiff" &
                             Test23_40_DT_SCDE$Result != "NoDiff"],
              ColSideColors = Cell.Colour[!grepl("G1", colnames(DC.all))],
              main = "Detected by SCDE but not by BASiCS", Rowv = TRUE)
legend('topright', c("S", "G2M"), pch = 15, col = unique(Cell.Colour)[c(2,3)])
```

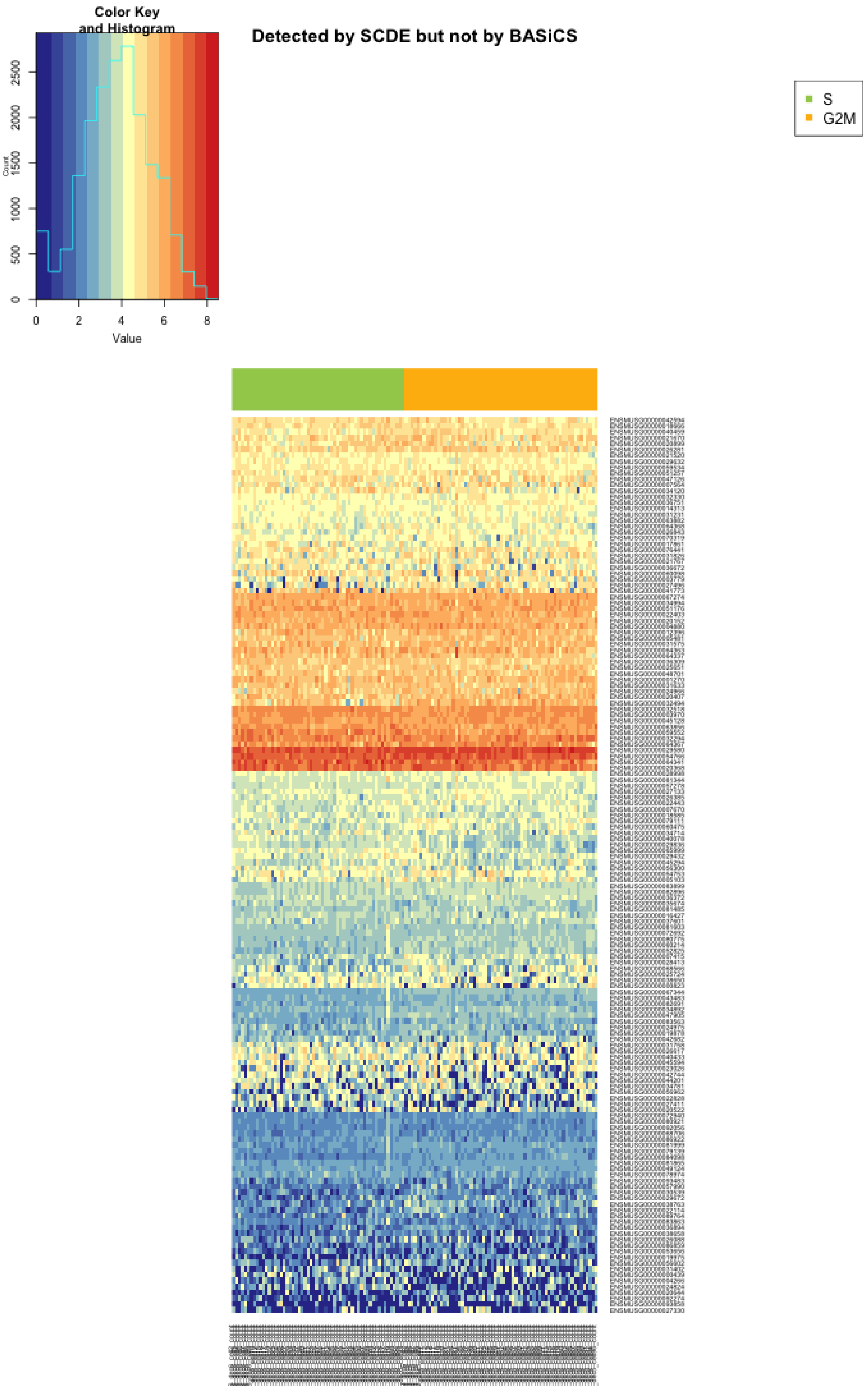
Color Key
and Histogram
Count
0
500
1000
1500
2000
2500
0
2
4
6
8
Value
Detected by SCDE but not by BASiCS
S
G2M
